# Supplementary material for: Historical overview and geographical distribution of neglected tropical diseases amenable to preventive chemotherapy in the Republic of the Congo: A systematic review
Source: PLoS Negl Trop Dis. 2022 Jul 11;16(7):e0010560. doi: 10.1371/journal.pntd.0010560 (PMC9302787; doi:10.1371/journal.pntd.0010560)
Supplement: S1 Appendix — (DOCX) [file pntd.0010560.s001.docx]

**List of abbreviations**

**Title:**

***Historical overview and geographical distribution of Neglected Tropical Diseases amenable to preventive chemotherapy in the Republic of the Congo: a systematic review***

AD: Administrative Districts

ALB: Albendazole

APOC: African Programme for Onchocerciasis Control

ATS: Alternative Treatment Strategies

CAA: Circulating Anodic Antigens

CAR: Central African Republic

CCA: Circulating Cathodic Antigens

CDTI: Community-Directed Treatment with Ivermectin

CERGEC: *Centre de Recherche Géographique et de Production Cartographique*

CFA: Circulating Filarial Antigens

CM-NTDs: Neglected Tropical Diseases amenable to Case Management

ESPEN: Expanded Special Project for Elimination of Neglected Tropical Diseases

GBD: Global Burden of Disease

GC: Geographic Coverage

HA: Health Area

HAT: Human African Trypanosomiasis

HD: Health District

ICT: Immunochromatographic card Test

IRD: *Institut de Recherche pour le Développement*

IU: Implementation Unit

IVM: Ivermectin

KfW: *Kreditanstalt für Wiederaufbau*

LF: Lymphatic Filariasis

MDA: Mass Drug Administration

MED: Major Endemic Diseases

MF: Microfilariae

NGO: Non-Governmental development Organizations

NTD: Neglected Tropical Diseases

OCCGEAC: *Organisation de Coordination et de Coopération pour la lutte contre les Grandes Endémies en Afrique Centrale*

OCEAC : *Organisation de Coordination pour la lutte contre les Endémies en Afrique Centrale*

OPC: Organisation pour la Prévention de la Cécité

PC-NTDs: Neglected Tropical Diseases amenable to Preventive Chemotherapy

PMF: Prevalence of skin Microfilariae

PNod : Prevalence of Nodules

PNLO: *Programme National de Lutte contre l’Onchocercose*

PNLSCH: *Programme National de Lutte contre la Schistosomiase*

RDT: Rapid Diagnostic Tests

REA: Rapid Epidemiological Assessment

REMO: Rapid Epidemiological Mapping of Onchocerciasis

RoC: The Republic of the Congo

SAC: School-Aged Children

SAE: Severe Adverse Events

STH: Soil-Transmitted Helminth infections

TC: Therapeutic Coverage

TF: Trachomatous inflammation-Follicular

TT: Trachomatous Trichiasis

WER: Weekly Epidemiological Record

WHO: World Health Organization
