# Supplementary material for: Historical overview and geographical distribution of neglected tropical diseases amenable to preventive chemotherapy in the Republic of the Congo: A systematic review
Source: PLoS Negl Trop Dis. 2022 Jul 11;16(7):e0010560. doi: 10.1371/journal.pntd.0010560 (PMC9302787; doi:10.1371/journal.pntd.0010560)
Supplement: S3 Appendix — (PDF) [file pntd.0010560.s003.pdf]

ORGANISATION DE COORDINATION
POUR LA LUTTE CONTRE LES ENDEMIES
EN AFRIQUE CENTRALE
(O.C.E.A.C)

CENTRE D'ENSEIGNEMENT SUPERIEUR EN SANTE PUBLIQUE
(YAOUNDE CAMEROUN)

Année Académique 1985 - 1986

L'ONCHOCERCOSE AU CONGO UNE ETUDE EPIDEMIOLOGIGUE REALISEE DANS LA REGION DU POOL

Mémoire de Fin d'Etudes en vue de l'Obtention
du Diplôme de Technicien Supérieur en Santé Publique
OPTION EPIDEMIOLOGIE APPLIQUEE

PRESENTE PAR :

Mialebama - Jean

REPUBLIQUE POPULAIRE DU CONGO

LE JURY :

Président :
Vice-Président :
Membres :

ORGANISATION DE COORDINATION
POUR LA LUTTE CONTRE LES ENDEMIES
EN AFRIQUE CENTRALE
(O.C.E.A.C)

CENTRE D'ENSEIGNEMENT SUPERIEUR EN SANTE PUBLIQUE
(YAOUNDE CAMEROUN)

Année Académique 1985 - 1986

L'ONCHOCERCOSE AU CONGO UNE ETUDE EPIDEMIOLOGIGUE REALISEE DANS LA REGION DU POOL

Mémoire de Fin d'Etudes en vue de l'Obtention
du Diplôme de Technicien Supérieur en Santé Publique
OPTION EPIDEMIOLOGIE APPLIQUEE

PRESENTE PAR :

Mialebama - Jean

REPUBLIQUE POPULAIRE DU CONGO

LE JURY :

Président :

Vice-Président :

Membres ;

DEDICACES

A LA MEMOIRE DE MON PERE

Tu nous as quitté trop prématurément, sans cueillir les fruits de ta semence. Nous gardons de toi le sens d'équité, de pondération, de charité et de courage. Puissent ces nobles principes nous guider tout au long de notre carrière.
Que la terre te soit légère.

A LA MEMOIRE DE MA GRAND-MERE

Pour le magnifique exemple de courage et de dignité que tu représentais pour nous.
Dieu t'a appelé à mon absence, tu es partie sans une aide quelconque de ma part.
Mes regrets éternels. Que le tout Puissant veille sur ton âme pour l'éternité.

A MA MERE KOUTALOU THERESE

- Pour ton dévouement et tes conseils
- Pour toute l'attention et tous les sacrifices que tu as consentis pour tes enfants.
- Pour toutes les peines que nous t'avons faites, ton calvaire n'a pas été vain, les fruits de tes entrailles émergent. Puisse Dieu t'accorder une longue vie.

A MA FEMME ALPHONSINE MILANDOU

- Pour ton dévouement et ton courage
- Pour ta compréhension, ton aide morale et matérielle mes remerciements et mon admiration.

  En témoignage de mon profond amour
  Tu as su supporter seule pendant toute la durée de ma formation , ce lourd fardeau que constitue l'encadrement de nos enfants. Ce travail est aussi le tient et je ne doute pas que tu partages ma satisfaction.

A MES ENFANTS

Que ce modeste travail vous serve d'exemple de courage, d'endurance, d'abnégation et de persévérance. Qu'il vous inspire à aller au delà de ce que j'ai fait pour votre vie future. En témoignage de mon amour paternel.

A MES FRERES

NGANGA - JOACHIM
BIKOYI DOMINIQUE

- Pour votre soutien moral et vos encouragements Ce travail est aussi le votre, qu'il vous donne la joie. Puissiez-vous comprendre que rien n'est plus grand et plus noble que ce qu'on gagne à la sueur de son front. En témoignage de mon affection fratenelle.

REMERCIEMENTS

L'appui et l'assistance dont nous avons bénéficié nous ont permis de réaliser l'Etude dont les résultats sont rapportés ici . Nous tenons tout particulièrement à exprimer notre profonde gratitude aux :

Docteur GABRIEL MADZOU Directeur de la Médecine Préventive (Brazzaville)

Docteur PIERRE EOZENOU Médecin Chef du service de l'Epidémiologie et des grandes Endémies (Brazzaville) pour nous avoir accordé la permission et les moyens matériels nécessaires à la réalisation de notre enquête.

- Nous remercions également Monsieur NTSOUMOU MADZOU Victor et Mr. MILONGO Gaston pour leur aide à la réalisation de notre enquête.

- Nos remerclements vont également à l'endroit de nos professeurs tant missionnaires que permanents et à nos encadreurs comme :

Professeur DALMEIDA (Togo)
Dr. ONDAYE (Congo)
Mr. KINDE (Tchad)
Mr. KAKUE
Mr. POUGOUE
Mr. SOH

qui ont fait preuve de beaucoup de sacrifices pour notre formation.

Ainsi qu'à tout le personnel de l'OCEAC pour leurs divers services rendus durant notre séjour de formation dans cet organisme.

A Mme NOUMSSI Madeleine, Secrétaire dactylographe au CUSS de Yaoundé quia. bien voulu accepter la dactylographie de ce travail : nos sincères remerciements.

NOS RESPECTUEUX HOMMAGES

Au Docteur DANIEL- KOUKA-BEMBA Secrétaire Général de l'OCEAC

Au Docteur V. MOUANDA Secrétaire Général Adjoint de l'OCEAC

Au Docteur R. JOSSERAN Directeur du Centre d'Enseignement Supérieur en Santé Publique (OCEAC - Yaoundé)

Au Docteur P. AMBASSA Directeur Adjoint du Centre d'Enseignement Supérieur en Santé Publique (OCEAC - Yaoundé)

Au Docteur MERLIN Chef du service de l'Epidémiologie (OCEAC-Yaoundé)

Au Docteur LE MAO Chef du service Technique de l'OCEAC (Yaoundé)

Au Docteur JOSSE Adjoint au Chef de Service Technique de l'OCEAC (Yaoundé)

Au Docteur QUILLEVERE (ORSTOM- Yaoundé)

Pour tous vos sages conseils et vos nobles connaissances que vous avez déversés en nous. Vous avez su faire de nous des hommes utiles à l'humanité et surtout à notre pays .

Veuillez trouver ici l'expression de notre très profonde gratitude.

LA VIE EST UN COMBAT
CEUX QUI VIVENT SONT CEUX QUI LUTTENT

VICTOR HUGO

SOMMAIRE

$$$$$$$$$$$$

- INTRODUCTION ET OBJECTIFS — Pages

1 - INTRODUCTION 1
2 - OBJECTIFS 3

CHAPITRE I — CADRE DU TRAVAIL

1-1 LA REPUBLIQUE POPULAIRE DU CONGO ... 5
1-1-1 - Situation géographique ... 5
1-1-2 - Etude physique 5
1-1-2-1 - Relief 5
1-2-2 - Végétation 7
1-2-3 - Hydrographie 8
1-2-4 - Climat 9
1-2-5 - Population 9
1-2-6 - Divisions administratives 11
1-2-7 - Voies de communications. 11
1-2-8 - Economie du Congo 13
1-2-9 - Divisions sanitaires ... 13

1-2 LA REGION DE L'ETUDE 14
1-2-1 - Présentation 14
- La région du Pool 14
1-2-2 - Le District de Boko ... 17

../..

CHAPITRE II Pages

2-1 GENERALITES SUR LES FILARIOSES HUMAINES ... 19

2-1-1 - Rappel Général 19
2-1-2 - Classification 19
2-1-3 - Répartition géographique .. 21

2-2 L'ONCHOCERCOSE 22

2-2-1 - Définition 22
2-2-2 - Rappels sur l'aspect épidémiologique 22
2-2-3 - Rappels sur la clinique 25
2-2-4 - Diagnostic Biologique 27

2-3 L'ONCHOCERCOSE AU CONGO 27
- Historique 27

CHAPITRE III METHODOLOGIE

3-1 ENQUETE RETROSPECTIVE 31

3-1-1 - Etudes anciennes sur l'onchocercose dans la région du Pool..... 31
3-1-2 - Résultats obtenus 32
3-1-3 - Résultats par zone 36

3-2 ENQUETE PROSPECTIVE 52
3-2-1 - Déroulement de l'enquête 52
3-2-2 - Personnel 52
3-2-3 - Matériel 52
3-2-4 - Méthodes et techniques 53

../..

CHAPITRE IV RESULTATS

Pages

4-1 RESULTATS GLOBAUX 56

4-2 - Présentation et Analyse des Résultats par Foyer. 64

4-2-1 - Foyer de Foota 64
4-2-2 - Foyer de Mantaba 72
4-2-3 - Foyer de Kimpenga 78
4-2-4 - Foyer de Bela 85
4-2-5 - Foyer de Mandombé 91

CHAPITRE V ... COMMENTAIRES ET DISCUSSIONS 97

5-1 - Sur le plan clinique 97
5-2 - Sur le plan parasitologique ... 102
5-3 - Relation entre la parasitose onchocerquienne et les manifestations cliniques 106
5-5 - Considérations générales de l'endémie onchocerquienne dans la région du Pool 112
5-6 - Considérations épidémiologiques. 114

CHAPITRE VI CONCLUSION ET SUGGESTIONS ... 117

6-1 - Conclusion 117
6-2 - Suggestions 119

CHAPITRE VII REFERENCES BIBLIOGRAPHIQUES 121

ANNEXES

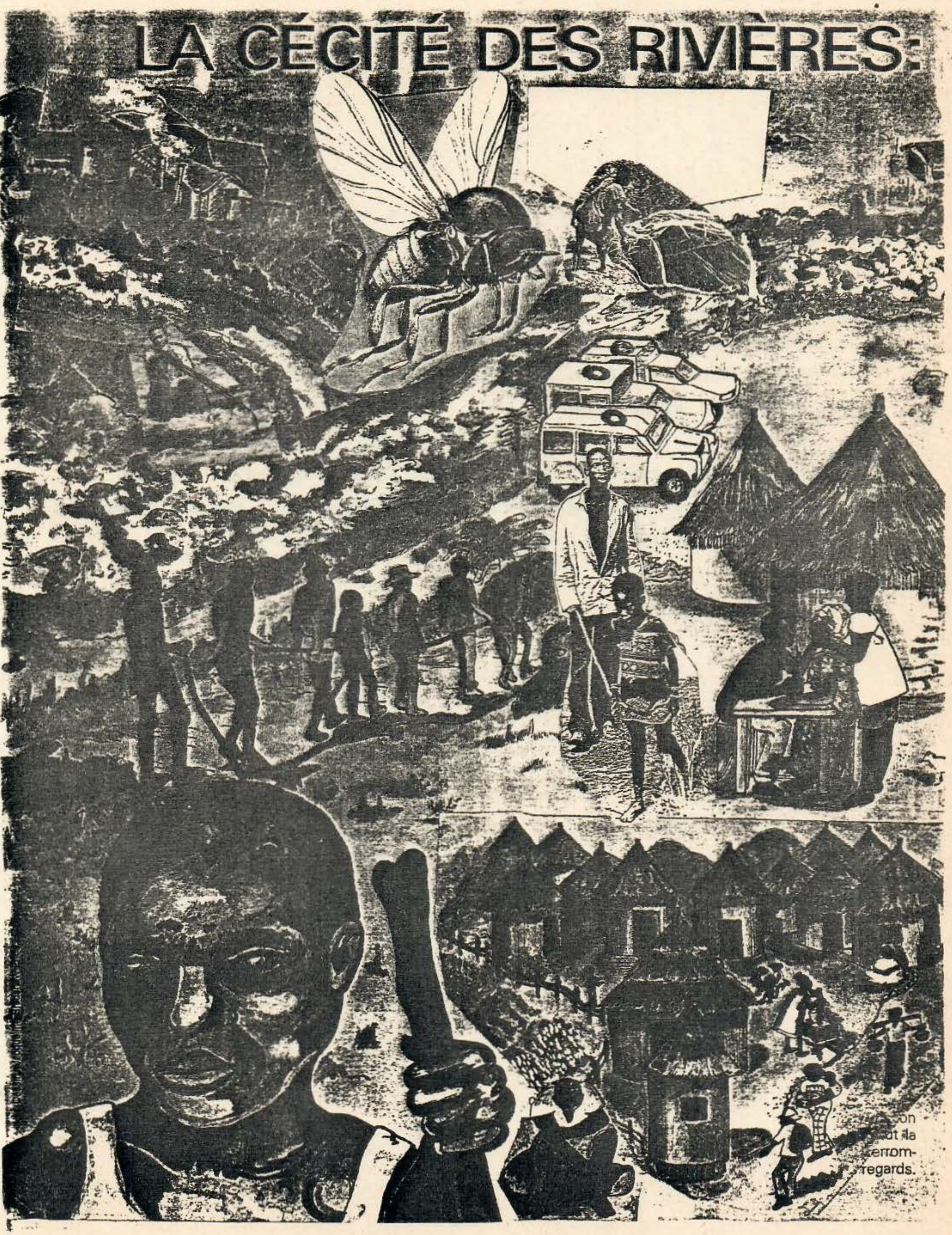

Ces Images symbolisent la gravité et le danger de L'ONCHOCERCOSE dans les Zones exposées à une forte endémicité.

INTRODUCTION

INTRODUCTION

L'Onchocercose ou volvulose, encore appelée autrefois "Cécité des rivières" est une maladie parasitaire, à effet cumulatif, essentiellement rurale, transmise à l'homme par la piqure des simulies. C'est une filariose due à Onchocerca-volvulus. Elle est caractérisée par des manifestations cutanéo-dermiques et des lésions oculaires pouvant aboutir à la cécité.

Sa distribution géographique dépend de celle du vecteur. En Afrique tropicale, tous les pays sont touchés, entre la 12e latitude Nord et la 15e latitude Sud.

- En Août 1959, les experts de l'OMS réunis à la Conférence de Genève estimaient à plus de 20 millions le nombre de personnes atteintes par cette maladie. (1)

- Actuellement d'après les estimations de l'OMS, ce nombre est passé à plus de 30 millions (2). Et l'Afrique seule compte près de 99 % des cas.

Rien qu'en 1952, 500.000 cas ont été dépistés en Afrisue (3) c'est de surcroît la deuxième cause mondiale de cécité après le trachome.

Dans les villages dits "de première ligne", situés à proximité des gîtes larvaires, on peut voir jusqu'à 30 % d'handicapés visuels

../..

et 1,5 à 10 % d'aveugles.

Cette affection pose des problèmes socio-économiques de première importance dans les Etats concernés.

- l'Organisation Mondiale de la Santé (OMS) en a souligné la gravité depuis 1953 date à laquelle une réunion d'Experts à Mexico revelait qu'il s'agissait d'un véritable fléau par son incidence sur l'économie et ses tragiques conséquences à la fois sur le malade et sur la collectivité.

- Aussi, ce fléau social n'épargne pas la République Populaire du Congo notre pays, moins encore la région du Pool.

- En effet, l'existence de l'onchocercose dans la région du Pool n'est plus à démontrer d'autant plus que les tous premiers cas d'onchocercose humaine au Congo ont été signalés dans cette région entre 1919 et 1921 par LEBOEUF (in OUZILLEAU et al), le long des rivières Djoué et la Foulakari. Puis par OVAZZA en 1953, au cours d'un sondage dans cette même région. Ces Etudes ont été complétées par les travaux de YEBAKIMA (1978-1982).

Cependant, on ne dispose pas d'informations assez précises sur la prévalence de la maladie dans les populations humaines de cette région, et l'épidémiologie de la maladie demeure encore quelque peu mal connue d'une part. Et d'autre part, la plupart des villages de cette région n'ont jamais été prospectés.

../..

- Face à cette situation, nous avons été amenés dans le cadre de notre formation à travailler sur l'etude épidémiologique de cette effection et sur l'évolution des différents foyers d'onchocercose existant dans la région du Pool.

- Notre travail dont le titre est intitulé : "L'Onchocercose au Congo : une étude épidémiologique réalisée dans la région du Pool", a pour buts :

1°) De compléter les quelques travaux déjà effectués sur l'onchocercose dans la région du Pool, notamment par : OVAZZA (1953) - RICHET (1953) - GILLES (1960) et YEBAKIMA (1978-1982).

2°) De faire le point des connaissances actuelles sur la prévalence de la maladie au sein de la population humaine et de déterminer les charges parasitaires des individus.

2 - OBJECTIFS

OBJECTIFS GENERAUX

1°) Déterminer la Prévalence de l'Onchocercose dans la région du Pool par la Recherche systématique des Onchocuquiens dans la population.

2°) Préciser l'Endémicité de l'infestation dans la Région.

3°) Formuler des suggestions nécessaires pour améliorer la situation.

../..

OBJECTIFS SPECIFIQUES

Nous tenterons de :

1°) Identifier l'impact de la maladie sur la santé des communautés exposées.

2°) Délimiter les principaux foyers d'onchocercose connus dans la région du Pool afin de compléter la cartographie de l'onchocercose de la République Populaire du Congo.

3°) Préciser le faciès épidémio-clinique de l'onchocercose dans cette zone par l'identification des tranches d'âges les plus touchées.

CHAPITRE I

CADRE DU TRAVAIL

CHAPITRE I

CADRE DU TRAVAIL

1 - LA REPUBLIQUE POPULAIRE DU CONGO

1-1 SITUATION GEOGRAPHIQUE

- La République Populaire du Congo qui a pour Capitale BRAZZAVILLE est située en Afrique Centrale à cheval sur l'Equateur. Elle est comprise entre le 5e degré de latitude Sud et le 3e degré de latitude Nord puis entre le 11e degré de longitude Ouest et le 18e degré de longitude Est.

-Elle s'ouvre à l'Océan Atlantique à partir d'une étroite façade maritime d'environ 170 Km.

Elle est limitée au Nord par le Cameroun et la République Centrafricaine. Au Sud par la Province Angolaise du Cabinda et le Zaïre. A l'Ouest par le Gabon. Au Sud-Ouest par l'Océan Atlantique et à l'Est par les Fleuves Congo et l'Oubangui qui font une frontière naturelle avec le Zaïre. La République Populaire du Congo couvre une superficie de 342 000 km^2 avec une population de 2.202.000 habitants (d'après le recensement de 1984) soit une densité de 6,4 habitants au Km^2.

1-2 ETUDE PHYSIQUE

1-2-1 RELIEF

Le relief et la structure de la République Populaire du Congo sont variés. On y rencontre :

../..

<u>La Plaine Cotière</u>, basse, marécageuse ou tapissée d'alluvions se termine par un littoral sablonneux et rectiligne d'accès malaisé. Vers l'intérieur, se trouvent quelques massifs montagneux ne dépassant pas 1500 mètres. (3)

<u>Le Massif du Mayombe</u> à l'Est de Pointe-Noire.

<u>Le Massif du Chaillu</u>, près de la frontière du Sud-Gabon.

- Au Nord, les altitudes s'abaissent vers la cuvette Congolaise. entre les vallées de la Sangha et de l'Oubangui.

La République Populaire dù Congo est divisée en deux ensembles principaux par une ligne passant par BZAZZAVILLE. (3)

- Le Congo septentrional et le Congo Sud-Occidental.

<u>Le Congo Septentrional</u>

Situé au Nord de la capital, il s'étend sur 250 000 km^2 et se compose de :

- La cuvette Congolaise
- La Sangha Occidentale
- Et le pays Batéké.

<u>Le Congo Sud-Occidental</u> :

Il couvre entre Brazzaville et la Côte de l'Océan Atlantique environ 100.000 km^2 et se compose de :

- La région du Pool "ou région des cataractes"
- Les pays du Niari et de la Nyanga avec le massif du Chaillu.

../..

- La vallée du Niari à roches schisto-calcaires.
- La façade maritime du Congo avec le Massif du Mayombe et le bassin de Pointe-Noire.

1-2-2 - Végétation

Deux principaux types de végétation se partagent le territoire Congolais ; la forêt et la savane.

La Forêt :

Elle occupe plus de la moitié du pays ; environ 60 % de la Superficie totale, avec une grande variété d'arbres. Elle se répartit en trois ensembles importants qui sont :

- La forêt du Mayombe
- La forêt du Chaillu
- La forêt du Nord Congo correspondant à la grande forêt équatoriale. Elle couvre la plus vaste superficie avec une partie de la cuvette Congolaise au Nord-Est entre la Sangha et l'Oubangui qui est occupée par des Zones marécageuses avec forêts inondées.(3)

La Savane

C'est un paysage végétal où dominent les herbes, mais n'exclu pas les arbres comme les fromagers ou les boquêtaux bien fournis. L'aspect de la Savane aussi n'est pas toujours le même. Elle est (la savane) faiblement arborée et couvre environ 40 % de la superficie totale du pays et se compose de :

- les savanes arbustives
- les savanes nues
- les galeries forestières qui bordent souvent chaque cours d'eau et remontent le long des petits affluents.

La savane couvre principalement la partie méridionale du pays.

1-2-3 HYDROGRAPHIE

- En totalité ou en partie, la République Populaire du Congo possède deux grands bassins fluvieux.

- Celui du Congo prolongé par l'Oubangui, et celui du Kouilou-Niari, qui constituent un régime complexe.

Entre Brazzavile (capital de la République Populaire du Congo et KINSHASSA (Capital de la République du Zaïre), le fleuve Congo s'étale pour former "Le Stanley-Pool". Son centre est occupé par l'Ile MBAMOU.

En Aval de Brazzaville, le fleuve Congo s'encaisse dans le plateau gresseux des cataractes et son cours est coupé de puissants rapides qui rendent toute navigation impossible.

Le fleuve Congo a des variations de débits peu importantes avec en hautes Eaux un débit de 61.170 m^3 par seconde et un débit de 33.250 m^3/ seconde en période de basses Eaux (relevés de 1980, d'après les archives de l'ORSTOM). Ces débits font du fleuve Congo le second fleuve du Monde après l'AMAZONE.

En dehors de ces grands bassins, il existe de nombreux autres cours d'eau à régime simple. Exemple : L'Alima, la Léfini, La Nkeni, la Likouala-Mossaka, la Sangha, Le Djoué, la Loufoulakari, la Louvoubi, la Louholo etc. Ainsi que quelques lacs tels que : le lac Cayo, le Lac Nanga, le Lac télé et la Lagune de Conkouati. (3)

../..

CARTE N°1

REPUBLIQUE POPULAIRE DU CONGO

---------0---------

Limite - Relief - Hydrographie

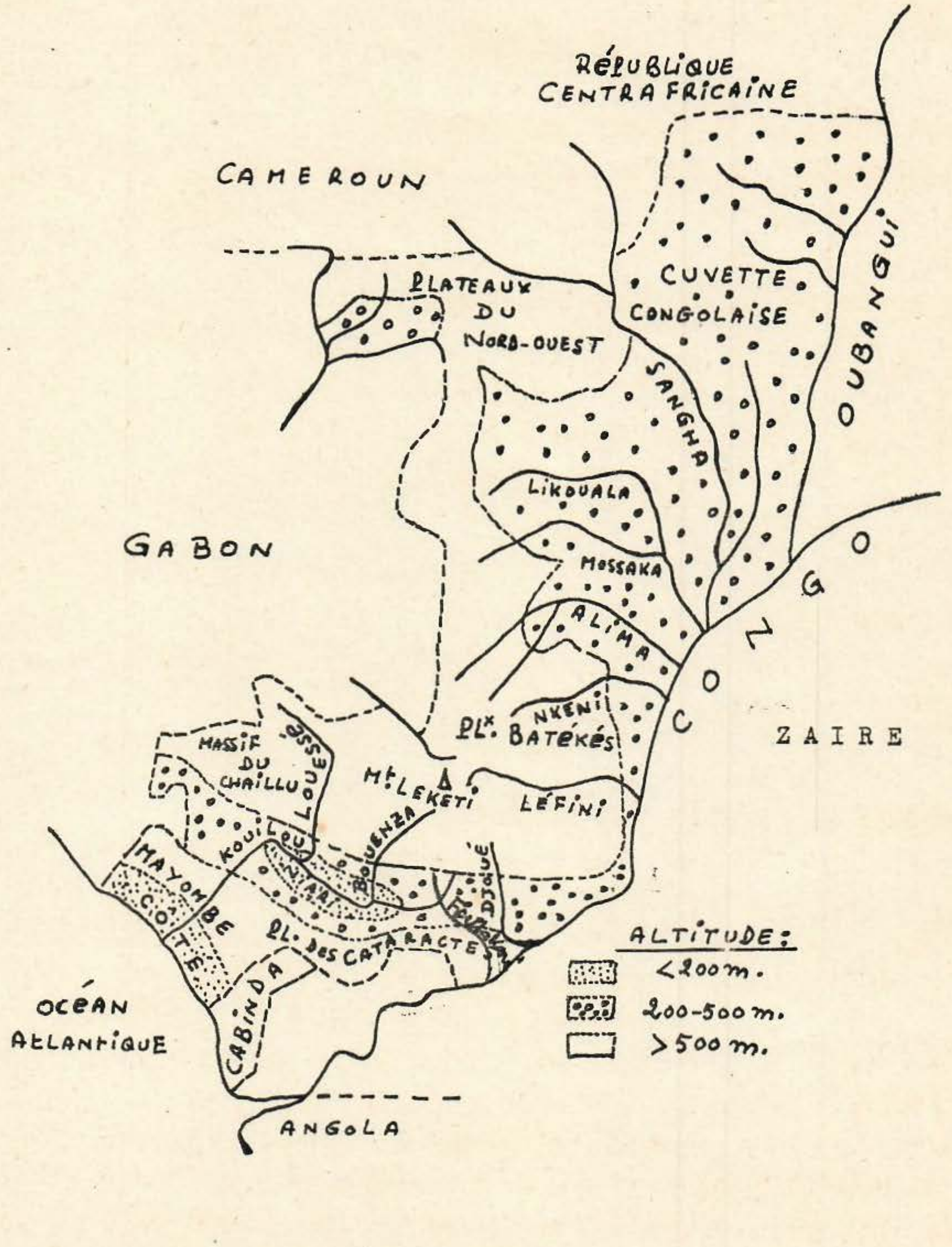

1-2-4 CLIMAT

La République Populaire du Congo est entièrement comprise dans la Zone Equatoriale donc soumise à un climat chaud et humide, et à des fortes pluies.

Ce climat présente néanmoins des différences sensibles en raison de l'allongement du pays le long des méridiens.

- On distingue alors deux saisons principales et deux saisons intermédiaires.

- La grande saison des pluies de Mars à Mai avec des températures élevées 25 à 30° et des précipitations abondantes comprises entre 1800 et 2100 mm d'eau de pluie par an.

- La grande saison sèche qui va de Juin à Septembre.

- La petite saison des pluies : d'Octobre à Décembre

- La petite saison sèche de Janvier à Février.

1-2-5 POPULATION

La République Populaire du Congo compte environ 2.202.000 habitants (d'après le recensement de 1984). Le Congo est un pays encore sous- peuplé avec une dansité d'environ 6,4 habitants au Km^2. Cette population est inégalement répartie. Les six communes que compte le pays :

Brazzaville (capital politique et administrative)

Pointe-Noire (capitale économique)

Nkayi-Loubomo-Mossendjo et Ouesso rassemblent

../..

dans leurs agglomérations plus de 800 000 habitants soit plus du tiers de la population totale du pays.

- En dehors de ces six communes urbaines, le pays compte une quarantaine d'autres centres de plus de 2.000 habitants dont KINKALA qui est le Chef lieu de la Région du Pool (notre Zone d'Etude).

- Ces centres qui ne sont pas de communes urbaines se distinguent des autres villages qui sont très nombreux et aussi très éparpillés, par leur structure démographique.

ETHNIES

Malgré ce faible peuplement, la diversité ethnique est très grande. On y trouve trois grands groupes ethiniques, et en dehors des pygmés, le reste de la population congolaise est de race Bantou (environ 98 %) comprenant :

- Le groupe Kongo 48 % (avec ses sous-groupes)
- Le groupe Téké 22 %(avec ses sous-groupes)
- Le groupe Mbochi 13 %(avec ses sous-groupes)

En plus du français qui est reconnu comme langue officielle, les congolais utilisent deux langues vernaculaires (ou langues véhiculaires) qui sont : le Lingala et le Mounoukoutouba ou Kikongo.

CROYANCES

On trouve au Congo des diverses croyances et religions telles que : le salutiste et de nombreux autres sectes ainsi que des animistes. Le catholicisme, le protestantisme, le kimbanguisme, le salutiste et de nombreux autres sectes, ainsi que des animistes.

1-2-6 DIVISIONS ADMINISTRATIVES

La République Populaire du Congo est gouvernée par un Président de la République qui est Président du Comité Centrale du Parti, Chef de l'Etat et Président du Conseil des Ministres .

_ Elle est divisée (la République Populaire du Congo) en 10 régions administratives et politiques. Elles mêmes (les régions) divisées en communes, districts et Postes de contrôles administratives (P C A)

- La Région est l'Unité administrative. Chaque région est dirigée par un commissaire politique.

- La République Populaire du Congo compte en outre 46 Districts et 30 Postes de Contrôles administratifs. (P C A) ;

- Les Districts sont dirigés par les Chefs de Districts, et les Postes des contrôles administratifs (P C A) par les Chefs de (P C A)

1-2-7 VOIES DE COMMUNICATION

Les infrastructures de transport et moyens de communication au Congo comprennent :

- Le Réseau Ferroviaire

Le chemin de fer Congo Océan (C F C O) long de 510 km construit en 1921 jusqu'à 1934 assure les échanges économiques et humaines entre Brazzaville et Pointe-Noire.
Un réalignement du C F C O vient d'être réalisé pour une meilleure traversée du Mayombe.

../..

CARTE N°2

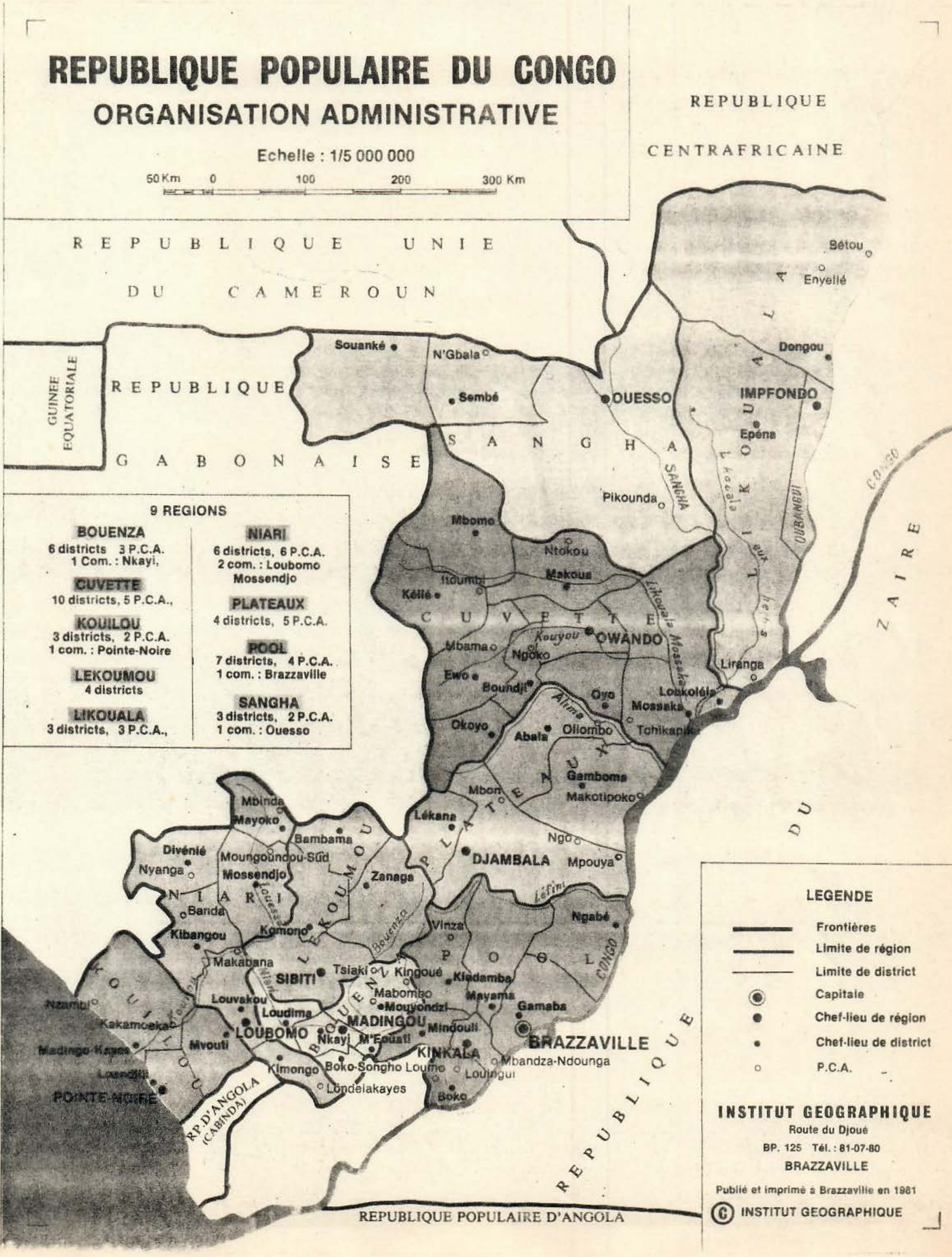
REPUBLIQUE POPULAIRE DU CONGO
ORGANISATION ADMINISTRATIVE
Echelle : 1/5 000 000
50 Km 0 100 200 300 Km
REPUBLIQUE CENTRAFRICAINE
REPUBLIQUE UNIE DU CAMEROUN
GUINEE EQUATORIALE
REPUBLIQUE GABONAISE
REPUBLIQUE DU ZAIRE
REPUBLIQUE POPULAIRE D'ANGOLA
R.P. D'ANGOLA (CABINDA)
9 REGIONS
BOUENZA
6 districts 3 P.C.A.
1 Com. : Nkayi,
CUVETTE
10 districts, 5 P.C.A.,
KOUILOU
3 districts, 2 P.C.A.
1 com. : Pointe-Noire
LEKOUMOU
4 districts
LIKOUALA
3 districts, 3 P.C.A.,
NIARI
6 districts, 6 P.C.A.
2 com. : Loubomo
Mossendjo
PLATEAUX
4 districts, 5 P.C.A.
POOL
7 districts, 4 P.C.A.
1 com. : Brazzaville
SANGHA
3 districts, 2 P.C.A.
1 com. : Ouesso
Bétou
Enyellé
Dongou
IMPFONDO
Epéna
Souanké
N'Gbala
Sembé
OUESSO
Pikounda
Mbomo
Ntokou
Makoua
Itoumbi
Kéllé
OWANDO
Mbama
Ngoko
Ewo
Boundji
Oyo
Loukoléla
Mossaka
Tchikapika
Okoyo
Abala
Ollombo
Gamboma
Mbon
Makotipoko
Lékana
Ngo
DJAMBALA
Mpouya
Mbinda
Mayoko
Bambama
Divénié
Nyanga
Moungoundou-Sud
Mossendjo
Zanaga
Banda
Kibangou
Komono
Vinza
Ngabé
Makabana
SIBITI
Tsiaki
Kingoué
Kindamba
Mabombo
Mouyondzi
Mayama
Gamaba
Louvakou
Loudima
MADINGOU
Mindouli
LOUBOMO
Nkayi
M'Fouati
Mvouti
KINKALA
BRAZZAVILLE
Mbandza-Ndounga
Louingui
Kimongo
Boko-Songho
Loumo
Londelakayes
Boko
Nzambi
Kakamoeka
Madingo-Kayes
Loandjili
POINTE-NOIRE
SANGHA
CUVETTE
PLATEAUX
LEKOUMOU
NIARI
BOUENZA
POOL
KOUILOU
LIKOUALA
CONGO
OUBANGUI
LEGENDE
Frontières
Limite de région
Limite de district
Capitale
Chef-lieu de région
Chef-lieu de district
P.C.A.
INSTITUT GEOGRAPHIQUE
Route du Djoué
BP. 125 Tél. : 81-07-80
BRAZZAVILLE
Publié et imprimé à Brazzaville en 1981
© INSTITUT GEOGRAPHIQUE

La COMILOG longue de 285 km entre Mbinda et Mont-belo assure l'évacuation du manganèse gabonais.

Le Réseau Fluvial

Entre Brazzaville (capitale de la République Populaire du Congo et Kinshassa (capitale du Zaïre) le fleuve congo s'étale et forme une immense étendue : "Le Stanley-Pool". A cet endroit se fait la traversée du fleuve pour des divers échanges entre les deux capitales.

- En amont du Stanley-Pool, le Congo et ses affluents de la rive droite permettent de pénétrer au coeur du Congo Septentrional et d'atteindre les pays voisins comme la République Centrafricaine. Ils représentent 3705 km de voies navigables entre le port de Brazzaville et ceux de Boundji-Mossaka-Owando-Makoua et Ouesso.

Le Réseau Maritime

- Pointe-Noire est un complexe maritime comprenant le Port public et le port terminal pétrolier de Ndjeno.

Le Réseau Routier

Longtemps rudimentaire, il est actuellement en pleine amélioration et comprend deux axes principaux. La Route Nationale N° 1 qui relie Brazzaville à Pointe-Noire et la route Nationale N° 2 reliant elle, Brazzaville avec le Nord du Pays jusqu'à Ouesso. (23)

Le Réseau Aérien

Il tient à une vingtaine de terrains d'atterrissage et deux aéroports internationaux accessibles aux avions long courrier et gros porteurs. Là Brazzaville et à Pointe-Noire.

Le Réseau de Télécommunication

Encore insuffisant, il est assuré par des liaisons radio-téléphoniques automatiques grâce à deux faisceaux hertziens (Nord et Sud-Ouest) et à une station terrienne de communication par satellite.

1-2-8 ECONOMIE DU CONGO

L'économie du Congo basée sur :

1°) L'exploitation forestière née vers 1930 avec l'exploitation de l'Okoumé fournit des forts revenus au pays ;

2°) Depuis 1973, la République populaire du Congo est devenu producteur du pétrole.

3°) Le sous-sol encore incomplètement prospecté regorge de minerais encore non exploité comme le fer de Zanaga et de Sembé, le zinc, le plomb à Mfouati

4°) L'Agriculture est de type rural, traditionnel. Les principalES cultures vivrières sont : le manioc, l'igname, la banane, l'arachide, le maïs, le riz, la pomme de terre. Les cultures commerciales sont peu importantes : café, cacao, huile de palme, canne à sucre.(3)

1-2-9 DIVISIONS OU STRUCTURES SANITAIRES

L'Organisation sanitaire au Congo est sous l'autorité du Ministre de la Santé et des Affaires Sociales. La République populaire du Congo est divisée en 10 régions sanitaires ou circonscription sanitaires. Elle suit (l'organisation sanitaire) la division administrative avec à la tête de chaque région sanitaire un médecin chef qui est directeur régional de la Santé Publique.

../..

CARTE N° 3

REPUBLIQUE POPULAIRE DU CONGO

ORGANISATION SANITAIRE

REPARTITION DES SECTEURS OPERATIONNELS

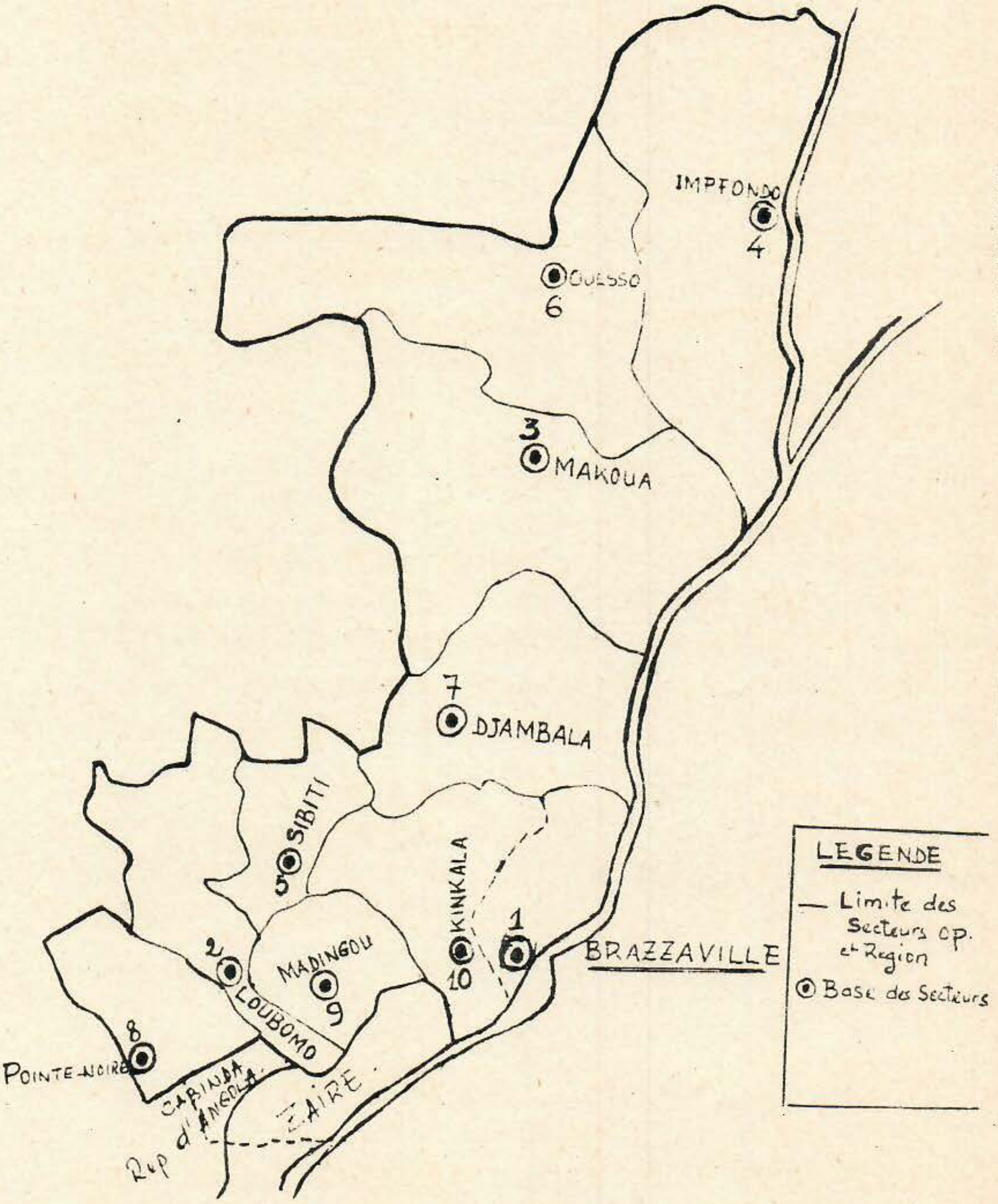

La République populaire du Congo compte trois hôpitaux généraux dont : 1 à Brazzaville
1 à Pointe-Noire
1 à Owando

1 hôpital secondaire à Loubomo.

Des centres hospitaliers à Brazzaville et à Pointe-Noire ainsi que dans chaque chef-lieu de région, et des centres médicaux dont 1 dans chaqueçhef lieu de District, ainsi que de nombreux dispensaires.

- Elle compte (La R P C) en outre 10 secteurs opérationnels. Chaque secteur est dirigé par un médecin Chef du secteur (voir carte N° 3).

D'après les archives de 1984 du Ministère de la Santé : La République Populaire du Congo à :

- un taux de natalité de 4,28 %
- un taux de mortalité de 1,59 %
- un taux de mortalité infantile de 1,40 %
- un taux d'accroissement naturel de 2,69 %

Et le rapport d'activité de 1983 DE L'UNICEF à Brazzaville signale que 65 % de la population a accès aux services de Santé en République populaire du Congo.

1-2 <u>LA REGION DE L'ETUDE</u>

2-2-1 <u>PRESENTATION</u>

<u>La Région du Pool</u> : Chef lieu Kinkala est l'une des dix régions que compte la République populaire du Congo. Elle est limitée au Nord par la Léfini (cours d'eau) et la région des plateaux, au Sud-Est par le fleuve Congo et la République du Zaïre au Sud Ouest par la Région de la Boueza et à l'Ouest par la région de la Lékoumou.

- Elle couvre une superficie de 35.000 km^2 soit environ 1/10 de la superficie totale du Congo et compte : 7 Districts qui sont :

1 District de Boko

2 District de Kinkala

3 District de Mindouli

4 District de Ngabé

5 District de Goma tsé-tsé

6 District de Kindamba

7 District de Mayama

1 (une commune qui est Brazzaville (capitale de la République populaire du Congo). Il convient de signaler que Brazzaville est devenue une région autonome administrativement et politiquement parlant depuis quelques années .

- Et 4 (quatre) P C A (postes de contrôles administratifs)
- P C A de Louingui
- P C A de Mbandza-Ndounga
- P CA de loumo
- P C A de Vindza

La région du Pool anciennement appelée"plateau des cataractes" est découpée tout au début du district de Boko en longues échines sableuses . où s'ouvrent des cirques dues aux érosions, des vallées à fond plat où coulent des rivières. Il a été ainsi transformé (le plateau des cataractes) en une série de collines par des affluents du fleuve Congo.

- La région du Pool est donc baignée par le fleuve Congo et certains de ses affluents comme : Le Djoué, la Foulakari, la Louvoubi, la Nkissi etc. Ainsi que par d'autres grands cours d'eau comme la Louholo.

- En aval du "Stanley Pool" (à Brazzaville) le fleuve Congo s'encaisse alors dans le dit "plateau des cataractes", et son cours est entrecoupé de puissants rapides qui rendent toute navigation impossible.

- Aussi, la puissance du fleuve et la profondeur de ses gorges obligent ses affluents à former également des rapides et des chutes. Exemple : les chutes de

../..

la Foulakari à Kimpandzou (District de Boko) et les chutes de la Louvoubi à Bela (District de Boko)

- Toujours en aval du "stanley-Pool" et au delà des cataractes, le fleuve Congo est par endroit un véritable rapide étroit qui gagne l'Océan en franchissant 32 chutes.

- Le cours du Congo (fleuve) est encaissé de 80 à 150 mètres entre deux talus, parfois même entre deux falaises véritables : Exemple : à bela et à Mandombé dans le district de boko.
Deux de ses affluents : La Foulakari et la Louvoubi dans le district de Boko le rejoignent par cascade.

- La région du Pool est soumise à un climat de type "bas congolais".
La Pluviométrie est de l'ordre de 1400 à 1500 mm
L'humidité de l'air est toujours importante (> 70 %)

Le sol dont la couleur est brun-rouge, tranche sur le vert de la savane, les côtes dépassent les 600 mètres.

La population totale de la région du Pool est de 180.051 habitants (récensement de 1984) repartie comme suit :

District de Boko	= 26.696 habitants
District de Kinkala	= 43.343 habitants
District de Mindouli	= 36.552 habitants
District de Ngabé	= 11.018 habitants
District de Goma-tsé-tsé	= 35.379 habitants
District de Kindamba	= 22.154 habitants
District de Mayama	= 4.909 habitants

99 % environ de cette population appartient au groupe ethnique Kongo.

../..

CARTE N° 4

REGION DU POOL

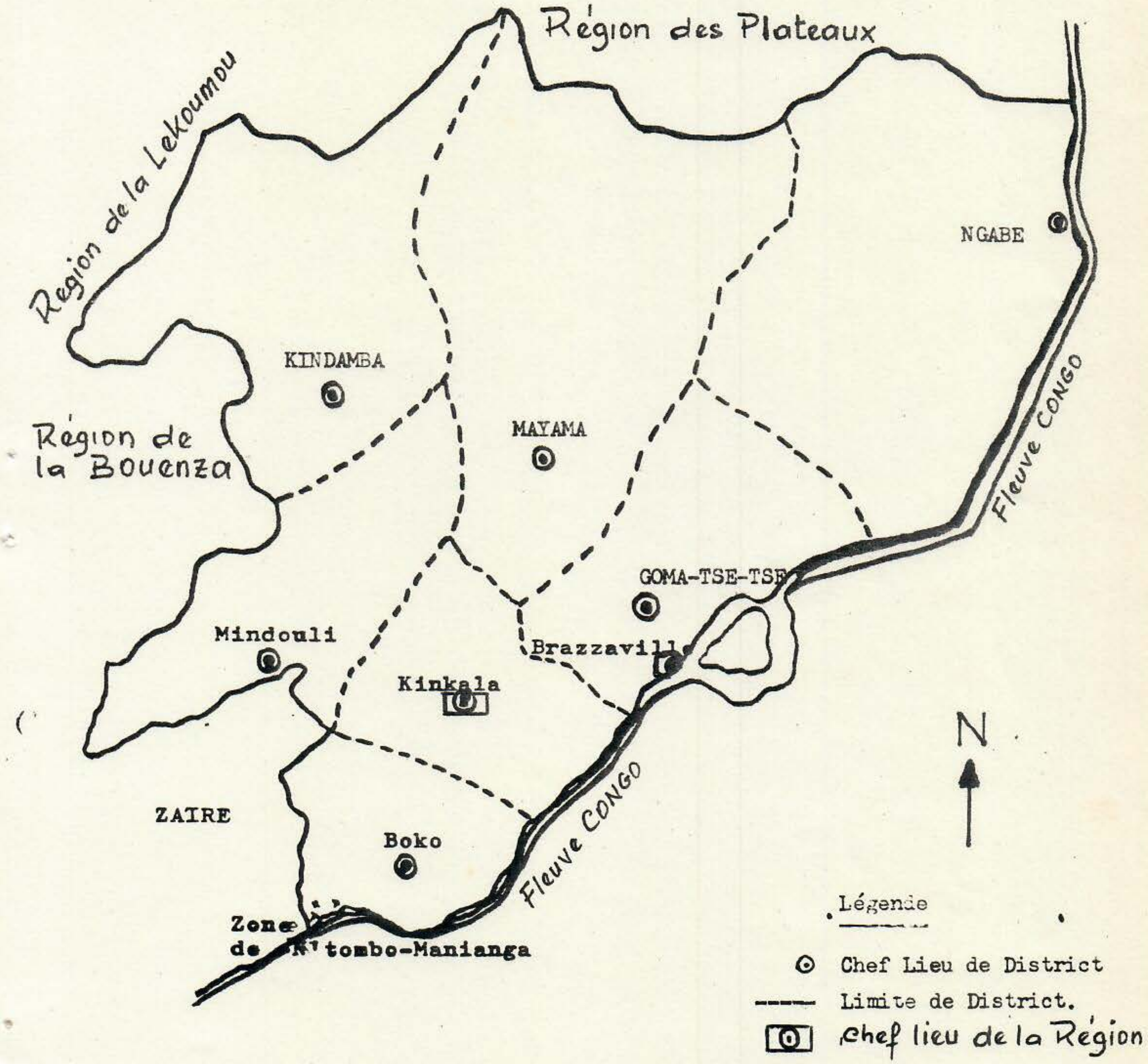

1-2-2 Le District de Boko : Chef lieu Boko qui semble le plus intéressé dans cette étude car il compte beaucoup plus de foyers d'onchocercose est situé dans la partie Sud de la région et est limité au Nord par la Foulakari et le District de Kinkala, au Sud-Ouest par le Zaïre et à l'Est par le fleuve Congo. Celui-ci (le fleuve Congo) offre aux populations riverraines du district de Boko l'une des principales acitivités:"la Pêche" .

Le District de Boko compte 26.696 habitants soit 1/7 environ de la population totale de toute la région du Pool. Leurs activités principales sont : la pêche et l'agriculture avec comme culture principale le manioc.

Les hommes comme les femmes participent aux travaux des champs : ceux-ci étant situés le plus souvent aux abords immédiats des cours d'eau comme le fleuve Congo ou ses affluents ; mais les femmes fréquentent beaucoup plus les champs que les hommes. Par contre la pêche est exclusivement l'activité des hommes et plus rarement pour les femmes.

Les enfants ne vont aux champs qu'en dehors des jours de classe. Ce qui peut limiter probablement leur degré d'infestation. Par contre, ils peuvent aller se baigner à n'importe quel moment de la journée, après les classes.

- On trouve en effet 1 (une) école dans chaque village visité.

../..

Comme partout ailleurs, la rivière occupe une place importante chez les habitants, car c'est là que tout se passe : baignade, lessives, vaisselles, rouissage du manioc, pêche etc.

Ainsi presque toute la partie Sud-Est du district de boko est baignée par le fleuve Congo et ses affluents comme la Foulakari, la Louvoubi et la Nkissi. Aussi les rapports entre la population et les cours d'eau (par l'emplacement des villages et des champs de cultures ou par les habitudes sociales), sont d'une importance capitale pour comprendre la transmission de l'onchocercose au sein de la collectivité.

CHAPITRE II

GENERALITES

CHAPITRE II

2-1 GENERALITES SUR LES FILARIOSES HUMAINES

2-1-1 Rappel Général

Les Filarioses humaines sont des helminthiases largement répandues en régions tropicales. On y rencontre plusieurs espèces qui peuvent être différenciées suivant leur localisation dans l'organisme, leur répartition géographique, leur mode de contamination et leurs manifestations cliniques.

Elles sont transmises à l'homme par des arthropodes et sont dues au parasitisme de l'homme par des nématodes à corps filiformes qui sont vivipares, c'est-à-dire pondent des larves et non des oeufs comme les autres helminthes. Ces larves sont appelées "microfilaires"

2-1-2 CLASSIFICATION

Il existe au total 8 (huit) filarioses humaines réparties en trois groupes suivant la localisation des filaires adultes : (voir tableau I) et en deux selon celle des microfilaires (voir tableau 2).

../..

Tableau I **Classification des Filarioses humaines selon la localisation des Filaires adultes**

Localisations	Filarioses (maladies)
Lymphatique	*Wuchérérioses-Brugioses*
Cutanéo-dermique	*Loase-Onchocercose-Dracunculose*
Sereuses	*Filarioses à :* *Mansonella Perstans* *Mansonnella Ozzardi* *Mansonnella Streptocerca*

Source : *Médecine tropicale (GENTILINI)*

Tableau N°2 **Classification des Filarioses selon la localisation des microfilaires :**

Localisations	Filarioses (maladies)
Sang = Microfilarémie	*Wuchereriose-Brugiose-Loase* *Filarioses à Mansonnella Perstans et Mansonnella Ozzardi*
PEAU = Microfilarodermie	*- Onchocercose ou Volvulose* *- Filariose à Mansonnella streptocerca* *- Filariose de Médine ou Filariose à Dracunculus Médinensis*

Source : Médécine tropicale (GENTILINI)

../..

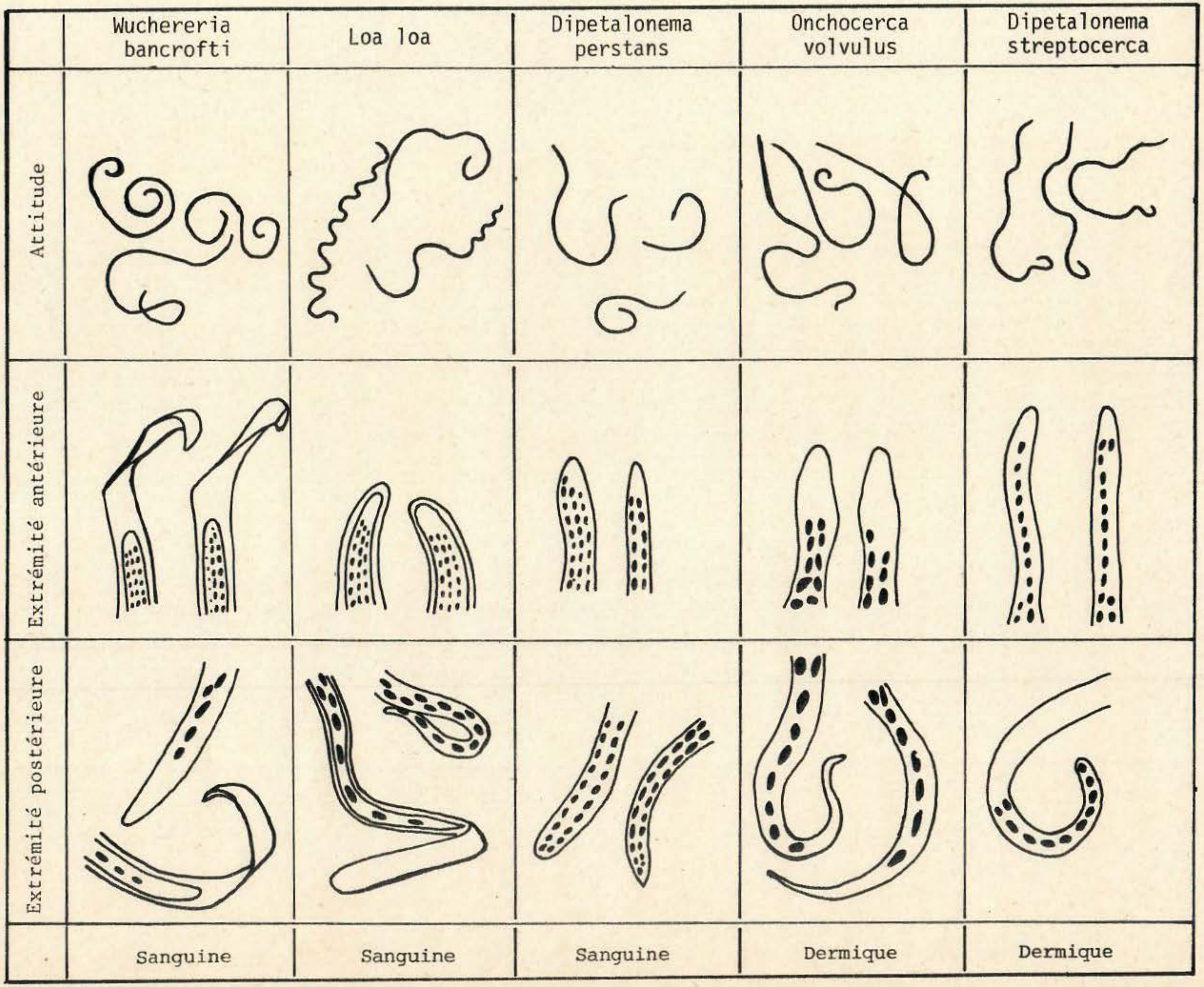

CARACTERES DES MICROFILAIRES DE L'HOMME EN AFRIQUE OCCIDENTALE ET CENTRALE

SCHEMA N°1

On remarque en particulier que les parasites embryonnaires ou microfilaires se localisent soit dans le sang, soit dans la peau.

- Classiquement, il existe les filaires pathogènes et les filaires non pathogènes.

Filaires pathogènes

- Onchocerca-volvulus
- Loa-Loa
- Dracunculus Médinensis (ou ver de Guiné)
- filaires de Bancroft et de Malaisie.

Fialaires non pathogènes ou peu pathogènes

- Mansonnella Perstans
- Mansonnella Ozzardi
- Mansonnella Streptocerca.

2-1-3 Répartition géographique

Les filaires sont rencontrées dans les régions tropicales et intertropicales du Globe. On compte ainsi : L'Wuchérériose (àwuchérérìa Bancroft) se rencontre dans toutes les régions intertropicales du globe.

La Brugiose : a une répartition géographique plus limitée: se rencontre surtout en Inde, Srilanka, Sud-Est Asiatique Indénosie et Philippines.

La Loase ; elle est exclusivement Africaine ; l'Afrique Centrale en est une Zone d'hyperendémie. (24)

La Filariose à Mansonnella Perstans : Elle est répandue en Afrique, dans le Nord de l'Amérique du Sud. (24)

../..

La Filariose à Mansonnella Ozzardi : Se rencontre en Amérique latine où elle sévit à l'Etat endémique.

La Filariose à Mansonnella Streptocerca :Elle est strictement africaine.

La Dracunculose : Elle sévit en Afrique et en Asie.

L'Onchocercose ou Volvulose :qui est notre propos, sévit en Afrique, en Amérique et au Yemen. (16)

2-2 L'ONCHOCERCOSE

2-2-1 DEFINITION

L'Onchocercose humaine ou cécité des rivières, encore appelée volvulose est une filariose cutanéo-dermique dûe à Onchocerca-volvulus. Elle est caractérisée par des manifestations cutanées-dermiques et des troubles occulaires pouvant aboutir à la cécité. Elle s'étend entre le 20e degré de latitude Nord et le 20e degré de latitude Sud et touche environ plus de 30 Millions de personnes dans le monde. L'Afrique à elle seule compte environ 99 % des cas. (2)

L'Onchocercose est une maladie grave, invalidante par ses complications dominées par les risques de cécité. Cette affection pose des problèmes socio-économiques de première importance dans les Etats concernés.

2-2-2 RAPPEL SUR L'EPIDEMIOLOGIE DE L'ONCHOCERCOSE

L'Onchocercose est une filariose transmise à l'homme dans nos régions par des piqures d'insectes

(femelles) vecteurs appelés simulies, appartenant au complexe simulium Damnosum qui est la principale espèce infestante.

Ces insectes vecteurs sont des diptères nématocères dont les larves et les nymphes vivent dans les eaux courantes.

- Les simulies pondent leurs oeufs sur les plantes aquatiques ou sur les rochers dans les eaux courantes et aérées, telles les cascades, les chutes, les rapides permanents.
Leurs gites sont habituellement situés entre la surface et 15 à 20 cm de profondeur. La femelle seule est hématophage.

L'Agent pathogène

L'agent pathogène est un ver rond (nématode) appelé : onchocerca-Volvulus, appartenant à la famille desfilaridés, à corps filiformes. (19)

Le Rapport entre le vecteur et le parasites :
S'effectue lors de l'infestation du vecteur à la suite d'un repas sanguin en prélevant le parasite chez l'homme malade.
Simulium damnosum supporte bien son parasitisme. (voir schema N° 2)

Quant au rapport entre le vecteur et l'homme ; il s'effectue dans la journée. Car les femelles des simulies ont une action diurne ; du lever au coucher du soleil.

../..

SCHEMA N° 2

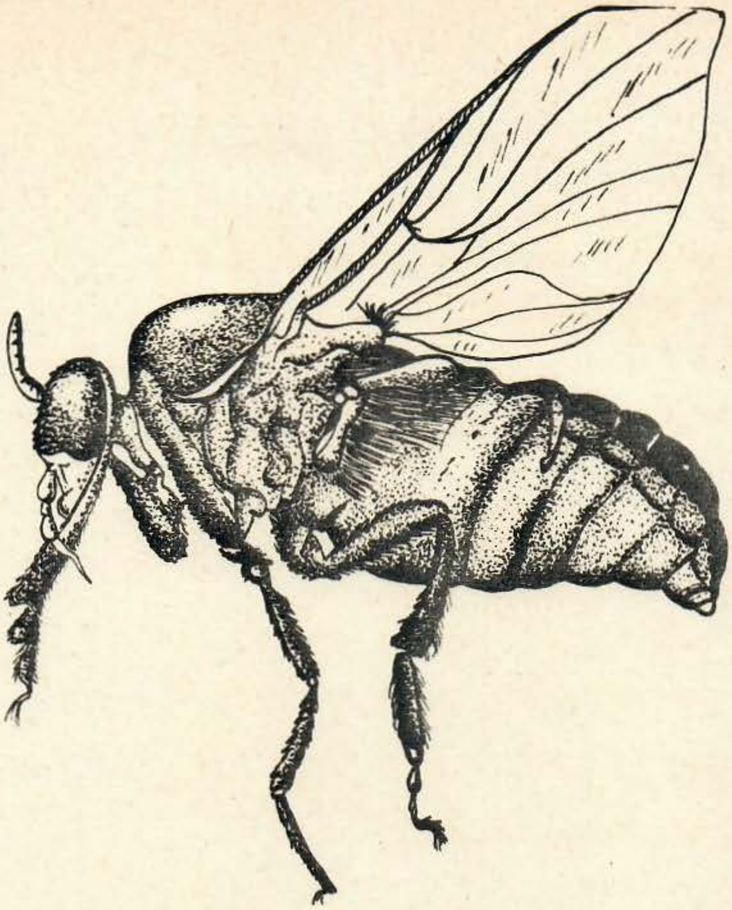

simulie ♀

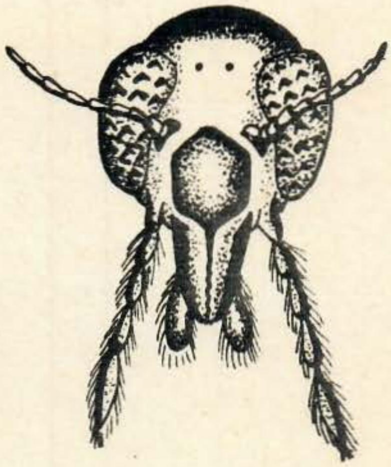

tête ♀

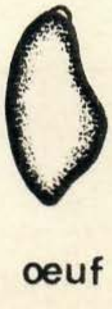

oeuf

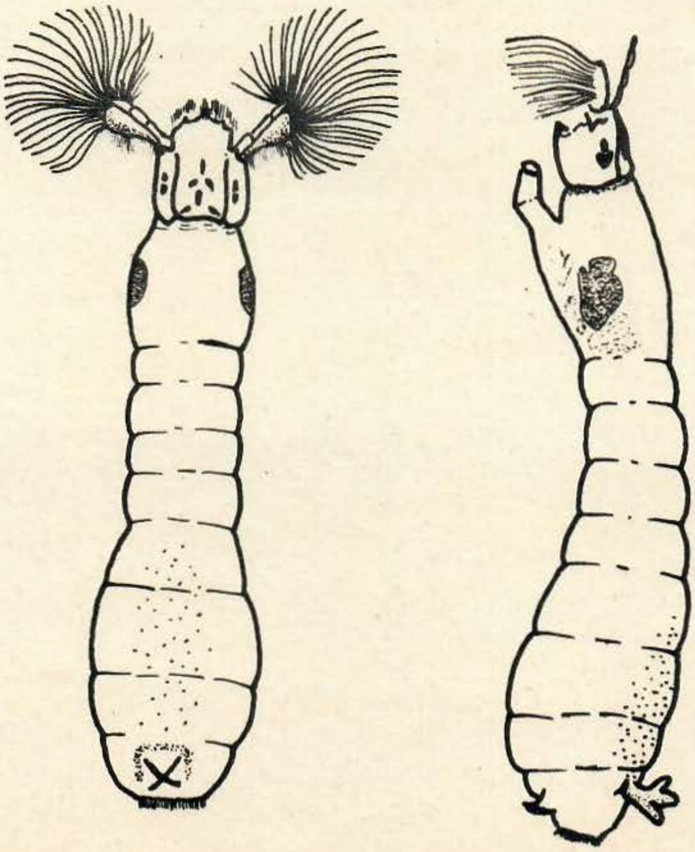

larve

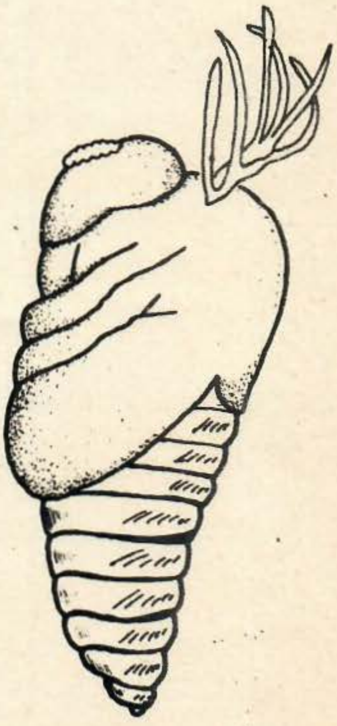

nymphe

Elles manifestent leur anthropophilie en piquant l'homme aux champs, le long des cours d'eau et rarement dans les villages, avec un ralentissement en mi-journée et en début d'après-midi lorsque la température excède 30° (19)

Transmission de la maladie

Les femelles des simulies s'infectent . en prélevant des microfilaires dans le derme d'un malade et contaminent un sujet sain en laissant s'échapper de sa trompe des larves infestantes qui traversent activement l'épiderme aü niveau de point de piqure. (16)

L'Ecologie des simulies conditionne la distribution de l'onchocercose. Ainsi il existe la différence entre l'onchocercose de forêt et l'onchocercose de savane.. Cette dernière étant beaucoup plus sévère par ses risques de cécité beaucoup plus dominant en savane qu'en forêt.

Le Réservoir de virus

Onchocerca-Volvulus est un parasite strict de l'homme. Le Réservoir est donc l'homme porteur de filaires adultes ou macrofilaires et des microfilaires. C'est même un excellent réservoir puisqu'il multiplie le parasite et le conserve longtemps dans son organisme permettant ainsi en zone d'endémie une réinfectation constante des populations simulidiennes. (19)

Facteurs favorisants :

Les professions obligeant l'homme de travailler proche des gites des vecteurs : Exemple : Cultivateurs

../..

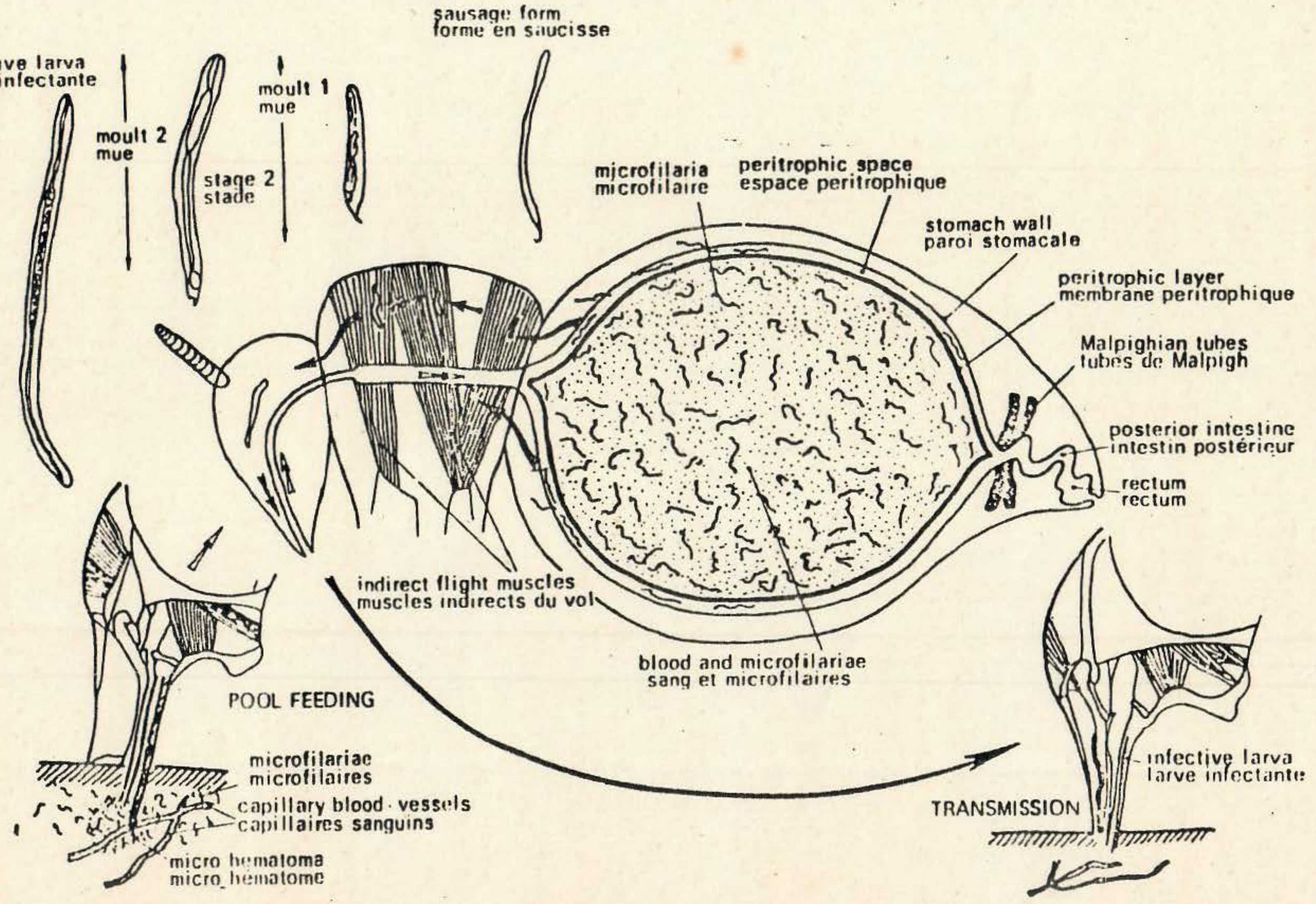

BLOOD - FEEDING MECHANISM OF SIMULIUM FEMALES ("POOL - FEEDING") AND DEVELOPMENT CYCLE OF ONCHOCERCA VOLVULUS IN SIMULIUM DAMNOSUM

MODE D'ABSORPTION DU SANG PAR LES FEMELLES DE SIMULIES (POOL-FEEDING) ET CYCLE DE DEVELOPPEMENT D'ONCHOCERCA VOLVULUS CHEZ SIMULIUM DAMNOSUM

SCHEMA N° 3

pêcheurs, chasseurs, piroguiers etc. et même certaines coutumes comme les bains constituent l'un des facteurs favorisant liés aux activités humaines.

RAPPEL SUR LA CLINIQUE

- L'Onchocercose, souvent cliniquement muette, s'exprime surtout par trois syndromes principaux.
- le syndrome cutané avec les onchodermites
- le syndrome kystique avec les onchocercomes
- le syndrome oculaire avec les kératites, Iritis et choriorétinites. (16)

Le Syndrome cutané

Il se manifeste d'abord par un prurit favorisant la formation des lésions de grattage aboutissant à ce qu'on appelle "Gale filarienne" qui siège surtout au niveau des lombes des fesses et des cuisses. D'autres manifestations cutanées qui en résultent sont :

La pachydermie

L'Atrophie cutanée.

Et certaines dépigmentations cutanées dont la Leucomelanodermie (ou pseudo-vitiligo) localisée surtout aux membres inférieurs. (19)

LE SYNDROME KYSTIQUE

Il est caractérisé par l'existence des nodules, kystes dermiques ou "onchocercomes" qui sont indolores, durs, fibreux, adhérents aux plans profonds et roulant sous les doigts. Ils sont particulièrement fréquents dans les régions trochantériennes, iliaques, sacrées, ou sur le gril costal, parfois dans le cuir chevelu.

Ces kystes indiquent l'ancienneté de la maladie (16).

LE SYNDROME OCULAIRE

Les signes oculaires sont dus à la présence des microfilaires dans l'oeil. Ils se traduisent par toute une série de troubles et de lésions de gravité croissante qui sont :

- Une photophobie avec larmoiement et céphalées
- une baisse de la vision crépusculaire (ou hemeralopie)
- une keratite ponctuée
- un rétrécissement du champ visuel
- une atrophie du nerf optique (ou atrophie optique)

Ces lésions oculaires évoluent lentement et inéluctablement, aboutissant à la cécité même si le parasitisme cesse. Elles dépendent de la durée et de l'intensité de l'infection (9).

2-2-4 RAPPELS SUR LA THERAPEUTIQUE

Dans l'onchocercose, deux types de médicaments sont utilisés :

Les macrofilaricides :actifs sur les filaires adultes procréatrices des microfilaires.

Les microfilaricides : actifs sur les microfilaires responsables des troubles cutanés et de lésions oculaires.

On administre à cet effet contre les microfilaires la DIETHYCARBAMAZINE ou NOTEZINE à dose effilée.
Et contre les filaires adultes : la SURAMINE SODIQUE ou MORANYL 20 mg/kg de poids soit 1 g pour un adulte par semaine pendant 5 à 7 semaines avec surveillance de l'albumine.

On peut également pratiquer la nodulectomie ou ablation de nodules.

Actuellement on parle d'un nouveau produit dans le marché pharmaceutique : l'IVERMECTINE.

Diagnostic biologique

Le diagnostic biologique se fait par la mise en évidence des microfilaires d'onchocerca-volvulus par la technique actuellement plus perfectionnée des BCE (biopsies cutanées exsangues).

Prophylaxie

On peut rompre la chaine épidémiologique en traitant les onchocerquiens ou en éliminant les simulies. Mais le traitement des Onchocerquiens est difficile et aléatoire, car les antihelmintiques disponibles sont onéreux et devraient être administrés sous surveillance médicale en raison de leur tolérance médiocre par l'organisme.

L'élimination des simulies donne des meilleurs résultats. C'est la lutte antilarvaire par épandage d'insecticides non rémanents comme le téméphos ou Abate, le Bacillus thuringiensis au niveau des gites aquatiques.

C'est ce qui se fait dans la région du Bassin des volta par un vaste programme de lutte contre l'onchocercose et qui a déjà donné de bons résultats.

2-3 L'ONCHOCERCOSE AU CONGO

HISTORIQUE

Au Congo, les premiers cas d'onchocercose ont été rapportés par LEBOEUF en 1919 dans la région de Brazzaville sur le Djoué. Puis deux ans plus tard OUZILLEAU et al (1921) et LAIGRET (1922) rapportent d'autres cas, montrant ainsi l'existence de cette maladie en terre congolaise.

Mais depuis, peu d'enquêtes précises ont été ...

../..

effectuées. Le seul travail important étant celui de OVAZZA qui remonte à 1953.

UNe étude bibliographique sur l'onchocercose effectuée par A. YEBAKIMA en 1978 a relevé de profondes lacunes dans les connaissances sur la taxonomie, la répartition et l'écologie des vecteurs, ainsi que sur leur répartition, la prévalence et l'incidence clinique de la maladie.

- Et depuis, est apparue la nécessité d'avoir des données épidémiologiques sur l'endemie onchocerquienne en République Populaire du Congo, de déterminer l'impact socio-économique de cette affection responsable de cécité ou des lésions oculaires graves sur les populations des zones concernées, et enfin la nécessité de mettre sur pied un programme de lutte adaptée en fonction des résultats obtenus.

- Ainsi, les premiers résultats obtenus ont conduit les autorités congolaises à créer un projet spécifique en 1981 à la tête duquel fut nommé un entomologiste médical de haut niveau Monsieur A. YEBAKIMA.

- Grâce à la création du dit projet de lutte contre l'onchocercose et au remarquable travail accompli sous la direction du Docteur A. YEBAKIMA qui a su animer ses collaborateurs et tout le projet de son dynamisme, un remarquable bilan entomologique, clinique et parasitologique a été effectué en République Populaire du Congo.

D'après les résultats de ces travaux : il ressort que l'endémie Onchocerquienne sévit dans les

../..

localités situées aux abords des rivières torrentueuses, possédant une végétation aquatique propice au développement des larves de simulies.

- Les enquêtes effectuées ont comporté plusieurs volets :

- Volet entomologique

- Destiné à localiser la présence du vecteur et des espèces en cause. C'est ainsi que l'on a identifié deux principaux groupes vecteurs de la maladie.

Simulium damnosum : dont les adultes parasités et des larves ont été découvertes sur les berges : du Djoué et du fleuve Congo (Mafouta-Massissia - Cité OMS - quartier T A N A F à Brazzaville.)
De la Louholo (à Kindamba dans la région du POOL) ;
Du Djoué encore (à Mayama Poste, à Kinsassa et à Mpayaka dans la région du Pool).
Du Niari (à Loudima dans la région de la Bouenza)
Du Koulou et de Rivière Moudji (à Mvouti et à Moudji dans la région du Kouilou).

Simulium Albivirgulatum: dans la zone du Nord du pays sur l'axe Brazzaville-Mbomo ; (rivières : Léfini, Nkeni Alima, Kouyou, Likouala).

Sur près de deux milles femelles de cet espèce disséquées, aucune n'a été trouvée infectée. (voir carte N°5 sur la Répartition des simulies au Congo)

Ainsi donc il semble que cet espèce n'est pas vectrice de la maladie au Congo contrairement à ce qui a été signalé dans la cuvette Centrale du Zaïre (qui est un pays limitrophe du Congo) où cette espèce est l'unique vecteur de la maladie (d'après FAIN et al 1969)

../..

CARTE N° 5

REPARTITION DES SIMULIES ET DE L'ONCHOCERCOSE EN REPUBLIQUE POPULAIRE DU CONGO

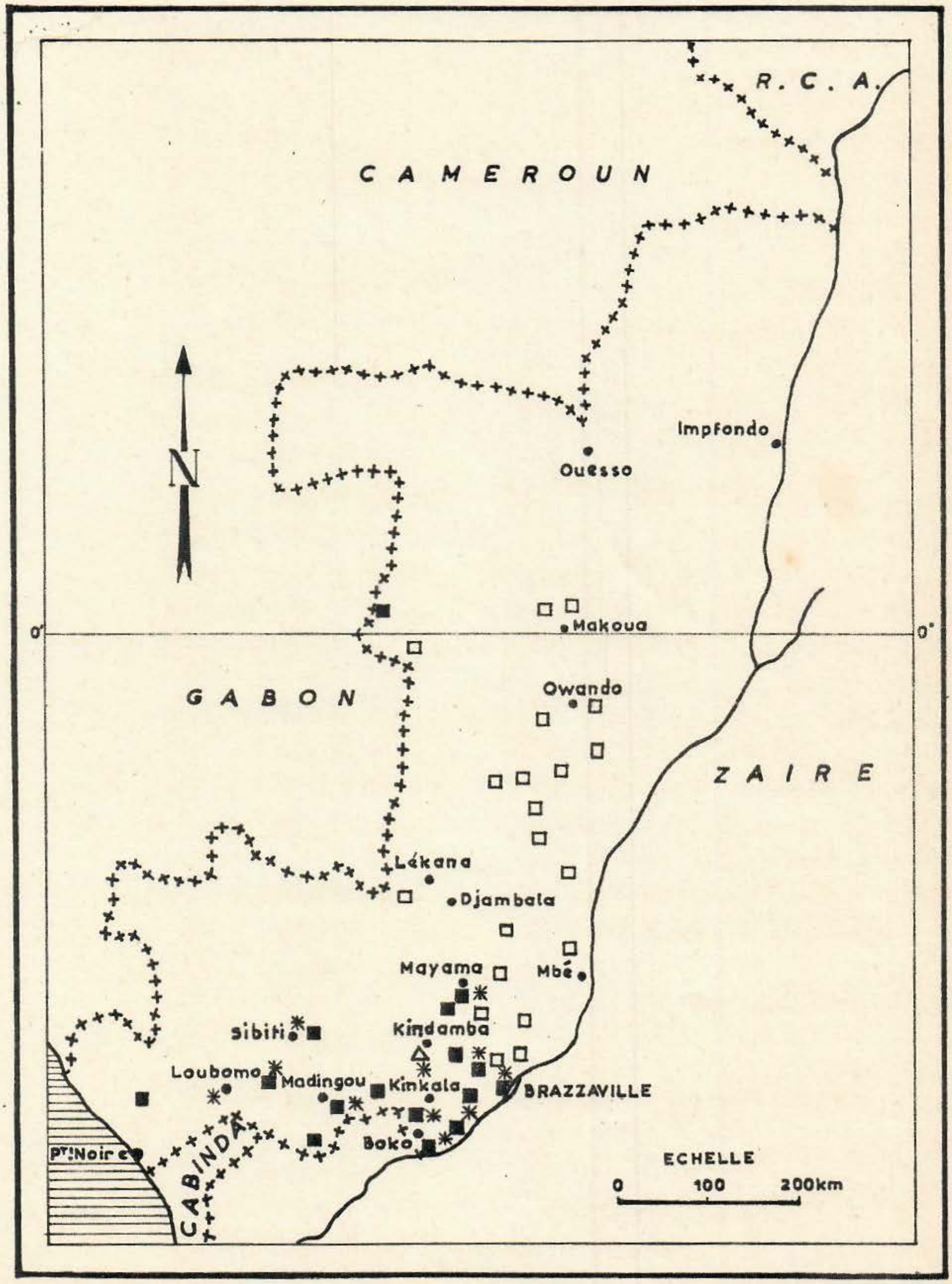

■ Simulium damnosum
□ Simulium albivirgulatum
Δ Simulium neavei
✱ Foyer d'onchocercose

- Volet clinique et ophtalmologique :

- *Destiné à mettre en évidence chez les populations riverraines des lésions cutanées et oculaires, d'étiologie onchocerquienne. (Résultats = voir enquête retrospective)*

- Volet parasitologique :

- *Destiné à évaluer le pourcentage de porteurs de microfilaires et donc l'intensité de la transmission de la maladie.*

RESULTATS ACQUIS

- *Les résultats acqùis ont permis d'avoir des éléments sur :*

1) La Répartition spaciale du vecteur : une communication, scientifique a été faite à ce propos par A. YEBAKIMA au Congrès international de MANILLE en 1980.

2) La Transmission de la maladie : qui est intense aux bords des cours d'eau.

3) La Prévalence : très élevée dans certaines localités : exemple : 75 % des sujets atteints à Kinsassa (District de Kinkala).

- Mais malheureusement, les activités du projet ont été brutalement interrompues en Avril 1983 par suite du départ définitif pour des raisons personnelles du chef de Projet.

- Ce n'est que deux ans plus tard (c'est-à-dire en 1985 que le projet a repris ses activités après la nomination du Professeur B. CARME à la tête du dit Projet. (14)

CHAPITRE III

METHODOLOGIE

CHAPITRE III

METHODOLOGIE

De la période du 25 septembre 1985 au 20 Février 1986 nous avons réalisé deux enquêtes sur l'Onchocercose dans la région du Pool.

- une enquête rétrospective du 2/10/1985 au 5/11/1985
- et une enquête prospective du 18/11/1985 au 26/11/1985.

3-1 ENQUETE RETROSPECTIVE

Dans les archives et services de documentation de la Direction de la Médecine Préventive et du Programme de lutte contre l'Onchocercose de la République Populaire du Congo, nous avons recherché tous les éléments nécessaires relatifs aux études antérieures effectuées dans la région du Pool sur, l'Onchocercose. De cette recherche nous avons élaboré une synthèse de tous les travaux que nous avons intitulé : "Etudes anciennes".

3-1-1 Etudes anciennes sur l'Onchocercose dans la région du Pool

Cette étude comporte les travaux déjà effectués dans la région du Pool.

En effet, les foyers d'onchocercose déjà connus dans cette région sont au nombre de 7 (sept) repartis comme suit :

Bassin du Djoué = 5 foyers

- Foyer du Kinsassa (District de Kinkala)
- Foyer de Mpayaka
- Foyer de Mayama (District de Kayama)
- Foyer de Kibossi (District de Goma-tsé-tsé)
- Foyer de Mafouta Massissia (Brazzaville)

Bassin du Congo = 1 Foyer

- Foyer de Ntombo-Manianga (District de Boko)

Bassin de la Louholo = 1 foyer

- 1 foyer de Bangou-Louholo

Ainsi donc les premières enquêtes dans cette zone ont été menées à partir de 1978. Une synthèse a fait l'objet d'une publication en 1982 par A. YEBAKIMA et Côll.

Elles se sont déroulées (les enquêtes) dans cinq zones qui sont :

- Brazzaville (cours inférieur du Djoué)
- Kibossi-Kinsassa et Mpayaka (cours moyen du Djoué)
- Mayama (cours supérieur du Djoué)
- Ntombo-Manianga (dans la partie Sud-Ouest de Boko)
- Et à Bangou-Louholo (dans le District de Kindamba)

3-1-2 Résultats obtenus

Résultats entomologiques

Ces enquêtes ont montré l'existence de deux types de gîtes préimaginaux le long de la rivière Djoué.

1°) Gîtes de hautes Eaux crées par l'accélération de la vitesse du courant d'eau de surface et l'existence de certains supports.

../..

2°) Gîtes de basses Eaux : Au niveau des dalles rocheuses, constituants le lit de la rivière et le long des berges de certains cours d'eau comme le Djoué et le fleuve Congo.

Certains gîtes préimaginaux à Simulium damnosum sont constitués par la série des cascades rencontrées tant dans le fleuve Congo que dans le Djoué, ainsi que par la végétation aquatique qui s'y trouve.(42)

Il y a également les rapides du fleuve Congo en aval de Brazzaville, donc dans la région du Pool même, qui favorise le développement des simulies et constituent d'importants gîtes/tout comme dans certains ouvrages artificiels tels que : le barrage hydro-électriques du Djoué (A. YEBAKIMA et Coll 1978).(42)

Identité du Vecteur

Le vecteur majeur de l'Onchocercose humaine connu et dépisté dans toute la région est le Simulium-damnosum : espèce répandue dans presque toute la partie Sud du Pays (A. YEBAKIMA 1978)(42)

Aussi les études effectuées dans la région ont montré une présence permanente du vecteur avec des variations de densité dans le temps et dans l'espace.
Exemples : Barrage du Djoué à Brazzaville 18 à 170 femelles par homme et par jour.
à Mayama 200 à 450 femelles par homme et par jour
à Kinsassa 180 à 500 femelles par homme et par jour
Tout ceci rentre bien dans l'étude de la dynamique de la transmission onchocerquienne (A. YEBAKIMA et Coll 1978) (42)

- Et les résultats des dissections ont montré que toutes ces femelles sont parasitées par Onchocerca-volvulus. L'âge physiologique est élevé (35 à 80 % des femelles pares).
On a observé en outre 1 à 5 % de femelles pares infectieuses avec un potentiel individuel de transmission de 2,5 à 7 larves par femelles dans le cours inférieur du Djoué (Foyer de Mafouta-Massissia) contre 64,5 % de femelles infectieuses dans le cours moyen du Djoué (Foyers de Kinsassa et Mpayaka.

- Aussi, nous pouvons affirmer comme le montre le tableau III ci-dessous que le stade de transmission de la maladie (ou femelles pares) se rencontre dans la totalité des foyers connus (A.YEBAKIMA 1978).(42)

- Il est à noter que l'âge physiologique moyen (ou proportion de femelles pares) permet d'apprécier la longévité des femelles piqueuses au sein d'une population et la possibilité de transmission de la maladie.

Tableau N° 3 : Valeur de l'âge physiologique moyen et du taux de parasitisme des femelles piqueuses de Simulium-damnosum en fonction de la saison et par village

Années	Saisons	Localités	Rivières	Age physiologique moyen	Femelles parasitées
1977	Novembre	Mandombé	Mbouessa	40%(32/80)	20 %
1977	Novembre	Bela	Louvoubi	61,8%(84/136)	19 %
1978	Mars	Mafouta-Massissia	Djoué et fleuve Congo	58%(107/182)	11 %
1977	Décembre	Bangou Louholo	Louholo	71,8%(1804/2511)	20 %
1978	Février	Bangou Louholo	Louholo	39,7%(1039/2618)	8 %
1978	Avril	Bangou Louholo	Louholo	65,5%(882/1347)	14,3 %
1979	Octobre	Tanaf (Brazzaville	Djoué	65%(345/530)	12,3 %
1979	Novembre	Mayama	Djoué	59%(1330/2230)	13 %

Source = Simulies et onchocercose en R P C.
Publication de A. YEBAKIMA et J.L.FREZIL 1978

Différentes saisons : Octobre - Novembre - Décembre = petite saison des pluies
Février - Mars = petite saison sèche
Avril - Mai = grande saison des pluies
Juin - Juillet - Août - Septembre = grande saison sèche

../..

3-1-3 **Résultat par zone**

3-1-3-1 Zone de Kibouendé : *Situé dans le cours Moyen du Djoué, il comprend les foyers de Mpayaka et Kinsassa. Simulium damnosum avait été capturé; les études entomologiques effectuées dans cette zone ont montré une densité de femelles piqueuses élevée et variant selon les saisons et les lieux.*

Exemple : *à Mpayaka : 134 femelles par homme et par jour en saison pluvieuse, et 180 femelles par homme et par jour en saison sèche contre 534 femelles par homme et par jour en saison sèche à Kinsassa avec un taux de femelles infectieuses égal à 64,5 % et 5,9 % ;*

- Les variations de taux de femelles parasitées par les larves d'Onchocerca-Volvulus et de femelles infectieuses sont peu importantes puisqu'elles passent respectivement de 8,8 % et 3,8 % en saison des pluies à 8,6 % et 4 % en saison sèche presque dans toute la zone étudiée. (A. YEBAKIMA et J.L. FREZIL 1978) (42)

3-1-3-2 Zone de Mafouta-Massissia : *Situé dans le cours inférieur du Djoué, comprend les foyers de la TANAF (Brazzaville) et de Mafouta-Massissia, (banlieue de Brazzaville).*

Dans cette zone, nous avons dit que les gites préimaginaux de Simulium-damnosum sont constitués par les rapides du Congo en aval de Brazzaville, les rapides et le barrage hydro-électrique du Djoué.

../..

10,9 % (20/182) des femelles disséquées sont parasitées et 7,5 % des femelles sont infectieuses avec une moyenne de 2,8 larves infectantes.

Les études cyto-taxonomiques faites à Brazzaville et ses environs ont montré que le cytotype en cause dans cette zone est Simulium-Squamosum

3-1-3-3 Zone de Bangou-Louholo

Dans la zone de Bangou-Louholo, l'étude menée en 1978 a montré que les gîtes sont localisés sur la Louholo et son affluent la Moulouri. Seule Simulium damnosum avait été capturé sur l'appât humain avec 20 % de femelles parasitées par Onchocerca-volvulus. 7,9 % de femelles infectieuses avec une moyenne de 6,3 larves infectantes par femelles infectieuses. (44)

Résultats clinico-Parasitologiques

Ici également, nous présenterons les résultats par zone.

3-1-3-4 Zone de Brazzaville

Située dans le cours inférieur de Djoué, cette zone est recouverte de savane arbustive et se localise à l'entrée Sud de l'agglomération de Brazzaville, dans l'angle formé par la rivière Djoué et le fleuve Congo. Elle est constituée par les foyers de Mafouta-Massissia située dans la banlieue Sud de la ville de Brazzaville, sur les bords du fleuve Congo et du Djoué ; et le foyer de la TANAF qui est l'un des quartiers de l'agglomération de Brazzaville dans l'arrondissement I Makélékélé.

../..

Tableau N° 4 : Prévalence de l'onchocercose dans la zone de Brazzaville.

Localités	Sujets examinés	Porteurs des microfilaires	%	Porteurs des kystes	%	Cas de cécité
Mafouta-Massississia	307	131	42,7	46	14,9	3
Tanal	62	22	35,5	7	11,3	0
Total	369	153	41,46	53	14,36	3

Source = Rapport d'enquête sur l'Onchocercose dans le foyer du Djoué (par A. YEBAKIMA-V. OBOUAKA-A. KONONGO-F. NANGA-J. GOMA et M. COSMANS 1978)

Dans cette zone, sur 369 sujets examinés : 153 personnes sont porteurs de microfilaires d'onchocerca-volvulus, soit un indice microfilarien (IM) de 41,46 %.
Et 53 sont porteurs de kystes soit un indice kystique (IK) de 14,36 %.

L'indice clinico-parasitologique (ICP) à Mafouta-Massississia est de 44,9 % (138/307) avec chez les enfants de 0-14 ans un indice clinico-parasitologique de 8,9 % et chez les adultes de plus de 14 ans, l'indice clinico-parasitologique (I C P) est de 65,6 % (A. YEBAKIMA et Coll 1978)(43)

- 3 cas de cécité ont été observés chez les sujets porteurs de microfilaires ; soit un taux de cécité de 0,97 % (A. YEBAKIMA et Coll 1978). (43)

3-1-3-5 Zone de Kibouendé

Situé dans le cours moyen du Djoué, cette zone constitue :

1°) Le foyer de Kinsassa, situé à 95 km (par piste de Brazzaville, et à 1 km de la rivière Djoué, qui lui-même (le Djoué) est bordé d'une galerie forestière, le reste de la zone étant une savane arbustive.

L'agriculture (manioc, patate, arachides) est l'activité essentielle des hommes et des femmes de toute la zone, et leurs champs de culture étant situés aux abords immédiats du Djoué.

2°) - Celui (le foyer) de Mpayaka situé à 88 km de Brazzaville (par piste)

3°) - Et celui de Ngamalie

Dans le cours moyen du Djoué (zone de kibouendé), l'indice microfilarien (IM) varie entre 37,2 et 67,8 %.

Tandis que l'indice kystique (IK) est de 11,7 % à Ngamalié ; 21,6 % à Mpayaka et 27,4 % à Kinsassa (23) (voir tableau V ci-dessous).

../..

Tableau N° 5 : Prévalence de l'Onchocercose dans la zone de Kibouendé en fonction des localités.

Localités	Sujets examinés	Porteurs de microfilaires	%	Porteurs de kystes	%	I C P %
Mpayaka	384	186	48,43	83	21,6	55 %
Kinsassa	84	57	67,8	23	27,4	75 %
Ngamalié	349	130	37,2	41	11,7	40 %
Total	817	373	48,10	147	17,99	52 %

Source = Rapport d'enquête sur l'onchocercose dans le cours-moyen du Djoué (par A. YEBAKIMA et Coll. 1979).

En Juin 1981 : B. CARME et Coll rapportant les résultats d'une enquête Ophtalmologique effectuée dans la zone de Kibouendé.

- Cette enquête ayant interessé le village de Mpayaka situé à 88 km de Brazzaville. Outre l'évaluation des paramètres cliniques et biologiques habituels, l'étude comportait donc en plus un examen ophtalmologique réalisé par un médecin spécialiste pour les sujets de plus de 20 ans.

- Au total 384 personnes ont été examinées et réparties comme suit :

Hommes = 185 (48,2 %)

Femmes = 199 (51,8 %)

Les résultats obtenus ont donné 186 sujets sur 384 sont porteurs de microfilaires d'onchocerques, soit un indice microfilarien (I M) de 48,43 % (186/384) dont 85,7 % chez les hommes

../..

adultes contre 62,4 % chez les femmes. Alors qu'il est équivalent chez les sujets de moins de 20 ans.

La densité microfilarienne moyenne (D M M) est de 31,3 avec des valeurs de 42,4 pour les hommes et 20,2 pour les femmes.

- 83 des 384 sujets sont porteurs de kystes soit un indice kystique (I K) global de 21,6 % avec 17,2 % pour les hommes et 25,6 % pour les femmes (voir tableau VI ci-dessous)

- Pour les adultes, l'indice kystique (IK) est de 39,4 % contre 48,8 % chez les moins de 20 ans, avec 7,7 % chez les garçons et 5,7 % chez les filles (Prof. B CARME et Coll 1981). (6)

Tableau N° 6 : Prévalence de l'Onchocercose à Mpayaka en fonction du sexe

Sujets examinés			Porteurs de microfilaires						porteurs de kystes					
Hommes	femmes	Total	hommes	%	femmes	%	Total	%	hommes	%	femmes	%	Total	%
185	199	384	159	85,9	124	62,3	283	73,69	32	17,2	51	25,6	83	21,6

Source = Rapport d'enquête sur l'onchocercose dans le village de Mpayaka (par Prof. B. CARME 1981)

Les examens Ophtalmologiques ont donné les résultats suivants :
à Mpayaka

Sur 200 onchocerquiens examinés, 34 sont porteurs des lésions oculaires dues vraisemblablement à l'onchocercose soit un pourcentage de 17 % avec la distribution suivante :

1°) Atteintes du segment antérieur = 19 cas

../..

avec : keratite ponctuée = 12
keratite sclérosante = 5
Iridocyclite = 2

2°) Atteintes du segment postérieur = 13 cas
avec : choriorétinite = 9
atrophie optique = 4
et 2 cas de cécité totale .

(voir tableau VII ci-dessous)

Tableau N° 7 : Distribution des atteintes oculaires dont l'origine est vraissemblablement onchocerquienne à Mpayaka.

Localisations	Lésions oculaires	Nombre de cas	%
Segment Antérieur	Keratite ponctuée	12	35,29 %
	Keratite sclérosante	5	14,70 %
	Iridocyclite	2	5,88 %
Segment Postérieur	Chorioretinite	9	26,47 %
	Atrophie optique	4	11,76 %
	Cécité totale	2	5,88 %
	TOTAL	34	100

Source : Rapport d'enquête sur les e aspects oculaires de l'onchocercose à Mpayaka (par A. YEBAKIMA - B. MENEZ et M. MDUYEKE (1980)

../..

A Kinsassa

Sur 48 onchocerquiens examinés : 17 sont porteurs de lésions typiques : soit un pourcentage de 35,4 % dont 1 cas de cécité totale. Ces lésions oculaires sont distribuées comme suit :

1°) Atteintes du segment antérieur = 9 cas
avec : keratite ponctuée = 5 cas
keratite sclérosante = 3 cas
Iridocyclite = 1 cas

Total = 9 cas : soit un pourcentage d'atteinte du segment antérieur de 52,9 % (9/17).

2°) Atteintes du segment postérieur = 6 cas
avec : atteintes rétiniennes = 5 cas
atrophie optique = 1 cas

Total = 6 cas soit un pourcentage d'atteinte du segment postérieur de 35,3 % (6/17) ;

3°) Atteintes simultanées des segments antérieurs et postérieurs = 2 cas soit un pourcentage de 11,8 % (2/17)

4°) Sujets avec baisse de l'acuité visuelle : (seules sont considérés les sujets présentant une acuité visuelle égale ou inférieure à 6/10e : nombre total = 10 soit un pourcentage de 20,8 % (10/48). Dont 8 cas chez les sujets avec lésions oculaires soit un pourcentage de 47 % (8/17). Et 2 cas chez les sujets sans lésions oculaires soit 6,45 % (2/31) (45)
(voir tableau ci-dessous

../..

Tableau N° 8 : Distribution des atteintes oculaires dont l'origine est vraissemblablement Onchocerquienne à Kinsassa

Localisations	Lésions oculaires	Nombre de cas	%
Segment antérieur	Kératite ponctuée	5	29,41
	Keratite sclérosante	3	17,64
	Iridocyclite	1	5,88
Segment postérieur	Atteintes rétiniennes	5	29,41
	Atrophie optique	1	5,88
Les deux segments simultanément		2	11,76
	Total	17	100

Source = Rapport d'enquête sur les aspects oculaires de l'onchocercose dans le village de Kinsassa : (par A. YEBAKIMA B. MENEZ. M. MOUYEKE 1980)

3-1-3-6 Zone de Mayama

Située dans le cours supérieur du Djoué, elle constitue le foyer de Mayama Poste.

La presque totalité du bassin du Djoué est une savane à Loudétia pauvre. Et vers le Sud apparaissent des portions de forêts mésophiles. Et une zone de savane à aristidia typique se développe dans la région de Mayama.

En effet, le foyer de Mayama Poste est située à quelques 80 kilomètres de Brazzaville (par piste) et juste au bord du Djoué. Ici également le Djoué est bordé par une étroite galerie forestière.

../..

- L'Enquête à Mayama s'est effectuée en 1981 et avait donné les résultats suivants :
Sur 900 personnes examinées à Mayama, 563 sont porteurs de microfilaires d'onchocerques, soit un indice microfilarien (IM) de 62,5 % et 153 personnes sont porteurs de kystes (voir tableau IX ci-dessous)

Tableau N° 9

Prévalence de l'onchocercose à Mayama (1981)

Sujets exmainés	Porteurs de microfilaires	%	Porteurs de kystes	%
900	563	62,5	153	17

Source = Rapport d'enquête sur l'onchocercose à Mayama (par A. YEBAKIMA et Coll 1981)

3-1-3-7 Zone de Bangou-Louholo

Dans la zone de Bangou Louholo : l'enquête s'est déroulée en 1978 dans les villages : Bangou-Louholo situé à 200 km au Nord-Ouest de Brazzaville, sur le bord de la rivière Louholo. Elle même (la louholo) localisée en lisière de la grande forêt de Bangou (District de Kindamba). Et, avec une enquête comparative à Ntari II village situé à 7 kilomètres de Bangou-Louholo et à environ 8 km du fleuve Niari.

Elle comportait (l'étude) en plus de l'évaluation des paramètres habituels (indice kystique (IK) et indice microfilarien (IM) une étude sérologique (application de la technique immunoenzymatique "Elisa"), par la méthode des prélèvements sur conféttis (A. YEBAKIMA) 1978) (44)

Résultats

Village Bangou-Louholo

Sur 266 personnes examinées : 109 sont porteurs de microfilaires d'onchocerca-volvulus soit un indice microfilarien (IM) de 40,97 % et 56 personnes sont porteurs de kystes soit un indice kystique (IK) de 21,05 %.

La prévalence totale de la maladie est de 44,4 % (indice clinico-parasitologique (ICP) soit 118/266.
La densité microfilarienne moyenne (DMM) est de 22,2 (A. YEBAKIMA et Coll 1978). (44)

- L'examen clinique relève des manifestations cutanées dans 46,6 % des cas.
4 cas de cécité totale sont observés les 4 sujets étant onchocerquiens

Résultats sérologiques à Bangou-Louholo

Le taux de positivité en "Elisa" est de 65 % dans une population où l'indice clinico-parasitologique (I C P) est de 45 %

Résultats à NTARI II

A Ntari II, aucune simulie n'a été capturée après 3 jours. 41 des 120 personnes visitées sont porteurs de microfilaires d'onchocerca-volvulus soit un indice microfilarien (IM) de 34,16 %. 15 personnes sur 120 sont porteurs de kystes, soit un indice kystique (IK) de 12,5 %
L'indice clinico parasitologique (I C P) est de 38,3 %. Les manifestations cutanées touchent 38 personnes soit un pourcentage de 31,66 %

- Et la baisse de l'acuité visuelle est retrouvée chez 11 personnes onchocerquiennes soit un pourcentage de 9,16 %.
Le taux de cécité déclarée ou observée est nul dans ce village. (44)

../..

Tableau 10 : Prévalence de l'onchocercose dans la zone de Bangou-Louholo

Localités	Sujets examinés	Porteurs de microfilaires	%	Porteurs de kystes	%
Bangou-Louholo	266	109	40,97	56	21,05
Ntari II	120	41	34,16	15	12,5
Total	386	150	38,86	71	18,39

Source L'Onchocercose en République Populaire du Congo (par A. YEBAKIMA et Coll.)

3-1-3-8 Zone de Ntombo-Manianga

A la lumière des résultats obtenus dans le bassin du Djoué et à Bangou-Louholo ; il a paru important de recueillir en Juin 1982 des éléments sur la transmission des filarioses à microfilaires dermiques dans la zone de Ntombo Manianga ; (District de Boko) situé le long du fleuve Congo, à 40 km de Boko; (chef lieu du District) au Sud-Ouest du district de Boko.

En effet, l'existence des rapides sur le fleuve Congo au niveau de Ntombo-Manianga laisse supposer l'existence des gîtes de simulies vectrices d'onchocerca-volvulus. Cette enquête a été complétée et approfondie en 1985

Résultats de 1982

En 1982, un total de 323 personnes dont 122 enfants ont été examinés.
172 sujets sur les 323 sont porteurs de microfilaires d'onchocerca-volvulus ; soit un indice microfilarien (IM) de 53,25 % (172/323). La palpation des nodules a revelé la présence des kystes chez 138 personnes, soit un indice kystique (IK) de 42,72 % (138/323)

../..

L'indice clinico-parasitologique (ICP) est dans ce village de 65 %.
Les manifestations cutanées atteignent 43,65 % (141/323)

1 cas de cécité évidente a été rencontré chez un sujet de 60 ans porteurs de 2 nodules.
La densité microfilarienne (DMM) est globalement de 20.

Tableau 11 Prévalence de l'onchocercose à Ntombo-Magnianga (1982)

Sujets examinés	Porteurs de microfilaires	IM %	Porteurs de Kystes	IK%	ICP %
323	172	53,25	138	42,72	65

Source = Rapport d'enquête sur l'onchocercose à Ntombo-Magnianga-District de Boko (par A.YEBAKIMA et Coll)

Enquête de 1985 dans la même zone de Ntombo-Magnianga

En 1985 un total de 190 personnes tous âges de plus de 20 ans a été examiné, dont 148 sont porteurs de microfilaires d'onchocerca-volvulus ; soit un indice microfilarien(IM) de 77,9 % 99 personnes sur 190 sont porteurs de kystes soit un indice kystique (IK) de 52,10 %.(23)

Tableau N° 12 Comparant les Prévalences de l'onchocercose des années 1982 et 1985 à Ntombo-Magnianga

Années	Localités	Sujets examinés	Porteurs de microfilaire	IM%	Porteurs de kystes	IK%
1982	Ntombo-Magnianga	323	172	52,25	138	42,72
1985	Ntombo-Magnianga	190	148	77,9	99	52,10

Dans le tableau ci-dessus, on remarque que malgré le nombre relativement faible des sujets examinés en 1985 par rapport à 1982, 190 sujets en 1985 contre 323 en 1982. En l'espace de 3 ans seulement, on constate donc qu'il y a une forte augmentation de porteurs de microfilaires 172 pour 323 examinés en 1982 contre 148 pour 190 examinés en 1985 ; avec évidemment comme conséquence, une augmentation de l'indice microfilarien (IM) 53,25 % en 1982 contre 77,9 % en 1985.

Il y a également une assez forte augmentation de l'indice kystique (IK) 42,72 % en 1982 pour 323 examinés contre 52,10 % en 1985 pour 190 examinés.
La comparaison statistique des prévalences de ces deux années par le test de l'Ecart-réduit nous donnent les résultats suivants:

$$\varepsilon = \frac{Pa - Pb}{\sqrt{\frac{Pq}{Na} + \frac{Pq}{Nb}}}$$

avec pour : Pa = Prévalencé en 1982 = 0,52
Pb = Prévalence en 1985 = 0,77
q = Complément à 1 de 0,52 = 0,48
q = Complément à 1 de 0,77 = 0,23
Na = Effectif de la Pop. en 1982 = 323
Nb = Effectif de la Pop. en 1985 = 190

on a donc $|\varepsilon| = \frac{0,52 - 0,77}{\sqrt{\frac{0,52 \times 0,48}{323} + \frac{0,77 \times 0,23}{190}}} = 2,77$

../..

ε = écart réduit

$|\varepsilon|$ = 2,77 > 1,96 (pratiquement 2) la différence est statistiquement significative au seuil de 5 %

Atteintes oculaires

71 sujets sur 190 sont porteurs de lésions oculaires typiques dont les principales reparties comme suit :

1°) Atteintes du segment antérieur = 34 cas
avec : keratite ponctuée = 30 cas
keratite sclérosante = 0
Iridocyclite = 4 cas

Total = 34 cas , soit un pourcentage de 47,88 % (34/71) pour les atteintes du segment antérieur.

2°) Atteintes du segment postérieur = 7 cas
avec : choriorétinite = 2 cas
atrophie optique = 5 cas

Total = 7 cas, soit un pourcentage de 9,85 % (7/71) pour les atteintes du segment postérieur

3°) Cécité totale = 2 cas (23)

Il est à noter que les examens ophtalmologiques ont été effectués par le docteur KAYA spécialiste à l'hôpital Général de Brazzaville

../..

Tableau N° 13 : Manifestations oculaires globalement observés en fonction du sexe en 1985 à Ntombo-Magnianga

Atteintes oculaires	Hommes 106 examinés	%	Femmes (84 examinées)	%	Total (190 exa' inés)	%
Karetite Ponctuée	9	8,5	21	25	30	15,8
Keratite sclérosante	0	0	0	0	0	0
Iridocyclite	2	1,9	2	2,4	4	2,1
Choriorétinite	2	1,9	0	0	2	1
Atrophie optique	3	2,8	2	2,4	5	2,6
Cécité	3	2,8	0	0	3	1,5
Baisse de l. A. visuel	13	12,2	14	16,6	27	14,2
Total	32	30,1	39	46,6	71	37,3

Source L'onchocercose au Congo (thèse de LOUZIEMI 1985)

../..

ENQUETE PROSPECTIVE

Notre enquête prospective s'est déroulée dans 5 villages. En effet, nous avons procédé à un dépistage systématique et exaustif des populations de cinq villages de la région du Pool jamais encore prospectés. Ces villages se trouvent tous dans le district de Boko ce sont :

1 - Foota
2 - Mantaba
3 - Kimpenga
4 - Bela
5 - Mandombé

Il convient de signaler que ces villages ont été retenus pour notre étude compte tenu de leur situation géographique (proche du fleuve Congo) ; et qu'ils n'avaient jamais été prospectés jusque là d'une part. D'autre part, l'existence dans la zone de nombreuses chutes et rapides tant sur le fleuve congo que sur ses affluents qui baignent la dite zone nous a fait préssentir la présence probable des simulies vectrices de la maladie.

3-2-1 DEROULEMENT DE L'ENQUETE

3-2-2 Personnel

Le personnel était composé de :

1 Technicien supérieur en santé publique sortant du C E D de l'OCEAC

1 Etudiant (T S S P) de l'OCEAC

1 chauffeur

3-2-3 MATERIEL

Matériel technique

2 microscopes
1 pince à SNIP (modèle NORIA)
10 plaques à microtitrations
200 lames porte objets
1 litre de sérum physiologique
1 rouleau de parafilm
200 pipettes pasteur
2 litres d'alcool à 90°
10 poires en caoutchouc.

../..

1 paquet de coton hydrophile
1 flacon d'exoseptoplix (poudre)
2000 fiches individuelles
1 cahier d'enregistrement
2 bics bleus
2 bics rouges.

Matériel logistique

1 véhicule tout terrain de marque TOYOTA (diesel)
200 litres de Gas-oil

3-2-4 METHODES ET TECHNIQUE

Nous avons utilisé le modèle de la fiche de l'OCEAC qui nous a permis de relever :

- l'identité du malade
- Nom et prénoms
- âge
- Sexe

Et de réaliser un interrogatoire succint
Nous avons en outre procéder à :

- un examen clinique
- un examen parasitologique
- un examen oculaire sommaire.

Examen clinique

L'examen clinique de chaque malade nous a permis de rechercher et de préciser le type de lésions cutanées, le nombre des nodules et leurs localisations par la palpation de chaque sujet déshabillé dans un isoloir ou dans une chambre.

Examen parasitologique

L'examen parasitologique a consisté à procéder au prélèvement de biopsie cutanée exsangue (BCE) quantitative aux deux crêtes iliaques de chaque sujets examinés pour la recherche de microfilaires d'onchocerca-volvulus et pour leur dénombrement.

Pour cette opération, nous avons utilisé la pince à prélèvement calibrée de 3 mm modèle MORIA
Deux biopsies ont été prélevées au niveau des deux crêtes iliaques, droite et gauche de chaque malade.

Milieu d'incubation et temps de lecture

Nous avons utilisé la méthode dite "Méthode de NELSON" : A l'aide d'une plaque à microtitrations dans laquelle était déposée une goutte d'eau physiologique et ceci dans chaque trou. Une personne représente deux trous.

Nous avons déposé le lambeau de peau prélevé à droite dans un trou et celui de gauche dans un autre.

Toute la plaque remplie était recouverte d'un papier spécial parafilm pour la protection. Les plaques étaient numérotées et les trous portaient le numéro de chaque malade.

La lecture se faisait 30 minutes à 1 heure après compte tenu du nombre important de la population qu'il nous fallait examiner à deux.

- Charges parasitaires

Nous avons calculé les charges parasitaires moyennes individuelles par tranches d'âge et par sexe en additionnant le nombre de microfilaires de BCE de droite et de gauche et en les divisant par le nombre de sujets porteurs de microfilaires de chaque tranche d'âges.

C'est la moyenne arithmétique du nombre de microfilaires émergées après incubation de 30 minutes en Eau physiologique des deux BCE calibrées des crêtes iliaques.

../..

Ces charges parasitaires moyennes calculées nous ont permis de mieux apprécier l'intensité de l'infestation des onchocerquiens et de mieux juger la corrélation existant entre cette densité microfilarienne moyenne (ou charge parasitaire moyenne) et les manifestations oculaires, ainsi qu'avec le syndrome kystique et les lésions cutanées.

Examen oculaire

Par manque de spécialiste d'ophtalmologie : nous avons pratiqué un examen oculaire sommaire comme le préconise l'OCEAC. Cet examen a consisté à faire compter les doigts de la main à tous nos sujets examinés à des distances variables (6 mètres et 3 mètres). En vue de préciser l'Etat de la fonction visuelle de chaque sujet examiné. Il est a noter que les principales perturbations de la vision ont été définies comme suit :

- La perte grave de la vue : nous l'avons définie comme étant l'impossibilité pour le malade de compter les doigts de la main à 3 mètres à la lumière du jour.

- Et la baisse de l'acuité visuelle : Par l'impossibilité pour le malade de les compter (les doigts de la main) à 6 mètres.

Définition des cas

Ont été retenus pour l'enquête :
- les porteurs de microfilaires
- les porteurs des nodules ou kystes
- les porteurs des lésions oculaires et troubles de la vue avec :
    - perte grave de la vue
    - baisse de l'acuité visuelle
    - Cécité totale

Nous avons également pu recenser le nombre des aveugles dans chaque village.

CHAPITRE IV

RESULTATS

C H A P I T R E IV RESULTATS

4-1 RESULTATS GLOBAUX

4-1-1 STRUCTURE D'ENSEMBLE DE LA POPULATION ETUDIEE

Au total, l'enquête a porté sur un échantillon de 1106 personnes examinées pour les 5 villages visités avec une répartition suivante :

Hommes = 470 soit 42,49 %
Femmes = 636 soit 57,50 % voir tableau IVX

Nous avons eu à examiner 464 enfants de moins de 15 ans soit 41,95 % de la population totale examinée et 642 adultes âgés de 15 à 50 ans et plus soit 58,04 % de la population totale examinée (voir tableau IVX ci-dessous

Tableau N° 14
Répartition de la population étudiée par village en fonction du sexe dans la zone Sud-Est de Boko (observations de la période du 18/11/85 au 26/11/85)

Villages	Population examinée	Hommes	%	Femmes	%
Foota	284	122	42,95	162	57,04
Mantaba	174	.85	48,[illegible]5	89	51,14
Kimpenga	304	147	48,35	157	51,64
Bela	137	53	38,68	84	61,31
Mandombe	207	63	30,43	144	69,56
Total	1 106	470	42,49	636	57,50

Tableau N° 15 Répartition de la population par village en fonction de l'"âge et du sexe dans la zone Sud-Est de Boko (observations de la période du 18/11/85 au 26/11?85)

Villages	Enfants de 0 - 14 ans				Adultes de 15-50 ans et +			
	H	F	H+F Total	%	Hommes	Femmes	Total H+F	%
Foota	74	69	143	50,35	49	92	141	49,64
Mantaba	41	27	68	39;08	44	62	106	60,91
Kimpenga	97	95	192	63,15	50	62	112	36,84
Bela	14	33	47	34,30	40	50	90	65,69
Mandombe	2	12	14	6,76	60	133	193	93,23
Total	228	236	464	41,95	243	399	642	58,04

H = Hommes
F = Femmes
H+F = Total Hommes + Femmes

Tableau N°16 Répartition de la population visitée par village en fonction de tranches d'âge et du sexe.

Villages \ Tranches d'âge	0 - 4 ans			5 - 9 ans			10-14 ans			15-29 ans			30-49 ans			50 ans et +			Total
	H	F	Total	H	F	Total	H	F	Total	H	F	Total	H	f	Total	H	F	Total	
Foota	0	0	0	36	43	79	38	26	64	6	13	19	8	28	36	35	51	86	284
Mantaba	2	3	5	19	11	30	20	13	33	12	17	29	11	22	33	21	23	44	174
Kimpenga	0	0	0	55	41	96	42	54	96	19	20	39	14	18	32	17	24	41	304
Bela	2	1	3	4	8	12	8	24	32	14	15	29	11	14	25	15	21	36	137
Mandombé	0	2	2	1	3	4	1	7	8	8	46	54	16	49	65	36	38	74	207
Total	4	6	10	115	106	221	109	124	233	59	111	170	60	131	191	124	157	281	1106

H = homme - F = Femmes - H + F = Hommes + Femmes

La répartition de la population étudiée entre différentes tranches d'âges groupées est représentée par le tableau XVI et la pyramide d'âge ci-après.

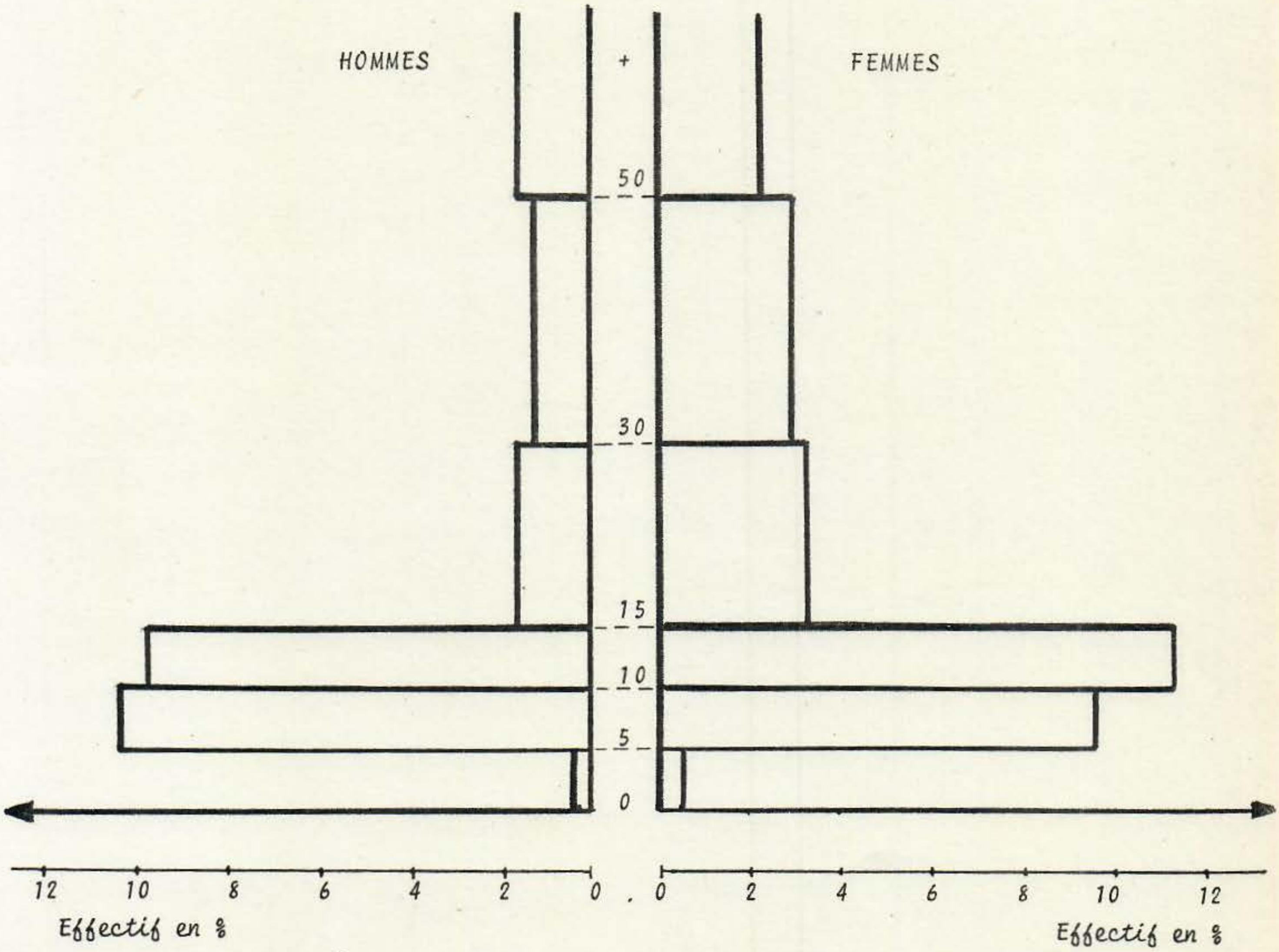

PYRAMIDE DES AGES DE LA POPULATION ETUDIEE POUR L'ENSEMBLE DES 5 VILLAGES (FOOTA-MANTABA-KIMPEÑGA-BELA-MANDEOMBE).
(*Observations de la période du 18 au 26 Novembre 1985*).

N.B. : *Nous signalons que la sensibilisation de la population était faite deux semaines avant par la Note de Service jointe en annexe que nous avions fait acheminer dans chacun des villages visités.-*

Le Nombre relativement bas des jeunes que nous avons eu à examiner dans cette zone explique le problème de l'exode rurale qui est beaucoup accentué dans notre pays.

Aussi cet exode rural ne peut toujours pas être incriminé par le seul fait que les jeunes vont en ville pour chercher du travail. Car il y a aussi ces problèmes de nuisances dues à certains arthropodes. Comme les simulies que l'on rencontre souvent dans certains villages visités et qui piquent les gens le long de la journée rendant ainsi la vie impossible à cause de la nuisance de leurs piqures.

Aussi pouvons-nous affirmer que non seulement les jeunes quittent les villages pour aller chercher du travail en ville, mais aussi pour échapper à la nuisance combien considérable que constitue dans ces villages l'agression constante des simulies

4-1-2 <u>PREVALENCE GLOBALE DE L'ONCHOCERCOSE DANS LES CINQ VILLAGES</u>

Les pourcentages de sujets porteurs des microfilaires d'onchocerques dépistés par biopsie cutanée exsangue (BCE) sont indiqués au tableau ci-après.

Ils montrent que par cette technique la prévalence de l'onchocercose dans toute la zone est comprise entre 68,32 et 91,30 % selon les villages.

La prévalence globale de la maladie dans toute la zone est de : au total 853 personnes sont porteurs

../..

de microfilaires d'onchocerques sur 1106 sujets examinés; soit un indice microfilarien (IM) de 77,12 % (853/1106).

Dont 339 sujets de sexe masculin porteurs de microfilaires sur 470 sujets examinés ; soit un indice microfilarien (IM) chez les hommes égal à 72,12 % (339/470.)

Et 85 personnes du même sexe c'est-à-dire du sexe masculin porteurs de nodules, soit un indice kystique (IK) chez les hommes égal à 18,04 % (85/471)

Par contre 510 sujets du sexe féminin sont porteurs de microfilaires sur un total de 636 femmes examinées soit un indice microfilarien (IM) chez les femmes de 80,18 % (510/636).

Et 146 sujets du même sexe (féminin) sont porteurs de nodules sur 636 examinées (du même sexe), soit un indice kystique (IK) chez les femmes égal à 22,95 % (146/636).

En outre, nous avons dépisté 277 enfants de moins de 15 ans qui sont porteurs de microfilaires d'onchocerques sur un total de 464 enfants examinés, soit un indice microfilarien de 59,69 % pour les enfants,

13 sujets seulement d'entre eux (les enfants de moins de 15 ans) sont porteurs de nodules, soit un indice kystique (IK) égal à 2,80 % chez les enfants.

Par contre 576 sujets de 15 à 50 ans et plus sont porteurs de microfilaires sur 642 adultes visités soit un indice crofilarien de 89,71 % (576/642) chez les

adultes.

Et 218 sujets de la même tranche d'âge (c'est -à-dire de 15 à 50 ans et plus sont porteurs de nodules soit un indice kystique (IK) chez les adultes de 33,95 % (218/642)

Atteintes oculaires

Au total dans l'ensemble de la zone, nous avons observé 64 personnes souffrant d'une perte de vue grave, soit un pourcentage global de 5,78% pour les 5 villages visités :

- Nous avons pu en outre recensé 13 aveugles dans les 5 villages, soit un taux de cécité global de 1,17 % (13/ 1106

.

Manifestations cutanées

Nous avons décelé 160 personnes porteurs des lésions cutanées, soit un pourcentage de 14,46 % pour l'ensemble de la population des 5 villages visités. (voir tableau N° 17 ci-dessous.)

Tableau N° 17 Prévalence globale de l'onchocercose en fonction du sexe dans la zone Sud-Est de Boko (village Foota-Mantaba-Kimpenga-Bela Mandombé) (observations de la période du 18 au 26 Novembre 1985)

Population visitée			Porteurs de microfilaires			Porteurs de kystes			Porteurs des lésions cutanées			Perte de vision grave			Cécité		
H	F	Total	H	F	Total H+F	H	F	Total H+F	H	F	Total H+F	H	F	Total H+F	H	F	Total
470	636	1106	339	510	849	85	146	231	44	48	92	17	36	53	4	8	12

Tableau N° 18 : Valeur des paramètres exprimant la prévalence de l'onchocercose dans la population humaine par village dans la zone Sud-Est de Boko en fonction du sexe. (Observations de la période du 18 au 26 Novembre 1985)

Villages / Paramètres	Foota		Mantaba		Kimpenga		Bela		Mandombé	
	H	F	H	F	H	F	H	F	H	F
I.M. en %	60,97	74,53	75,29	78,65	70,06	76,43	83,33	80,72	90,32	91,72
I.K. %	10,56	13,66	24,70	20,22	8,84	19,74	27,77	20,48	37,09	39,31
I C P %	62,60	74,53	76,47	79,77	72,78	77,07	85,18	80,72	95,16	92,41
Taux de cécité %	0,81	0,62	0	0	0,68	0,63	1,85	4,81	1,61	1,37

I.M. = Indice microfilarien
I.K = Indice Kystique
I.C.P = Indice Clinico-parasitologique
H = Hommes
F = Femmes

PRESENTATION ET ANALYSE

DES RESULTATS

PAR FOYER

4-2 PRESENTATION ET ANALYSE DES RESULTATS PAR FOYER

4-2-1 FOYER DE FOOTA

Le village Foota est situé au bord du fleuve Congo à quelques 90 kilomètres de Brazzaville. Il est bâti à environ 3 kilomètres du fleuve.

Structure de la population étudiée

A Foota, nous avons eu à visiter 284 personnes sur 375 recensées (recensement de 1984 d'après les archives du district de Boko) soit un taux de présentation de 75,73 %. La population est répartie comme suit :

Hommes = 123 soit un pourcentage de 43,30 %

Femmes = 161 soit un pourcentage de 56,69 %

Les sujets âgés de moins de 15 ans sont au nombre de 143 soit 50,35 % de la population totale examinée.
Et ceux âgés de 15 à 50 ans et plus sont au nombre de 141 soit 49,64 % de la population totale examinée.
On remarque qu'à Foota ; les proportions entre les âges c'est-à-dire entre les enfants et les adultes sont presque identiques (voir tableau N° 20 ci-après).

Tableau N° 19

Répartition de la population en fonction du sexe à Foota (observations de la période du 18 au 26 Novembre 1985)

Population examinée	Hommes	%	Femmes	%
284	123	42,95	161	57,04

../..

Tableau N° 20 / *Répartition de la population en fonction de l'âge et du sexe à Foota (observations de la période du 18 au 26 Novembre 1985)*

Tranches d'âges \ Sexe	Hommes	Femmes	Total	%
Enfants 0 - 14 ans	74	69	143	50,35
Adultes 15 - 50 ans et +	49	92	141	49,64
Total	123	161	284	100

Résultats cliniques

43 personnes parmi les examinés (284 examinés) soit 15,14 % présentent au moins un des signes cliniques majeurs de l'onchocercose : (nodules, signes oculaires et lésions cutanées); Le tableau N° 21 ci-dessous résume les résultats retrouvés qui relèvent plusieurs aspects

../..

Tableau N° 21 Répartition des signes cliniques observés à Foota pendant la période du 18 au 26 Novembre 1985)

Tranches d'âges	Sujets examinés	Porteurs de Nodules	Porteurs de lésions cutanées				Atteintes Oculaires	
			Dépigmentation tibiale	Atrophie cutanée	Gale filarienne	Pachydermie	Perte de vue grave	Cécité
0-4 ans	0	0	0	0	0	0	0	0
5-9 ans	79	3	0	0	0	0	0	0
10-14 ans	64	2	0	0	0	0	0	0
15-29 ans	19	1	1	0	0	0	0	0
30-49 ans	36	11	3	0	0	0	0	0
50 et +	86	18	21	15	1	4	5	3
Total	284	35	25	15	1	4	5	3

Premier Aspect

Dans l'ensemble, les manifestations cliniques sont rares chez les enfants de 0 - 14 ans. Le seul signe que nous avons rencontré chez quelques enfants est la présence de nodules. Et le signe le plus fréquemment rencontré tant chez les enfants que chez les adultes est le prurit.

../..

Deuxième Aspect

Nous avons remarqué que les lésions cutanées sont absentes chez les enfants de 0 - 14 ans.

Troisième Aspect

Le tableau N° 22 ci-après nous résume ce troisième aspect.

Tableau NO 22 = Répartition de porteurs de nodules onchocerquiens en fonction du sexe et de la topographie à Foota (observations de la période du 18 au 26 Novembre 1985)

Localisations des nodules / Sexe des porteurs	Nodules Céphaliques	Nodules thoraciques	Nodules pelviens	Total
Porteurs masculins	2	0	12	14
Porteurs féminins	1	1	20	22
Total	3	1	32	36

Au total 36 personnes sont porteurs de nodules sur 284 examinés, soit un indice kystique (IK) de 12,67 % (36/284). Nous remarquons ici qu'il y a prédominance des porteurs de nodules pelviens. Car sur un total de 36 porteurs de nodules à Foota 32 sont porteurs de nodules pelviens : soit un pourcentage de nodules pelviens de 88,88 %. 3 personnes seulement sont porteurs de nodules céphaliques dont 2 hommes et 1 femme, soit un pourcentage de nodules céphaliques de 8,33 % . Et 1 seule personne est alors porteur de nodules thoraciques, soit un

../..

pourcentage de nodules thoraciques de 2,77 %.
L'Indice kystique chez les femmes à Foota est de 13,66 % (22/161) contre 11,38 % chez les hommes (14/123)

Quatrième aspect

L'examen oculaire sommaire pratiqué à Foota a révélé l'existence des atteintes oculaires chez 25 personnes dont 17 avec une baisse d'acuité visuelle. et 8 avec perte grave de la vue.
Parmi les 8 sujets avec perte grave de la vue, nous avons décélé 3 personnes avec cécité.
Une seule personne est porteur de nodules onchocerquiens parmi les 3 aveugles, la même personne fait une cécité bilatérale, alors que les 2 autres ne font qu'une cécité unilatérale à qui l'origine onchocerquienne peut être mise en doute.

Le taux de cécité à Foota est donc de 1,05 % (3/284). Il est à noter que tous les 22 sujets présentant une atteinte oculaire ont tous une B C E positive.

Résultats parasitologiques

Le tableau N° 23 ci-dessous nous résume les résultats parasitologiques à Foota.

BCE = Biopsie cutanée exsangue

../..

Tableau N° 13 : Pévalence de l'onchocercose en fonction de l'âge et du sexe à Foota (observations de la période du 18 au 26 Novembre 1985)

Tranches d'âges	Sujets examinés			Nombre d'onchocerquiens dépistés parasitologiquement					
	Hommes	Femmes	Total	Hommes	% IM	Femmes	% IM	Total H + F	IM %
0 - 4 ans	0	0	0	0	0	0	0	0	0
5 - 9 ans	36	43	79	18	50	22	51,16	40	50,63
10 - 14 ans	38	26	64	15	39,47	14	53,84	29	45,31
15 - 29 ans	6	13	19	4	66,66	12	92,30	16	84,21
30 - 49 ans	8	28	36	8	100	25	89,28	33	91,66
50 ans et +	35	51	86	30	85,71	47	92,15	77	89,53
Total	123	161	284	75	60,97	120	74,53	195	68,66

Sur 284 sujets examinés à Foota, 195 sont porteurs des microfialires d'onchocerques, soit un indice microfilarien (IM) de 68,66 % : dont 75 sujets de sexe masculin soit un indice microfilarien (IM) chez les hommes de 60,97 % (75/123). ET 120 de sexe féminin soit un indice microfilarien de 74,53 % (120/161) chez les femmes.

-69 enfants âgés de 0 -14 ans sont porteurs de microfilaires d'onchocerques sur un total de 143 enfants examinés, soit un indice microfi lárien de 48,25 % chez les enfants (69/143). Notre plus jeune enfant porteur de microfilaires à Foota est âgé de 5 ans. Nous avons eu donc à Foota au total 195 sujets porteurs soit uniquement des microfilaires soit en même temps porteurs de microfilaires et kystes. ET 2 personnes seulement sont porteurs uniquement de kystes soit un indice clinico-parasitologique (ICP) de 69,36 % (197/284).

Charges parasitaires

Tableau N° 24 Distribution des charges parasitaires en fonction du sexe et de l'âge à Foota (observations de la période du 18 au 26 Novembre 1985)

Tranches d'âges	Sexe masculin				Sexe féminin				Ensemble Mas.+Fém.			
	Sujets examinés	Cas +	CPT	CPM	Sujets examinés	Cas +	CPT	CPM	S.E.	Cas +	CPT	CPM
0- 4 ans	0	0	0	0	0	0	0	0	0	0	0	0
5 - 9 ans	36	18	414	23	43	22	446	20,27	79	40	860	21,5
10 - 14 ans	38	15	354	23,6	26	14	262	18,71	64	29	616	21,24
15 - 29 ans	6	4	75	18,75	13	12	1023	85,25	19	16	1098	68,62
30 - 49 ans	8	8	995	124,37	28	25	1034	41,36	36	33	2029	61,48
50 ans et +	35	30	2260	75,33	51	47	2781	59,17	86	77	5041	65,46
Total	123	75	4098	54,64	161	120	5546	46,21	284	195	9644	49,45

CPT = charge parasitaire totale
CPM = Charge parasitaire Moyenne
S.E = Sujets examinés

Le tableau ci-dessus nous résume l'intensité de l'infestation par onchocerca-volvulus : à Foota Nous remarquons en effet qu'à Foota la charge parasitaire moyenne pour l'ensemble de la population examinée et porteur des microfilaires d'onchocerques est de 49,45 . Et que les hommes hébergent beaucoup plus de microfilaires soit une charge parasitaire de 54,65 que les femmes qui n'ont que 46,21.

La tranche d'âge qui héberge plus de microfilaires chez les hommes est celle de 36-49 ans. Par contre chez les femmes c'est la tranche d'âge de 15-29 ans qui héberge. . beaucoup plus de microfilaires.

4-2-2 FOYER DE MANTABA

Le village Mantaba est situé à environ 120 kilomètres de Brazzaville (par piste). Il est distant de Foota d'environ 30 kilomètres. Il est bâti au bord de la rivière la Nkissi qui est un affluent du fleuve Congo. La distance qui sépare le village et la rivière est d'environ 1 kilomètre.

Structure de la population étudiée

- A Mantaba, un total de 174 personnes a été examiné sur 234 recensés (d'après les archives du district de Boko : recensement de 1984) soit un taux de présentation de 74,35 %.

Les sujets examinés sont répartis comme suit :

- Hommes = 85 soit 48,85 % de la population totale examinée
- Femmes = 89 soit 51,14 % de la population totale examinée

dont : enfants de 0-14 ans = 68 soit 39,08 %
(voir tableau ci-dessous)

Tableau N°25 : Répartition de la population en fonction du sexe à Mantaba (observations de la période du 18 au 26 Novembre 1985)

Sujets examinés	Hommes	%	Femmes	%
174	85	48,85	89	51,14

Tableau N° 26 Répartition de la population en fonction de l'âge et du sexe à Mantaba (observations de la période du 18 au 26 Novembre 1985)

Tranches d'âges	Hommes	Femmes	Total	%
Enfants 0 - 14 ans	41	27	68	39,08
Adultes 15 - 5.0 ans et +	44	62	106	60,91
Total	85	89	174	100

La composition de la population visitée à Mantaba selon l'âge et le sexe (voir tableaux N° 25-26) montre qu'il y a presque autant d'hommes que de femmes. Et la population infantile ne réprésente que 39,08 % de la population totale examinée.

Résultats cliniques

A Mantaba, sur 174 sujets exminés : 56 d'entre eux soit 32,18 % présentent au moins un signe clinique majeur de l'onchocercose le tableau ci-dessous résume les résultats observés et révèle 4 aspects principaux.

Tableau N° 27 Répartition des signes cliniques en fonction de l'âge à Mantaba (observations de la période du 18 au 26 Novembre 1985)

Tranches d'âges	Sujets examinés	Porteurs de nodules	Porteurs des lésions cutanées				Atteintes oculaires	
			dépigmentation	Atrophie	Gale filarienne	Pachydermie	Perte de vue grave	Cécité
0 - 4 ans	5	2	0	0	0	0	0	0
5 - 9 ans	30	1	0	0	0	0	0	0
10 - 14 ans	33	2	0	0	0	0	0	0
15 - 29 ans	29	2	0	0	0	0	0	0
30 - 49 ans	33	12	3	1	0	0	0	0
50 -et +	44	20	9	13	0	1	5	0
Total	174	39	12	14	0	1	5	0

1er Aspect

Nous remarquons ici qu'en dehors des nodules onchocerquiens et du prurit que l'on rencontre jusqu'à certains enfants, aucun autre signe patent ne se manifeste chez les jeunes gens jusqu'à l'âge de 30 ans environ.

2e Aspect

Le tableau N° 28 nous résume le 2e aspect.

Tableau N° 28 Répartition des porteurs de nodules onchocerquiens en fonction du sexe et de la topographie à Mantaba (observations de la période du 18 au 26 Novembre 1985)

Localisations des nodules / Sexe des porteurs	Nodules Céphaliques	Nodules thoraciques	Nodules Pelviens	Total
Porteurs masculins (nombre)	2	2	17	21
Porteurs féminins (nombre)	2	2	14	18
Total	4	4	31	39

Nous avons ainsi remarqué que : du point de vue de la répartition des nodules par sexe, il n'y a presque pas de différence significative entre le nombre de porteurs masculins et les porteurs féminins.

Au total 39 personnes sont porteurs de nodules dont : 21 du sexe masculin et 18 du sexe féminin sur 174 examinés. La seule différence que nous avons observée se situe au niveau de la topographie des nodules. Il y a également ici prédominance des nodules pelviens, puisque nous avons rien que pour les nodules pelviens 31 porteurs sur un total de 39 porteurs de nodules de toutes topographies : dont 17 de sexe masculin et 14 de sexe féminin. Soit un pourcentage de porteurs de nodules pelviens de 79,48 % (31/39) : alors qu'on a seulement 4 porteurs de nodules thoraciques et 4 autres porteurs de nodules céphaliques soit un pourcentage de 10,25 % pour chacune des 2 topographies ; (céphaliques et thoraciques)

- L'indice kystique global (IK) à Mantaba est donc de 22,41% dont 24,70 % (21/85) pour les hommes et 20,22 % (18/89) pour les femmes.

3e Aspect :

Le troisième aspect nous a révélé l'inexistence à mantaba de sujets avec cécité. Donc le taux de cécité est de zéro à Mantaba (nul).

En effet, nous n'avons recensé aucun aveugle dans ce village en dehors de quelques 11 personnes qui accusent des troubles de la vision dont 6 avec baisse d'acuité visuelle et 5 avec perte de vue grave.

4e Aspect

A mantaba les lésions cutanées sont absentes chez les jeunes personnes âgées de 0-29 ans.

Résultats parasitologiques

Le tableau N° 29 ci-après nous montre la répartition de la parasitose onchocerquienne à Mantaba.

Tableau N° 29=Prévalence de l'onchocercose en fonction de l'âge et du sexe à Mantaba (observations de la période du 18 au 26 Novembre 1985)

Tranches d'âges	Sujets examinés			Nombre d'onchocerquiens dépistés parasitologiquement					
	Hommes	Femmes	Total	Hommes	IM %	Femmes	IM %	Total	IM %
0 - 4 ans	2	3	5	1	50	1	33,33	2	40
5 - 9 ans	19	11	30	11	57,89	6	54,54	17	56,66
10 - 14 ans	20	13	33	12	60	9	69,23	21	63,63
15 -29 ans	12	17	29	10	83,33	13	76,47	23	79,31
30 -49 ans	11	22	33	10	90,90	20	90,90	30	90,90
50 ans et +	21	23	44	20	95,23	21	91,30	41	93,18
Total	85	89	174	64	75,29	70	78,65	134	77,01

IM = Indice microfilarien

Sur 174 sujets examinés à Mantaba, 134 sont porteurs de microfilaires d'onchocerques, soit un indice microfilarien (IM) de 77,01 % (134/174) dont 64 sujets de sexe masculin, soit un indice microfilarien (IM) chez les hommes de 75,29 % (64/85) et 70 sujets de sexe féminin, soit un indice microfilarien (IM) chez les femmes de 78,65 % (70/89).

40 Enfants de la tranche d'âges de 0-14 ans sont porteurs de microfilaires d'onchocerques, soit un indice microfilarien chez les enfants de 58,82 % (40/68).
Il est à noter que les 134 onchocerquiens dépistés à Mantaba : quelques uns sont porteurs uniquement des microfilaires d'onchocerca-volvulus et d'autres porteurs à la fois de microfilaires et de kystes.

Par contre 2 autres personnes considérées comme onchocerquiens sont uniquement porteurs de kystes onchocerquiens d'où l'indice clinico-parasitologique (ICP) à Mantaba de 78,16 % (136/174). Nous avons remarqué que notre plus jeune enfant porteur de microfilaires d'onchocerca-volvulus à Mantaba est âgé de 2 ans.

Charges parasitaires : La charge parasitaire à Mantaba est résumée par le tableau 30 ci-après.
Tableau 30 Distribution des charges parasitaires à Mantaba en fonction de l'âge et du sexe. (observations de la période du 18 au 26 Novembre 9185)

Tranches d'âges	Sexe Masculin				Sexe Féminin				Ensemble Sexe masculin+sexe F.			
	Sujets examinés	Cas +	CPT	CPM	Sujets examinés	Cas +	CPT	CPM	Sujets exami.	Cas +	CPT	CPM
0 - 4 ans	2	1	19	19	3	1	19	19	5	2	38	19
5 - 9 ans	19	11	396	36	11	6	341	56,83	30	17	737	43,35
10 - 14 ANS	20	12	246	20,5	13	9	389	43,22	33	21	635	30,25
15 - 29 ans	12	10	1081	108,1	17	13	828	63,69	29	23	1909	83
30 -49 ans	11	10	2385	238,5	22	20	2025	101,25	33	30	4410	147
50-ans et +	21	20	3653	182,65	23	21	1048	49,90	44	41	4701	114,65
Total	85	64	7786	121,56	89	70	4650	66,42	174	134	12430	92,76

CPT = Charge parasitaire totale
CPM = Charge parasitaire moyenne

Nous remarquons sur le tableau des charges parasitiares à Mantaba ci-dessus que la charge parasitaire moyenne pour l'ensemble des porteurs de microfilaire dans ce village est de 92,76.

Et que la charge parasitaire moyenne des hommes est plus élevée 121,56 que celle des femmes qui n'est que de 66,42. La tranche d'âge qui héberge beaucoup plus de microfilaires tant chez les hommes que chez les femmes est celle de 30-49 ans avec chez les hommes une charge microfilarienne moyenne de 238,5 et chez les femmes la charge microfilarienne moyenne pour la même tranche d'âge est de 101,25.

4-2-3 FOYER DE KIMPENGA

Le Village Kimpenga encore appelé MOWA est situé à environ 140 kilomètres de Brazzaville (par piste). Il est bâti à une distance d'environ 7 kilomètres du fleuve Congo. La distance qui le sépare de Mantaba est d'environ 20 kilomètres.

Structure de la Population étudiée

A Kimpenga, nous avons eu à visiter 304 personnes sur 410 recensés (archives du district de Boko : recensement de 1984) soit un taux de présentation de 74,14 % . Parmi les personnes examinées ; nous avons :

147 hommes soit 48,35 % de la population totale visitée et 157 femmes soit 51,64 % de la population totale visitée. Les sujets de moins de 15 ans étaient au nombre de 192 soit 63,15 % de la population visitée.

Et ceux âgés de plus de 15 ans (adultes) étaient au total 112 soit 36,84 %.

Tableau N° 31 :Répartition de la population en fonction du sexe à Kimpenga (observations de la période du 18 au 26 Novembre 1985);

Sujets examinés	Hommes	%	Femmes	%
304	147	48,35	157	51,64

Tableau N° 32 / Répartition de la population en fonction de l'âge et du sexe à Kimpenga (observations de la période du 18 au 26 Novembre 1985)

Sexe / Tranches d'âges	Hommes	Femmes	Total Hommes + femmes	%
Enfants 0 - 14 ans	97	95	192	63,15
Adultes 15 - 50 ans et +	50	62	112	36,84
Total	147	157	304	100

A Kimpenga également la composition de la population visitée nous montre selon la répartition par sexe qu'il y a eu presque autant d'hommes que de femmes. Par contre la répartition par âge nous montre que le nombre d'enfants de moins de 15 ans dépasse légèrement celui des adultes. (Voir tableau N° 32)

Résultats cliniques

Sur 304 sujets examinés à Kimpenga, 88 personnes soit 28,94 % présentent au moins un signe clinique majeur de l'onchocercose (nodules, signes oculaires, lésions cutanées).
Le tableau ci-dessous résume les résultats obtenus.

Tableau N° 33

Répartition des signes cliniques en fonction de l'âge à Kimpenga (observations de la période du 18 au 26 Novembre 1985)

Tranches d'âges	Sujets examinés	Porteurs de nodules	Porteurs de lésions cutanées				Atteintes oculaires	
			Dépigmentation tibiale	Atrophie cutanée	Gale filarienne	Pachydermie	Perte de vue grave	Cécité
0 - 4 ans	0	0	0	0	0	0	0	0
5 - 9 ans	96	1	0	0	0	0	0	0
10 - 14 ans	96	1	0	0	0	0	0	0
15 - 29 ans	39	5	1	0	0	0	0	0
30 - 49 ans	32	14	3	1	0	0	2	0
50 et +	41	22	22	9	1	10	14	2
Total	304	43	26	10	1	10	16	2

N B. : Pour la dépigmentation : il s'agit de la dépigmentation tibiale.

Nous constatons encore ici que les mêmes observations signalées dans les deux précédents villages se retrouvent ici également, c'est-à-dire : absence de signes cliniques patents de l'onchocercose chez les enfants jusqu'à l'âge au moins de 15 ans, mis à part les nodules onchocerquiens et le prurit qui sont rencontrés à tout âge.
Quant à la localisation des nodules : (voir tableau N°34 ci-dessaus)

Tableau N° 34 : Répartttion des porteurs de nodules en fonction du sexe et de la topographie à Kimpenga (observations de la période du 18 au 26 Novembre 1985)

Localisations des nodules / Sexe des porteurs	Nodules Céphaliques	Nodules thoraciques	Nodules Pelviens	Total
Porteurs masculins (Nbre)	0	2	11	13
Porteurs féminins (Nbre)	1	2	27	30
Total	1	4	38	43

Ici, nous avons observé une nette différence quant à la répartition des nodules selon le sexe.
Nous remarquons en effet que sur 157 sujets de sexe féminin examinés, 30 sont porteurs de nodules, soit un indice kystique (IK) de 19,10 % chez les femmes.

Par contre sur 147 sujets de sexe masculin examinés il n'y a que 13 qui sont porteurs de nodules onchocerquiens, soit un indice kystique (IK) de 8,84 % chez les hommes.

Quant à la répartition selon l'âge, nous avons remarqué que sur 192 enfants âgés de 0-14 ans examinés, il n'y a que 2 qui sont porteurs de nodules, soit un indice kystique (IK) de 1,04 % chez les enfants (2/192) contre 36,60 % (42/112) d'indice kystique (IK) chez les adultes dont l'âge va de 15 à 50 ans et plus

Nbre = Nombre

Ici également nous remarquons la nette prédominance de nodules pelviens. 38 porteurs de nodules pelviens pour un total de 43 porteurs de nodules de toutes topographies soit un pourcentage de porteurs de nodules pelviens de 88,37 % (voir tableau N°34 cidessus).

Au total 43 personnes sont porteurs de nodules onchocerquiens à Kimpenga soit un indice kystique global de 14,14 % (43/304)
Et sur 43 porteurs de nodules, nous avons dit qu'il y a 38 qui sont porteurs de nodules pelviens soit un pourcentage de 88,37 % (38/43) alors qu'il n'y a qu'une seule personne (1 personne) porteur de nodules céphaliques et 4 pour les nodules thoraciques, soit un pourcentage respectivement de 2,32 % (1/43) et 9,30 % (4/43)

Comme dans les deux précédents villages, à KImpenga également nous n'avons pas observé de lésions cutanées chez les enfants âgés de 0- 14 ans.

Enfin la dernière observation sur le plan clinique dans ce village concerne les atteintes oculaires. L'examen oculaire sommaire que nous avons pratiqué nous a permis de déceler 5 sujets avec baisse de l'acuité visuelle et 18 avec perte de vue grave dont 2 avec cécité totale.

Le taux de cécité à Kimpenga est donc de 0,65 % (2/304)

../..

Résultats Parasitologiques :

Tableau N° 35 Prévalence de l'onchocercose en fonction de l'âge et du sexe à Kimpenga (observations de la période du 18 au 26 Novembre 1985)

Tranches d'âges	Sujets examinés			Nombre d'onchocerquiens dépistés parasitologiquement					
	Hommes	Femmes	Total	Hommes	%	Femmes	%	Total	%
0 - 4 ans	0	0	0	0	0	0	0	0	0
5 - 9 ans	55	41	96	31	56,36	20	48,78	51	53,12
10 - 14 ans	42	54	96	32	76,19	42	77,77	74	77,08
15 - 29 ans	19	20	39	13	68,42	17	85	30	76,92
30 - 49 ans	14	18	32	12	85;71	17	94,44	29	90,62
50 ans et +	17	24	41	15	88,23	24	100	39	95,12
Total	147	157	304	103	70,06	120	76,43	223	73,35

Le tableau N° 35 ci-dessus nous montre la répartition de la parasitose onchocerquienne à Kimpenga.
Les examens parasitologiques des BCE (biopsies cutanées exsangues) nous ont permis de dépister 223 sujets porteurs de microfilaires d'onchocerca-volvulus sur 304 sujets examinés, soit un indice microfilarien (IM) de 73,35 % (223/304).
Parmi les 223 sujets positifs : 103 sont de sexe masculin soit un indice microfilarien (IM) de 70,06 % (103/147) chez les hommes.
ET les 120 autres sont de sexe féminin soit un indice microfilarien (IM) de 76,43 % (120/157) chez les femmes.

Et 125 enfants âgés de 0-14 ans sont porteurs de microfilaires d'onchocerques sur un total de 192 enfants examinés de la même tranche d'âges; soit un indice microfilarien (IM) chez les enfants de 65,10 % (125/192).
Contre 98 adultes (âgés de 15-50 ans et plus) porteurs de microfilaires d'onchocerques, sur un total de 112 adultes examinés. soit un indice microfilarien (IM) de 87,5 % chez les adultes.

Nous signalons que sur les 223 sujets dépistés parasitologiquement : certains sont porteurs uniquement de microfilaires d'<u>onchocerca-volvulus</u>, d'autres sont porteurs à la fois de microfilaires et de kystes.

Par contre 5 autres personnes considérées onchocerquiens sont uniquement porteurs de kystes.

D'où l'indice clinico-parasitologique (ICP) à Kimpenga de 75 % (228/304)

Le plus jeune enfant porteur de microfilaires d'onchocerques à Kimpenga est âgé de 6 ans.

<u>Tableau N° 36</u> : Distribution des Charges parasitaires en fonction de l'âge et du sexe à Kimpenga (observations de la période du 18 au 26 Novembre 1985)

Tranches d'âges	Sexe Masculin				Sexe Féminin				Ensemble Sexe masculin+sexe Féminin			
	Examinés	Cas +	CPT	CPM	Examinés	Cas +	CPT	CPM	Exa.	Cas +	CPT	CPM
0 - 4 ans	0	0	0	0	0	0	0	0	0	0	0	0
5 - 9 ans	55	31	761	24,54	41	20	422	21,1	96	51	1183	23,19
10 -14 ans	42	32	1327	41,46	54	42	1156	27,52	96	74	2483	33,55
15 - 29 ans	19	13	744	57,23	20	17	1580	92,94	39	30	2324	77,46
30 - 49 ans	14	12	873	72,75	18	17	1503	88,41	32	29	2376	81,93
50 ans et +	17	15	1789	119,26	24	24	1855	77,29	41	39	3644	93,43
Total	147	103	5494	53,33	157	120	6516	54,3	304	223	12010	53,85

Les charges parasitaires à Kimpenga sont résumés par le tableau N° 36 ci-dessus et qui nous montre une charge parasitaire moyenne pour l'ensemble du village de 53,85. Nous remarquons ici que la charge parasitaire moyenne est presque la même tant chez les femmes que chez les hommes avec pour les hommes, une charge parasitaire moyenne de 53,33 et pour les femmes 54,3.

../..

Dans ce village les tranches d'âges qui hébergent beaucoup plus de microfilaires sont celles de 50 ans et plus chez les hommes avec une charge parasitaire moyenne de 119,26. Et la tranche d'âges de 15-29 ans chez les femmes avec une charge parasitaire moyenne de 92,94.

Le village Bela est situé à environ 175 kilomètres de Brazzaville. Il est bâti au bord de la rivière Louvoubi qui est un affluent du fleuve Congo. La distance qui le sépare de la rivière est d'environ 2 kilomètres. Bela est distant des chûtes de la Louvoubi d'environ 5 kilomètres.

STRUCTURE DE LA POPULATION ETUDIEE

A bela, un total de 137 personnes a été examiné dont 54 hommes soit 39,41 % de la population totale examinée et 83 femmes soit 60,58 % de la population totale examinée.
Les sujets de moins de 15 ans étaient au nombre de 47 soit un pourcentage de 34,30 %..
Et ceux âgés de 15-50 ans et plus (adultes) au nombre de 90 soit 65,69 % de la population totale examinée.

A Bela, nous avons donc examiné plus de femmes que d'hommes et plus d'adultes que de jeunes enfants (voir tableaux 37 et 38).

Tableau N°37 : Répartition de la population en fonction du sexe à Bela (observations de la période du 18 au 26 Novembre 1985)

Sujets examinés	Hommes	%	Femmes	%
137	54	39,41	83	60,58

Tableau N° 38 : Répartition de la population en fonction de l'âge et du sexe à Bela (observations de la période du 18 au 26 Novembre 1985)

Sexe / Tranches d'âges	Hommes	Femmes	Total Hommes+Femmes	%
Enfants 0 - 14 ans	14	33	47	34,30
Adultes 15 - 50 ans et plus	40	50	90	65,69
Total	54	83	137	100

Les deux tableaux de répartition de la population visitée nous montrent qu'à Bela nous avons eu à examiner beaucoup plus de femmes que d'hommes tout comme dans les villages précédents. Et la population infantile visitée correspond presque à la moitié des adultes visités.

RESULTATS CLINIQUES

Sur 137 personnes examinées à Bela : 54 personnes soit 39,41 % présentent au moins un signe clinique majeur de l'onchocercose (nodules, signes oculaires, lésions cutanées). Le tableau N°39 ci-dessous résume les résultats obtenus.

Tableau N° 39 Répartition des signes cliniques en fonction de l'âge à Bela (observations de la période du 18 au 26 Novembre 1985)

Tranches d'âges	Sujets examinés	Porteurs de nodules	Porteurs de lésions cutanées				Atteintes oculaires	
			Dépigmentation tibiale	Atrophie cutanée	Gale filarienne	Pachydermie	Perte de vue grave	Cécité
0-4 ans	3	1	0	0	0	0	0	0
5-9 ans	12	0	0	0	0	0	0	0
10-14 ans	32	0	0	0	1	0	0	0
15 -29 ans	29	5	0	0	0	0	0	0
30-49 ans	25	11	1	0	0	0	0	2
50 et +	36	16	5	5	0	4	6	3
Total	137	33	6	5	1	4	6	5

Sur 47 enfants âgés de 0 - 14 ans examinés : 2 seulement soit 4,25 % sont porteurs d'au moins un signe clinique majeur de l'onchocercose. L'un est porteur de nodules onchocerquiens et l'autre porteur de lésions cutanées (gale filarienne).
Ce village enfin fait exception quant à la présence des signes cliniques chez les enfants. ../.

NB : Pour la dépigmentation : il s'agit de la dépigmentation tibiale.

Le tableau N° 40 ci-après nous montre la répartition des nodules onchocerquiens à Bela.

Tableau N° 40 : Répartition des porteurs de nodules en fonction du sexe et de la topographie à Bela (observations de la période du 18 au 26 Novembre 1985)

Localisations des nodules / SExe des porteurs	Nodules céphaliques	Nodules thoraciques	Nodules pelviens	Total	%
Porteurs masculins	1	0	13	14	25,92
Porteurs féminins	1	0	18	19	22,89
Total	2	0	31	33	24,08

Nous remarquons que : le nombre de porteurs de nodules est de 33 à Bela sur 137 personnes examinées ; soit un indice kystique (IK) global de 24,08 % (33/137).
Parmi eux il y a 14 du sexe masculin, soit un indice kystique (IK) chez les hommes de 25,92 %.

Et 19 du sexe féminin soit un indice kystique (IK) chez les femmes de 22,89 %.

A Bela comme dans tous les autres précédents villages, les porteurs de nodules pelviens prédominent en nombre, tant chez les hommes que chez les femmes. Car nous avons eu 31 porteurs de nodules pelviens sur un total de porteurs de nodules de 33. soit un pourcentage de porteurs de nodules pelviens de 93,93 %. Donc 2 personnes seulement dont 1 homme et 1 femme sont porteurs de nodules céphaliques soit 6,06 %
Par contre, aucune personne n'est déclarée porteurs de nodules thoraciques à Bela.

Mais l'aspect le plus frappant quant aux signes cliniques de l'onchocercose dans ce village est celui des atteintes oculaires.

En effets, sur 137 sujets examinés à Bela, 7 présentent une baisse de l'acuité visuelle, et 11 autres présentent une

perte de vue grave dont 5 avec cécité totale. soit un taux de cécité pour ce seul village égal à 3,64 % (5/137). C'est le village le plus touché du point de vue atteintes oculaires.

RESULTATS PARASITOLOGIQUES

Tableau N° 41 : Prévalence de l'onchocercose en fonction de l'âge et du sexe à Bela (observations de la période du 18 au 26 Novembre 1985)

Tranches d'âges	Sujets examinés			Nombre d'onchocerquiens dépistés parasitologiquement					
	Hommes	Femmes	Total	Hommes	%	femmes	%	Total	%
0-4 ans	2	1	3	1	50	1	100	2	66,66
5-9 ans	4	8	12	2	50	3	37,5	5	41,66
10-14 ans	8	24	32	7	87,5	21	87,5	28	87,5
15-29 ans	14	15	29	13	92,85	13	86,66	26	89,65
30-49 ans	11	14	25	8	72,72	13	92,85	21	84
50 ans et +	15	21	36	14	93,33	16	76,19	30	83,33
Total	54	83	137	45	83,33	67	80,72	112	81,75

112 personnes sont porteurs de microfilaires d'onchocerca-volvulus à Bela ; sur 137 examinées , soit un indice microfilarien (IM) de 81,75 % (112/137) dont : 45 personnes du sexe masculin soit un indice microfilarien (IM) chez les hommes égal à 80,72 d'une part.

D'autre part, nous avons pu mettre en évidence la présence des microfilaires d'onchocerca-volvulus chez 35 enfants de moins de 15 ans sur un total de 47 enfants du même âge examinés soit un indice microfilarien de 74,46 % chez les enfants.

../..

Et parmi les 9o adultes âgés de 15 à 50 ans examinés, il y a eu 77 qui ont des microfilaires d'onchocerca-volvulus soit un indice microfilarien (IM) chez les adultes de 85,55 %.

Notre plus jeune enfant porteur de microfilaires d'onchocerques à Bela est âgé de 3 ans.

En outre, nous avons remarqué qu'à Bela sur 33 sujets porteurs de nodules onchocerquiens, il y a eu 2 à qui nous n'avons pas pu mettre en évidence les microfilaires. D'où l'indice clinico-parasitologique à Bela égal à 83,21 % (114/137).

CHARGES PARASITAIRES

Tableau N° 42 Distribution des charges parasitaires en fonction de l'âge et du sexe à Bela (observations de la période du 18 au 26 NOvembre 1985).

Tranches d'âges	Sexe Masculin				Sexe Féminin				Ens.=Masc. + Fém.			
	Exa.	Cas +	CPT	CPM	Exa.	Cas +	CPT	CPM	Exa.	Cas +	CPT	CPM
0-4 ans	2	1	1	1	1	1	3	3	3	2	4	2
5-9 ans	4	2	56	28	8	3	376	125,33	12	5	432	86,4
10-14 ans	8	7	351	50,14	24	21	1184	56,38	32	28	1535	54,82
15-29 ans	14	13	1957	150,53	15	13	1893	145,61	29	26	3850	148,07
30-49 ans	11	8	1497	187,12	14	13	2099	161,46	25	21	3596	171,23
50 ans et +	15	14	2229	159,21	21	16	2313	144,43	36	36	4542	151,4
TOTAL	54	45	6091	135,35	83	67	7868	117,43	137	112	13959	124,63

Nous remarquons sur le tableau des charges parasitaires ci-dessus que la charge parasitaire moyenne pour l'ensemble des porteurs de microfilaires dans le village est de 124,63. Et que la charge parasitaire moyenne des hommes est plus élevée (135,35) que celle des femmes qui n'est que de 117,43.

CPT = Charge parasitaire totale

CPM = Charge parasitaire moyenne.

La tranche d'âges qui héberge plus beaucoup de microfilaires tant chez les hommes que chez les femmes est celle de 30 - 49 ans avec une charge microfilarienne moyenne de 187,12 chez les hommes, et 161,46 chez les femmes.

4-2-5 FOYER DE MANDOMBE

Le village Mandombé est situé à environ 198 kilomètres de Brazzaville (sur piste) et à 30 kilomètres environ de Boko Poste. Il est bâti au bord du fleuve Congo. La distance qui le sépare du fleuve est d'environ 3 kilomètres. Il est en outre distant de Bela d'environ 23 kilomètres.

STRUCTURE DE LA POPULATION ETUDIEE

Mandombé qui a été notre dernier centre de travail pour notre enquête a eu une structure toute particulière. Car, faute de temps, nous n'avons pas pu examiner les enfants dans ce village.

En effet, Mandombé compte une école avec un effectif d'au moins 320 élèves que nous n'avons pas pu visiter, compte tenu du temps qui nous était imparti. Car, le véhicule qui nous transportait durant la mission était attendu à Brazzaville pour une autre mission.

Aussi, les quelques enfants que nous avons pu voir dans ce village sont ceux qui ne vont pas à l'école ou qui n'étaient pas à l'école ce jour-là. D'où nous n'avons pu visiter que 14 enfants de moins de 15 ans soit 6,76 % de la population totale visitée à Mandombé contre 193 adultes âgés de 15 à 50 ans et plus soit 93,23 %.
Au total donc 207 sujets ont été visités à Mandombé dont 62 de sexe masculin, soit 29,95 % de la population totale visitée . Et 145 de sexe féminin, soit 70,04 % (voir tableaux N°43 et 44).

Tableau N° 43 : Répartition de la population en fonction du sexe à Mandombé (observations de la période du 18 au 26 Novembre 1985).

Sujets examinés	Hommes	%	Femmes	%
207	62	29,95	145	70,04

Tableau N° 44 Répartition de la population en fonction de l'âge et du sexe à Mandombé (observations de la période du 18 au 26 Novembre 1985)

Tranches d'âges	Hommes	Femmes	Total hommes+ Femmes	%
Enfants 0 - 14 ans	2	12	14	6,76
Adultes 15 - 50 ans	60	113	193	93,23
Total	62	145	207	100

RESULTATS CLINIQUES

A Mandombé, sur 207 sujets examinés : 121 présentent au moins un signe clinique majeur de l'onchocercose (nodules, signes oculaires, lésions cutanées). Soit un pourcentage de 58,45 %Le tableau N°45 ci-dessous résume les résultats obtenus.

Tableau N°45 Répartition des signes cliniques observés pendant la période du 18 au 26 Novembre 1985) en fonction de l'âge à Mandombé.

Tranches d'âges	Examinés	Porteurs de nodules	Porteurs des lésions cutanées				Atteintes oculaires	
			Dépigmentation tibiale	Atrophie cutanée	Gale filarienne	Pachydermie	Perte de vue grave	Cécité
0 - 4 ans	2	0	0	0	0	0	0	0
5 - 9 ans	4	0	0	0	0	0	0	0
10 -.14ans	8	0	0	0	0	0	0	0
15 - 29 ans	54	9	0	0	0	0	1	0
30 - 49 ans	65	31	3	0	0	0	6	1
50 ans et plus	74	40	12	7	0	3	15	2
Total	207	80	15	7	0	3	22	3

Sur 14 enfants examinés, aucun ne présente même un signe clinique majeur de l'onchocercose.

Nous avons remarqué par contre que le nombre de porteurs de nodules croit selon l'âge. Mais les sujets les moins âgés, c'est à dire ceux âgés de 0-14 ans : aucun d'entre eux n'est porteur de nodules ; et à partir de 15 ans, on voit une croissance véritable du nombre des porteurs de nodules échelonnés respectivement de 0-9 -31 jusqu'à 40 porteurs pour les tranches d'âges des sujets plus âgés (exemple : 50 ans et plus = 40 porteurs).

La gale filarienne est inexistante à Mandombé parmi les sujets examinés. Quant à la topographie des nodules, le tableau 46 ci-après nous montre leur répartition selon le sexe.

Tableau N° 46 : Répartition des nodules en fonction du sexe et de la topographie à Mandombé (observations de la période du 18 au 26 Novembre 1985)

Localisations des nodules / Sexe des porteurs	Nodules Céphaliques	Nodules Thoraciques	Nodules pelviens	Total	%
Porteurs masculins (Nbre)	2	0	23	25	40,32
Porteurs féminins (Nbre)	0	0	55	55	37,93
Total	2	0	78	80	38,64

Au total, 80 personnes sont porteurs de nodules onchocerquiens sur 207 personnes examinées, soit un indice kystique (IK) de 38,64 : dont 25 personnes du sexe masculin, soit un indice kystique (IK) de 40,32 % chez les hommes (25/62).
ET 55 personnes du sexe féminin soit un indice kystique (IK) chez les femmes de 37,93 % (55/145).

Comme nous n'avons pas examiné assez d'enfants dans ce village, nous n'avons donc pas considéré le nombre d'enfants visités. L'échantillon des enfants visités étant trop faible.

../..

Ici, nous ne considérons donc que les indices chez les adultes qui sont respectivement de 40,32 % chez les hommes et 37,93 % chez les femmes.(Indice kystique.)

Tous les nodules décélés à Mandombé sont localisés au niveau de la ceinture pelvienne sauf chez 2 personnes à qui ils sont de localisation céphalique. Ainsi donc : 97,5 % des nodules sont de topographie basse (pelvienne) à Mandombé et 2,5 % seulement ont une topographie (ou localisation) céphalique. Aucun nodule n'a été dépisté au niveau thoracique à Mandombé.

Quant aux atteintes oculaires, nous avons pu déceler 25 personnes avec perte de vue grave dont 3 avec cécité totale parmi les 3 aveugles il y a 1 homme et 2 femmes. ET 10 personnes avec une baisse d'acuité visuelle . Le taux de cécité à Mandombé est donc de 1,44 % (3/207).

RESULTATS PARASITOLOGIQUES

Le tableau N°47 ci-après résume la répartition de la parasitose onchocerquienne à Mandombé.

Tableau N°47 Prévalence de l'onchocercose en fonction de l'âge et du sexe à Mandombé (observations de la période du 18 au 26 Novembre 1985)

Tranches d'âges	Sujets examinés			Nombre d'onchocerquiens dépsités parasitologiquement					
	Hommes	Femmes	Total	Hommes	IM %	Femmes	IM %	Total	IM %
0 - 4 ans	0	2	2	0	0	1	50	1	50
5 - 9 ans	1	3	4	0	0	2	66,66	2	50
10 - 14 ans	1	7	8	0	0	5	71,42	5	62,5
15 - 29 ans	8	46	54	7	87,5	43	93,47	50	92,59
30 - 49 ans	16	49	65	15	93,75	47	95,91	62	95,38
50 et +	36	38	74	34	94,44	35	92,10	69	93,24
Total	62	145	207	56	90,32	133	91,72	189	91,30

189 personnes sont porteurs de microfilaires d'onchocerca-volvulus à Mandombé sur 207 personnes examinées ; soit un indice microfilarien (IM) de : 91,30 % (189/207)

56 sujets de sexe masculin sont porteurs de microfilaires d'onchocerques ; soit un indice microfilarien(IM) de 90,32 % chez les hommes. (56/62).

Contre 133 sujets de sexe féminin porteurs de microfilaires d'onchocerques; soit un indice microfilarien (IM) de 91,72 % (133/145) presque la totalité des sujets examinés étant des adultes âgés de 15-50 ons et plus, soit 193 adultes : l'indice microfilarien chez les adultes est donc de 93,78 % à Mandombé.

En outre, parmi les 80 personnes porteurs des nodules onchocerquiens, nous avons pu mettre en évidence les microfilaires d'onchocerques chez 75 d'entre eux.

Par contre les 5 autres personnes ne sont pas porteurs de microfilaires.
D'où l'indice clinico-parasitologique (ICP) à Mandombé de 93,71 % (194/207).

CHARGES PARASITAIRES

Tableau N°48 : Distribution des charges parasitaires en fonction de l'âge et du sexe à Mandombé (observations de la période du 18 au 26 Novembre 1985).

(voir page suivante)

Tableau N° 48 Distribution des charges parasitaires en fonction de l'âge et du sexe à Mandombé (observations de la période du 18 au 26 Novembre 1985)

Tranches d'âges	Sexe Masculin				Sexe Féminin				Ensemble= Masc. + Féminin			
	Examinés	Cas +	CPT	CPM	Exam.	Cas +	CPT	CPM	EXam.	Cas +	CPT	CPM
0-4 ans	0	0	0	0	2	1	11,5	11,5	2	1	11,5	11,5
5 - 9 ANS	1	0	0	0	3	2	31	15,5	4	2	31	15,5
10-14 ans	1	0	0	0	7	5	170,5	34,1	8	5	170,5	34,1
15-29 ans	8	7	267	38,14	46	43	2503	58,20	54	50	2770	55,4
30-49 ans	16	15	1012	67,46	49	47	4076	86,72	65	62	5088	82,06
50 et + ans	36	34	273,5	80,45	38	35	1660,5	47,44	74	69	4396	63,71
Total	62	56	4014,5	71,68	145	133	8452,5	63,55	207	189	12467	65,96

Le tableau ci-dessus nous résume l'intensité de l'infestation des sujets par Onchocerca-volvulus à Mandombé.
Nous remarquons en effet qu'à Mandombé la charge parasitaire moyenne pour l'ensemble de la population examinée et porteur des microfilaires est de 65,96.

Et que comme dans tous les autres villages visités : la charge parasitaire moyenne des hommes est plus élevée 71,68 que celle des femmes 63,55 ; 50 ans et plus est la tranche d'âges qui héberge plus de microfilaires chez les hommes. Par contre chez les femmes c'est la tranche d'âges de 30-49 ans qui héberge beaucoup plus de microfilaires.

NB : CPT : Charge parasitaire totale
CPM : Charge parasitaire moyenne
Exam : Examinés

CHAPITRE V

COMMENTAIRES ET DISCUSSION

CHAPITRE V

1 COMMENTAIRES ET DISCUSSIONS

La méthode dite de "NELSON" que nous avons utilisé pour le milieu d'incubation et temps de lecture de nos BCE (Biopsie cutanée exsangue) est bonne dans la mesure où l'équipe désignée pour effectuer l'enquête est réduite (2 ou 3 personnes) comme celle que nous avons constituée pour cette enquête, car elle permet pendant que 3 ou 4 plaques sont remplies et bien recouvertes du parafilm de continuer le travail.

Par contre si l'équipe est constituée de plusieurs personnes, cette méthode devient une perte de temps dans la mesure où il faut à tout moment reprendre la même solution dans les plaques de microTitrations pour la remettre ensuite sur la lame puis examiner au microscope. Dans ce cas il serait souhaitable d'utiliser la méthode dite "de PICQ" (59,60,61) qui consiste à placer directement sur une lame porte - objet et dans une goutte d'eau physiologique ou eau distillée les biopsies et la numération est faite 30 minutes après :

Les données fournies par les examens cliniques et parasitologiques nous permettent de constater plusieurs aspects principaux dans l'Ensemble de l'enquête qui sont :

5-1 Premier Aspect : Sur le Plan Clinique

Sur le plan clinique : nous avons constaté que dans l'ensemble des 5 villages visités : exceptés les nodules onchocerquiens et le prurit que l'on rencontre presque dans toutes les tranches d'âges : les autres manifestations cliniques sont inexistantes ou presque chez les jeunes enfants dans la tranche d'âges de 0 - 14 ans.

../..

5-1-1 SYNDROME CUTANE

Les manifestations cutanées que nous avons relevées vont donc du simple prurit (très fréquent) à la dépigmentation tibale. Le Prurit est fréquemment rencontré dans la population tant aux porteurs des microfilaires qu'aux non porteurs, car nous avons totalisé pour l'ensemble de l'enquête 357 personnes porteurs de microfilaires d'onchocerques qui se plaignent de prurit soit un pourcentage de 41,85 % (357/853) parmi les porteurs des microfilaires d'onchocerques. Et 52 autres non porteurs de microfilaires, cette fois se plaignent de prurit , soit un pourcentage de 20,55 % (52/253) pour les non porteurs de microfilaires.

Au total, 149 personnes sont porteurs des lésions cutanées sur 1106 examinés; soit un pourcentage de 13,47 % (149/1106) réparties comme suit :

- Dépigmentation tibiale	= 6,05 % (67/1106)
- Atrophie cutanée	= 5,15 % (57/1106)
- Gale filarienne	= 0,36 % (4/1106)
- Pachydermie	= 0,81 % (9/1106)
- Dépigmentation + pachydermie	= 1,08 % (12/1106)

Gale filarienne + Pachydermie + Dépigmentation = 0,09 % (1/1106) (voir graphique N°6 en Annexe 10)

Dans presque tous les villages visités : la lésion cutanée la plus fréquemment rencontrée est la dépigmentation tibiale. Nous avons en outre remarqué un très faible pourcentage de porteurs de la gale filarienne : 0,36 % (4/1106)
Soit 4 sujets porteurs de la gale filarienne sur 1106 personnes examinées.

Il est à noter que les 4 sujets porteurs de la gale filarienne ont tous une BCE (Biopsie cutanée exsangue) positive. Donc tous porteurs de microfilaires d'onchocerques, contrairement à l'hypothèse selon laquelle les porteurs de gale filarienne sont rarement SNIP positif d'après une constatation faite par J. LEBRAS. B. BOUCHITE. M.LAMIZANA et J. BRENGES (1976) dans le foyer de Touboro au Cameroun.

Par ailleurs, nous avons constaté que la dépigmentation tibiale, l'atrophie cutanée et la pachydermie restent l'apanage du sujet âgé, car elles sont d'autant plus fréquemment rencontrées que les sujets sont plus âgés. Aucun sujet de moins de 15 ans n'en est atteint. Cette même constatation a été faite par A. YEBAKIMA à Bangou-Louholo en 1978.

Nous signalerons en passant qu'aucun cas d'éléphantiasis n'a été observé tout au long de notre enquête.

5-1-2 **SYNDROME KYSTIQUE**

Nous avons vu plus loin qu'au total pour l'ensemble de l'enquête, 231 sujets sont porteurs de nodules onchocerquiens, soit un indice kystique (IK) global de 20,88 % dont 85 sujets du sexe masculin. L'indice kystique chez les hommes est donc de 18,08 % contre 146 sujets du sexe féminin, d'où l'indice kystique chez les femmes égal à 22,95 %.

13 enfants seulement (âgés de 0-14 ans) sont porteurs de nodules sur un total de 464 enfants examinés. D'où l'indice kystique (IK) chez les enfants égal à 2,80 % (13/464).

Ces chiffres avoisinent aussi bien ceux trouvés par J.LEBRAS et Coll 1976 dans le foyer de touboro au Cameroun.

Que ceux trouvés par A. YEBAKIMA (1978) à Bangou-Louholo, région du Pool ; A. YEBAKIMA et Coll 1982 dans la vallée du Djoué région du Pool ; A. YEBAKIMA et Coll (1982) à Kinsassa (région du Pool) respectivement de 21,27 % ; 20 ; 35 % ; 27,4 %.

Le nombre de porteurs de nodules varie d'un foyer à l'autre. C'est ainsi que la proportion globale pour toute la région varie de 20 à 50 sauf à Mandombé où cette proportion a atteint un maximum de 80 porteurs de nodules sur 207 sujets visités.

Nous avons également noté une augmentation progressive du nombre de porteurs des nodules en fonction de l'âge, atteignant son maximum dans la tranche d'âges de 50 ans et plus (voir tableau N°51 en Annexe.)

../..

Nos observations ont montré en outre que les nodules ont essentiellement les mêmes localisations que celles observées partout en Afrique dans les foyers d'onchocercose. Nous avons ainsi noté que la plupart des nodules sont de topographie basse avec une prédominance à la ceinture pelvienne dans 90,90 % des cas.

La localisation thoracique a été observée dans 3,89 % des cas seulement.

Et la localisation céphalique dans 5,19 % des cas pour la zone Sud-Est de Boko (regroupant les 5 villages visités). Par contre pour la zone Sud-Ouest de Boko c'est-à-dire à Ntombo-Manianga (région du Pool) : LOUZIEMI a observé en 1985 les taux suivants; 86 % à la ceinture pelvienne et 0,7 % de nodules céphaliques (23).

- LABRAULET et Coll (1964) en Haute-Volta (actuel BOURKINA-FASO) cités par LOUZIEMI trouvent 76,7 % de nodules de localisation basse dont 75,5 % sur la seule ceinture pelvienne et 0,5 % seulement de nodules céphaliques. (23).

ILs évoquent la possibilité d'un lien de cause à effet entre le lieu de piqûre et la localisation des nodules. Simulium damnosum qui est le vecteur de l'onchocercose au BURKINA-FASO ainsi qu'au Congo pique le plus souvent à l'endroit situé le plus près du sol ; or les localisations basses sont les plus fréquemment exposées.

Les résultats observés dans la région du Pool comparés à ceux de BURKINA-FASO est un argument en faveur de cette hypothèse.

5-1-3 SYNDROME OCULAIRE

Nous savons que les manifestations oculaires dominent le tableau clinique de l'onchocercose dite de savane comme c'est le cas de notre zone d'étude dominée par la savane. Aussi, leur étude est de ce fait capitale, mais il va de soi qu'au niveau d'une équipe sans spécialiste ni moyens d'investigation particuliers comme celle que nous avons constituée pour notre enquête;

L'interprétation de certaines manifestations observées est parfois très délicate.

C'est pourquoi nous nous sommes attachés à relever un minimum d'observations pour ne retenir finalement que les signes les plus évidents cliniquement, les plus caractéristiques et les plus habituels.

Ainsi donc les manifestations oculaires que nous avons pu observées cliniquement par notre examen sommaire et que nous avons définies comme suit :

<u>Perte de vue grave</u> = Impossibilité pour le malade de compter les doigts d'une main à 3 mètres à la lumière du jour.
Baisse de l'acuité visuelle = Impossibilité pour le malade de les compter à 6 mètres, d'après le critère d'ANDERSON et FUGLSANG : Sans oublier la cécité, sont quelque peu discrets dans certains foyers, comme à Bangou-Louholo qui est un foyer de forêt et où le taux de cécité est de 1,8 % (A.YEBAKIMA 2e Conférence Technique de L'OCEAC 1978).

A Ntombo-Manianga, foyer de savane où le taux de cécité est $\angle$ 2 (Louziemi 1985). Mafouta-Massissia (région de Brazzaville) où le taux de cécité est de 0,97 % (A.YEBAKIMA et Coll 1978).

Mais comparativement aux résultats (taux de cécité) des foyers connus avant, certains foyers connus récemment (par notre Etude, voir carte en Annexe) ont des taux de cécité plus élevés. C'est le cas de Bela où le taux de cécité est de 3,64 % (5/137) et de Mandombé avec 1,44 % .

Les quelques signes subjectifs et fonctionnels que nous avons relevés par l'interrogatoire sont :

1°) Larmoiement chez 16 personnes
2°) Prurit chez 20 personnes
3°) Sensations de corps étranger chez 7 personnes
4°) Photophobie chez 5 personnes
5°) Hemeralopie chez 3 personnes.

../..

Aussi dans l'ensemble de la zone Sud-Est de Boko nous avons observé au total 64 personnes souffrant d'une, perte de vue grave, soit un pourcentage de 5,78 % de la population totale examinée dont 13 avec cécité prononcée, soit un taux de cécité global pour toute la zone de 1,17 % (13/1106)

Il est à noter que nous n'avons considéré comme porteur de cécité que tous ceux qui sont aveugles.

5-1-4 INCIDENCE DE L'ONCHOCERCOSE SUR LES ATTEINTES OCULAIRES

L'Incidence de l'onchocercose sur les atteintes oculaires dans la zone Sud-Est de BOko peut se démontrer par le fait que tous les 64 sujets présentant des manifestations oculaires cliniques (tableau N°53 en Annexe) portent des microfilaires d'onchocerca-volvulus d'une part.

D'autre part, nous pouvons affirmer que les atteintes oculaires observées dans la région du Pool sont bien vraisemblablement dues à l'onchocercose, car les examens ophtalmologiques effectués antérieurement dans cette région par des spécialistes nous le prouve à suffisance.

5-2 DEUXIEME ASPECT : SUR LE PLAN PARASITOLOGIQUE

Dans l'ensemble des 5 villages prospectés, nous avons eu à pratiquer 1106 BCE (biopsie cutanée exsangue) dont 853 se sont revelées positives donc avec présence des microfilaires d'onchocerca-volvulus ;soit un indice microfilarien (IM) global (pour l'ensemble de la zone) de 77,12 % (853/1106)

De tous les résultats obtenus sur le plan parasitologique tant dans l'enquête rétrospective que dans l'enquête prospective : Il ressort que dans l'ensemble des zones d'observations

(de la région du Pool), l'indice microfilarien (IM) varie selon les foyers.

Ainsi de 91,30 % à Mandombé et 81,75% à Bela ; 77 % à Mantaba ; 73,35 % à Kimpenga et 71,5 à Ntombo-Manianga : L'indicé microfilarien (IM) passe à 40 % à Bangou-Louholo et est compris entre 35,5 et 67,8 % dans le bassin du Djoué.
D'une manière spécifique, Mandombé qui est situé très proche du fleuve Congo (à 3 kilomètres du fleuve environ) et de ses rapides ainsi que Bela qui est également situé proche de la Louvoubi (à 2 km environ) et de ses rapides semblent être les foyers les plus touchés. Leurs taux d'infestation dépassent ceux des autres foyers, bien qu'étant eux aussi élevés. Cette constatation rejoint celle faite par LOUZIEMI à Lemba (Boko région du Pool) en 1985, celles de RIPPERT et Coll (1978, CHANDENIER (1983) qui confirment la notion des villages de 1ère, 2e et 3e ligne qui régit les zones d'endémicité onchocerquienne à partir d'un gîte à simulies.

Pour l'ensemble des 5 villages de la zone Sud-Est de Boko que nous avons prospectés ; le nombre total de porteurs des microfilaires est de 853 sur 1106 sujets examinés soit un indice microfilarien global de 77,12 % ; dont 343 sujets de sexe masculin, soit un indice microfilarien (IM) de 72,82 % chez les hommes (343/471) contre 510 sujets de sexe féminin, soit un indice microfilarien (IM) de 80,31 % (510/035) chez les femmes. (voir tableau N° 50 en annexe).
Et 277 enfants âgés de 0-14 ans sont porteurs des microfilaires sur un total de 464 enfants du même âge visités soit un indice microfilarien de 59,69 % (277/464) chez les enfants.

../..

Contre 576 adultes âgés de 15 à 50 ans et plus soit un indice microfilarien de 89,71 % (576/642) chez les adultes. Ces résultats avoisinent ceux trouvés par J. LEBRAS ; B. BOUCHITE ; M.LAMIZANA et J. BRENGUES en 1976 dans le foyer de TOUBORO au Cameroun quant à la considération selon l'âge. Mais très différents de la plupart des résultats des enquêtes effectuées tant dans notre pays qu'ailleurs en Afrique dans les foyers d'onchocercose, quant à la considération selon le sexe. (Nous le verrons plus loin dans l'influence du sexe dans l'onchocercose).

Les courbes tracées à partir des données sur l'indice microfilarien (graphique N°1) semblent avoir une même allure et montre une légère pente douce ascendante et progressive à partir de la tranche d'âges de 10-14 ans, atteignant leur maximum correspondant au taux maximal d'infestation à partir de la tranche d'âges de 30-49 ans jusqu'au delà. (voir annexe 2)

1-2-1 **CHARGE PARASITAIRE**

Le calcul de la charge parasitaire moyenne est une étude qui nous a permis de démontrer objectivement l'effet cumulatif de l'onchocercose tant chez l'individu qu'à la collectivité dans la zone de notre étude.

Nous avons donc constaté que la charge parasitaire moyenne varie également comme l'indice microfilarien selon les foyers, selon le sexe, et selon l'âge.. Elle augmente (la charge parasitaire moyenne) également au fil des années.

C'est ainsi que: la moyenne de la charge parasitaire à Ntombo-Manianga (zone Sud-Ouest de Boko) est de 24 microfilaires. (LOUZIEMI 1985). Par contre, nous avons trouvé dans la zone Sud-Est de Boko des personnes avec 200 à 250 microfilaires par BCE (biopsie cutanée exsangue).

La charge parasitaire moyenne pour cette zone est de 42,77 (environ 43 microfilaires). (voir tableau N°56 en annexe)

Le tableau N°56 enannexe nous montre donc la variation de la charge parasitaire moyenne dans l'ensemble des 5 villages visités de la zone Sud-Est de Boko, et nous constatons que la charge parasitaire moyenne globale est de 42,77 pour l'"ensemble de deux sexes avec pour les hommes une charge parasitaire moyenne de 45,90 contre 40,67 pour les femmes.

La charge parasitaire moyenne pour l'ensemble des enfants de 0-14 ans porteurs de microfilaires est de 16,15. Et celle des adultes de 15 à 50 ans et plus est de 55,57. Par ailleurs nous avons noté que la tranche d'âge de 30-49 ans héberge beaucoup plus de microfilaires dans l'ensemble. Les courbes tracées à partir de ces données (voir Fig. N°5 en annexe) ont une allure globale identique avec cependant la différence que la courbe des charges parasitaires moyennes des hommes a une amplitude plus importante que celle des femmes.

Ces courbes qui montrent une ascension brutale à partir de la tranche d'âges de 15-29 ans, connaissent une baisse progressive dans la tranche d'âge de 50 ans et plus.

Nous pensons que cette baisse de la charge parasitaire observée dès l'âge de 50 ans concorde probablement avec l'idée avancée par certains auteurs qui énoncent qu'il s'établit un équilibre chez l'homme à partir d'un certain âge par disparition (mort) des vers filaires) ayant atteint la limite de leur longévité.

En outre si la montée des courbes de prévalence commence à partir de la tranche d'âge de 10-14 ans pour les deux sexes : les courbes de variations des charges parasitaires moyennes elles commencent une montée lente et progressive dès le jeune âge à partir de la tranche d'âge de 5-9 ans puis opèrent une montée brutale à partir de la tranche d'âges de 15 à 29 ans atteignant leur pic dans la tranche d'âges de 30-49 ans, ce qui correspond pour les deux sexes au groupe d'âge le plus infesté. (Voir onnexes 7 et 8)

.../..

Cette variation qui s'observe à l'intérieur d'une même zone voir d'un même village rejoint certainement l'hypothèse avancée par plusieurs auteurs sur l'existence d'une corrélation entre le taux d'infestation onchocerquienne, l'âge et la durée de séjour dans une région d'endémie onchocerquienne.

Troisième Aspect - 5-3 : RELATION ENTRE LA PARASITOSE ONCHOCERQUIENNE ET LES MANIFESTATIONS CLINIQUES .

5-3-1 Relation avec le syndrome kystique

Dans la relation entre la parasitose onchocerquienne et le syndrome kystique, nous avons remarqué qu'il existe une légère discordance entre les résultats parasitologiques et cliniques (voir tableau N°52 en annexe).
En effet, les porteurs des microfilaires n'ont pas toujours de kystes et, inversement, les porteurs de kystes ne sont pas toujours microfilariens ou microfilaires (+)

C'est ainsi que sur 231 porteurs de nodules onchocerquiens, nous avons pu mettre en évidence les microfilaires d'onchocerca-volvulus chez 216 personnes soit un pourcentage de porteurs simultanés des microfilaires et de kystes de 93,50 % (216/231).
15 personnes seulement ont un SNIP négatif parmi les 231 porteurs de nodules soit un pourcentage de 6,49 % (15/231).
Donc les 15 personnes sont uniquement porteurs de kystes onchocerquiens.

Par contre sur 853 onchocerquiens dépistés parasitologiquement 637 sont porteurs uniquement de microfilaires d'onchocerca-volvulussans kystes onchocerquiens (donc 637 avec SNIP (+) et kystes (-); soit un pourcentage de 74,67 % (637/853).

Ces résultats peuvent ainsi donc formuler un argument en faveur de l'hypothèse avancée par J LE BRAS (1976 selon laquelle " il est normal de rencontrer des sujets avec SNIP positif et kyste négatif pour cause d'omission de kyste à la

../_.

palpation par exemple. Il est tout aussi normal de rencontrer l'inverse".
LAGRAULET, cité par LARIVIERE ET DIALLO (1967) admet que "60 % des nodules sont en voie de dégénérescence au dessus de 45 ans (mort naturelle des filaires).
Et un SNIP de la crête iliaque peut par ailleurs être négatif chez un porteur de kyste céphalique". Ceci justifie également nos résultats qui montrent que sur 12 SNIPS ou BCE pratiqués chez les porteurs de nodules céphaliques : 9 seulement sont positifs et les 3 autres négatifs.

5-3-2 RELATION AVEC LE SYNDROME CUTANE

Dans notre zone d'étude, le syndrome cutané semble beaucoup lié à la parasitose onchocerquienne, car sur 149 porteurs des lésions cutanées 142 ont une BCE positive; soit un pourcentage de 95,30 % (142/149). ET 7 seulement ont une BCE négative soit un pourcentage de 4,69 % (7/149).

5-3-3 RELATION AVEC LE SYNDROME OCULAIRE

Le syndrome oculaire semble le plus lié à la parasitose onchocerquienne dans notre zone d'étude. Car nous l'avons dit plus haut : les 64 sujets présentant des manifestations oculaires observées dans l'ensemble des 5 villages visités sont tous porteurs des microfilaires d'onchocerca-volvulus . Ce qui explique d'ailleurs l'originalité étiologique de ces manifestations.

Enfin, l'étude des correlations existant entre la parasitose onchocerquienne et la clinique onchocerquienne nous a permis d'établir les courbes de l'indice clinico-parasitologique (ICP) dans la limite des valeurs observées. Ceci nous a permi d'estimer le nombre total et le pourcentage des onchocerquiens d'après le nombre de porteurs des microfilaires et/ou de kystes. C'est ainsi que nous avons trouvé pour l'ensemble de la zone un indice clinico-parasitologique de 78,48 % (868/1106) (voir tableau N°52 en annexe). (annexes 5 et 6)

../..

Quatrième Aspect INFLUENCE DU SEXE DANS L'ONCHOCERCOSE

Les résultats de notre enquête prospective nous ont revelé un fait assez curieux et contraire aux autres enquêtes menées tant dans notre région d'étude ou dans notre pays qu'ailleurs en Afrique.
"En règle générale, l'incidence de l'onchocercose est sensiblement plus élevée chez les hommes que chez les femmes;" LE BRAS ; B. BOUCHITE et Coll 1976).

Mais à une exception près, dans presque tous les villages visités sauf 1 (Bela) : nous avons constaté que les femmes sont les plus touchées par l'endémie onchocerquienne que les hommes sauf à Bela. Comme le démontre le tableau comparant les paramètres épidémiologiques ci-après.

Tableau N° 49 : Paramètres épidémiologiques en fonction du sexe dans l'ensemble des 5 villages (Foota, Mantaba-Kimpenga Bela-Mandombé ; (observations de la période du 18 au 26 Novembre 1985).

Paramètres	Chez les hommes	Chez les femmes
Indice microfilarien (IM)	72,82 % (343/471)	80,31 % (510/635)
Indice kystique (IK)	18,04 % (85/471)	22,99 % (146/635)
Perte de vue grave + Cécité	4,03 % (19/471)	7,24 (46/635)

Pour les indices microfilarien, kystique et les pourcentages des atteintes oculaires : (voir tableaux N°23 page 69 N°29 page 75 N°35 page 82 ; N°4[illegible] page 93; N° 47 page 94 pour chaque village. Puis N°50 et 53 en annexe.

Aussi la comparaison des résultats de deux sexes analysés Statistiquement donne les résultats suivants :

../..

Pour l'indice microfilarien :

Avec le test de l'Ecart-réduit : Nous avons utilisé la formule suivante :

$$\varepsilon = \frac{P_M - P_F}{\sqrt{\frac{Pq}{N_M} + \frac{Pq}{N_F}}}$$

avec PF = Prévalence féminine = 0,80

PM = Prévalence Masculine = 0,72

q = Complémant à 1 de 0,72 = 0,28

q = Complément à 1 de 0,80 = 0,20

N_F = Effectif des sujets féminins = 635

N_M = Effectif des sujets masculins = 471

ε = Ecart-réduit

$$|\varepsilon| = \frac{0,72 - 0,80}{\sqrt{\frac{0,72 \times 0,28}{471} + \frac{0,80 \times 0,20}{635}}} = 4$$

$|\varepsilon|$ = 4 > 1,960 (pratiquement 2) la différence est statistiquement significative au seuil de 5 %

avec le test de X^2 'sur la prévalence des porteurs de microfilaires

X^2 = 8,88 ddl = 1

La différence est statistiquement significative au seuil de 5%.

Pour l'indice kystique : Test de l'Ecart-réduit

toujours avec la même fomule :

$$|\varepsilon| = \frac{P_M - P_F}{\sqrt{\frac{P_q}{N_M} + \frac{P_q}{N_F}}}$$

avec P_M = Prévalence masculine = 0,18

P_F = Prévalence féminine = 0,22

q = Complément à 1 de 0,18 = 0,82

q = Complément à 1 de 0,22 = 0,78

N_M = Effectif masculin = 471

N_F = Effectif féminin = 635

$$|\varepsilon| = \frac{0,18 - 0,22}{\sqrt{\frac{0,18 \times 0,82}{471} + \frac{0,22 \times 0,78}{635}}} = 2$$

La différence est sensiblement significative au seuil de 5 %.

../..

Quant aux charges parasitaires : la comparaison des résultats des charges parasitaires moyennes entre les hommes et les femmes nous a permis de vérifier l'hypothèse avancée par PICQ J.J et ROUX J. (1974) dans : "Instantanés sur l'Endémie Onchocerquienne en Afrique de l'Ouest".
En effet : ces auteurs disent : " Il existe de façon pratiquement constante, une différence entre les sexes dans la gravité de l'Infection. Ces différences apparaissent dans les prévalences des lésions oculaires et des cécités onchocerquiennes et sont objectivées par les charges microfilariennes moyennes plus faibles chez les hommes."

Nous, de notre part, nos observations nous ont prouvé le contraire là également. Car, pour notre enquête prospective dans la zone Sud-Est de Boko : nous avons remarqué que les charges parasitaires moyennes sont plus faibles chez les femmes avec 40,67 contre 45,90 pour les hommes. (voir tableau N°56 en annexe).

L'analyse statistique des charges parasitaires moyennes par le test de l'écart-réduit nous donne les résultats suivants :

$$\varepsilon = \frac{m_M - m_F}{\sqrt{\frac{S_M^2}{N_M} + \frac{S_F^2}{N_F}}}$$

avec : m_M = moyenne des charges parasitaires pour les masculins = 45,90

m_F = moyenne des charges parasitaires pour les femmes = 40,66

$$\varepsilon = \frac{45,90 - 40,66}{\sqrt{\frac{(25,36)^2}{343} + \frac{(16,91)^2}{510}}} = 3,38$$

$S_M = \sqrt{\frac{\Sigma ni(xi-m)^2}{n-1}}$ pour les masculins = 25,36

$S_F = \sqrt{\frac{\Sigma ni(xi-m)^2}{n-1}}$ pour les féminins = 16,91

N_m = Effectif des sujets masculins = 3

N_F = Effectif des sujets féminins

Nous avons : ε = 3,38 > 2, la différence est statistiquement significative au seuil de 5 %

ε = écart réduit

../..

Toutes ces observations nous incitent à penser que la différence sur l'onchocercose qui existe entre les deux sexes dans la zone Sud-Est de Boko est due probablement soit à une receptivité meilleure de l'agent pathogène de la part des sujets du sexe féminin : soit à une exposition plus grande des sujets du sexe féminin. Ce qui laisse croire qu'il existe bien une exposition inégale aux piqûres du vecteur entre les hommes et les femmes. Ces dernières fréquentant beaucoup plus fréquemment les plantations que les hommes ; celles-ci (les plantations) étant placées le plus souvent aux abords immédiats des cours d'eau (lieu des gîtes des simulies), et qui conditionne la fréquence des contacts homme-vecteur, qui peuvent être plus importants chez les femmes . que chez les hommes.

Mais il est aussi possible que le district de Boko (particulièrement en sa partie Sud-Est), entretienne ou développe vraisemblablement , une souche d'onchocercose particulière.. , très différente .

Aussi sommes-nous tenter d'admettre à la suite de nombreux auteurs (LE BERRE (1966) ; DUKE (1968) et (1975) BELLEC (1974) : PHILIPPON (1977) , que l'épidémiologie de l'onchocercose est complexe ; c'est de l'interaction des facteurs entomologiques (dispersion, longévité, nature cytotaxonomique, pouvoir vectoriel) des facteurs parasitaires (pathogénicité différente des souches) et des facteurs humains (activités sociales, différence d'attrativité, réactions immunologiques), que dépend le faciès épidémiologique d'un foyer.

: Cinquième Aspect 5-5 CONSIDERATIONS GENERALES DE L'ENDEMIE ONCHOCERQUIENNE DANS LA REGION DU POOL

5-5-1 Comparaison des Foyers Connus recemments
Comparaison entre le foyer de Foota et celui de Manataba

Si nous comparons le foyer de Foota à celui de Mantaba tous deux distants entre eux de 40 kilomètres environ et l'un étant situé à environ 3 kilomètres du fleuve (celui de Foota , l'autre à environ 1 kilomètre du grand cours d'eau la Nkissi (pour Mantaba).
Nous constatons que la prévalence totale à Mantaba 78,16 % est plus élevée qu'à Foota 69,36 %
$X^2 = 4,19$
pour un ddl = 1
La différence est significative.
Les enfants de 0-14 ans sont plus infestés à Mantaba 58,82% (40/68) qu'à Foota 48,25 % (69/143).
Par contre chez les adultes des deux villages, la prévalence est pratiquement la même à Foota 89,36 % (126/141) qu'à Mantaba 88,67 % (94/106).

5-5-2 Comparaison entre le foyer de Kimpenga et celui de Bela

Pour le foyer de Kimpenga avec celui de Bela distants l'un de l'autre d'environ (30 km et Kimpenga pour sa part distant du fleuve Congo (lieu probable de contact homme-vecteur) de 7 kilomètres et Bela distant de la Louvoubi (lieu probable de contact homme-vecteur) d'environ 2 kilomètres.
Nous constatons que la prévalenca totale à Bela qui est de 83,21 % (114/137) est légèrement plus élevée qu'à Kimpenga 75 % (228/304)

../..

$\chi^2 = 3,64$
pour un ddl = 1
la différence n'est pas significative

La prévalence chez les enfants de 0-14 ans est plus élevée à Bela 74,46 % qu'à Kimpenga 65,10 %
Par contre les adultes ont sensiblement la même prévalence tant à Bela 85,55 % qu'à Kimpenga 87,5 %.

5-5-3 Comparaison entre le foyer de Foota et celui de Mandombé

Et si nous comparons le foyer de Foota avec celui de Mandombé qui sont à peu près à égale distance du fleuve Congo (lieu probable de contact homme-vecteur) ; mais distant l'un de l'autre d'environ 70 kilomètres : nous constatons là également qu'à Mandombé la prévalence totale qui est de 93,71 % (194/207) est plus élevée qu'à Foota où elle est de 69,36 % (197/284).

$\chi^2 = 43,75$
pour un ddl = 1
la différence est statistiquement significative.

Ici nous ne pouvons pas parler de la différence selon l'âge d'autant plus que l'échantillon des enfants à Mandombé n'est pas viable.

5-5-4 Comparaison entre le foyer de Bela et celui de Mandombé

La comparaison faite entre le foyer de Bela situé presque au bord d'un affluent du Congo (la Louvoubi) à environ 2 kilomètres avec celui de Mandombé situé presque au bord du fleuve Congo à environ 3 kilomètres ; nous montre que la prévalence chez les adultes est plus élevée à Mandombé (93,78 %) qu'à Bela (85,55 %)

../..

$\chi^2 = 5,13$

pour un ddl = 1

La différence est statistiquement significative

Ici également, nous ne parlerons pas de la différence selon l'âge d'autant plus que l'échantillon des enfants à Mandombé n'est pas viable.

5-5-5 Comparaison entre le foyer de Mantaba et celui de Bela

Quant à la comparaison du foyer de Mantaba avec celui de Bela tous les deux situés aux bords de deux affluents du Congo : l'un (Mantaba) à environ 1 kilomètre du bord de la Nkissi, l'autre (Bela) à environ 2 kilomètres du bord de la Louvoubi. Nos observations montrent que la prévalence totale est légèrement plus élevée à Bela (83,21 %) qu'à Mantaba (78,16 %). .

$\chi^2 = 1,22$

pour ddl = 1 la différence n'est pas significative.

De toutes ces comparaisons, nous constatons que : plus le foyer est proche d'un cours d'eau, plus sa prévalence est grande.

5-6 Sixième Aspect

CONSIDERATIONS EPIDEMIOLOGIQUES

L'emplacement des villages et des plantations aux abords immédiats des cours d'eau entraine un contact étroit et permanent entre les habitants de ces villages et le vecteur, par les habitudes des uns et les caractères des autres. Ces habitudes humains et les caractères entomologiques du vecteur créent des conditions favorables à une transmission intense dans la plupart des villages visités.

Toutes les populations humaines visitées : que ce soit pour les enquêtes antérieures à la nôtre (voir enquête rétrospective) ou pour l'enquête prospective que nous avons effectuée, cultivent et pêchent soit sur les bords du Djoué, soit sur les bords de la Louholò, ou sur le bord du fleuve Congo.
C'est donc aux abords des cours d'eau que s'effectue le plus souvent la transmission.

Ces observations soulignent ainsi l'importance du comportement de l'homme dans l'exposition à la maladie bien que le rôle de la simulie soit prépondérant, de par sa dispersion et la recherche d'un repas sanguin : . celui de l'homme par ses activités socio-économiques (plantations aux bords des cours d'eau pêche etc), n'est pas pour le moins négligeable.

L'observation faite par A. YEBAKIMA en 1978 sur les jardiniers de Brazzaville cultivant aux bords du Djoué et du Fleuve Congo et qui se sont trouvés infestés par <u>onchocerca-volvulus</u> à cause de leurs activités professionnelles est bien démonstrative' .

IL convient toutefois de signaler que dans la région du Pool, plus particulièrement dans le district de Boko ; la cécité était depuis longtemps considérée comme étant une maladie des pêcheurs exerçant au fleuve qui, à force de trop rester longtemps dans l'eau pour poser leurs nasses (technique des plongées) perdent leur vue.
D'autres encore, croient à un mauvais sort qui leur est jetté.

Aussi, la population de cette zone ne fait aucune relation entre la maladie et les simulies. Celles-ci appelées "Bimbouni" en langue locale sont plutôt connues pour leurs piqûres prurigineuses et désagréables.

../..

Mais la démonstration que nous leur avons faite lors de notre enquête, en leur montrant les microfilaires issues de leurs fragments de peau, bouger au microscope, et nos explications sur la transmission de la maladie par les simulies les ont convaincu.

CARTE N°6

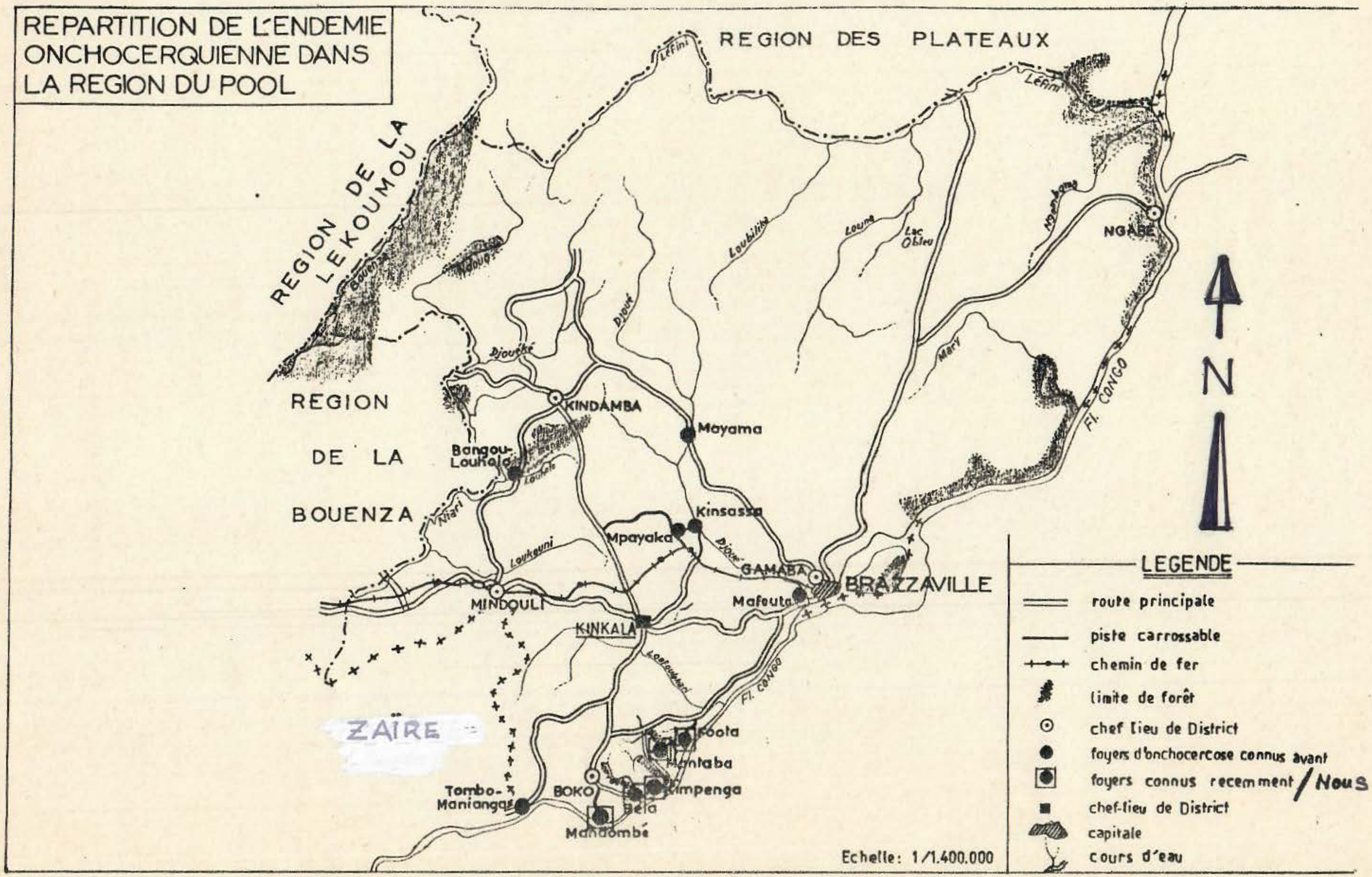

REPARTITION DE L'ENDEMIE ONCHOCERQUIENNE DANS LA REGION DU POOL
REGION DES PLATEAUX
REGION DE LA LEKOUMOU
REGION DE LA BOUENZA
ZAÏRE
Léfini
Loubilika
Louna
Lac Obleu
Djoué
Djouéké
Mari
Ngambama
Niari
Loukouni
Fl. CONGO
NGABE
KINDAMBA
Mayama
Bangou-Loukolo
Kinsassa
Mpayaka
GAMABA
BRAZZAVILLE
Mafouta
MINDOULI
KINKALA
Foota
Mantaba
Kimpenga
Bela
BOKO
Tombo-Maniangas
Mandombé
N
Echelle: 1/1.400.000
LEGENDE
route principale
piste carrossable
chemin de fer
limite de forêt
chef lieu de District
foyers d'onchocercose connus avant
foyers connus recemment / Nous
chef-lieu de District
capitale
cours d'eau

CHAPITRE VI

CONCLUSION ET SUGGESTIONS

CHAPITRE VI

CONCLUSION ET SUGGESTIONS

6-1 CONCLUSION

Au terme de ce travail qui est une mise au point sur les connaissances actuelles de l'onchocercose dans le foyer du Pool et où nous avons voulu faire ressortir à la lumière des travaux déjà réalisés par le projet National de lutte contre l'onchocercose en République Populaire du Congo et ceux que nous avons effectués recemment ; les aspects épidémio-cliniques, biologiques et enfin entomologiques de cette affection.

Et, après l'analyse des différentes enquêtes menées de 1978 à ce jour, puis la confrontation des résultats fournis par l'enquête rétrospective d'une part et l'enquête prospective d'autre part : Il ressort que l'onchocercose est répandue et sévit sous le mode endémique dans la région du Pool et admet un caractère de focalisation.
Simulium damnosum constitue le seul vecteur de la maladie.

Dans toutes les localités visitées, les atteintes cutanées habituellement décrites dans l'onchocercose ont été observées et la majorité des nodules onchocerquiens siège au niveau de la ceinture pelvienne.

Les prévalences évaluées d'après les paramètres épidémiologiques connus : indice microfilarien, indice kystique et Indice clinico-parasitologique sont élevés et atteignent en certains lieux des taux de 68,66 % ; 77,71 % ; 81 et 91,30 % respectivement à Foota ; Mantaba Ntombo-Manianga, Bela et à Mandombé.
Et les taux de cécité vont de 1,8 % à 3,40 %. C'est le cas

../..

de Bangou-Louholo (district de Kindamba) et de Bela (district de Boko.)

La région du Pool dans son ensemble abrite de nombreux foyers caractéristiques de la forme de l'onchocercose dite de savane, avec une baisse majeure de l'acuité visuelle (perte de vue grave) et même des cas de cécité complète, dans les villages où la prévalence de l'affection est élevée.

La classification des degrés d'endémicité proposée par le Comité de l'OMS (1966) est basée sur le pourcentage des sujets porteurs de microfilaires dans la population visitée.

0 à 33 % = hypoendémie

34 à 66 % = mesoendémie

plus de 66 % = hyperendémie

D'après cette classification nous pensons que l'onchocercose sevit dans la plupart des foyers du Pool à l'Etat hyperendémique; plus particulièrement dans les zones Sud-Est et Sud-Ouest du district de Boko, avec un maximum d'infestation le long du fleuve Congo.

Il apparaît en conclusion que la région du Pool qui est l'un des pôles agricoles de notre pays mériterait par son degré d'endémicité onchocerquienne et par sa démographie sans cesse croissante, une importance considérable; du point de vue de la surveillance épidémiologique de ce fléau par la multiplication des enquêtes clinico-parasitologique et entomologiques dans les zones non encore prospectées, car il y en a beaucoup. Et la finalité de ces actions sera certes de proposer une action de lutte contre ce fléau.

S U G G E S T I O N S

******
****
**
*

Sans revenir sur les recommandations habituelles préconisées dans le cadre de cette affection touchant essentiellement un bon nombre de la population :

- Il nous parait souhaitable de souligner les points suivants adaptés à la situation actuelle de l'onchocercose en République Populaire du Congo.

1°) - Disignation officielle d'un spécialiste d'ophtalmologie pour la consultation des malades dépistés par le projet onchocercose et pour certaines enquêtes concernant cette affection.

2°) - Révalorisation effective du projet de lutte contre l'onchocercose en tout point de vue.

3°) - Affectation d'un médecin responsable à plein temps au projet onchocercose.

4°) - Extension des activités de recherche sur l'onchocercose au niveau de tous les Secteurs opérationnels

5°) - Formation des entomologistes opérationnels

6°) - Poursuite et multiplication des enquêtes par le projet onchocercose surtout dans les zones non encore prospectées afin de trouver un moyen et une solution pour endiguer cette maladie. Car même si les retentissements oculaires sont de moindre importance comme le pensent certaines personnes : les divers degrés de détérioration de la vision observés affaiblissant

l'aptitude au travail dans une mesure difficile à évaluer doivent être aussi pris en considération. Puisque même une simple conjonctivite, en raison de son caractère permanent est un handicap pour ceux qui

en sont affectés.

Aussi, n'attendons pas que nous ayions des villages d'aveugles dans notre pays comme ce fut le cas dans le bassin du Sénégal autrefois (village Toukoto), pour crier au scandale après.

Nous devons dès lors que nous connaissons l'existence de la maladie dans notre pays, suivre l'exemple de ce qui se fait ailleurs : exemple dans le bassin de la Volta avec le concours des organismes comme l'OMS, l'OCEAC et les pays voisins pour mener une lutte commune afin d'endiguer cette affection qui risque à la longue de peser sur notre Economie nationale.

REFERENCES BIBLIOGRAPHIQUES

REFERENCES BIBLIOGRAPHIQUES

****
******
****
**

1 - ANONYME : Rapport Intermédiaire de l'Etude de faisabilité d'une campagne de lutte contre l'onchocercose dans la région du bassin du Logone (Cameroun) Tirés-à-part O C. E A C (1984) P. 30-32

2 - ANONYME : Colloque International sur l'onchocercose et autres filarioses humaines An. Socio-Belge Med. Trop? 1981 Vol. 61 N°2 P. 92

3 - AUGER (R) - COUDOU (A) - GIBERT (G) - GUILLOT (B)- LA COGNATTA (G) - PEYROT (B) - ZALACAIN (V) Atlas de la République Populaire du Congo. Les atlas jeune Afrique 1977 P. 62-63

4 - AMBROISE THOMAS (1975) Etude critique des procédés diagnostics parasitologiques et immunologiques des filarioses humaines An. Soc. Belge Med. Trop. V. 55 P. 497-504

5 - BRUMPT E : Les filarioses humaines en Afrique An. Soc. Biol. 1904 V. 56 P. 758-760

6 - CARME (B) - YEBAKIMA (A) - MENEZ et Coll Enquête pluridisciplinaires sur l'onchocercose dans la zone de Kibouendé résultats préliminaires. Rev. Med. du Congo 1982 P.2-19-26.

7 - <u>CAVALLO A.</u>Second rapport et mise au point sur l'avancement des travaux pour l'Etude de Faisabilité d'une campagne de lutte contre l'onchocercose dans les bassins du Logone Chari et la Benoué au 1er Septembre 1983 Tirés-à-part O C E A C (1984)

8 - <u>CAVALLO A.P.</u> Enquête sondage sur l'endémie onchocerquienne en République Centrafricaine dans le foyer de l'OUHAM_PENDE Bull de liaison et de doc. de l'OCEAC 1984 N°4 P.17.

9 - <u>CAVALLO A.</u>Diagnostic de l'onchocercose Bull de liaison et de doc. de l'OCEAC N°65 Sept-Oct 1984 P. 53-55.

10 - <u>CHANDENIER. J.</u>
Les filarioses à microfilaires dermiques au Gabon. Résultats préliminaires à propos de Sept-Enquêtes recentes thèse de Doct. en Méd. P. 45-48 (1976).

11 - <u>CLAVEAU (A.M.) - PICQ (J.J.)-ROUX (J)</u>
A propos de l'onchocercose ... suite du bilan de 4 années de collaboration entre la section parasitologie du Centre MURAZ et le Service d'ophtalmologie de l'hôpital de Bobo-Dioulasso.
XIV Conférence technique de l'OCCGE. Avril 1974 P. 52.

../..

12 - COULAUD et Coll

Onchocercose 30 millions de malades
Revue Med. Digest (1983) Vol. IX N° 6
P.6-13

13 - DOLL

Répartition géographique et Incidence de l'onchocercose dans la République du Congo (capitale Brazzaville) Epidémiologie locale:
Rapport Ronéo 1961 P. 17 (AFR/onchocercose/OMS

14 - EOZENOU PIERRE

Fiche technique concernant le projet onchocercose en République Populaire du Congo (1983) P. 1-2
(Archives DMP Brazzaville).

15 - FAIN (A) - ELSEN (P) - WERY (M) - MAERTENS (K)

(1974) Les Filarioses humaines au Mayumbe et dans les régions limitrophes (République du Zaïre). Evaluation de la densité microfilarienne.
An. Soc. belge Med. Trop. Vol. 54 N° 1 P.5-34.

16 - GENTILINI (Marc) : DUFLO (BERNARD).

Onchocercose
Med. Tropicale P. 182-188.

17 - GENTILINI Marc

Les filarioses pathogènes de l'homme : leur diagnostic et leur traitement.
An. Soc. Belge Med. Trop. Vol. 72 P. 73-76.

18 _ JOSSERAN (R) et (A) CAVALLO
Les enquêtes épidémiologiques dans l'onchocercose . Bull de liaison et de doc. de l'OCEAC N° 65 Sept-Oct 1984 P. 57-60.

19 - JOSSERAN R.
Epidémiologie de l'onchocercose
Bull de liaison et de doc. de l'OCEAC N°65 sept-Oct 1984 P. 35-42.

20 - LE BRAS (J) - BOUCHITE (B) - LAMIZANA (M) - et BRENGUES (J)
Enquête onchocercose dans le bassin Vina-pendé. Le foyer de touboro (Cameroun)
Onzième conférence technique de l'OCEAC (tirés-à-part OCEAC) P.32-37.

21 - LE BRAS (J) et TRAORE - LAMIZANA (1978)
Enquête onchocercose dans le District de Fontem et Nguiti (République Unie du Cameroun)
Rapport final 12 e Conférence Technique. de l'OCEAC (YDE)
T 1 P. 263-266.

22 - LE MAO (1985)
Enquête sondage sur l'onchocercose dans le foyer de l'OUHAM en République Centrafricaine
Tirés-à-part OCEAC) P. 2-4.

23 - LOUZIEMI J.
L'onchocercose au Congo
Connaissances actuelles - Etude du foyer de Manianga . Thèse de Doct. en Méd. (1985) INSSSA Brazzaville P. 20-25.

24 - MAMBOUENI A. J.P.
Filariose à Loa-Loa
Revue bibliographique et Enquête récentes menées en République Populaire du Congo.
Thèse de Doct. en Med. (1984) INSSSA Brazzaville P.34-38.

25 - MOREAU (J.P) - PROST (A) - PROD'HON (J)
Normalisation de la méthode des enquêtes clinico-parasitologiques sur l'onchocercose en A.O.F
An. Soc. belge Med. Trop. N°41 (1978) P.43-51.

26 - MOREAU et Coll
Essai de normalisation de la méthodelogie des enquêtes clinico-parasitologiques sur l'onchocercose en Afrique de l'Ouest.
An. Soc. belge Med. trop. Vol. 38 N°1 Janvier-Février 1978 P. 120-125.

27 - OUZILLEAU - LAIGRET et LEFROU (1921)
Contribution à l'étude de l'onchocerca-volvulus Bull Soc. Path Exot T 14 P.717-728.

28 - PENCHENIER (M) - LOUVET (M) - THERIZOL (F)
Etude parasitologique de l'onchocercose en population lèpreuse et non lèpreuse.
Bull Soc. Path Exot 1981 Vol. 74 P.273-277.

29 - PHILIPPON Bernard
L'onchocercose . Travaux et documents de l'ORSTOM P. 226-227.

30 - PHILIPPON (B) - LE BERRE (R) - BALAY (G) et ROLLAND (A)
Mise au point à partir de critères entomologiques d'un modèle mathématique traduisant l'intensité de la transmission de l'onchocercose. Relation avec les manifestations cliniques applications.
Rapport final 9e Conférence de l'OCCGE (1974) Bobo-Dioulasso. T 1 P. 232.

31 - PICQ (J.J.) et ROUX (J)
Intantanés sur l'éndémie onchocerquienne en Afrique de l'Ouest.
XIV Conférence technique de l'OCCGE (Avril 1974) P. 49.

32 - PICQ (J.J.) et JARDEL (J.P) (1974)
Une méthode d'évaluation des densités microfilariennes d'onchocerca volvulus. Leuckart (1893) chez des onchocerquiens Technique et temps de lecture des biopsies cutanées.
Bull OMS Vol. 45 P. 517-520.

33 - PROST (A) - PRODHON (J)
Le Diagnostic parasitologique de l'onchocercose. Revue critique des méthodes en usage.
An. Soc. belge Med. Trop. Vol. 38 N°5 Sept-Oct. 1978 P. 519-529.

35 - RIPERT (Ch) - RIEDEL (D) - YANG (R) et al (1977)
Etude épidémiologique de l'onchocercose dans cinq villages de la vallée de la Sanaga (Cameroun) Bull Soc. Path. Exot. T.70 P.178-186.

36 - SAME EKOBO
Contribution à l'Etude de l'onchocercose dans la vallée de la Sanaga (Cameroun)
Thèse de Doct. en Méd. (1976) P. 81-86.

37 - SEVENY (J) - MALOSSE (D) - APRIOU (M) - TRIBOULEY (J) -ENYONG ([illegible]) - SAME EKOBO - RIPERT (Ch)
Etude épidémiologique de l'onchocercose chez les matakams des Monts du Mandara (Nord Cameroun)
Bull Soc. path Exot. T 74 (1981) P.197-206.

38 - TCHAMFONG-DJABO-ROGER
Contribution à l'Etude épidémiologique des filarioses humaines chez les pêcheurs Douala de l'Estuaire de Wouri (Cameroun)
Thèse de Doct. en Méd. (1978) P.25-28.

39 - YANG (R)-FOUDA (A) - RIEDEL (D)
Etude épidémiologique de l'onchocercose dans la vallée de la Sanaga du village de Ndjoré (sous-Prefecture de Mbandjok (Cameroun)
Med. d'Afrique Noire (Mars 1977) T. 24 P.191-195.

40 - YEBAKIMA (A) - MENEZ (B) - MOUYEKE (M) 1979
Rapport d'enquête sur les aspects oculaires de l'onchocercose dans le village Kinsassa (district de Kinkala-Congo (archives DMP Brazzaville) P. 2-3.

41 - YEBAKIMA (A) - BAYA-TSIKA (N) -COULM (J) - MOLOUBA (R)
L'onchocercose en République populaire du Congo Conférence . Technique de l'OCEAC (Avril 1978)
(Archives DMP Brazzaville) P.3-7.

../..

42 - YEBAKIMA (A) et FREZIL (J.L) (1978)
Simulies et onchocercose au Congo
Rapport d'enquête
(archives DMP Brazzaville) P.3-9.

43 - YEBAKIMA (A) - BAYA-TSIKA et Coll
L'Onchocercose dans la région de Brazzaville
(Congo) Note préliminaire
Bull Soc. Path: . Exot. 1979 Vol. 72 P.35-40.

44 - YEBAKIMA (A) (1978)
L'Onchocercose humaine au Congo
Etude du foyer de Bangou-Louholo
(Thèse de Doct. en Méd. page 50-58.

45 - YEBAKIMA (A) - FREZIL (J.L) - MAHOUKOU (F) - NKOUKA (D) et MANNONI (F) (1980)
L'onchocercose dans le village Kinsassa
(district de Kinkala, région du Pool) RPC
(Archives DMP Brazzaville) P. 4 et 5.

46 - YEBAKIMA (A) - OBOUAKA (V) - KONONGO (J.D) - NANGA (M) et COSMANS (M) (1979)
Enquête onchocercose dans le foyer du Djoué (RPC)
(Archives DMP Brazzaville) Page 2-3.

ANNEXES

Annexe "A"

MINISTERE DE LA SANTE ET DES AFFAIRES SOCIALES
-=-=-=-=-
DIRECTION GENERALE DE LA SANTE PUBLIQUE
-=-=-=-=-=-

REPUBLIQUE POPULAIRE DU CONGO
TRAVAIL-DEMOCRATIE-PAIX
-=-=-=-=-=-=-

N° 4440 /MSAS/DGSP/DMP.-

NOTE DE SERVICE

-=-=-=-=-=-=-%%%-=-=-=-=-=-=-=-

portant Mission Onchocercose dans la Région du POOL.

Une équipe du Programme Onchocercose de la Direction de la Médecine Préventive se rendra dans le district de Boko du 18 Novembre 1985 au 25 Novembre 1985 pour prospecter les localités suivantes :

- Foota
- Kimpenga
- Bela
- Mantaba
- Mandombé

But : 1) Poursuite de l'Etude Cartographique de l'Onchocercose au Congo.

2) Evaluation de l'Endémie Onchocerquienne dans le district de Boko.

Composition de l'Equipe :

1) Monsieur NTSOUMOU MADZOU Victor Technicien supérieur Epidémiologiste

2) Monsieur MIALEBAMA Jean Etudiant OCEAC

3) Monsieur MILONGO Gaston Chauffeur.

Moyen de Transport : Toyota N° 950 BHQ4.

Prestation demandée sensibilisation de la population.

Ampliations :

- MSAS.......... 2
- DMP........... 2
- REGION POOL....2
- DIR. REG. POOL..2
- DISTRICT BOKO...2
- SECT. DP. N° 10..2
- SEGE.............2
- SETP.............2
- PROGRAMME ONCH...1
- ARCHIVES.........2

Fait à Brazzaville, le 13 Novembre 1985

DR. A. GANDO.-

Annexe "B"

PROGRAMME DE TRAVAIL

-=-=-=-=-=-=-=-=-%%%-=-=-=-=-=-

Le 18 - 11 - 85 départ - Trajet Brazzaville - Foota

Du 19 - 11 - 85 au 21 - 11 - 85 - Travail à Foota

Le 21 - 11 - 85 nuit à Kimpenga

Le 22 - 11 - 85 Travail à Kimpenga nuit à Mantaba

Le 23 - 11 - 85 Travail à Mantaba nuit à Bela

Le 24 - 11 - 85 Travail à Bela nuit à Mandombé

Le 25 - 11 - 85 Travail à Mandombé

Le 26 - 11 - 85 Retour à Brazzaville.

Annexe 1

Tableau N° 50

Répartition de l'indice microfilarien (IM) en fonction de l'âge et du sexe dans l'ensemble des 5 villages Foota Mantaba - Kimpenga - Bela - Mandombe (observations de la période du 18 au 26 Novembre 1985)

Tranches d'âges	Sujets examinés			Porteurs des microfilaires d'onchocerca-volvulus					
	Hommes	Femmes	Total	Hommes	IM %	Femmes	IM %	Total hommes+femmes	IM %
0 - 4 ans	4	6	10	2	50	3	50	5	50
5 - 9 ans	115	106	221	62	53,91	53	50	115	52,03
10 - 14 ans	109	124	233	66	60,55	91	73,38	157	67,38
15 - 29 ans	59	111	170	47	79,66	98	88,28	145	85,29
30 - 49 ans	60	131	191	53	88,33	122	93,12	175	91,62
50 ans et +	124	157	281	113	91,12	143	91,08	256	91,10
Total	471	635	1106	343	72,82	510	80,31	853	77,12

IM = Indice microfilarien

ANNEXE 2

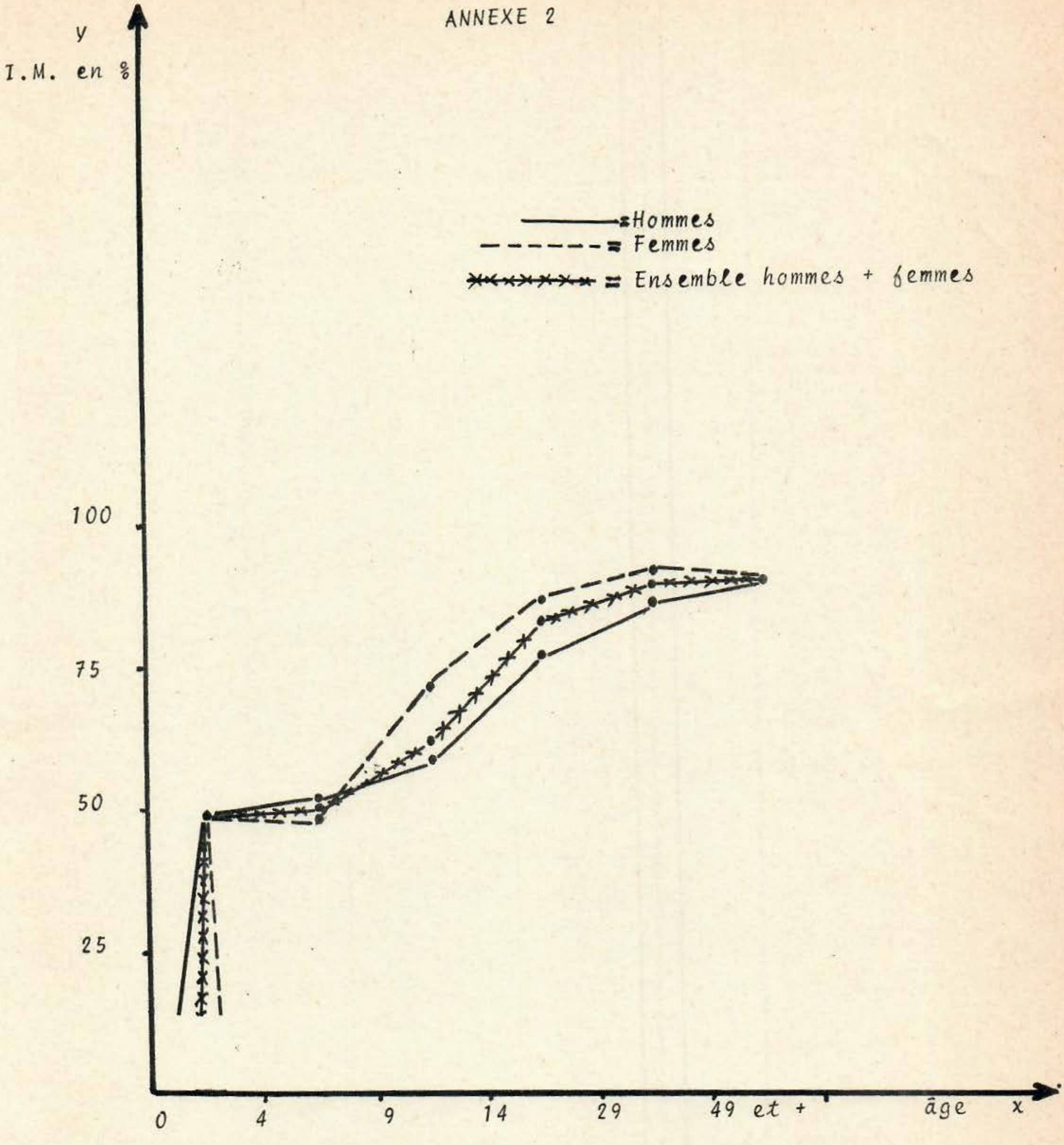

<u>Graphique N°1</u>

REPARTITION DE L'INDICE MICROFILARIEN EN FONCTION DE L'AGE ET DU SEXE DANS L'ENSEMBLE DES VILLAGES FOOTA-MANTABA - KIMPENGA - BELA - MANDOMBE

(Observations de la période du 18 au 26 Novembre 1985)

Annexe 3

Tableau N° 51

Répartition de l'indice kystique (IK) en fonction de l'âge et du sexe dans l'ensemble des 5 villages (Foota-Mantaba-Kimpenga-Bela-Mandombe) (Observations de la période du 18 au 26 Novembre 1986)

Tranches d'âges	Sujets examinés			Porteurs de modules onchocerquiens					
	Hommes	Femmes	Total	Hommes	IK %	Femmes	IK %	Total hommes+femmes	IK %
0 - 4 ans	4	6	10	2	50	1	16,66	3	30
5 - 9 ans	115	106	221	2	1,73	3	2,83	5	2,26
10 - 14 ans	109	124	233	4	3,66	1	0,80	5	2,14
15 - 29 ans	59	111	170	8	13,55	14	12,61	22	12,94
30 - 49 ans	60	131	191	24	40	56	42,74	80	41,88
50 ans et +	124	157	281	45	36,29	71	45,22	116	41,28
Total	471	635	1106	85	18,04	146	22,99	231	20,88

Annexe 4

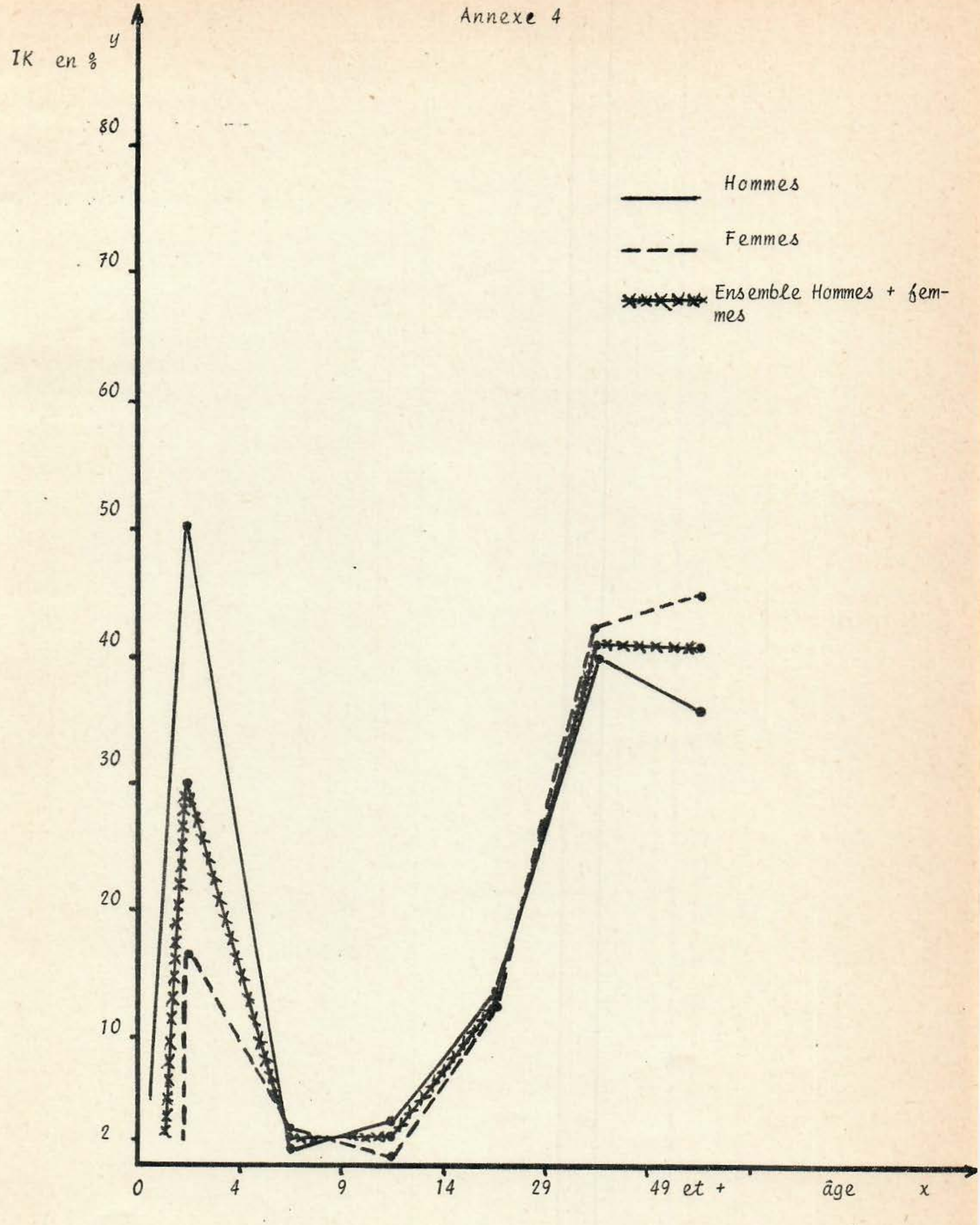

Graphique N° 2

REPARTITTION DE L'INDICE KYSTIQUE EN FONCTION DE L'AGE ET DU SEXE DANS L'ENSEMBLE DES VILLAGES FOOTA-MANTABA-KIMPENGA-BELA-MANDOMBE)

Annexe 5

Tableau N° 52

Répartition des porteurs de microfilaires et/ou de nodules en fonction de l'âge et du sexe dans l'ensemble des 5 villages (Foota-Mantaba-Kimpenga-Bela-Mandombe; (observations de la période du 18 au 26 Novembre 1985).

Tranches d'âges	Sujets examinés			Porteurs de microfilaires		Porteurs de nodules uniquement		Total des sujets considérés onchocerquiens	I C P %
	Hommes	Femmes	Total	Hommes	Femmes	Hommes	Femmes		
0 - 4 ans	4	6	10	2	3	0	0	5	50
5 - 9 ans	115	106	221	62	53	1	1	117	52,94
10 - 14 ans	109	124	233	66	91	1	0	158	67,81
15 - 29 ans	59	111	170	47	98	2	0	147	86,47
30 - 49 ans	60	131	191	53	122	2	2	179	93,71
50 ans et +	124	157	281	113	143	6	0	262	93;23
Total	471	635	1106	343	510	12	3	868	78,48

Ce tableau indique donc la répartition de l'indice clinico-parasitologique (I C P)

Annexe 6

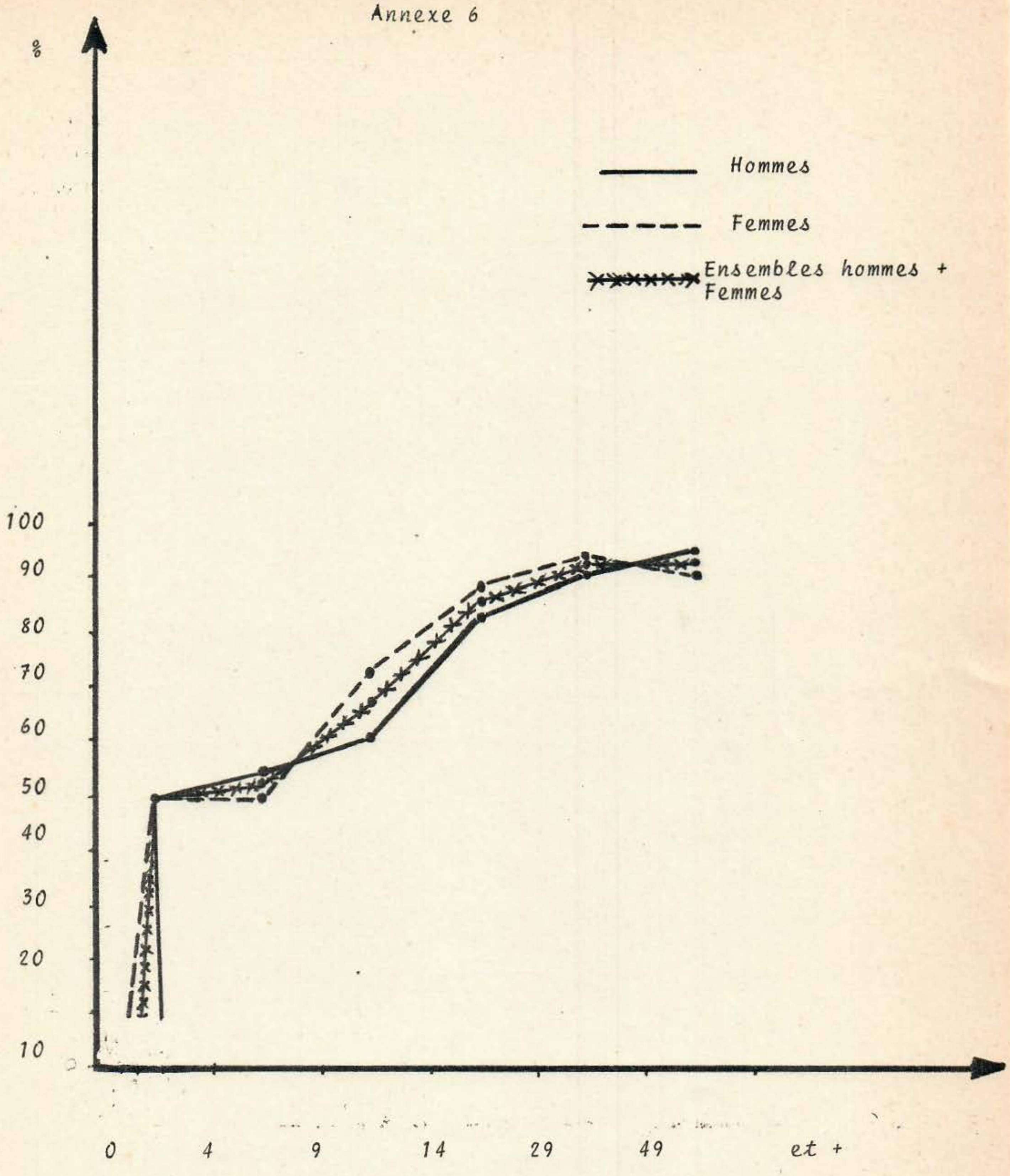

Graphique N° 3

Variation de l'I C P dans l'ensemble de 5 villages Foota-Mantaba-Kimpenga-Bela-Mandombe en fonction de l'âge et du sexe (observations de la période du 18 au 26 Novembre 1985)

ICP = Indice clinico-parasitologique

Annexe 7

Tableau N° 56 Charges parasitaires observées dans l'ensemble des 5 villages :

Foota-Mantaba - Kimpenga - Bela - Mandombe en fonction de l'âge et du sexe pendant la période du 18 au 26 Novembre 1985)

Tranches d'âges	Sexe masculin				Sexe féminin				Ensemble sexe masculin + sexe féminin			
	Sujets examinés	Cas +	C P T	C P M	Sujets examinés	Cas +	C P T	C P M	Sujets examinés	Cas +	C P T	C P M
0 - 4 ans	4	2	10	5	6	3	22,5	7,5	10	5	32,5	6,5
5 - 9 ans	115	62	813,5	13,12	106	53	823,5	15,53	221	115	1637	14,23
10 - 14 ans	109	66	1139	17,25	124	91	1666	18,30	233	157	2805	17,86
15 - 29 ans	59	47	2195,5	46,71	111	98	5165	52,70	170	145	7360,5	50,76
30 - 49 ans	60	53	3887	73,33	131	122	7406,5	60,70	191	175	11293,5	64,53
50 ans et+	124	113	7701	68,15	157	143	5659	39,57	281	256	13360	52,18
Total	471	343	15746	45,90	635	510	20742,5	40,67	1106	853	36488,5	42,77

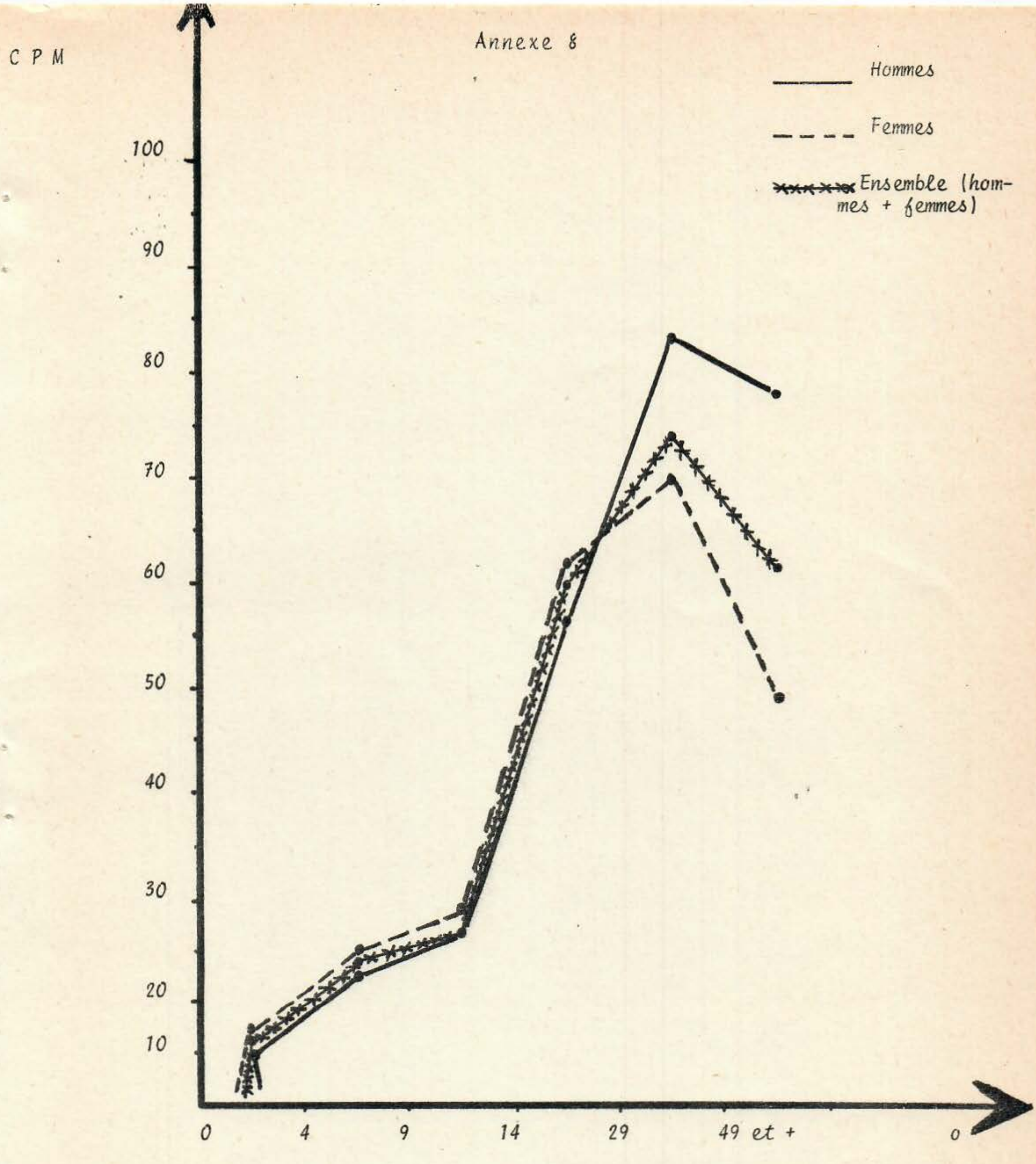

Graphique N° 5

Distribution des charges parasitaires moyennes dans l'ensemble des cinq villages : Foota-Mantaba-Kimpenga-Bela- Mandombé (observations de la période du 18 au 26 Novembre 1985)

Annexe 9

Tableau N° 54

Distribution des Signes cliniques en fonction de l'âge dans l'ensemble des 5 villages (Foota-Mantaba-Kimpenga-Bela-Mandombé) (observations de la période du 18 au 26 Novembre 1985)

Tranches d'âges	Sujets examinés	Porteurs des nodules	Porteurs des manifestations cutanées					Atteintes oculaires	
			Prurit	Dépigmentation tib	Atrophie cutanée	Gale filarienne.	Pachydermie	Perte de vue grave	Cécité totale
0 - 4 ans	10	3	4	0	0	0	0	0	0
5 - 9 ans	221	5	39	0	0	0	0	0	0
10 - 14 ans	233	5	42	0	0	1	0	0	0
15 - 29 ans	170	22	53	2	0	0	0	1	0
30 - 49 ans	191	80	102	13	2	0	0	8	2
50 ans et +	281	116	169	69	49	2	22	43	11
Total	1106	236	406	84	51	3	22	52	13

Annexe 10

Graphique N° 6

Barrogramme représentant la distribution des signes cliniques observés dans l'ensemble des 5 villages : (Fogta-Mantaba Kimpenga -Bela-Mandombe) pendant la période du 18 au 26 Novembre 1985)

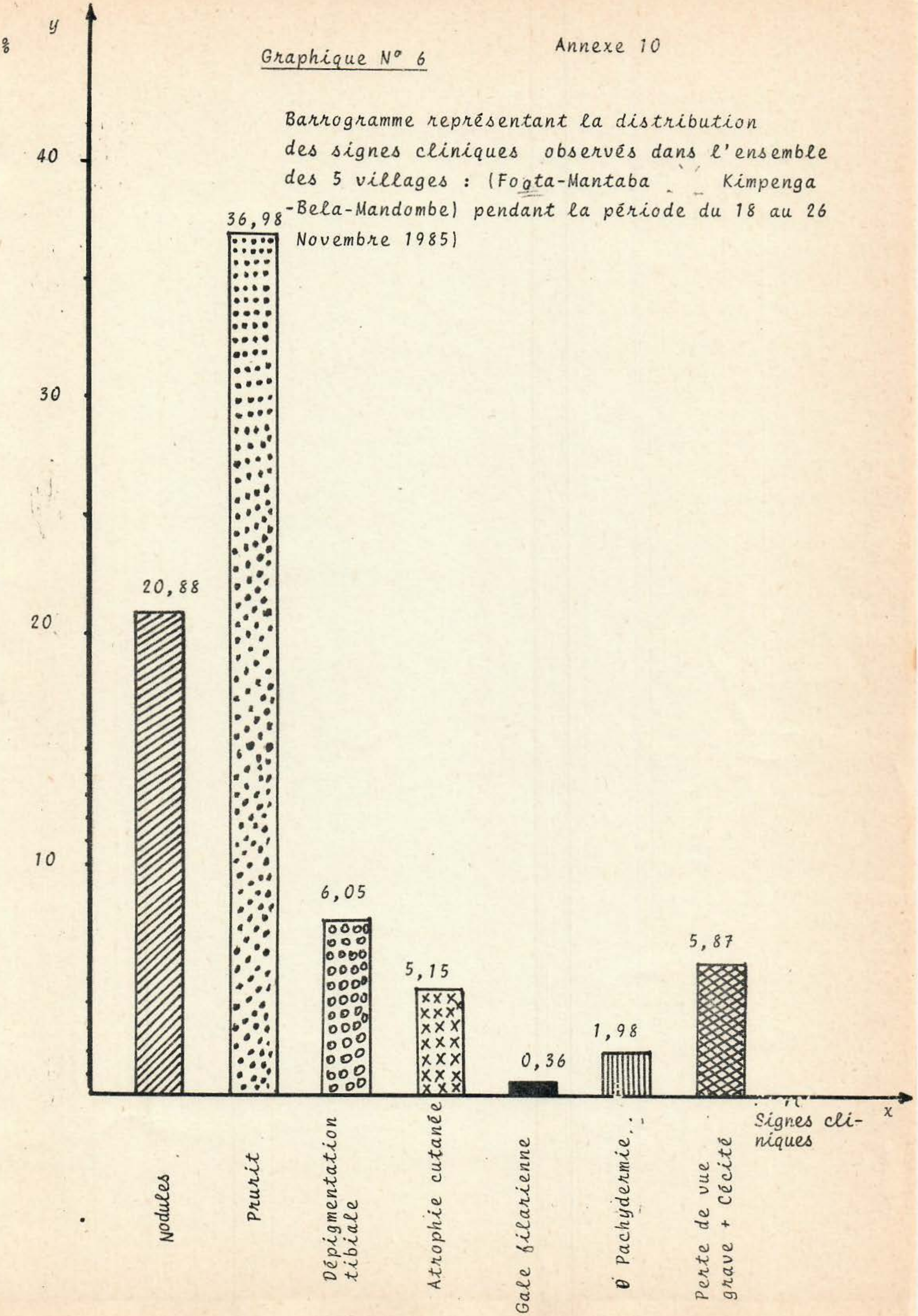

Annexe 11

Tableau N° 53

Distribution des atteintes oculaires dans l'ensemble des 5 villages (Foota - Mantaba - Kimpenga - Bela - Mandombé) en fonction de l'âge et du sexe (observations de la période du 18 au 26 Novembre 1985)

Tranches d'âges	Sujets examinés			B A V				Perte de vue grave + Cécité			
	Hommes	Femmes	Total	Hommes	%	Femmes	%	Hommes	%	Femmes	%
0 - 4 ans	4	6	10	0	0	0	0	0	0	0	
5 - 9 ans	115	106	221	0	0	0	0	0	0	0	
10 - 14 ans	109	124	233	0	0	0	0	0	0	0	
15 - 29 ans	59	111	170	1	1,69	1	0,90	0	0	1	0,90
30 - 49 ans	60	131	191	3	5	5	3,81	2	3,33	7	5,34
50 ans et +	124	157	281	16	12,90	14	8,91	17	13,70	38	24,20
Total	471	635	1106	20	4,24	20	3,14	19	4,03	46	7,24

BAV = Baisse de l'acuité visuelle

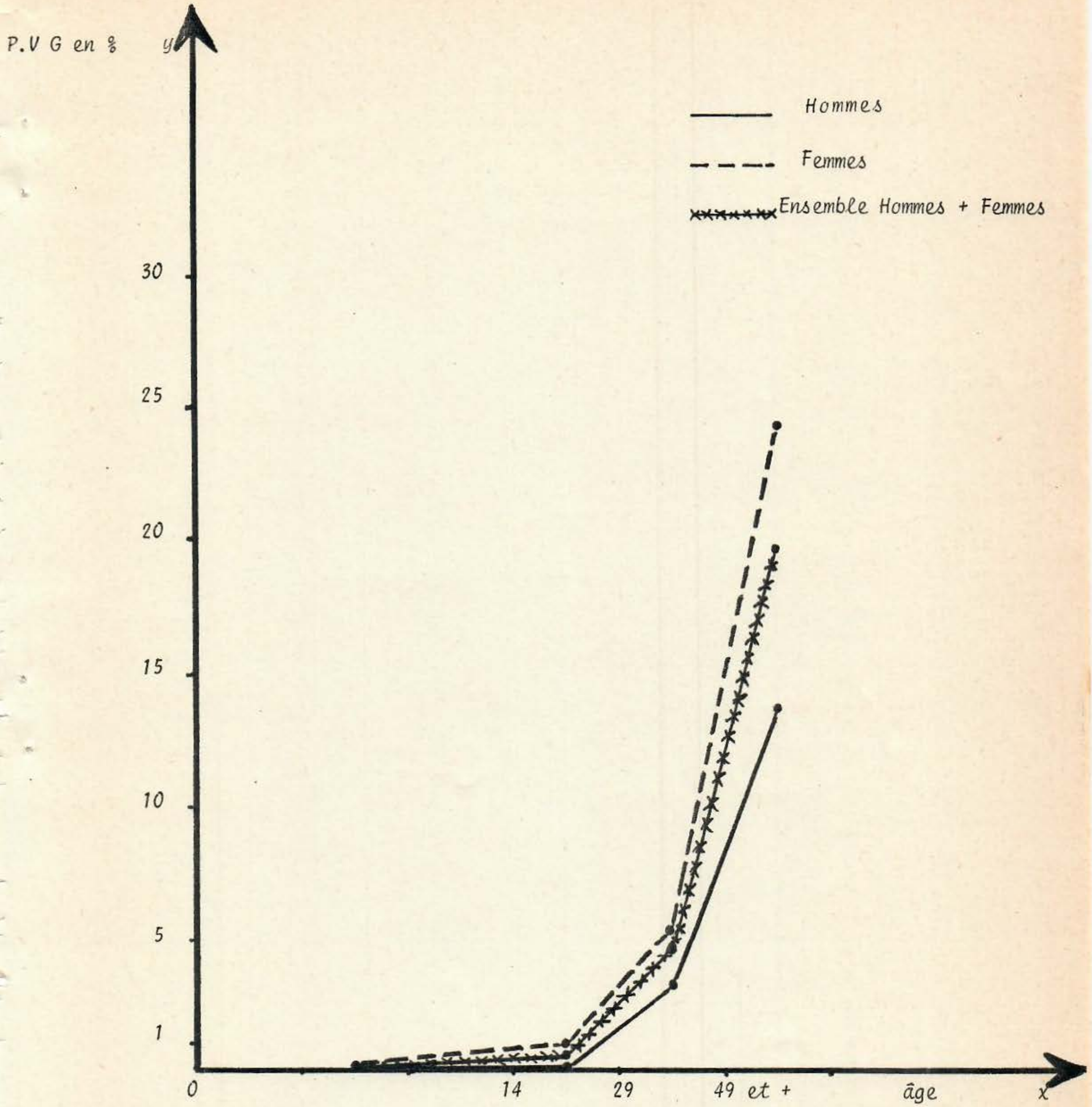

<u>Graphique N° 4 :</u> GRAVE

DISTRIBUTION DE LA PERTE DE VUE + CECITE EN FONCTION DE L'AGE ET DU SEXE DANS L'ENSEMBLE DES VILLAGES : FOOTA-MANTABA-KIMPENGA-BELA-MANDOMBE

(Observations de la période du 18 au 26 Novembre 1985)

(Perte de vue grave = impossibilité pour le malade de compter les doigts de la main à 3 mètres.)

Annxe 13

Tableau N° 55

Répartition de l'endémie onchocerquienne en fonction de l'âge et du sexe dans l'ensemble des 5 villages (Foota-Mantaba-Kimpenga-Bela-Mandombé)
(observations de la période du 18/11/85 au 26 Novembre 1985)

Tranches d'âges	Sujets examinés			Sujets onchocerquiens			Sujets non onchocerquiens		
	Hommes	Femmes	Total	Hommes	Femmes	Total	Hommes	Femmes	Total
0 - 4ans	4	6	10	2	3	5	2	3	5
5 - 9 ans	115	106	221	63	54	117	52	52	104
10 - 14 ans	109	124	233	67	91	158	42	33	75
15 - 29 ans	59	111	170	49	98	147	10	13	23
30 - 49 ans	60	131	191	55	124	179	5	7	12
50 ans et +	124	157	281	119	143	262	5	14	19
Total	471	635	1106	355	513	868	116	122	238

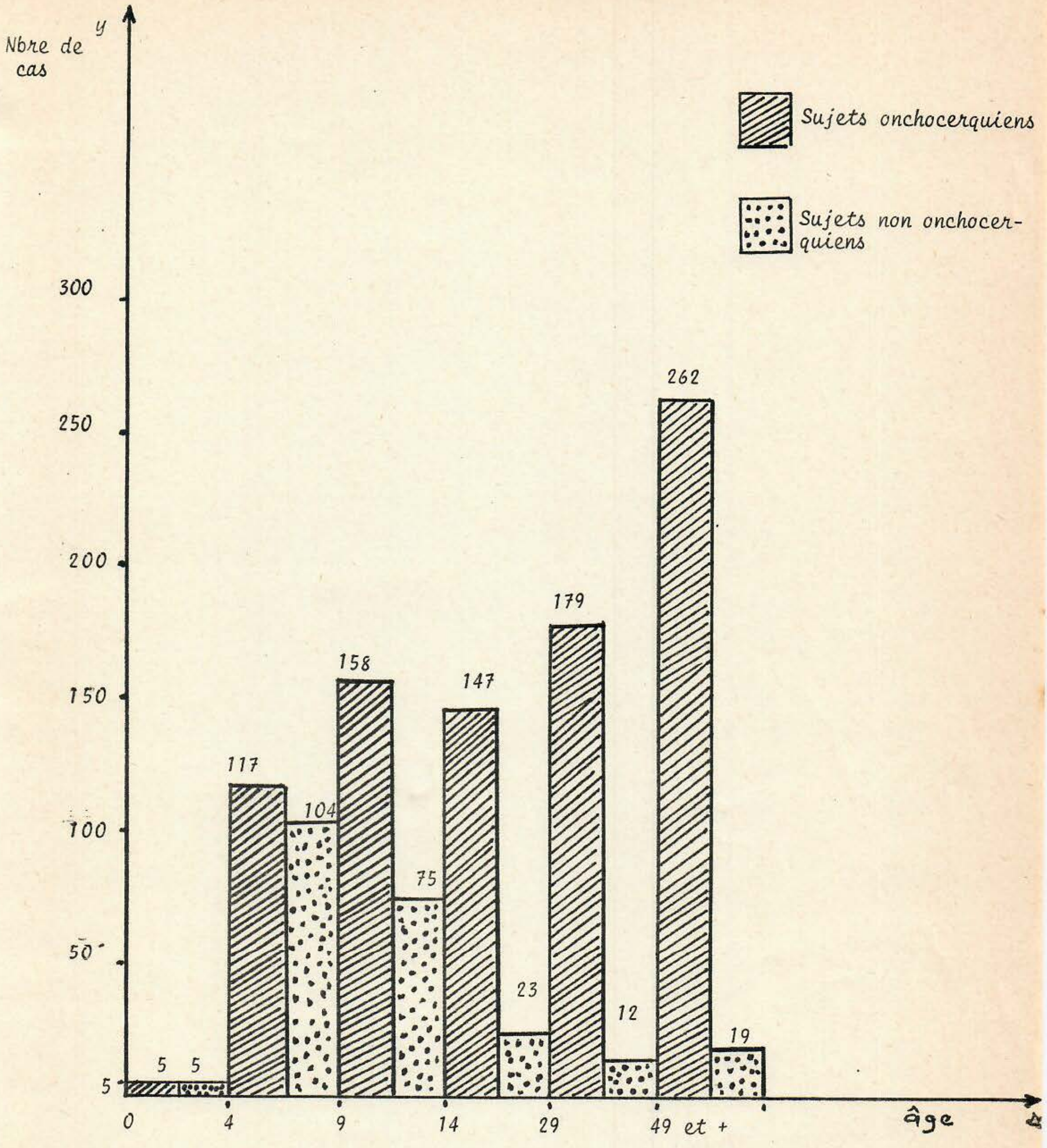

Graphique N° 7 Histogramme représentant la distribution de l'onchocercose en fonction de l'âge dans l'ensemble des villages : Foota-Mantaba- Kimpenga - Bela - Mandombe (observations de la période du 18 au 26 Novembre 1985)

Annexe 15

Onchocercose dans LE BASSIN DU DJOUE

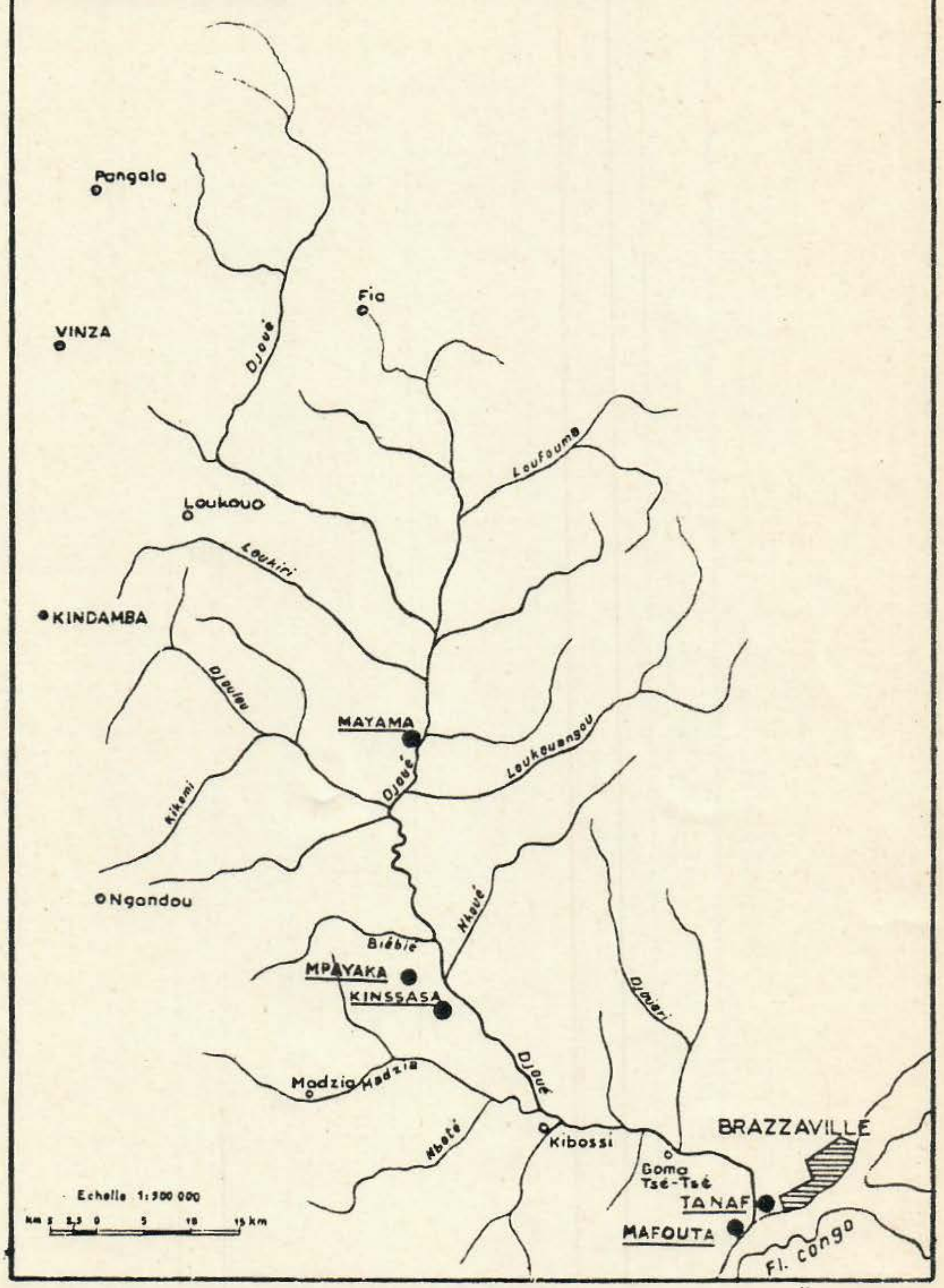

Annexe 16

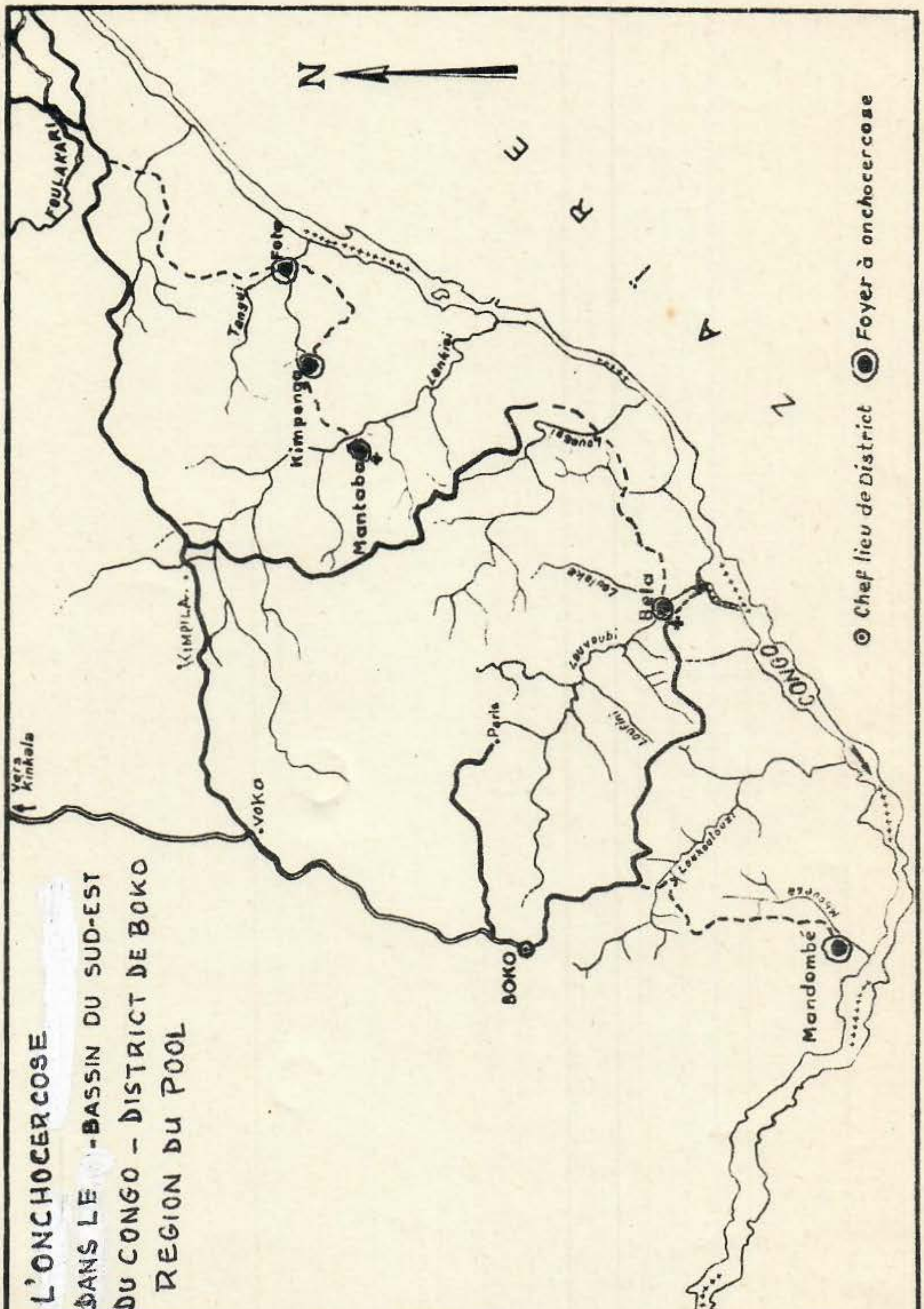
L'ONCHOCERCOSE
DANS LE -BASSIN DU SUD-EST
DU CONGO - DISTRICT DE BOKO
REGION DU POOL
N
ZAIRE
FOULAKARI
Kimpanga
Mantaba
Bela
Louvoubi
Paris
KIMPILA
Vers Kinkala
VOKO
BOKO
CONGO
Mandombe
Chef lieu de District
Foyer à onchocercose

ANNEXE 17

MODELE DE FICHE D'ENQUETE.

N° FICHE [ ] PAYS [ ] DATE [ ] FORET/SAVANE [ ] SAISON [ ]

J.M.A.

PROVINCE [ ] ARRONDISSEMENT [ ] VILLAGE [ ]

N O M [ ] P R E N O M [ ] A G E [ ]

NOM - PRENOM DU CHEF DE FAMILLE [ ] QUARTIER [ ]

EPOUX - EPOUSE de (1) [ ] PROFESSION [ ]

Fils, Fille de (1) [ ] PROFESSION [ ]

RESIDE AU VILLAGE DEPUIS (année) [ ] DATE PRECEDENT EXAMEN [ ]

(1) rayer la mention inutile.

EXAMEN PARASITOLOGIQUE

BIOPSIE CUTANEE EXSANGUE QUANTITATIVE

RESULTATS

CRETE ILIAQUE DROITE

CRETE ILIAQUE GAUCHE

AUTRES LOCALISATIONS.

OBSERVATIONS :

ANNEXE 18

N° FICHE	N O M	Age	Sexe M/F	BIOPSIE			LESIONS CUTANEES								ACUITE VISUELLE		Nbre de nodules		
				I	II	III	1	2	3	4	5	6	7	8	PERTE GRAVE	CECITE	I	II	III

ANNEXE 19

A G E	SUJETS EXAMINES			PERTE GRAVE VISION			CECITE			TAUX DE CECITE			DENSITE MICROFILARIENNE MOYENNE		
	M	F	TOTAL	M	F	TOTAL	M	F	TOTAL	M	F	TOTAL	M	F	TOTAL
0-4 ANS															
5-9 ANS															
10-14 ANS															
15-29 ANS															
30-49 ANS															
PLUS DE 50 ANS															
TOTAL															
