# Supplementary material for: Historical overview and geographical distribution of neglected tropical diseases amenable to preventive chemotherapy in the Republic of the Congo: A systematic review
Source: PLoS Negl Trop Dis. 2022 Jul 11;16(7):e0010560. doi: 10.1371/journal.pntd.0010560 (PMC9302787; doi:10.1371/journal.pntd.0010560)
Supplement: S4 Appendix — (PDF) [file pntd.0010560.s004.pdf]

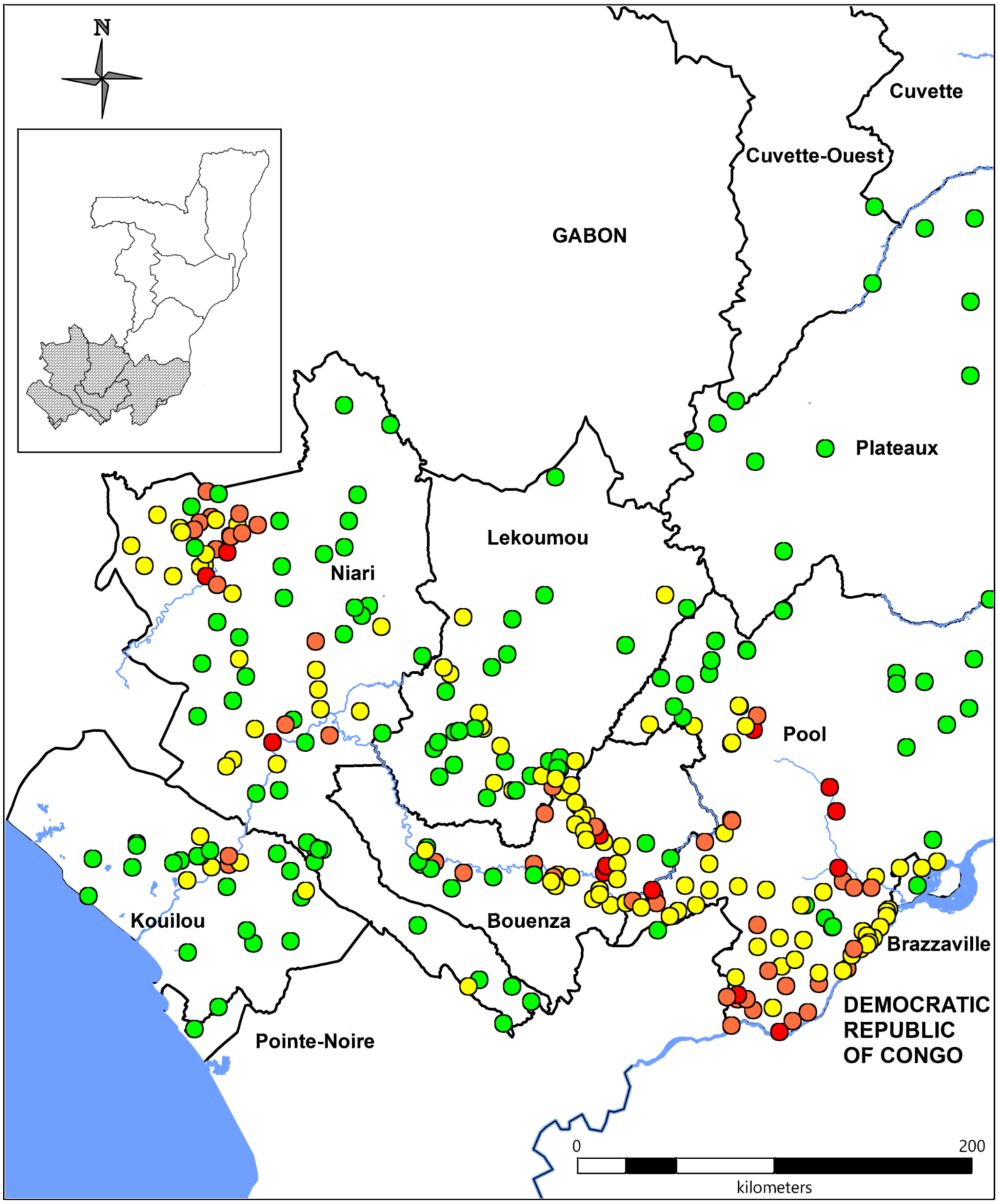

Administrative division

Departmental boundaries

Hydrography

River

Atlantic Ocean

Prevalence of nodules (%)

REMO results

Non-endemic : < 5

Hypo-endemic : [5 - 20[

Meso-endemic : [20 - 40[

Hyper-endemic : >= 40

Niari = Department name

GABON = Border country name

Created by : *Joseph A. Ngatse*

Juin 2021
