# Supplementary material for: Historical overview and geographical distribution of neglected tropical diseases amenable to preventive chemotherapy in the Republic of the Congo: A systematic review
Source: PLoS Negl Trop Dis. 2022 Jul 11;16(7):e0010560. doi: 10.1371/journal.pntd.0010560 (PMC9302787; doi:10.1371/journal.pntd.0010560)
Supplement: S5 Appendix — (DOCX) [file pntd.0010560.s005.docx]

**Table.** Most recent (2018) information on the target population size for MDA against onchocerciasis in each Department and Health District.

| **Department** | **Heath Districts** | **Administrative Districts** | **Year of first CDTI** | **Target population** |
| --- | --- | --- | --- | --- |
| Bouenza | Loudima | Nkayi, Kayes, Loudima | 2002 | 48,342 |
|  | Loutété | Yamba, Mfouati | 2002 | 20,376 |
|  | Madingou | Madingou, Mabombo | 2002 | 48,886 |
|  | Mouyondzi | Mouyondzi, Ntsiaki, Kingoué | 2002 | 33,612 |
|  | **Total** |  |  | **151,216** |
|  |  |  |  |  |
| Brazzaville | Bacongo | Bacongo | 2001 | 120,222 |
|  | Madibou | Madibou | 2001 | 101,185 |
|  | Makélékélé | Makélékélé | 2001 | 89,151 |
|  | Mfilou | Mfilou | 2001 | 75,367 |
|  | **Total** |  |  | **385,925** |
|  |  |  |  |  |
| Lekoumou | Sibiti | Mayeye | 2004 | 595 |
|  | **Total** |  |  | **595** |
|  |  |  |  |  |
| Niari | Dolisie | Makabana | 2003 | 8,525 |
|  | Kibangou | Kibangou, Divenie | 2003 | 11,379 |
|  | **Total** |  |  | **19,904** |
|  |  |  |  |  |
| Pool | Kinkala-Boko | Boko, Louingui, Loumo, Kinkala | 2001 | 39,419 |
|  | Goma tsetse | Goma Tsetse, Mbanza Ndounga | 2001 | 20,706 |
|  | Ignié | Mayama | 2001 | 2,155 |
|  | Kindamba | Kindamba | 2015 | 11,504 |
|  | Mindouli | Mindouli | 2002 | 39,719 |
|  | **Total** |  |  | **113,503** |
|  |  |  |  |  |
| Kouilou | Mvouti-Kakamoeka | Kakamoeka | 2003 | 1,134 |
|  | **Total** |  |  | **1,134** |
| **TOTAL** |  |  |  | **672,276** |
