# Supplementary material for: Historical overview and geographical distribution of neglected tropical diseases amenable to preventive chemotherapy in the Republic of the Congo: A systematic review
Source: PLoS Negl Trop Dis. 2022 Jul 11;16(7):e0010560. doi: 10.1371/journal.pntd.0010560 (PMC9302787; doi:10.1371/journal.pntd.0010560)
Supplement: S6 Appendix — (DOCX) [file pntd.0010560.s006.docx]

**S1. Mass drug administration of ivermectin for onchocerciasis in the Republic of Congo. Source: National Program for Onchocerciasis Control**

|  |  |  | **Communities** | | | **Population** | | | **Health District** | | |
| --- | --- | --- | --- | --- | --- | --- | --- | --- | --- | --- | --- |
| **N°** | Year | **Number of years of treatment** | **Total number** | **Number of treated communities** | **Geographic coverage (%)** | **Total** | **Number of treated people** | **Therapeutic coverage (%)** | **Total** | **Number of treated HD** | **Geographic coverage (%)** |
| 0 | 1999 |  |  |  |  |  |  |  |  |  |  |
| 0 | 2000 |  |  |  |  |  |  |  |  |  |  |
| 1 | 2001 | 1 | 748 | 425 | 56,8 | 578 812 | 228 220 | 39,4 |  |  |  |
| 2 | 2002 | 2 | 748 | 468 | 62,6 | 583 480 | 195 950 | 33,6 |  |  |  |
| 3 | 2003 | 3 | 748 | 719 | 96,1 | 569 652 | 353 281 | 62,0 |  |  |  |
| 4 | 2004 | 4 | 770 | 765 | 99,4 | 575 418 | 383 302 | 66,6 |  |  |  |
| 5 | 2005 | 5 | 770 | 770 | 100,0 | 582 192 | 406 031 | 69,7 |  |  |  |
| 6 | 2006 | 6 | 770 | 770 | 100,0 | 593 934 | 416 963 | 70,2 |  |  |  |
| 7 | 2007 | 7 | 770 | 770 | 100,0 | 609 925 | 449 171 | 73,6 |  |  |  |
| 8 | 2008 | 8 | 770 | 770 | 100,0 | 644 224 | 478 692 | 74,3 |  |  |  |
| 9 | 2009 | 9 | 770 | 770 | 100,0 | 764 915 | 617 167 | 80,7 |  |  |  |
| 10 | 2010 | 10 | 770 | 770 | 100,0 | 803 026 | 651 922 | 81,2 |  |  |  |
| 11 | 2011 | 11 | 770 | 770 | 100,0 | 844 656 | 686 127 | 81,2 |  |  |  |
| 12 | 2012 | 12 | 770 | 770 | 100,0 | 848 620 | 690 234 | 81,3 |  |  |  |
| 13 | 2013 | 13 | 770 | 770 | 100,0 | 886 606 | 710 492 | 80,1 |  |  |  |
| 14 | 2014 | 14 | 842 | 819 | 97,3 | 756 814 | 575 731 | 76,1 |  |  |  |
| 16 | 2015 | 15 | 1 157 | 1 157 | 100,0 | 597 912 | 427 647 | 71,5 | 17 | 17 | 100 |
| 17 | 2016 | 16 | 1 242 | 1 242 | 100,0 | 605 580 | 479 469 | 79,2 | 17 | 17 | 100 |
| 18 | 2017 | 17 | 1 242 | 1 061 | 85,4 | 628 645 | 509 072 | 81,0 | 17 | 17 | 100 |
| 19 | 2018 | 18 | 1 311 | 1 270 | 96,9 | 681 801 | 546 358 | 80,1 | 17 | 17 | 100 |
|  | 2019 | 19 | 1 213 | 1 213 | 100,0 | 713 514 | 581 904 | 81,6 | 19 | 19 | 100 |
