# Supplementary material for: Historical overview and geographical distribution of neglected tropical diseases amenable to preventive chemotherapy in the Republic of the Congo: A systematic review
Source: PLoS Negl Trop Dis. 2022 Jul 11;16(7):e0010560. doi: 10.1371/journal.pntd.0010560 (PMC9302787; doi:10.1371/journal.pntd.0010560)
Supplement: S7 Appendix — (PDF) [file pntd.0010560.s007.pdf]

REPUBLIQUE DU CONGO

Unité*Travail*Progrès

MINISTERE DE LA SANTE ET DE LA POPULATION

....

UNITE DE COORDINATION DES PROGRAMMES ET PROJETS

....

COORDINATION DES MALADIES TROPICALES NEGLIGEES

PLAN DIRECTEUR DE LUTTE CONTRE LES MALADIES TROPICALES NEGLIGEES (MTN)

2018-2022

SOMMAIRE

SOMMAIRE i
ABREVIATIONS ET ACRONYMES iii
LISTE DES FIGURES v
LISTE DES GRAPHIQUES vi
LISTE DES TABLEAUX vii
PREFACE viii
INTRODUCTION 1
I. PREMIERE PARTIE ANALYSE DE LA SITUATION 2
I.1. PROFIL DU PAYS 3
I.1.1. Caractéristiques géographiques 3
I.1.2. Structures administratives, démographiques et communautaires 5
I.1.3. Contexte économique et social (PNDS 2018) Indicateurs 6
I.1.4. Transports et communications (PNDS 2018) 7
I.2. ANALYSE DU SYSTEME DE SANTE 7
I.2.1. Vision, Principes, Valeurs, But, Objectifs et Priorités de la PNS 7
I.2.2. Organisation du système de santé 8
I.3. ANALYSE DE LA SITUATION DES MTN 23
I.3.1. Situation épidémiologique et charge des maladies 23
I.3.2. Lacunes et priorités 47
II. DEUXIEME PARTIE CADRE STRATEGIQUE 48
II.1. MISSION ET OBJECTIFS DU PROGRAMME DE LUTTE CONTRE LES MTN 49
II.1.1. Vision… 49
II.1.2. Mission 49
II.1.3. But…. 49
II.1.4. Centre d'intérêt des programmes 49

II.2. PRINCIPES DIRECTEURS ET PRIORITES STRATEGIQUES 49

II.2.1. Principes directeurs 49

II.2.2. Les priorités stratégiques (PS) 50

II.2.3. Tableau Synoptique par programme 53

II.2.4. Tableau de Suivi de la performance des Programmes Nationaux 55

III. TROIXIEME PARTIE CADRE OPERATIONNEL 57

III.1. Tableau des objectifs et des activités par priorités spécifiques 58

III.2. Tableau des activités et des sous-activités par priorités spécifiques 60

IV. BUDGET ESTIMATIF DES ACTIVITES DU PLAN QUINQUENAL MTN 2018-2022 66

V. Annexe 76

ABREVIATIONS ET ACRONYMES

CAMEPS : Centrale d'Achat des Médicaments Essentiels et des Produits de Santé

CAT : Centres antituberculeux

CCIA : Comité de coordination inter-agences

CEMAC : Communauté économique et monétaire de l'Afrique centrale

CHUB : Centre Hospitalier Universitaire de Brazzaville

CNRD : Centre National de Référence de la Drépanocytose

CNTS : Centre National de Transfusion Sanguine

COGES : Comité de gestion

COMEG : Congolaise de Médicaments Essentiels Génériques

COSA : Comité de santé

CSI : Centre de santé intégré

CTA : Centres de Traitement Ambulatoire

CTS : Comité Technique de Suivi

DDS : Direction départementale de la santé

DS : District sanitaire.

ECD : Equipe Cadre du District

EPIC : Etablissement public industriel et commercial

FMI : Fonds Monétaire International

FOSA : Formation sanitaire

GAVI : Global Alliance for Vaccines and Immunization

IDH : Indicateur de Développement Humain

INS : Institut National de la Statistique

LAPCO : Laboratoire Pharmaceutique du Congo

LNSP : Laboratoire National de Santé Publique

MSP : Ministère de la Santé et de la Population

MTN : Maladies tropicales négligées

MTN-CTP : Maladies Tropicales Négligées à chimiothérapie préventive

MTN-PCC : Maladies Tropicales Négligées à prise en charge des cas

ODD : Objectifs du développement durable

OMS : Organisation mondiale de la santé

PADHRS : Projet d'Appui au Développement des Ressources Humaines de Santé

PAPN : Port Autonome de Pointe-Noire.

PBF : Financement basé sur la performance

PCA : Paquet Complémentaires d'Activités

PCIME : Prise en charge intégrée des maladies de l'enfance

PIB : Produit intérieur brut

PMA : Paquet Minimum d'Activités

PMAE : Paquet minimum d'activités élargi

PMAS : Paquet Minimum d'Activités Standard

PNDS : Plan National de développement sanitaire

PNS : Politique nationale de santé

RGPH : Recensement général de la population et de l'habitat

RHS : Ressources humaines de santé

RNDH : Rapport national sur le développement humain

SIMR : Surveillance intégrée de la maladie et la riposte

SSID : Système de santé intégré de district

SSP : Soins de Santé Primaires

SSPE : Services de Santé de Premier Echelon

LISTE DES FIGURES

Figure 1 : Carte administrative du Congo 3
Figure 2 : Répartition des schistosomiases par district sanitaire selon la cartographie de 2011. (OMS cartographie 2011) 24
Figure 3 : Répartition des géohelminthisases par district sanitaire selon la cartographie de 2011 25
Figure 4 : Présentation du statut endémique d'onchocercose au Congo. 26
Figure 5 : Situation de la délimitation de la zone de traitement et de transmission de l'onchocercose .. 27
Figure 6 : Endémicité de la Filariose lymphatique au Congo selon la cartographie de 2015 28
Figure 7 : Prévalence de la Loase au Congo (RAPLOA, 2004/2010) 30
Figure 8 : Endémicité du trachome au Congo selon la cartographie de 2015 31
Figure 9 : La répartition des foyers de THA en République du Congo ces dix dernières années (Atlas OMS 2018) 33
Figure 10 : Carte de l'endémicité de la Lèpre au Congo 2016 38
Figure 11 : Carte de l'endémicité de l'ulcère de Buruli au Congo en 2016 41

LISTE DES GRAPHIQUES

Graphique 1 : La répartition des cas de THA en République du Congo ces dix dernières années 2008-2017. 34
Graphique 2 : Situation épidémiologique du foyer de NGABE 35
Graphique 3 : Situation épidémiologique du foyer de Mossaka 36
Graphique 4 : Situation épidémiologique du foyer de Gamboma 36
Graphique 5 : Situation épidémiologique du foyer de LOUKOLELA. 37
Graphique 6 : Evolution de l'incidence de la Lèpre au Congo de 2003-2017 39
Graphique 7 : Evolution de l'incidence de la Lèpre dans les départements du Congo de 2013-2017 ... 39
Graphique 8 : Evolution de la prévalence de la Lèpre au Congo, selon les départements de 2013-2017 40
Graphique 9 : Incidence des cas d'ulcère de Buruli au Congo de 2006 à 2016 41

LISTE DES TABLEAUX

Tableau I : Structure de la population par département en 2018 6

Tableau II : Différentes densités de certaines catégories du personnel pour 1.000 habitants................ 17

Tableau III : Situation globale des Hôpitaux de District 21

Tableau IV : Synthèse des résultats RAPLOA obtenus par zone (Enquête de validation du RAPLOA dans la Lékoumou, la Bouenza et le Niari en 2003).. 29

Tableau V : Résultats de la cartographie de la Loase au Congo, menée en 2015................................. 30

Tableau VI : Résultats de la cartographie du trachome au Congo, menée en 2015. 32

Tableau VII : Répartition des foyers endémiques de THA selon les types d'endémicité dans les départements. 35

Tableau VIII : Dépistage et traitement de la THA durant cinq dernières années.................................... 37

Tableau IX : Evolution des cas d'ulcère de Buruli au Congo par départements de 2013 à 2017 42

Tableau X : Evolution des cas de Pian au Congo par départements de 2013 à 2017 42

Tableau XI : La Co endémicité des MTN et la situation de la cartographie se résument comme suit 44

Tableau XII : Les interventions des Programmes de Lutte contre les MTN-CP au Congo de 2013-2017 45

Tableau XIII : Les interventions des Programmes de Lutte contre les MTN-PCC au Congo de 2013 à 2017. 46

Tableau XIV : : Récapitulatif des Priorités Stratégiques (PS) et Objectifs retenus du plan directeur 2018-2022 51

Tableau XV : Rappel des objectifs Régionaux pour les programmes par MTN...................................... 52

Tableau XVI : Total des Activités et sous-activités retenues du plan directeur MTN quinquennal 2018-2022 65

PREFACE

Préface

Les Maladies Tropicales Négligées (MTN) demeurent encore une préoccupation majeure de santé publique en République du Congo comme dans la plupart des pays d'Afrique sub-saharienne.

Bon nombre de ces maladies ont un poids non négligeable sur la santé, les conditions de vie et la situation socio-économique des populations vivant dans les zones à risque.

L'Onchocercose, la Filariose Lymphatique (FL), la Schistosomiase, les Géo-helminthiases, le Trachome, la Trypanosomiase Humaine Africaine (THA), la Lèpre, l'Ulcère de Buruli et le Pian sont ces maladies du paysage épidémiologique du Congo aux effets délétères sur la santé et le développement.

Les priorités stratégiques choisies pour lutter contre ces MTN sont les suivantes :

- Mise à l'échelle des interventions de lutte, des traitements et des capacités de prestation de service des programmes MTN ;
- Renforcement de la planification axée sur les résultats, de la mobilisation des ressources et de la pérennité financière des programmes nationaux de lutte contre les MTN ;
- Renforcement de l'appropriation par le gouvernement, de la sensibilisation, de la coordination et des partenariats ;
- Renforcement du suivi, de l'évaluation, de la surveillance et de la recherche.

La déclinaison en activités opérationnelles de ces axes stratégiques s'inscrit dans la dynamique impulsée par l'Organisation Mondiale de la Santé pour la lutte contre les MTN.

La constitution et les différents textes fondamentaux consacrent la santé comme un droit humain et une urgence des temps présents.
Les principes et valeurs intègrent les réformes du système de santé amorcées et contenues dans le document de la Politique Nationale de Santé. Aussi la mise en œuvre des actions du présent Plan Directeur contre les MTN 2018-2022 est en harmonie avec les préoccupations du gouvernement contenues dans le projet de société de son Excellence **Denis SASSOU-NGUESSO**, « la Marche vers le développement ».

L'élaboration de ce Plan directeur de lutte contre les MTN ayant impliqué les cadres du Ministère de la Santé et de la Population et les partenaires techniques et financiers, constitue de ce fait, le seul et unique Plan MTN de référence à partir duquel devront s'aligner les stratégies des programmes et projets MTN du Ministère en charge de la santé les cinq prochaines années.

La Ministre de la Santé et de la Population

Jacqueline Lydia MIKOLO

INTRODUCTION

Les maladies tropicales négligées (MTN) sont principalement des maladies infectieuses qui sévissent dans les milieux déshérités favorisées par la chaleur et l'humidité des climats tropicaux.

Il s'agit pour la plupart des parasitoses transmises par des insectes : (1) moustiques, (2) simulies, (3) phlébotomes, (4) mouches tsétsé, (5) triatomes, (6) mouches des immondices et par des gastéropodes. D'autres sont propagées par l'eau contaminée et par les œufs de vers présents dans le sol.

La transmission se perpétue par contamination de l'environnement. Celle-ci est à son tour perpétuée par de piètres conditions de vie et une mauvaise hygiène. Autrefois très dispersées, ces maladies se concentrent aujourd'hui dans les endroits extrêmement pauvres. Ailleurs dans le monde, elles ont disparu à mesure que les conditions de vie et d'hygiène se sont améliorées.

Plus d'un milliard de personnes, soit un sixième de la population mondiale souffrent d'une ou de plusieurs de ces maladies, qui figurent très bas sur la liste de priorités des programmes sanitaires.

Ces maladies sont surtout concentrées dans les pays à faible revenu d'Afrique et d'Amérique latine, où parfois cinq à sept d'entre elles sévissent ensemble dans de vastes secteurs géographiques.

Certaines d'entre elles réduisent considérablement la productivité économique des jeunes adultes, qui se trouvent pourtant dans la fleur de l'âge. D'autres compromettent la croissance de l'enfant et le développement cognitif. Par conséquent elles provoquent une grande détresse, qui s'accompagne très souvent d'une stigmatisation et d'une discrimination sociale.

La plupart d'elles rendent aveugle ou affaiblissent l'organisme, provoquant des déformations ou des mutilations. Les troubles graves apparaissent souvent après des années d'infection subclinique pendant lesquelles les malades ne cherchent pas à se faire soigner.

D'autres maladies tropicales négligées peuvent entraîner la mort en quelques semaines ou quelques mois une fois qu'elles ont atteint un stade avancé.

La République du Congo n'est pas épargnée du fardeau de ces maladies, ou elles constituent encore un problème de santé publique. Aucun département du pays n'échappe à ce fardeau des MTN.

Parmi les MTN répertoriées par l'OMS, le Congo a engagé des actions de lutte contre l'onchocercose, la Filariose lymphatique, les schistosomiases, les géo-helminthiases, la lèpre, le pian, l'ulcère de Buruli et la Trypanosomiase Humaine Africaine.

C'est ainsi que le Gouvernement du Congo, à travers le Ministère de la Santé et de la Population (MSP) a élaboré le présent Plan Directeur de lutte contre les MTN (2018 – 2022).

PREMIERE PARTIE

ANALYSE
DE LA SITUATION

I.1 PROFIL DU PAYS

I.1.1 Caractéristiques géographiques

a. Situation et limites

La République du Congo est située au cœur de l'Afrique centrale et à cheval sur l'équateur entre les latitudes 3°30' Nord et 5° Sud, et les longitudes 11° et 18° Est. Elle s'étend sur une superficie de 342.000 km² et partage ses frontières avec 5 pays : le Gabon à l'Ouest sur 1.903 km et l'océan Atlantique sur 170Km, le Cameroun au Nord-Ouest sur 523 km, l'Angola et l'enclave du Cabinda au Sud sur 201 km, la République Centrafricaine au Nord sur 467 km et la République Démocratique du Congo à l'Est sur 2.410 km le long du fleuve Congo et la rivière Oubangui.

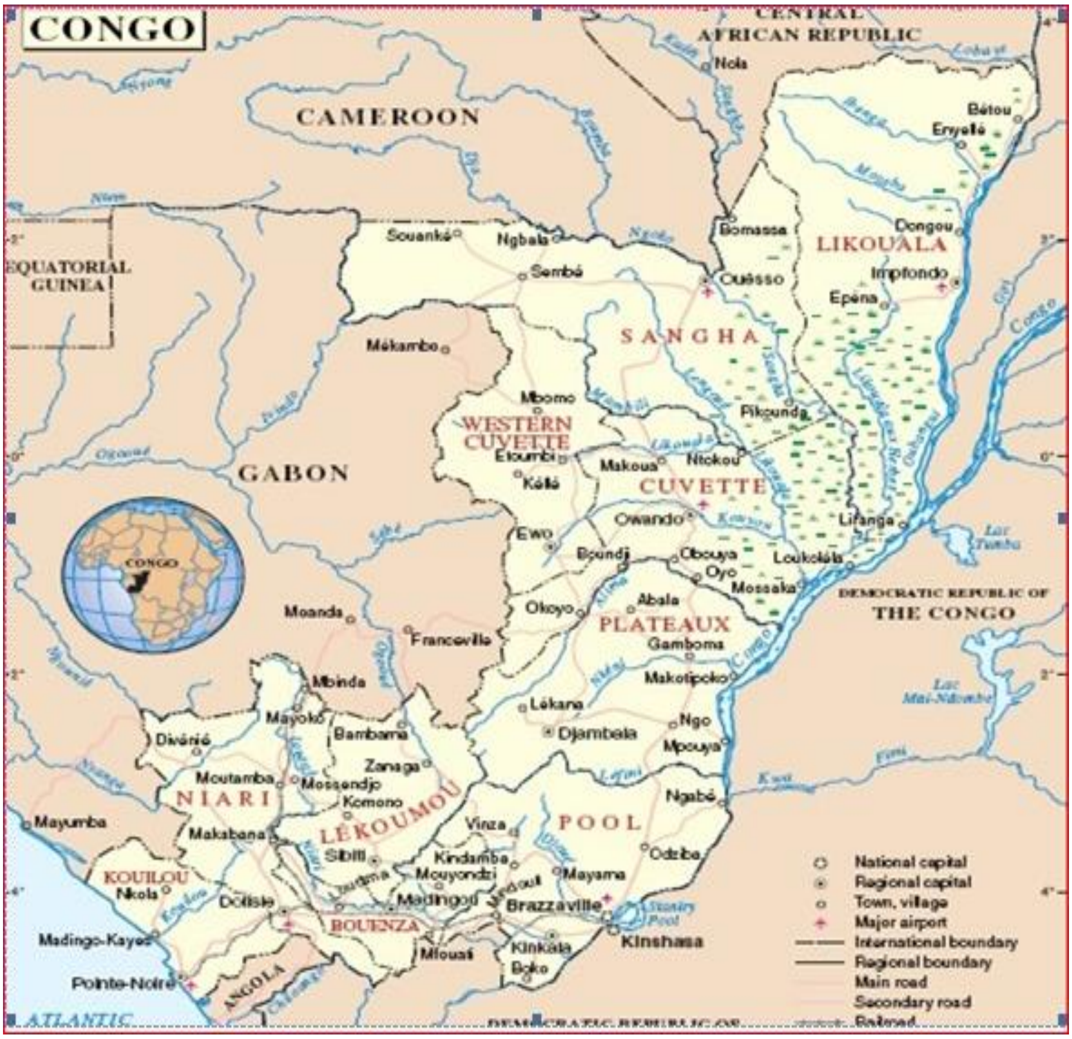

Figure 1 : Carte administrative du Congo

b. **Climat**

La République du Congo est située dans la zone des climats chauds et humides. Elle reçoit des précipitations moyennes de l'ordre de 500 ml d'eau au sud et près de 2000 ml au nord. La température moyenne se situe autour de 25˚C ; les écarts thermiques sont faibles au nord (2° C) et s'amplifient au sud (6°C). Le nord du pays bénéficie d'un climat équatorial et il pleut toute l'année avec une accalmie de décembre à février, puis en juillet. Dans le sud-ouest le climat prend une tendance tropicale humide; la saison des pluies dure 8 à 9 mois (d'octobre à mai), interrompue par une petite saison sèche en janvier ou février. La grande saison sèche dure 3 à 4 mois (mai – septembre). Les régions du centre (Plateaux Batékés et Cuvette Congolaise) connaissent un climat intermédiaire : le climat subéquatorial. Ces climats sont nuancés suivant les départements par divers facteurs notamment les masses d'eau, les courants marins et le relief.

c. Végétation

Le Congo est couvert par deux grandes zones de végétation constituées par :

- les zones forestières dont la superficie couvre environ 60% du territoire national, avec 10 % qui sont constitués de forêts denses et humides qui ont une superficie estimée à 20 millions d'hectares ;
- les zones de savane représentent près de 40% de la superficie du Congo et totalisent environ 12 millions d'hectares ;

Ces écosystèmes sont propices à l'éclosion de nombreuses pathologies tropicales.

d. **Réseau hydrographique** :

Le réseau hydrographique du Congo est composé de deux principaux bassins fluviaux : Le bassin du Congo dont 7% seulement de la superficie totale, soit 230.000 Km^2, est situé sur le territoire Congolais. Le principal collecteur est le fleuve Congo qui borde le pays sur plus de 600 Km. Ses affluents de la rive droite sont : l'Oubangui, la Likouala aux herbes, la Sangha, la Likouala - Mossaka, la Ndeko, l'Alima, la N'Keni, la Léfini et le Djoué.

Le bassin du Kouilou-Niari qui s'étend sur une superficie de 56.000 km^2, le collecteur le plus important étant le fleuve Kouilou. Il porte le nom de Niari dans son cours moyen et celui de N'Douo dans son cours supérieur. Les différents affluents sont la Bouenza, la Loutété, la Loudima et la Louessé.

Ces deux bassins constituent d'importants axes de circulation et une réserve riche en ressources halieutiques. Des crues occasionnent des inondations cycliques particulièrement dans les Départements de la Likouala, de la Cuvette et de la Sangha.

I.1.2 Structures administratives, démographiques et communautaires

a. Contexte politique et administratif (PNDS 2018)

Indépendante depuis le 15 Août 1960, la République du Congo est, comme le postule sa nouvelle constitution, un État de droit, souverain, unitaire et indivisible, décentralisé, laïc et démocratique. Le pays a en effet adopté une nouvelle constitution qui a été promulguée le 6 novembre 2015. Cette dernière a instauré le régime semi présidentiel. Les institutions issues de cette nouvelle constitution ont été progressivement mises en place. Au plan politique, le Congo a opté au début des années 90 pour le multipartisme. Le dernier processus électoral a généré des conflits qui ont occasionné l'insécurité dans le département du Pool avec des déplacements importants des populations dans les départements limitrophes. Depuis décembre 2018, le gouvernement a signé un accord de cessation d'hostilités qui a permis le retour progressif à la paix dans cette partie du pays.

La loi n°3-2003 du 17 janvier 2003 fixant l'organisation administrative territoriale, subdivise le pays en douze (12) départements, seize (16) communes, vingt-trois (23) arrondissements, quatre-vingt-huit (88) districts, cinquante-deux (52) communautés urbaines, six (6) communautés rurales, sept cent vingt (720) quartiers et trois milles deux cent quatre-vingt-seize (3296) villages.

La loi n°10 du 06 février 2003 transfert trois domaines de compétence aux collectivités locales : (i) la gestion de la santé, (ii) l'activité sociale et (iii) la protection civile. Les départements sont placés sous l'autorité des préfets, les districts, les communes et les arrondissements sont administrés, respectivement par les sous-préfets, les maires et les administrateurs maires délégués.

La décentralisation a été amorcée par la mise en place de conseils départementaux et municipaux. Le transfert de compétence et des ressources associées est en cours d'étude pour une mise en place effective. En parallèle, le statut de la fonction publique territoriale a été prévu par la loi n°5-2005. Cette dernière a fait l'objet de dix-huit décrets d'application adoptés en conseil des ministres le 29 décembre 2011. Il sied aussi de noter que dans les faits, le fonctionnement de l'Etat demeure encore assez centralisé. Le processus de décentralisation tarde à produire tous ses effets.

b. Contexte Démographie (PNDS 2018)

Le recensement Général de la population et de l'habitat (RGPH) de 2007 avait évalué la population résidente à 3 697 490 habitants, avec un taux d'accroissement de 3%. Les projections démographiques du pays calculées par l'Institut National de la Statistique (INS) en se basant sur cette hypothèse, indique le chiffre à 5 203 073 habitants en 2018, avec une prédominance des femmes à hauteur de 51%. Soixante-sept pour cent (67%) de la population vit en milieu urbain et les jeunes de moins de 18 ans représentent 45 %. Le taux brut de natalité était de 41,7 pour mille en 2007 avec un indice synthétique de fécondité de 4,4 en 2015. Selon le dernier rapport de développement humain, l'espérance de vie à la

naissance est de 62,3 ans. La structure de la population en 2018 par département est présentée dans le tableau ci-dessous.

Tableau I : Structure de la population par département en 2018

Départements	Population totale	Femmes en âge de procréer	Enfants de 0-11mois	Enfants de 0-59mois	Adolescents et jeunes 10-24 ans
**Kouilou**	129 398	25 880	5 176	25 880	38 819
**Niari**	325 442	65 088	13 018	65 089	97 633
**Lekoumou**	135 643	27 129	5 426	27 129	40 693
**Bouenza**	434 925	86 985	17 397	86 985	130 477
**Pool**	332 934	66 587	13 317	66 586	99 880
**Plateaux**	245 683	49 137	9 827	49 136	73 705
**Cuvette**	219 584	43 917	8 783	43 916	65 875
**Cuvette- ouest**	102 724	20 545	4 109	20 545	30 817
**Sangha**	120 650	24 130	4 826	24 130	36 195
**Likouala**	216 869	43 374	8 675	43 374	65 061
**Brazzaville**	1 932 610	386 522	77 304	386 522	579 783
**Pointe- Noire**	1 006 611	201 322	40 264	201 322	301 983
**Congo**	5 203 073	1 040 616	208 122	1 040 614	1 560 922

Source : INS, Perspectives démographiques

I.1.3 Contexte économique et social (PNDS 2018) Indicateurs

L'économie congolaise reste très peu diversifiée, car principalement axée sur l'industrie pétrolière (environ 60 % du PIB) et dans une moindre mesure sur le bois (5%) et l'agriculture (3%), la part des autres secteurs dans le PIB restant très marginale. Depuis 2014, l'économie éprouve des difficultés pour son équilibre macroéconomique à cause de la baisse des cours du pétrole. Pour la quatrième année consécutive, la croissance du PIB est en baisse. Cette situation se traduit par le déclin du PIB (-1,5 %) sur la période 2014-2016, les autres secteurs n'ayant pas véritablement pris le relais (3,3 %).

En 2016, la croissance du PIB avait déjà enregistré une contre-performance de -2,8%. La baisse de la production pétrolière expliquerait en grande partie cette contre-performance qui confirme la persistance de la conjoncture économique que traverse le pays depuis deux années. D'ailleurs, face à l'amplification de cette crise économique, le Congo a, à l'instar des autres Etats membres de la CEMAC, fini par faire appel au Fonds Monétaire International (FMI) avec lequel les discussions sont en cours en vue d'aboutir à la signature d'un programme économique et financier.

Selon le RNDH 2015, l'Indicateur de Développement Humain (IDH) était de 0,591 au Congo en 2014, poursuivant ainsi sa hausse amorcée depuis le début des années 2000. Ce qui plaçait le Congo à la 136ème position mondiale. En dépit de la situation économique et financière difficile, que traverse le

pays, le Congo demeure un pays à revenu intermédiaire de la tranche inférieure avec un PIB/habitant de 1 600 USD en 2016. Cette situation limite l'accès du pays aux sources de financements extérieures. A titre illustratif, le pays ne bénéficie plus de l'appui des fonds GAVI depuis le début de l'année 2018. La conséquence de cette sortie est que le pays devra acheter seul tous les vaccins et supporter les charges opérationnelles de la vaccination, ce qui représente un coût annuel de 5 000 000 000 F CFA, soit 8 821 500 dollars US.

I.1.4 Transports et communications (PNDS 2018)

Le réseau routier Congolais compte 20 925 km, de route en grande partie, fortement dégradée et seulement bitumé à moins de 10,0% (1976 km), y compris les voiries urbaines. Un effort important a été consenti par le gouvernement pour renforcer les infrastructures de transport ferroviaire à travers la réhabilitation et l'équipement en 2007. Le pays dispose de trois aéroports internationaux, situé à Brazzaville (ville capitale), Pointe-Noire (ville économique localisée dans la partie sud) et à Ollombo. Les voies navigables desservent en grande partie le nord du pays. Le transport maritime est essentiellement concentré autour du Port Autonome de Pointe-Noire (PAPN).

I.2 ANALYSE DU SYSTEME DE SANTE

I.2.1 Vision, Principes, Valeurs, But, Objectifs et Priorités de la PNS

- **Vision.**

Un Congo doté d'un système de santé performant, résilient et à même de garantir l'accès universel à des services de santé de qualité et un état de santé optimal pour soutenir durablement la croissance et le développement du pays.

- **Principes.**

Cette vision est guidée par neuf principes à savoir : un leadership fort, une redevabilité, une gestion axée sur les résultats, une efficacité et une efficience du système, la décentralisation, le respect des engagements internationaux notamment l'ODD n°3, la participation et appropriation communautaire, le partenariat, l'intersectorialité.

- **Valeurs.**

Cette vision est soutenue par les valeurs d'équité, de justice sociale, de solidarité nationale, de pérennité, d'éthique, de rigueur, de transparence et de démocratie.

- **But.**

Permettre à toute la population congolaise de vivre en bonne santé et promouvoir le bien-être de tous à tout âge.

- **Objectif Général.**

Améliorer le niveau de santé de la population congolaise.

- **Objectif spécifique.**

Assurer d'ici à 2030, un accès universel aux soins de santé intégrés, continus et centrés sur la personne.

- **Les Problèmes Prioritaires.**

Les principaux problèmes au fonctionnement du système sont liés à la gouvernance, à la réforme et la décentralisation, aux Ressources Humaines, au financement, aux médicaments et intrants spécifiques, aux infrastructures, équipements/matériels, laboratoires et nouvelles technologies, à l'information sanitaire, ainsi qu'à l'insuffisance qualitative et quantitative de l'offre de santé à travers le pays, à la sous-utilisation des soins et services de santé par la population, à un environnement peu favorable à l'amélioration de la santé de la population et, à une préparation et une gestion inadéquate des épidémies et des catastrophes.

I.2.2 Organisation du système de santé

a. Organisation administrative

Le décret n° 2018 - 268 du 2 juillet 2018, organise le ministère en charge de la santé en trois niveaux hiérarchiques : central, intermédiaire et périphérique ou opérationnel.

- **Niveau central**

Le rôle du niveau central est stratégique, normatif et régulateur. Il a également la responsabilité de la coordination de l'ensemble du secteur et de la mobilisation et l'allocation des ressources. Il est représenté par le cabinet du ministre en charge de la santé et de la population, les directions rattachées au cabinet, l'inspection générale de la santé et 3 directions générales.

En dehors des directions générales et des directions centrales, le ministère de la santé dispose des programmes spécifiques concernent divers domaines de santé prioritaires. A ce jour, on dénombre 11 programmes rattachés à l'unité de coordination des programmes et projets qui dépendent directement

du cabinet ce sont : le Programme élargi de vaccination, le Programme national de lutte contre le paludisme, le Programme national de lutte contre la tuberculose, le Programme national de lutte contre le VIH/SIDA, le Programme national de lutte contre l'onchocercose, le Programme national de lutte contre la lèpre, le pian et l'Ulcère de Buruli, le Programme national de lutte contre la trypanosomiase, le Programme national de lutte contre la schistosomiase, le Programme national de lutte contre les maladies diarrhéiques et le Programme national de lutte contre les maladies non transmissibles ;

- **Niveau intermédiaire**

Le niveau intermédiaire sera représenté par : (i) les 12 directions départementales de la santé (DDS), (ii) les 12 directions départementales de la population et (iii) les 12 inspections départementales de la santé et de la population. Ces dernières jouent respectivement le rôle d'appui technique aux districts sanitaires et de respect de la réglementation du secteur à ce niveau. Les territoires des départements sanitaires obéissent au découpage administratif du pays.

- **Niveau périphérique**

Il est représenté par le district sanitaire (DS). Le Congo avait opté pour le Système de santé intégré de district (SSID) depuis les années 90, avec l'expérimentation de la 1ère circonscription socio-sanitaire de Dolisie. Actuellement le pays est découpé en 51 districts sanitaires selon l'arrêté n°5369 du 02 août 2017. Chaque DS se subdivise en plusieurs aires de santé. Le DS est géré par une Equipe Cadre du District (ECD) composée de 5 à 8 personnes qui assument cinq fonctions principales (gestion des ressources, planification des activités, Formation/encadrement du personnel, supervision, approvisionnement en médicaments essentiels et génériques, recherche-action). La population bénéficiaire participe à la gestion du système de santé à travers les comités de gestion (COGES) et des comités de santé (COSA). Ce sont des organes de participation communautaire qui soutiennent le fonctionnement du système de santé au niveau périphérique.

Organigramme du Ministère de la Santé et de la Population

(Décret 2018-268 du 02 juillet 2018)

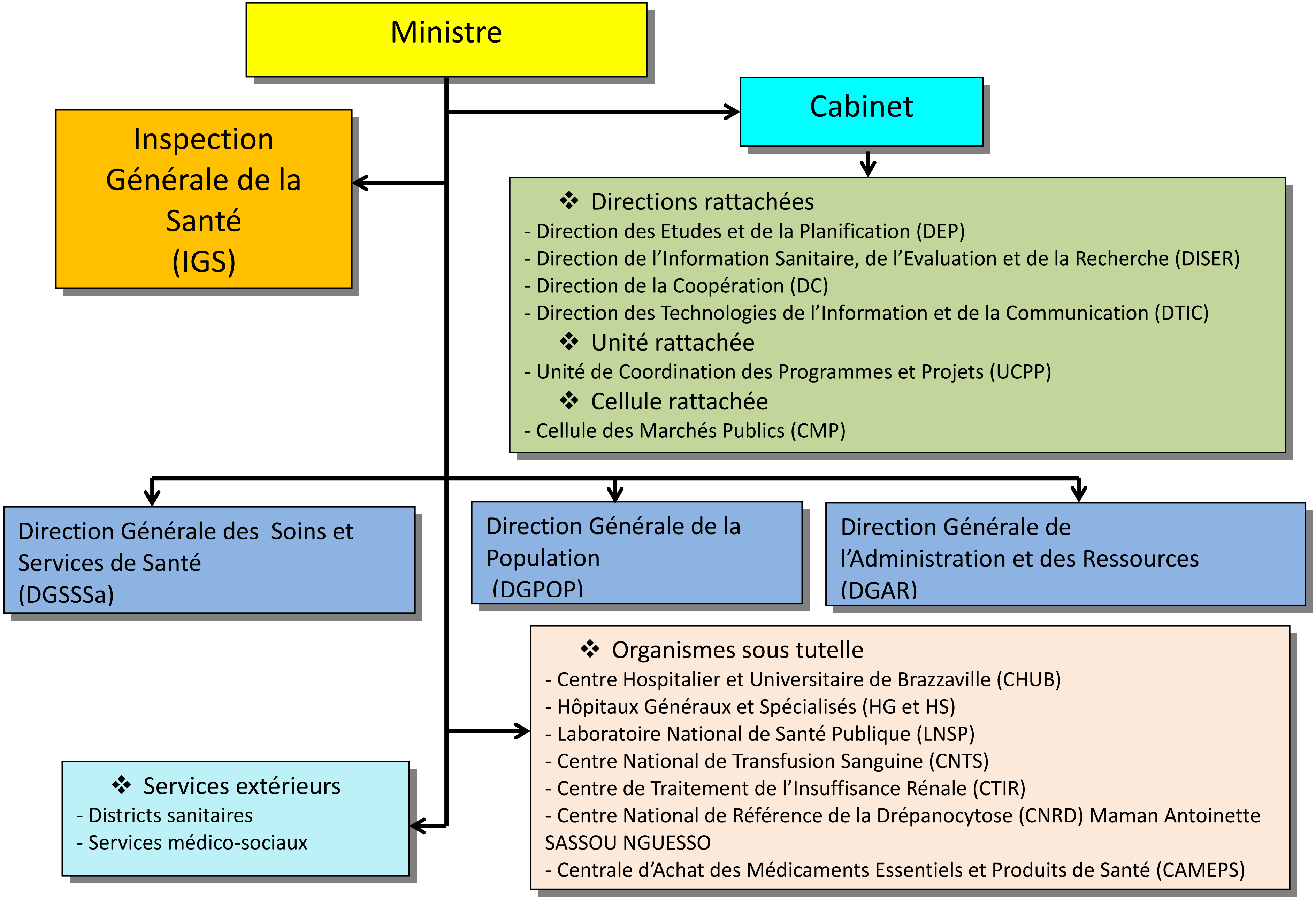

b. Organisation de l'offre des prestations de soins et services de santé

Le système de dispensation des soins et services de santé congolais est assuré par deux secteurs (public et privé). Ce système est structuré en trois échelons parmi lesquels les deux premiers constituent le district sanitaire et le 3ème échelon représente le deuxième niveau de recours.

⇨ Secteur public

Premier échelon

Le premier échelon est constitué par l'ensemble des Services de Santé de Premier Echelon (SSPE). C'est le niveau de premier contact entre la population et le système de santé. Les formations sanitaires du 1er échelon offrent un ensemble d'activités appelées « Paquet Minimum d'Activités (PMA) », dont le fondement principal reste les Soins de Santé Primaires (SSP) adoptés à Alma-Ata. Ces soins et services sont voulus intégrés, globaux, continus, accessibles (géographiquement, financièrement, culturellement) et acceptables par les bénéficiaires. D'après la carte sanitaire de 2015, le pays compte 337 CSI, dont 214 CSI à Paquet Minimum d'Activités Standard (PMAS), 96 CSI à Paquet Minimum d'activités Elargi de type1 et de type 2 et 8 centres de santé scolaires

Par ailleurs, pour des raisons de faible densité géographique, le pays dispose des formations sanitaires placées sous la responsabilité d'un agent de santé communautaire, appelées dispensaires ou postes de santé. On dénombre 280 dispensaires ou postes de santé. Il convient de noter que plusieurs DS n'étant pas encore développés, le nombre total d'aires de santé n'est pas connu.

En plus, d'après l'annuaire statistique sanitaire de 2012, il ressort que les ratios CSI/population sont très variables selon les départements. A titre d'exemple, dans les six (6) départements les plus peuplés, on trouve (1 CSI pour 46.360 habitants à Brazzaville, 1 CSI pour 24.844 habitants à Pointe-Noire, 1 CSI pour 14.463 habitants dans la Sangha, 1 CSI pour 16.589 habitants dans la Bouenza, 1 CSI pour 13.161 habitants dans la Cuvette et 1 CSI pour 16.544 habitants dans la Likouala).

Conformément à la politique nationale de santé à base communautaire adoptée par le pays, il est prévu un paquet de soins et services de santé qui seront offerts dans la communauté par des relais communautaires. C'est le cas de la PCIME-Communautaire (prise en charge intégrée des maladies du nouveau-né et de l'enfant communautaire), dont les outils sont en train d'être expérimentés dans quelques Districts sanitaires.

Deuxième échelon

Le deuxième échelon du système de santé congolais est constitué des 27 hôpitaux de district, appelé communément au Congo, «hôpitaux de Base » ou « hôpitaux de référence ». Cet échelon constitue le premier niveau de recours. Les Formations sanitaires du 2ème échelon sont souvent localisées dans les chefs-lieux de Départements ou des sous-préfectures. Le Kouilou est le seul département qui ne dispose d'aucun hôpital de district. Les hôpitaux de district sont gérés par des directions placées sous la tutelle administrative et technique des directions départementales de la santé (DDS). Ils ont pour missions d'offrir un ensemble d'activités appelées « Paquet Complémentaires d'Activités (PCA) » constitué de : hospitalisation, urgences médico-chirurgicales, urgences gynéco-obstétricales, urgences pédiatriques, explorations et examens paramédicaux (imagerie médicale et examens de laboratoire). Les dispositions légales précisent qu'un Hôpital Général situé dans un district sanitaire sans un premier niveau de référence joue aussi le rôle de 2ème échelon pour cette entité. C'est le cas des DS de Poto-poto, de Moungali, de Ouenzé à Brazzaville qui utilisent le CHUB, du DS Lumumba qui réfère, à l'hôpital général Adolphe Sicé et du DS de Loandjili qui utilise l'hôpital général de même nom à Pointe-Noire. Les 12 hôpitaux généraux départementaux en construction ne combleront malheureusement pas le gap en matière d'hôpitaux de district puisque localisés tous dans les chefs-lieux de département. Cette situation devra être réglée avec la transformation de certains CSI à PMAE en FOSA capables d'assurer l'offre du PCA.

Troisième échelon

Le niveau tertiaire de soins et services de santé au Congo est représenté par les 8 hôpitaux généraux, dont la fonction principale est d'offrir des soins et services spécialisés. Ces hôpitaux généraux dont le sommet est occupé par le Centre Hospitalier Universitaire de Brazzaville (CHUB) ne sont pas repartis de façon équitable, puisque localisés dans quatre départements avec 3 HG pour Brazzaville (CHUB, l'hôpital Central des armées et l'hôpital général Mère-Enfant Blanche GOMEZ), 2 HG pour Pointe-Noire (l'hôpital général Adolphe Sicé, et l'hôpital général de Loandjili) ; 1 HG dans le Niari (l'hôpital général de Dolisie), 2 HG dans la Cuvette (l'hôpital général 31 juillet Owando et l'hôpital général Edith Lucie Bongo Odimba).Dans le souci d'améliorer l'accès aux soins et services de santé spécialisés à travers le pays, le Gouvernement de la République a entrepris la construction des 12 nouveaux hôpitaux généraux départementaux dont le positionnement dans la pyramide reste à préciser par voie règlementaire. En plus des hôpitaux généraux, le troisième échelon est aussi constitué des établissements d'appui au diagnostic et au traitement. Il s'agit : (i) du Centre National de Transfusion Sanguine (CNTS), (ii) de la Centrale d'Achat des Médicaments Essentiels et des Produits de Santé (CAMEPS) , (iii) du Laboratoire National de Santé Publique (LNSP) avec un labo P3, (iv)du Centre

National de Référence de la Drépanocytose (CNRD) et (v) des deux Centres de Traitement Ambulatoire (CTA,) du VIH et les deux Centres antituberculeux

⇨ Secteur privé

Le secteur privé est en plein essor au Congo. Il est composé de : (i) polycliniques, (ii) cliniques, (iii) centres de santé confessionnels, (iv) cabinets médicaux, (v) officines, (vi) pharmacies et (vii) infirmeries. La plupart de ces structures ne sont pas homologuées et ne respectent pas les plans de découpage des districts sanitaires, donc de la carte sanitaire. Par conséquent ces formations sanitaires posent d'énormes problèmes de contrôle et de régulation. Aussi, ces structures sanitaires utilisent les mêmes agents évoluant dans le secteur public déstabilisant ainsi ce secteur. Les 2/3 de ces structures sanitaires privées sont concentrées dans les deux grandes villes du pays que sont Brazzaville et Pointe-Noire, au lieu de combler le déficit en milieu rural. En 2017, le pays comptait 54 cliniques (6,2%), 71 centres médico-sociaux (8,1%), 167 cabinets médicaux (19,2%), 19 cabinets dentaires (2,2%), 11 cabinets de kinésithérapie (1,3%) et 499 cabinets de soins infirmiers (57,2%).

Le secteur privé confessionnel : ce dernier participe en effet à l'offre de soins au niveau primaire.

Le secteur pharmaceutique privé occupe une place prépondérante dans le système de santé national. Le pays comptait 192 officines et 155 dépôts pharmaceutiques en 2015.

⇨ Médecine traditionnelle

Le pays compte 51 centres de médecine traditionnelle. L'intégration de la médecine traditionnelle dans le système de santé obéit aux recommandations de l'OMS. Elle permet de compléter l'offre de soins et d'en améliorer les performances.

c. Performance du système de santé :

⇨ Gouvernance, leadership et pilotage du système

Il existe un cadre institutionnel formel pour la régulation du système de santé. Toutefois, des multiples difficultés subsistent encore au sein de celui-ci. A titre illustratif, on note la caducité, l'inadéquation et l'absence de textes appropriés dans le secteur de la santé. C'est le cas de plusieurs textes régissant certains programmes de santé et du décret n° 69/290 du 21 juillet 1969, portant organisation du laboratoire national de santé publique).

Le district sanitaire qui est l'unité opérationnelle de mise en œuvre de toutes les politiques de santé, présente un déficit des documents normatifs de leur organisation et de fonctionnement. Cette situation ne favorise pas l'estimation des besoins et leur évaluation, en vue d'une meilleure planification.

Les capacités nationales du secteur en matière de planification stratégique et opérationnelle, du suivi et évaluation, de l'organisation des revues sectorielles ainsi que de la redevabilité restent encore faibles à tous les niveaux.

Du point de vue de la coordination des interventions sanitaires, le cadre de dialogue sectoriel, le Comité de coordination inter-agences (CCIA), les organes de mise en œuvre du PNDS (Comité de Pilotage du PNDS et Comité Technique de Suivi), n'ont pas fonctionné de façon optimale depuis 2011.

En sus de la loi sur la décentralisation, le ministère en charge de la santé et de la Population et celui en charge de la décentralisation ont signé un arrêté découpant le territoire en 52 districts sanitaires. Cependant la mise en œuvre effective de cette décentralisation qui confère la gestion des structures de base comme les centres de santé et les hôpitaux de district aux collectivités locales tarde à se faire à cause du déficit en textes d'application.

Sur le plan multisectoriel des interventions sanitaires, des progrès importants restent encore à accomplir par le pays, notamment dans le volet de la promotion de la santé à travers la mise en œuvre de l'approche « santé dans toutes les politiques » afin de vulgariser les comportements et attitudes favorables à la santé.

Sur le plan de la planification, plusieurs observations traduisent les limites et les faiblesses en la matière. A titre d'exemple le cycle de planification des plans nationaux de développement sanitaire (PNDS) n'a toujours pas été respecté. C'est ainsi que plusieurs périodes ont été gérées en l'absence d'un cadre de référence clair, partagé par l'ensemble des acteurs du secteur santé. Par ailleurs, plusieurs cadres stratégiques ont été mis en place, dictés par les spécificités de divers programmes, projets et autres interventions.

La réponse communautaire aux divers défis auxquels le pays est confronté dans le domaine de la santé, s'exprime à travers l'implication de trois entités distinctes : (i) les organes de participation communautaires : les Comités de santé (COSA) et Comité de gestion (COGES), (ii) les relais communautaires et (iii) les Organisations non gouvernementales (ONG).

Résumé des problèmes prioritaires retenus dans la Gouvernance, leadership et pilotage du système (PNDS 2018)

- Insuffisance dans la vulgarisation des textes, procédures et support de gestion du secteur de la santé à tous les niveaux ;
- Insuffisance de fonctionnement des organes de pilotage, de coordination et de partenariat à tous les niveaux ;
- Faibles capacités dans la planification, le suivi et l'évaluation à tous les niveaux du système ;
- Insuffisance des mécanismes de redevabilité dans le secteur de la santé ;
- Insuffisance de leadership à tous les niveaux du système de santé ;

- Retard dans la mise en œuvre de la décentralisation dans le secteur de la santé ;
- Insuffisance de l'implication de la communauté et des organisations de la société civile dans la gestion de la santé.

⇨ Le financement du système de santé

Selon les comptes nationaux de santé de 2012 à 2015, la dépense courante du Gouvernement par habitant est de 16.380 FCA (30 dollars US). Le taux d'exécution du budget de santé était de 32% en 2015. Ce faible taux d'exécution est tributaire à la complexité de la chaine de dépense publique.

La dépense totale en santé a été relativement constante. Rapportée au PIB, elle a évolué de 3% en 2012 à 4% en 2015. La dépense totale par habitant est estimée à 39.736 FCFA (72 dollars US). Au Congo, celle-ci, avait même dépassé la norme de dépense totale par habitant, recommandée aux états par la commission de macroéconomie et de santé pour l'atteinte des OMD (46 USD par habitant). Les sources de financement de la santé en 2015 étaient : (i) l'administration publique avec 46,50%, (ii) les ménages avec 31,25% et (iii) l'aide internationale (20, 75%).

Le Congo est sorti de l'éligibilité des fonds GAVI depuis le début de l'année 2018. La conséquence de cette sortie est que le pays devra acheter seul tous les vaccins et supporter les charges opérationnelles de la vaccination, ce qui représente un coût annuel de 5 000 000 000 F CFA, soit 8 821 500 dollars US.

Tenant compte de la DTS, le Congo devrait enregistrer des résultats meilleurs que ceux atteints dans le cadre des OMD. Le paiement direct par les ménages représente 32% des dépenses courantes en santé. La part du paiement directe dans la dépense totale de santé avait dépassé la limite supérieure (25%) au-delà de laquelle l'incidence des difficultés financières est qualifiée de catastrophique. Il existe des initiatives de gratuités en cours de mise en œuvre dans le pays, c'est le cas de la gratuité du traitement du paludisme, du Sida, de la tuberculose, de la césarienne et des autres interventions obstétricales majeures. A propos des sociétés d'assurance privées, on trouve quelques sociétés d'assurance privées (exemple : NSIA, AGC, Allianz etc.). Cependant, il n'existe pas encore des mécanismes de partages de risque (assurance maladie) destinés à couvrir l'ensemble de la population, en dépit de la loi n° 37-2014 du 27 juin 2014 instituant le régime d'assurance maladie universelle.

Le budget-programme est en cours d'introduction dans certains ministères dont celui de la santé. Globalement les achats continuent à se faire de façon passive. Cependant, le Congo en partenariat avec la Banque Mondiale met en œuvre l'approche de financement basé sur la performance (PBF) dans vingt un (21) districts sanitaires.

Le Congo fait partie des pays ayant des dépenses de santé par habitant les plus élevés d'Afrique centrale (72 USD par habitant), paradoxalement ses indicateurs de santé demeurent en deçà des niveaux souhaités. Il doit se poser plusieurs problèmes dans la distribution et dans l'utilisation efficiente

des fonds. Parmi ces problèmes, on peut citer : (i) l'inadéquation dans l'allocation des ressources (financières et humaines) entre les structures stratégiques et celles du niveau opérationnel du système. A titre illustratif, selon les comptes de la santé de 2012- 2015, les districts sanitaires qui offrent les Soins de Santé Primaires (SSP) ne bénéficient que de moins de 5% du budget du secteur santé. Tout comme les hôpitaux généraux et les structures administratives du niveau central absorbent à eux seules plus de 50% de ce budget.

Résumé des principaux problèmes retenus dans le financement du système de santé

- Insuffisance des ressources financières consacrées à la santé ;
- Inefficience dans l'affectation et l'utilisation des ressources financières disponibles ;
- L'assurance maladie universelle n'est pas encore opérationnelle ;
- Inexistence des mécanismes d'achat stratégique dans le secteur de la santé ;
- Faibles ressources consacrées au niveau opérationnel du système de santé ;
- La part des ménages dans le financement de la santé est très élevée.

⇨ **Les ressources humaines de santé**

Le Ministère de la Santé et de la Population ne dispose pas d'un cadre organique pour une gestion rationnelle et prévisionnelle des ressources humaines pour la santé. En ce qui concerne la mise en œuvre des ODD, l'OMS estime que la densité du personnel de santé doit être d'au moins 4,45 pour 1000 habitants pour que le pays dispose d'assez de ressources humaines pour offrir l'ensemble des paquets de service de santé requis. Avec une densité moyenne de 1,72 personnel de santé pour 1.000 habitants, le Congo ne dispose donc pas d'un effectif suffisant pour garantir l'atteinte de l'ODD n°3 d'ici 2030.

La répartition du personnel de santé sur le territoire national n'est pas équitable. En effet, la densité du personnel de santé est de 4,6 personnels de santé pour 1000 habitants à Pointe Noire, alors qu'elle varie entre 0,1 et 0,5 personnel de santé pour 1000 habitants en milieu rural.

En ce qui concerne la production des ressources humaines pour la santé, l'analyse de situation réalisée en décembre 2014 dans le cadre du Projet d'Appui au Développement des Ressources Humaines de Santé (PADHRS)-Formation initial a fait ressortir les problèmes suivant : (i) un déficit d'espaces formels de concertation entre les diverses instances impliquées dans la formation paramédicale et les instances chargées de l'utilisation des effectifs formés, (ii) une insuffisance des normes devant guider les mécanismes clés de régulation comme la détermination des effectifs et l'allocation des ressources et, (iii) une mobilisation de ressources peu alignée sur les besoins de formation

En plus, Bien que le gouvernement ait entrepris la formation en masse des médecins à l'extérieur (Cuba, Chine, Maroc, Russie, Turquie etc…), les capacités de production au niveau local demeurent encore insuffisantes pour espérer combler le déficit en vue de l'atteinte des ODD.

Tableau II : Différentes densités de certaines catégories du personnel pour 1.000 habitants

Départements	Population en 2017	Effectif En 2017	Densité du personnel pour 1000 habitants	Besoins en effectifs complémentaires*
**Kouilou**	120047	167	1,4	367
**Niari**	301790	85	0,3	1258
**Lékoumou**	125000	141	1,1	415
**Bouenza**	403330	31	0,1	1764
**Pool**	308730	231	0,7	1143
**Plateaux**	227836	164	0,7	850
**Cuvette**	203722	78	0,4	829
**Cuvette-Ouest**	95263	80	0,8	344
**Sangha**	111994	54	0,5	444
**Likouala**	201136	354	1,8	541
**Brazzaville**	1794372	2704	1,5	5281
**Pointe-Noire**	927861	4298	**4,6**	-169
**Congo**	**4 821 892**	**8 357**	**1,72**	**13 067**

Résumé des Problèmes prioritaires retenus au niveau des ressources humaines de santé

- Insuffisance des effectifs au niveau du secteur de la santé ;
- Répartition inéquitable des RHS sur le territoire national entre les milieux urbain et rural ;
- Inadéquation entre les besoins et la production des RHS ;
- Absence d'un cadre de concertation entre les différents ministères de la santé, de la fonction publique, des finances, les ministères des enseignements et les collectivités locales ;
- Absence d'un cadre organique pour la gestion prévisionnelle des RHS.

⇨ Le système national d'information sanitaire (SNIS)

En matière de SNIS, le Congo dispose d'un système d'information organisé autour des trois niveaux de la pyramide sanitaire. Cependant, à ce jour, le Congo ne dispose pas d'un plan stratégique du développement du SNIS. Malgré cette situation, il faut noter un certain nombre de forces au titre des quels on peut citer : (i) l'existence d'une direction de l'information sanitaire au sein du MSP, (ii) l'élaboration et la mise œuvre du plan stratégique de suivi et évaluation du secteur de la santé 2011-2015, (ii) existence des gestionnaires des bases des données au sein de chaque DS et (ii) la mise à disposition des outils de collecte des données standardisés dans tous les CSI et hôpitaux de district.

En ce qui concerne les hôpitaux généraux et les autres structures sous tutelles, des rapports non standardisés sont envoyés au cabinet.

Avec l'appui de la Banque Mondiale, le Congo a instauré le logiciel DHIS-2 dans 21 districts sanitaires sur un total de 52 que compte le pays.

Les faiblesses du SNIS peuvent être résumées comme suit : (i) non standardisation des outils de collecte, de compilation et d'analyse dans les hôpitaux généraux, les directions centrales, les institutions d'appui au diagnostic et la centrale d'achat, (ii) l'insuffisance des ressources matérielles, humaines, financières et de moyens dédiés à la gestion des données, (iii) la faible complétude et promptitude des rapports (iv) l'irrégularité des enquêtes explique l'insuffisance des données dans le secteur de la santé, (v) la fragmentation du système d'information due à l'existence des systèmes parallèles mis en place par certains programmes de santé et/ou certains partenaires au développement et, (vi) l'absence de mécanisme de capitalisation des données de la recherche en matière de santé, (vii) l'absence de l'institutionnalisation des études telles que les comptes de la santé, l'enquête SARA, etc., (viii) la qualité insuffisante des données est une préoccupation majeure, (ix) les décisions prises dans le secteur ne sont pas toujours fondées sur les bases factuelles générées par le SNIS.

S'agissant de la surveillance épidémiologique, on note : (i) l'existence d'une unité de coordination au niveau central , (ii) l'existence des structures de surveillance de routine au niveau national, intermédiaire et périphérique , (iii) la liste de maladies à potentiel épidémiques ou prioritaires avec définition de cas , (iv) la fiche de notification des cas disponibles dans les FOSA, (v) le secteur privée intégré dans la surveillance et (vi) l'implication de relais communautaires en cas d'épidémie et campagne de vaccination.

Parmi les problèmes relatifs à la surveillance épidémiologique, on peut citer : (i) l'absence de politique nationale et de plan stratégique pour la surveillance épidémiologique, (ii) au sein du MSP, coexistent plusieurs sous unités de surveillance épidémiologiques et peu coordonnées : PEV et Surveillance intégrée de la maladie et la riposte (SIMR) ; (iii) la faible qualité des données recueillies, (iv) la non exhaustivité de recueil de l'information, (v) l'insuffisance des équipements, matériels et logistiques à tous les niveaux du système.

Résumé des problèmes prioritaires retenus du SNIS

- Inexistence d'un plan stratégique de développement du SNIS ;
- Existence des circuits parallèles d'information sanitaire ;
- Non prise en compte des données du secteur privé dans le SNIS ;
- Le processus de prise des décisions non basée sur les données factuelles générées par le SNIS ;

- Les études comme les comptes nationaux de la santé et l'enquête SARA ne sont pas encore institutionnalisées ;
- Absence d'articulation entre les enquêtes et le processus de planification, suivi et évaluation ;
- Absence des mécanismes de capitalisation des données de la recherche en santé ;

⇨ **Médicaments, vaccins, produits sanguins et technologies**

Les médicaments sont distribués au Congo selon deux circuits : le circuit public qui approvisionne les formations sanitaires publiques et les formations sanitaires privées à but non lucratif et le circuit privé qui se charge des formations sanitaires du secteur privé.

Sur le plan institutionnel et juridique, le projet de loi pharmaceutique élaborée en 2009 n'a jamais abouti. L'absence de cette loi pharmaceutique laisse libre cours à toutes les pratiques y compris le commerce illicite et la circulation de faux médicaments.

S'agissant des centrales d'achat, le Congo a connu trois expériences à savoir : (i) la Centrale Nationale d'Achats de Médicaments, vaccins et consommables médicaux essentiels CENAMES créée par Décret n° 95-207 du 13 novembre 1995; (ii)la Congolaise de Médicaments Essentiels Génériques (COMEG) crée en 2005 sous forme d'ASBL ; (iii) la Centrale d'achats des Médicaments Essentiels et des Produits de Santé (CAMEPS) créée par loi n° 26-2015 du 29 octobre 2015 sous forme d'établissement public industriel et commercial (EPIC). Les deux premières centrales n'ont pas pu assurer l'acquisition, le stockage et la distribution des médicaments et autres consommables, avec pour conséquences les ruptures récurrentes dans les formations sanitaires (moins de 50%) de tous les échelons, ce qui a conduit le Gouvernement a créé la CAMEPS. Le prix d'achat des médicaments au Congo est supérieur de 70% par rapport au prix de référence international, et de 65%, par rapport au prix obtenu par la Centrale d'achat en 2007, en raison entre autres, du non-respect des procédures en matière d'approvisionnement (d'achat par appel d'offre). Le prix du médicament générique vendu par le centre de santé au patient représente le double de celui auquel il l'a été acheté. Au niveau de l'hôpital de base, il représente le triple du prix d'achat. La structure des prix est telle que le même médicament générique, arrivant au même prix au Congo, coûte plus chère dans une formation sanitaire publique que dans une officine de pharmacie privée, en raison du taux élevé de la marge cumulée dans le secteur public. C'est pourquoi le ministère de la santé et de la population en partenariat avec l'OMS ont élaboré les documents relatifs à la structure du prix et à la mise en place de la pharmacie hospitalière.

Sur le plan de la production, le Gouvernement avait créé le Laboratoire Pharmaceutique du Congo (LAPCO) en 1974 pour produire localement un certain nombre de molécules. Malheureusement, suite à des difficultés de fonctionnement ce laboratoire avait été privatisé et transformé en LAPHARCO, qui a également mis fin à ses activités pour des raisons fiscalo-douanières. Actuellement la production locale

est limitée aux solutés massifs (sérums glucosés isotoniques et salés), par le Laboratoire BIOCARE. Quant aux fluides et gaz médicaux, « Air liquide » est la seule société qui assure la vente dans la plupart des formations sanitaires.

En ce qui concerne le secteur pharmaceutique privé, il est composé de sept (07) grossistes-répartiteurs (SEP, LABOREX, UBIPHARM, PHARMACREDIT, ZENUPHA, MEDIPHARM, EDS PHARMA), de 155 officines privées de pharmacie ; de 3 fournisseurs de produits médicaux (EMS, BIOSERVICE, COMATEC). Le Congo est classé par l'OMS parmi les 30% des pays de la région africaine, dont la réglementation pharmaceutique accuse encore des faiblesses. Il est reconnu aussi l'absence d'un système d'assurance qualité des produits pharmaceutiques.

Résumé des problèmes prioritaires retenus au niveau des Médicaments, vaccins, produits sanguins et technologies

- Faible disponibilité des MEG dans les formations sanitaires ;
- Faible accessibilité financière de la population aux MEG ;
- Insuffisance des ressources financières dédiées à l'approvisionnement, au stockage et à la distribution des MEG ;
- Absence de laboratoire de contrôle de qualité des médicaments ;
- Insuffisance de la production locale des médicaments ;
- Vente illicite des médicaments et circulation des médicaments contrefaits ;
- Faible couverture du pays en structures de dispensation des produits sanguins sécurisés.

⇨ Infrastructures et équipements médico-sanitaires

La situation actuelle concernant les normes des infrastructures sanitaires n'est pas satisfaisante. Le Congo ne dispose pas des normes et standard en matière d'infrastructures sanitaires. En effet, un document de normes et standards a été élaboré en 2011 mais il n'est toujours pas validé. Les critères géographiques et démographiques d'implantation ne semblent pas être maitrisés. Il y a une inégale répartition des infrastructures de santé dans les départements et entre les districts sanitaires

Les infrastructures du niveau central sont souvent vieillissantes et nécessitent un plan de réhabilitation global qui doit prendre en compte les problèmes d'adduction d'eau et d'épuration des eaux usées.

Les Infrastructures du niveau intermédiaire et périphérique nécessitent des réhabilitations majeures

Tableau III : Situation globale des Hôpitaux de District

Département Sanitaire	Population	Nombre de DS	Nombre des DS qui disposent d'un Hôpital de District (HD)	Nombre d'HD non fonctionnels	Nombre de DS qui ne disposent pas d'HD
**Kouilou**	92 708	3	0	0	3
**Niari**	284 466	5	2	0	3
**Lékoumou**	118 588	2	1	0	1
**Bouenza**	380 177	5	4	0	1
**Pool**	291 007	7	5	1	2
**Plateaux**	214 756	4	3	0	1
**Cuvette**	192 028	3	3	0	0
**Cuvette-Ouest**	89 794	2	2	0	0
**Sangha**	105 565	2	1	0	1
**Likouala**	189 591	2	1	0	1
**Brazzaville**	1 691 369	9	4	0	5
**Pointe-Noire**	874 598	7	1	0	6
**Total**	**4 524 647**	**51**	**27**	**1**	**24**

Résumé des problèmes prioritaires retenus dans les Infrastructures et équipements médico-sanitaires

- Absence d'un organe de concertation entre les différents intervenants dans la construction des infrastructures sanitaires.
- Faible capacité de l'organe technique du Ministère de la santé en charge du suivi des projets de construction
- Absence des normes et procédures pour la construction des formations sanitaires.
- Absence d'une politique claire de développement des infrastructures sanitaires

⇨ **Prestations des soins et services (PNS 2018)**

Utilisation des services curatifs

Le taux d'utilisation des services de santé est relativement faible selon l'ECOM 2011. Ce taux d'utilisation des services de santé est limité à 24% où 0,24 consultation par habitant et par an.

Ceci veut dire qu'en moyenne un Congolais utilise les SS une fois tous les quatre ans. Le milieu urbain est favorisé par rapport au milieu rural. Ce taux est supérieur ou égal à 30% dans la Lékoumou, Cuvette, Pool et Likouala mais baisse de 17% dans la sangha, 19% dans la le Kouilou et 20% dans les plateaux.

Organisation des soins de santé primaires

Toutes les composantes des soins de santé primaires tant préventives, curatives et promotionnelles sont offertes à travers le Paquet Minimum et Paquets Complémentaires d'Activités respectivement dans les CSI et les Hôpitaux de district qui constituent les deux échelons des soins du DS.

Couverture sanitaire du pays.

Le Congo est subdivisé en 52 districts sanitaires. Il est desservi par un réseau de 682 formations publiques (280 postes de santé, 337 Centres de Santé Intégrés.

Il existe deux types des CSI : (i) les CSI à paquet minimum d'activités standards (PMAS) et, (ii) les CSI à paquet minimum d'activités élargi (PMAE).

La carte sanitaire nationale n'a pas à ce jour, déterminé le nombre d'aires sanitaires dont dispose le pays. Il n'est donc pas possible de dégager les besoins en termes de CSI.

Offre des prestations au premier échelon :

Le CSI, premier niveau de contact de la population avec le système de soin est le niveau de base du système. Le CSI à Paquet Minimum d'Activités Standards (PMAS) offrent les services de base incluant les activités de consultation curative, consultation prénatale, consultation préscolaire, les activités promotionnelles et le planning familial. Le CSI à Paquet Minimum d'Activités Elargi (PMAE) offre les examens de laboratoires, les services d'accouchement et de chirurgie.

L'évaluation de la situation sanitaire conduite en 2012 avait relevé qu'en dehors de la vaccination, les autres activités des CSI sont caractérisées par des faibles indicateurs de fréquentation.

Le rapport de la revue de 2018 souligne qu'en dehors des formations sanitaires des 21 districts sanitaires qui bénéficient de l'appui de la Banque Mondiale à travers le projet du Financement Basé sur la Performance (PBF) et ceux qui reçoivent le financement de GAVI-RSS, les autres districts sanitaires présentent des performances faibles en ce qui concernent l'offre des soins de santé primaires.

Offre des prestations dans les hôpitaux de district (HD).

Le pays compte 27 HD répartis dans 11 départements. Les HD offrent le Paquet complémentaire d'activités (PCA) qui comprend la prise en charge des patients référés par les centres de santé, prise en charge des urgences médico-chirurgicales de base, prise en charge des urgences néonatales, obstétricales et gynécologiques de base, hospitalisation dans les quatre spécialités de base qui sont ; la médecine, la chirurgie, la pédiatrie et la gynéco-obstétrique, la prise en charge des urgences stomatologies, oto-rhino-laryngologiques et maxillo-faciales de base, explorations biologiques et radiologiques de base et le stockage et la dispensation des médicaments et produits de santé essentiels. Il existe 27 HD pour un total de 52 districts sanitaires, soit un gap de 25 HD.

Parmi les dysfonctionnements notés au niveau des HD, on peut signaler : la faiblesse dans la gestion et l'administration des HD et la faible intégration des HD dans les activités de leurs districts sanitaires respectifs. De façon générale, les hôpitaux de base ne bénéficient pas d'appui technique des hôpitaux généraux d'une part et, d'autre part, ils n'apportent pas non plus systématiquement un appui technique aux structures de santé de 1er échelon. Le fonctionnement des hôpitaux de base est marqué par les

contraintes suivantes : (i) insuffisance et obsolescence des équipements médico techniques ; (ii) vétusté des infrastructures, (iii) insuffisance qualitative et quantitative du personnel et (iv) faible participation des médecins des HD aux activités des districts sanitaires.

Offre des soins et services spécialisés dans les hôpitaux généraux (HG) :
Le pays compte 7 HG fonctionnels qui sont repartis dans 4 départements (Brazzaville, Pointe-Noire, Niari, Cuvette). Douze autres HG sont en construction dont un hôpital par département. Le degré de fonctionnalité de ces hôpitaux n'est pas connu car aucune étude n'a été réalisée à ce sujet. Hormis les services d'urgences médico-chirurgicales, gynéco-obstétrique, pédiatrie clinique, stomatologie, radiologie conventionnelle et numérisée, échographie et panoramique dentaire qui sont disponibles dans les 7 hôpitaux, la disponibilité des autres services n'est pas satisfaisante. La qualité des soins offerts par les HG est préoccupante étant donné le délabrement de leurs plateaux techniques. Seul l'HG Lucie Mbongo Odimba qui est seul à disposer d'un équipement moderne de pointe, offre les soins de santé de qualité acceptable.
Actuellement l'offre de soins spécialisés au CHUB (service de réanimation, service d'imagerie médicale, unité neuro-vasculaire, unité d'hémodialyse et unité de drépanocytose) et HGAS dans de Pointe-Noire (unité d'hémodialyse) est confronté à plusieurs problèmes dont celui de vétusté de l'outil de travail évoqué plus haut.

I.3 ANALYSE DE LA SITUATION DES MTN

I.3.1 Situation épidémiologique et charge des maladies

Au nombre des Maladies Tropicales Négligées répertoriées par l'OMS, les suivantes sont connues endémiques au Congo :

- Les MTN à chimiothérapie préventive CTP : L'onchocercose, La filariose lymphatique, Les géo helminthiases, Les schistosomiases, et le trachome ;
- Les MTN à prise en charge des cas : La lèpre, L'Ulcère de Buruli, Le pian, La Trypanosomiase Humaine Africaine (THA),

La Loase fortement endémique au Congo influence énormément la stratégie du traitement de l'onchocercose et de la filariose lymphatique. A ce jour il n'existe aucun traitement adéquat. Les maladies telles que la Dengue, la rage, le chikungunya et la fièvre jaune sont considérées comme des maladies à potentiel épidémique au Congo.

LA SCHISTOSOMIASE

La schistosomiase ou Bilharziose est l'une des principales maladies transmissibles ayant des répercussions sanitaires majeures dans les pays en développement.

Au Congo, malgré des gros efforts réalisés dans la lutte contre cette endémie au cours des années 80, la schistosomiase reste encore un problème de santé publique. Elle affecte les départements du Kouilou, du Niari, de la Bouenza, de la Lékoumou, du Pool, de Brazzaville et de la Sangha.

Les résultats de la cartographie nationale réalisée en 2011 montrent que les districts de Madingo-kayes (Kouilou) et la commune de Nkayi (Bouenza) sont les plus touchés avec des prévalences de 58,8% et 41,4%. Une attention particulière devrait être accordée aussi au district de Komono (Lékoumou) avec une prévalence de 17,8 %. La schistosomiase urinaire à *Schistosoma Haematobium* est la plus rencontrée, mais quelques cas de la schistosomiase rectale à *Schistosoma Intercalatum* ont été trouvés dans la Sangha, la Cuvette-ouest, le Pool et le Niari.

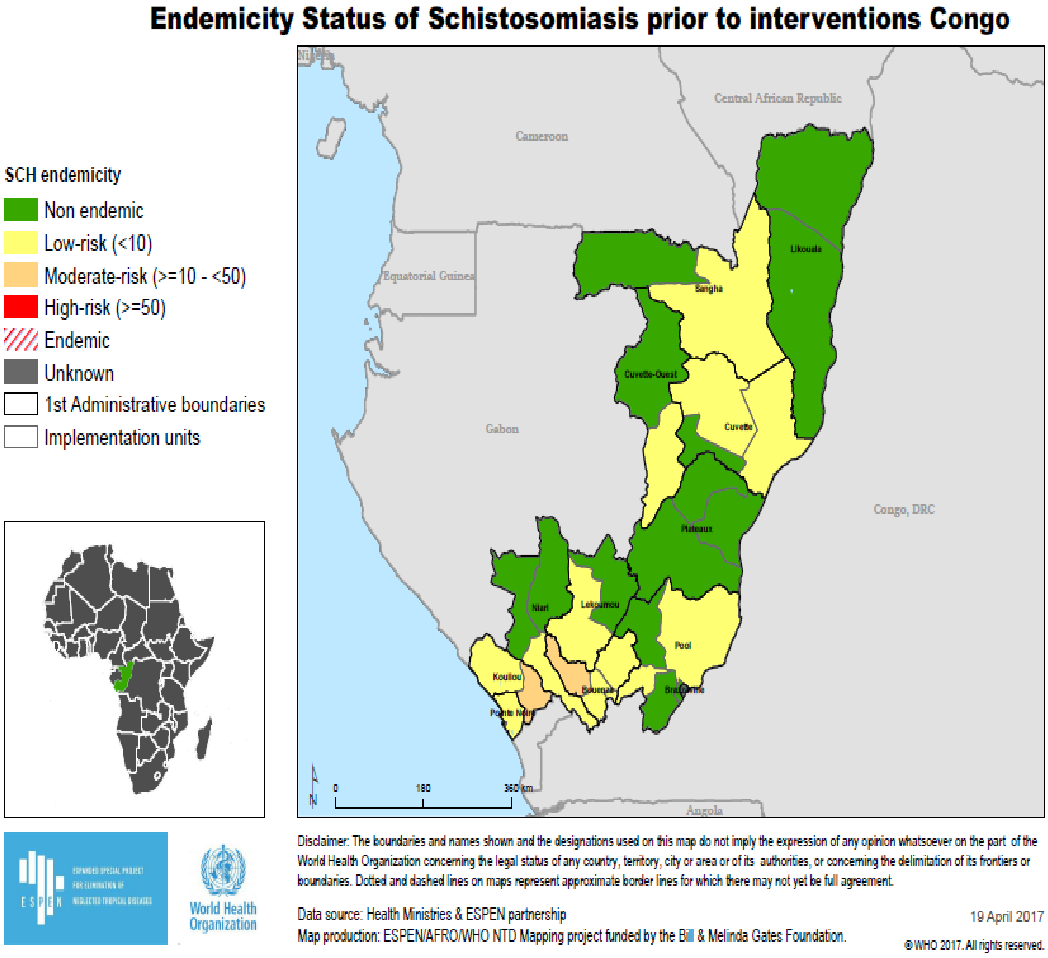

Figure 2 : Répartition des schistosomiases par district sanitaire selon la cartographie de 2011. (OMS cartographie 2011)

LES GEO-HELMINTHIASES

Les helminthiases intestinales sont des affections à majorité cosmopolites liées à la qualité de la vie. Elles jouent un rôle pathogène dans les pays en développement ou le niveau d'hygiène est en générale bas. De plus les affections n'ont jamais fait l'objet d'un plan concerté, comme c'est le cas de certaines endémies comme la lèpre, la trypanosomiase, les schistosomiases et l'onchocercose.

Le pays a réalisé une cartographie complète des géo helminthiases en 2011 de manière intégrée avec celle des schistosomiases.

Les résultats montrent, qu'en dehors des zones urbaines de Brazzaville et Pointe-Noire, et des districts sanitaires d'Owando, Oyo (Cuvette) et Abala (Plateaux) qui ont des prévalences inférieures à 20%, tous les autres districts sanitaires du pays avec des prévalences allant jusqu'au-delà de 50%

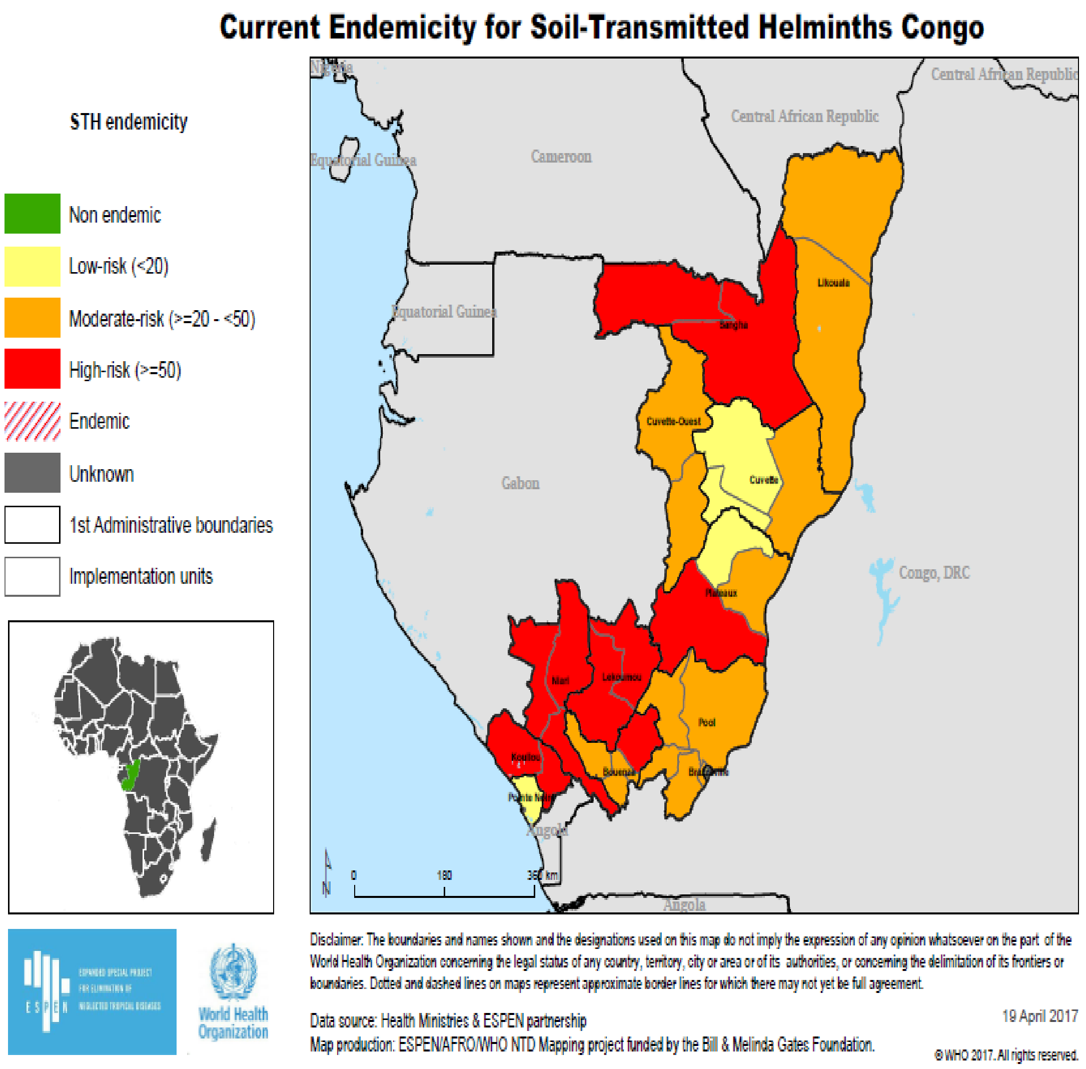

Figure 3 : Répartition des géohelminthisases par district sanitaire selon la cartographie de 2011

L'ONCHOCERCOSE

Au Congo les premiers cas d'onchocercose ont été notifiés par Le Bœuf en 1919 dans la vallée de la rivière Djoué (région de Brazzaville). Les différentes enquêtes épidémiologiques réalisées de 1978 à 2015 ont conduit à la maîtrise de la cartographie de l'onchocercose. La maladie sévit principalement dans le sud du pays où l'on distingue deux grands foyers :

- Le foyer du bassin du fleuve Congo et son affluent le Djoué qui touche les populations des départements de Brazzaville et du Pool ;
- Le foyer du bassin du fleuve Kouilou-Niari qui touche les populations des départements du Pool, de la Bouenza, de la Lékoumou, du Niari et du Kouilou.

Dans les communautés touchées, la maladie sévit en mode hypo, méso et hyper endémique. Les indices kystiques (IK) varient entre 0 et 67%. Chez les adultes, les taux de cécité de type1 et de type 2 sont respectivement de 1,7 et 3,4% en zone d'endémie onchocerquienne, contre 0,5 et 0,7 en zone non onchocerquienne. Environ 1169 280 personnes sont exposées à l'onchocercose dans ces deux grands foyers où 1144 communautés sont sous Traitement par Ivermectine sous Directives Communautaires (TIDC). A ce jour les départements de la Sangha, de la Cuvette-Ouest, de la Cuvette, des Plateaux, de Pointe-Noire et de la Likouala peuvent être considérées comme indemnes d'onchocercose.

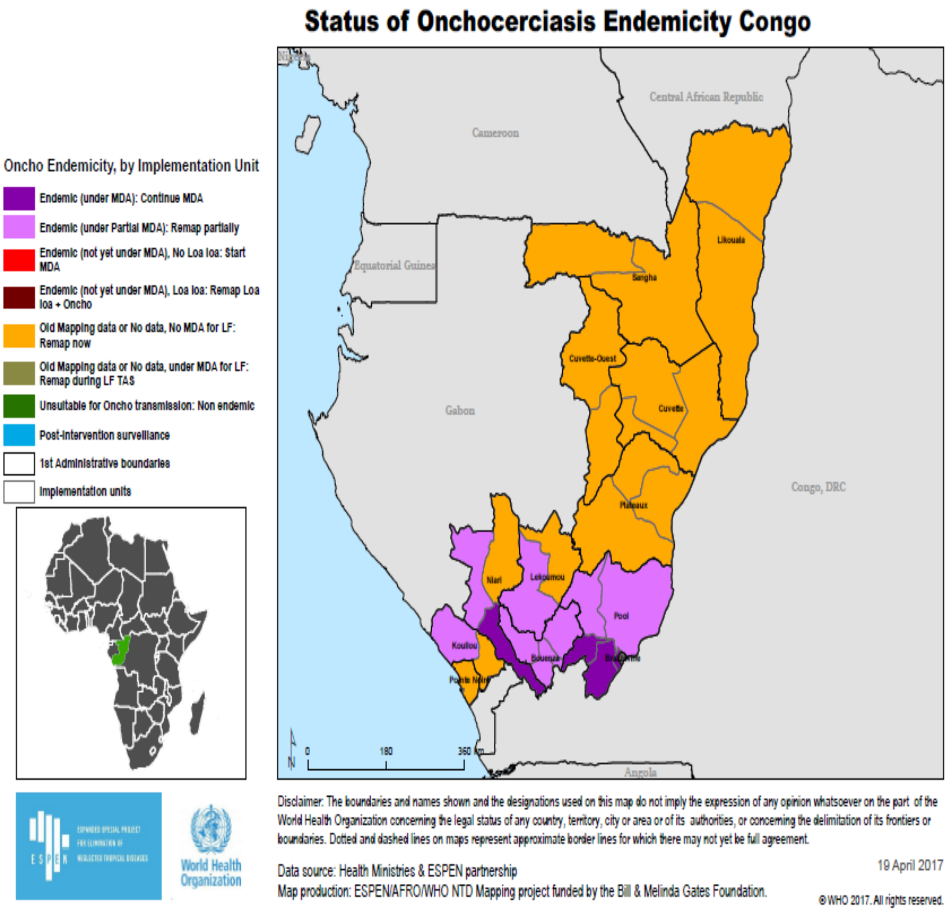

Figure 4 : Présentation du statut endémique d'onchocercose au Congo.

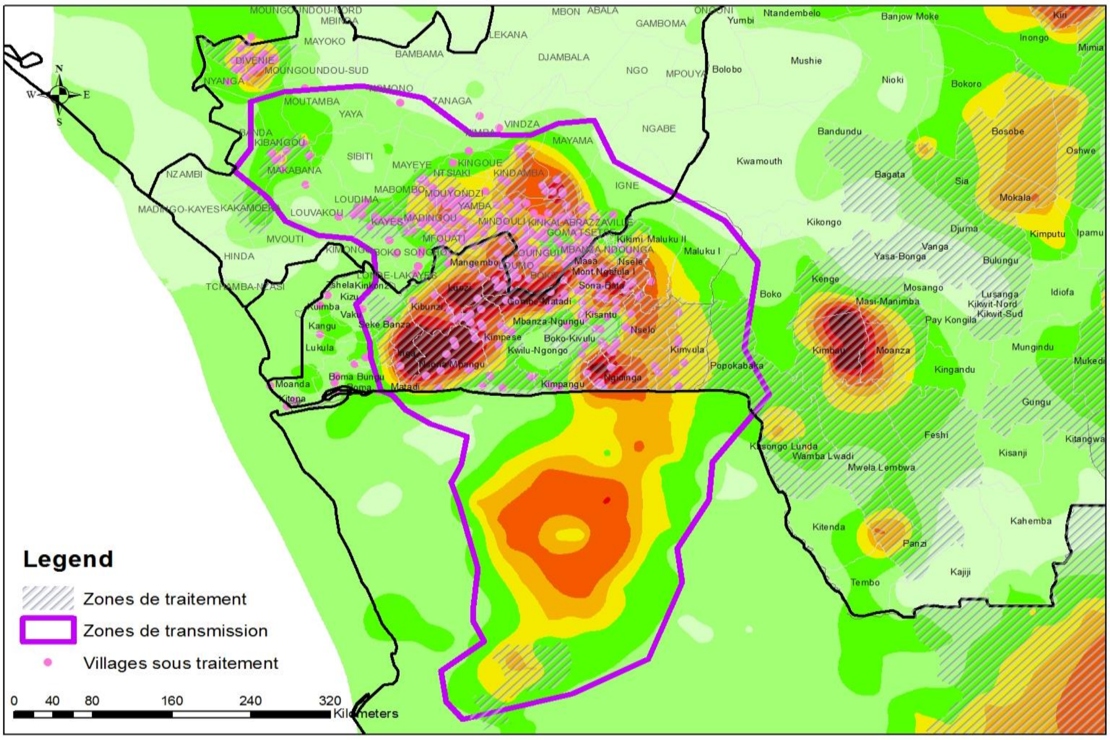

Figure 5 : Situation de la délimitation de la zone de traitement et de transmission de l'onchocercose

LA FILARIOSE LYMPHATIQUE

La filariose lymphatique (FL) est une maladie débilitante se traduisant, à la fois, par des effets directs de la filaire (obstruction mécanique, actions inflammatoires et allergisantes) et des effets indirects (portes ouvertes aux infections streptococciques et réactions secondaires scléro-fibreuses des tissus).

Elle est causée par de vers filiformes (*Wucherreriabancrofti, W. pacifica*, et *Brugiamalayi*) (1).

Au Congo, la collecte des données sur la filariose lymphatique ne faisait pas partie des maladies cibles sous surveillance épidémiologique dans les formations sanitaires, mais des cas d'éléphantiasis sont fréquemment rencontrés dans les villages et ceux d'hydrocèle font parfois l'objet d'une chirurgie esthétique dans les blocs opératoires.

L'enquête nationale sur la FL réalisée entre Décembre 2007 et Février 2008 a montré que la filariose lymphatique est endémique dans les départements de la Likouala (epena 1%.), la Sangha (ouesso 12%), la Cuvette Ouest (Ewo 2%, Kelle 1,2%.), le Pool (Ignie 4%.), la Bouenza (Mabombo 33,3%, Mfouati 1%), et le Niari (Kimongo 37%, Banda 22% Nianga 33%).

Toutefois, vue l'endémicité très focalisée de la FL au Congo, il a paru nécessaire de considérer le district sanitaire comme unité de mise en œuvre de la FL à la place du département. Cela a exigé ainsi un raffinage de la cartographie FL par district sanitaire en 2015. Depuis lors 578 communautés

(villages) sont bénéficiaires de la distribution semestrielle de masse d'Albendazole et 480 bénéficient de la distribution annuelle de masse d'Albendazole + ivermectine.

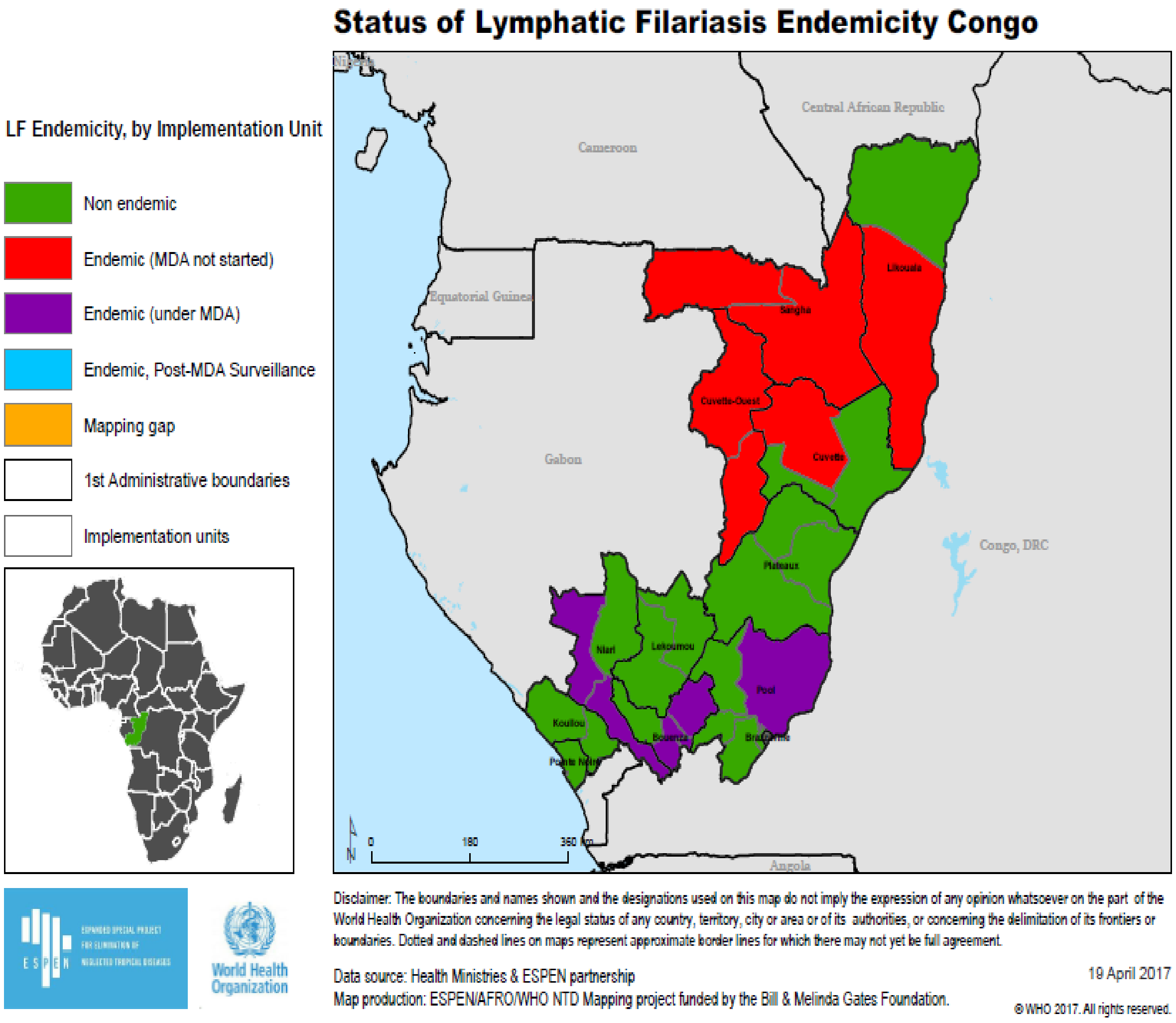

Figure 6 : Endémicité de la Filariose lymphatique au Congo selon la cartographie de 2015

LA LOASE

La loase est une filariose endémique en zone forestière d'Afrique de l'Ouest et centrale où la distribution est associée à celle de ses vecteurs *Chrysopssilacea* et *Chrysops dimidiata* (Fain, 1981). Les signes cliniques classiques de la loase sont la migration sous-conjonctivale des vers adultes, l'œdème de Calabar, le prurit et l'arthralgie.

L'intérêt pour cette filariose, longtemps considérée comme non pathogène comparée à d'autres espèces filariennes (Pinder, 1988) tient au fait que plusieurs rapports du Cameroun ont signalé que les fortes charges microfilariennes de *Loaloa* étaient associées aux troubles neurologiques (encéphalopathie) chez les individus ayant pris de l'Ivermectine contre l'onchocercose (Poitevin, 1996 ;

Chippaux et al. 1996 ; Gardon et al. 1997 ; Boussinesq et al, 1998). Le risque des effets secondaires sévères est une préoccupation majeure des programmes de traitement à l'Ivermectine dans les zones qui sont potentiellement endémiques de *Loa loa*.

Le risque des effets secondaires sévères suite au traitement à l'Ivermectine chez les individus infectés par *Loa Loa* est lié à l'intensité de l'infection : Il est significativement élevé quand la charge microfilarienne de *L. Loa* est supérieure à 8000 mf/ml. La sévérité des réactions devient évidente quand la charge microfilarienne est supérieure à 30.000 microfilaires/ml et le risque devient très grand pour les charges au-dessus de 50.000 microfilaires/ml (Chippaux *et al*. 1996 ; Gardon *et al*, 1997). Il devient absolument nécessaire que les programmes de distribution de l'Ivermectine s'assurent que les individus avec des telles fortes intensités de l'infection à *L. Loa* ne soient pas traitées ou que des mesures spéciales soient mises en place pour détecter rapidement les réactions secondaires sévères et gérer les cas correctement.

Les enquêtes de RAPLOA réalisées dans les départements de la Lékoumou, de la Bouenza, du Niari et du Kouilou (quatre départements sur douze) pour déterminer les zones de Co endémicité Loase - Onchocercose ont démontré que les zones forestières de ces départements sont fortement touchées par la Loase.

Tableau IV : Synthèse des résultats RAPLOA obtenus par zone (Enquête de validation du RAPLOA dans la Lékoumou, la Bouenza et le Niari en 2003)

**Zone**	**Nombre de personnes Interviewées**	**% RAPLOA**	**Taux de prévalence des Porteurs de mfl**	**% de sujets avec plus de 8000 mfl**	**% de sujets avec plus de 30000 mfl**
Forêt ou présomption de forte endémicité	933	83,5	29,8 %	9,1	**2,8**
Zone de transition ou d'incertitude	1369	45,7	15,2 %	4,2	1,6
Zone de savane ou présomption de faible endémicité	1021	17,3	6,9 %	2,3	0,9

mfl : microfilaires

Du fait du traitement à l'Ivermectine contre la filariose lymphatique dans les zones potentiellement endémiques et du risque des effets neurologiques de ce traitement aux individus ayant une charge importante de micro filaires de *Loa Loa*, il a été donc nécessaire qu'une enquête soit menée dans tous les départements du pays pour identifier les communautés touchées par la Loase. Aujourd'hui la cartographie de la Loase est complète au Congo.

Prévalence de la Loase (RAPLOA 2004/2010) au Congo

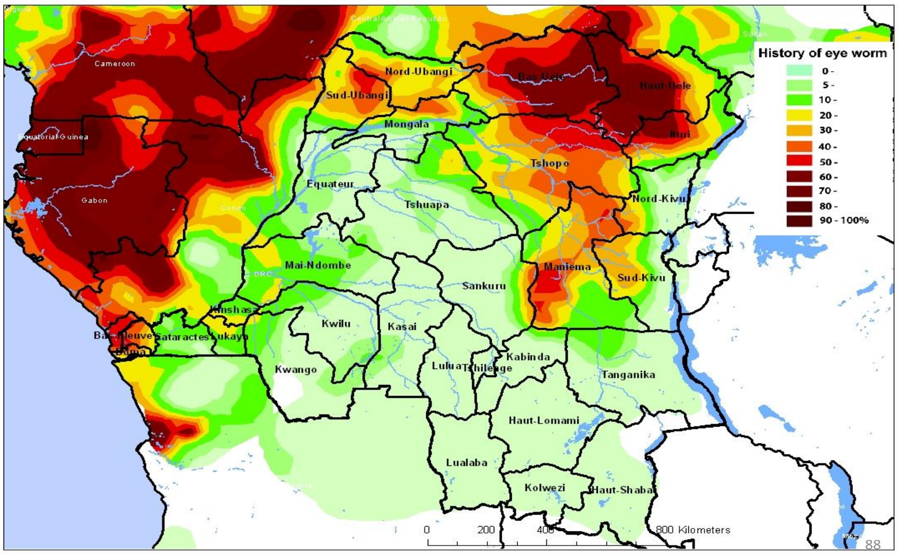

Figure 7 : Prévalence de la Loase au Congo (RAPLOA, 2004/2010)

Une cartographie nationale de cette maladie, intégrée à celle de la filariose lymphatique a été réalisée en 2015. Au cours de celle-ci, la méthode de la goutte épaisse calibrée a été utilisée montrant la charge microfilarienne à loa loa.

Tableau V : Résultats de la cartographie de la Loase au Congo, menée en 2015.

Départements	Districts sanitaires	Nombre personnes examinées	Nombre de cas positifs à la Loase	Taux Prévalence (%)	Charge maximale Loa loa
**Cuvette Ouest**	Etoumbi	122	44	36,1	47133
	Ewo	252	9	5,3	4540
**Sangha**	Ouesso	157	57	34,5	22567
	Sembé - Souanké	143	45	31,8	21833
**Pool**	Goma Tsé-Tsé	174	8	3,8	5430
	Mindouli	241	1	0,5	527
	Kindamba	183	5	2,6	3000
	Kinkala – Boko	226	2	0,9	545
**Plateaux**	Gamboma	194	5	2,4	1320
	Abala	169	25	14,4	6713
	Djambala	213	2	0,9	193
**Cuvette**	Owando	175	17	8,8	4547
	Oyo	138	0	0,0	0
	Mossaka-Loukoléla	165	2	0,8	1667
**Bouénza**	Loutété	155	1	0,6	680
	Nkayi-loudima	162	0	0,0	0
**Lékoumou**	Sibiti	72	14	19,4	13173
	Zanaga	90	18	20,2	17800
**Niari**	Mossendjo	140	27	19,3	0
**Kouilou**	Madingou-kayes	116	24	21,3	0
	Hinda-Mvouti	130	130	100,0	0
**Likouala**	Impfondo	180	2	1,1	500
	Bétou	169	5	2,8	793

Situation épidémiologique du trachome

Le trachome est une maladie oculaire et cécitante figurant parmi les maladies tropicales négligées à chimiothérapie préventive présente dans la région Africaine.

Pour éliminer cette maladie en Afrique et ailleurs dans le monde, l'OMS et tous les autres partenaires impliqués, recommandent la mise en œuvre de la stratégie « CHANCE ». Celle-ci implique quatre étapes : la cartographie, la prise en charge préventive et médicale, la surveillance des actions et la vérification de l'élimination.

Or, le trachome n'avait jamais été mentionné dans la littérature scientifique au Congo. Pourtant, ce pays est limitrophe d'un foyer actif de trachome de la République Centrafricaine. Un département, où un rapport de mission d'une équipe de Médecin du Monde a mentionné des cas de trachome actif, c'est essentiellement la Likouala.

Dans cette perspective de prévenir les incapacités visuelles, le Congo a franchi la première étape de la stratégie CHANCE par la réalisation de la cartographie dans trois districts sanitaires (Impfondo et Bétou dans la Likouala, et Ouesso dans la sangha).

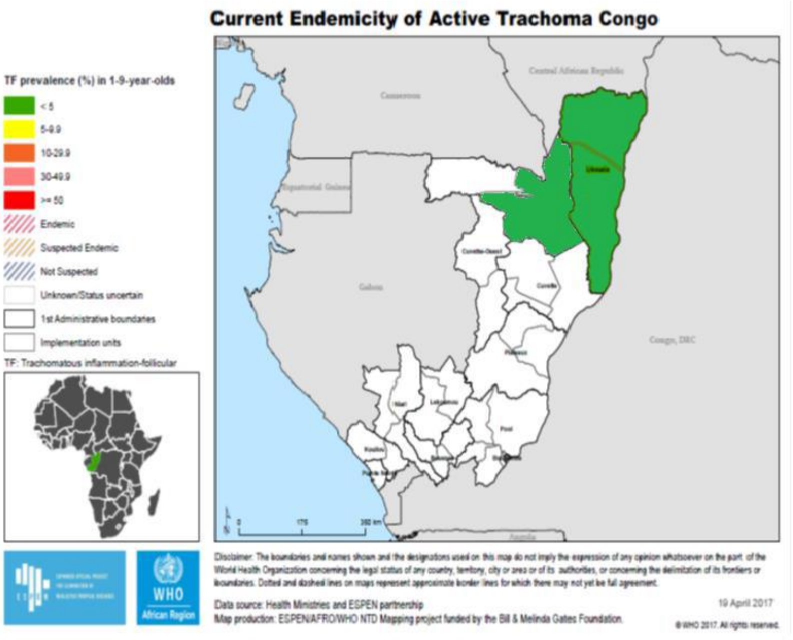

Figure 8 : Endémicité du trachome au Congo selon la cartographie de 2015

Tableau VI : Résultats de la cartographie du trachome au Congo, menée en 2015.

Départements	Districts sanitaires	Districts Administratifs	Population totale recensée	Pop totale examinée	Population adulte examinée	Nombre d'enfants examinés (1 à 9 ans)	Nombre de cas de trachome dépistés				Prévalence brute du trachome chez les enfants (1 à 9 ans)
							TF	TI	TT	Total	
**Sangha**	**Ouesso**	Mokéko	131	105	28	59	5	0	0	5	8,5
**Sangha**						54	1	0	0	1	1,9
**Sangha**			111	98	34	58	12	0	0	12	20,7
**Sangha**						55	0	0	0	0	0,0
**Sangha**			115	92	29	50	0	0	0	0	0,0
**Sangha**	**Total**		**357**	**295**	**91**	**276**	**18**	**0**	**0**	**18**	**6,5**
**Likouala**	**Impfondo**	Impfondo	106	95	36	54	0	0	0	0	0,0
**Likouala**			99	88	31	56	1	0	0	1	1,8
**Likouala**			110	93	27	53	1	0	0	1	1,9
**Likouala**			**315**	**276**	**94**	**163**	**2**	**0**	**0**	**2**	**1,2**
**Likouala**		Epéna	97	92	29	53	0	0	0	0	0,0
**Likouala**						55	0	0	0	0	0,0
**Likouala**						53	0	0	0	0	0,0
**Likouala**						54	0	0	0	0	0,0
**Likouala**			**97**	**92**		**215**	**0**	**0**	**0**	**0**	**0,0**
**Likouala**		Dongou	107	93	31	56	4	1	0	5	8,9
**Likouala**			119	101	32	58	0	0	0	0	0,0
**Likouala**			107	94	38	54	0	0	0	0	0,0
**Likouala**			96	93	38	53	1	0	0	1	1,9
**Likouala**			98	89	29	56	0	0	0	0	0,0
**Likouala**			**527**	**470**	**168**	**277**	**5**	**1**	**0**	**6**	**2,2**
**Likouala**	**Total DS Impfondo**		**939**	**838**	**262**	**655**	**7**	**1**	**0**	**8**	**1,2**
**Likouala**	**Bétou**	Betou	113	98	25	66	4	0	0	4	6,1
**Likouala**			118	105	30	67	3	1	0	4	6,0
**Likouala**			**231**	**203**	**55**	**133**	**7**	**1**	**0**	**8**	**6,0**
**Likouala**		Enyelle	119	103	38	58	1	0	0	1	1,7
**Likouala**			104	92	32	54	0	0	0	0	0,0
**Likouala**			112	91	33	52	0	0	0	0	0,0
**Likouala**			**335**	**286**	**103**	**164**	**1**	**0**	**0**	**1**	**0,6**
**Likouala**	**Total DS Bétou**		**566**	**489**	**158**	**297**	**8**	**1**	**0**	**9**	**3,0**
**Total**		**6**	**1862**	**1622**	**511**	**1228**	**33**	**2**	**0**	**35**	**2,9**

Situation épidémiologique de la THA.

La trypanosomiase humaine africaine (THA) ou maladie du sommeil est une maladie zoonotique négligée qui affecte les communautés rurales pauvres vivant dans les régions infestées par les mouches tsé-tsé en Afrique. Étant donné que cette maladie affecte également le bétail, entraînant la baisse de la productivité ou la mort, elle attaque non seulement la santé des personnes, mais également leurs moyens de vie. Cette maladie pose un problème majeur de santé publique, avec 100% de fatalité pour des cas non traités. Cinq des douze départements du pays restent endémiques avec des cas notifiés chaque année tant en passif qu'en actif avec le dépistage de masse assuré par l'équipe mobile. Les foyers du couloir sont ceux répertoriés tout au long du fleuve Congo comme notifié par la figure 9.

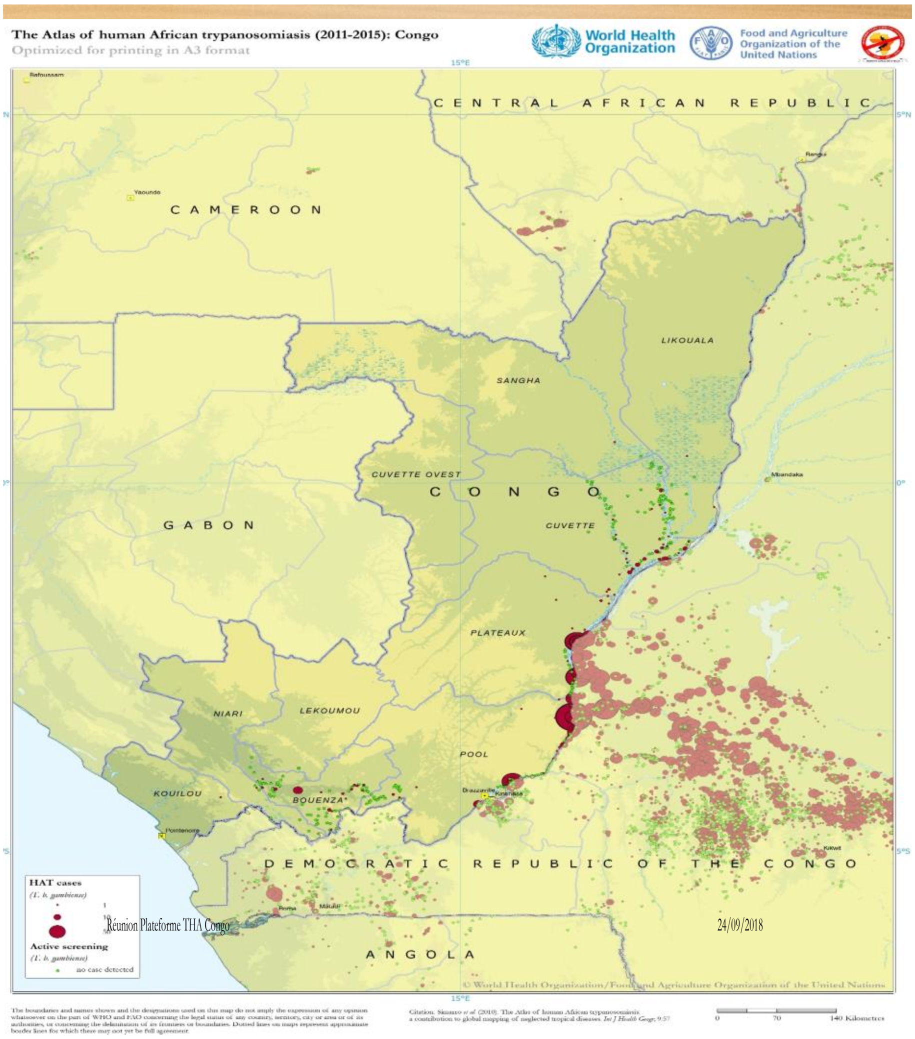

Figure 9 : La répartition des foyers de THA en République du Congo ces dix dernières années (Atlas OMS 2018)

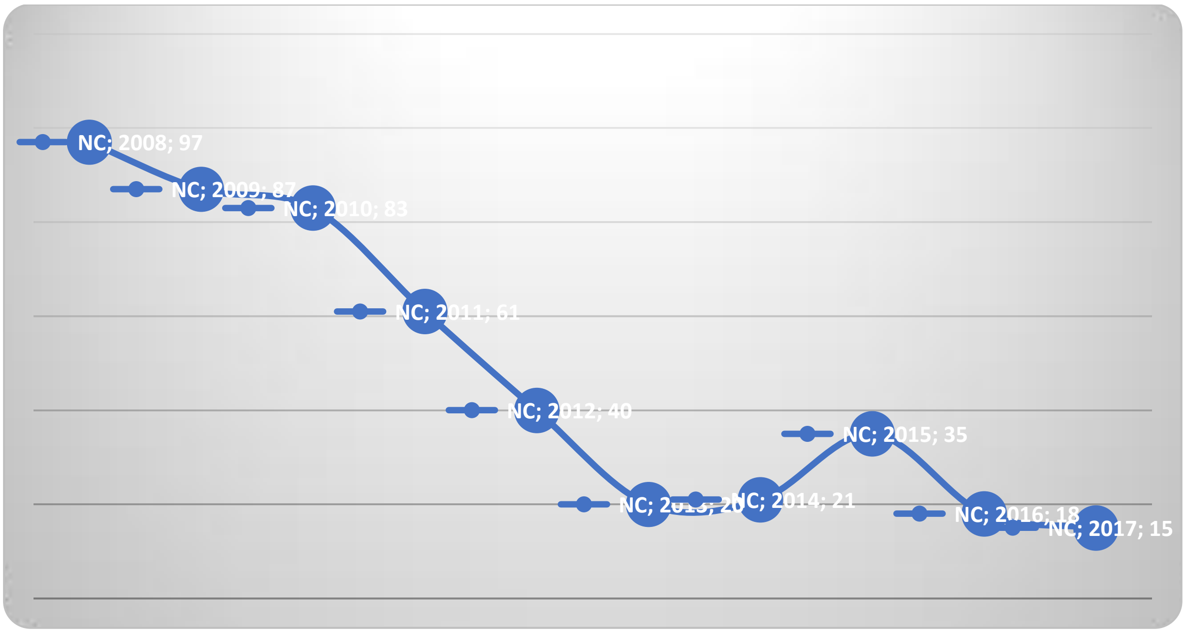

Graphique 1 : La répartition des cas de THA en République du Congo ces dix dernières années 2008-2017.

On note une baisse significative des cas de THA les dix dernières années grâce au renforcement de la surveillance passive et aux activités de terrain menées par l'équipe mobile du niveau central avec l'appui des agents évoluant dans les foyers endémiques prospectés. Cependant, la faible participation des populations aux activités de lutte contraste avec les résultats obtenus qui en fait devraient être corrélés à la population totale examinée par foyer et de façon annuelle.

Tableau VII : Répartition des foyers endémiques de THA selon les types d'endémicité dans les départements.

	Foyers endémiques	Types d'endémicité		Foyers sans données actuelles
		**élevé**	**faible**	
**Pool**	**Ngabé**	**X**		
	**Mindouli**			**X**
	**Kimbedi**	**X**		
**Plateaux**	**Mpouya**		**X**	
	**Gamboma**	**X**		
	**Makotimpoko**		**X**	
**Cuvette**	**Loukolela**	**X**		
	**Mossaka**	**X**		
**Bouénza**	**Yamba**	**X**		
	**Loudima**	**X**		
	**BKS**		**X**	
	**Mfouati**		**X**	
	**Nkayi**	**X**		
**Niari**	**Londelakaye**			**X**
	**Kimongo**			**X**
	**Dolisie**		**X**	

Situation épidémiologique des foyers notifiant des cas chaque année jusqu'en 2017.

Foyer de NGABE

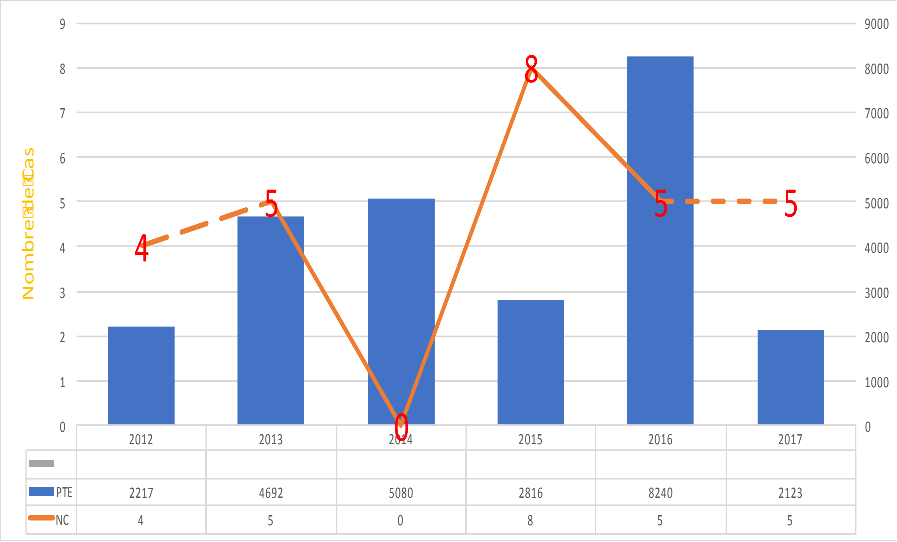

Graphique 2 : Situation épidémiologique du foyer de NGABE

La population totale examinée à NGABE reste variable d'une année à l'autre selon les stratégies développées. Le foyer reste actif avec des cas dépistés chaque année prospectée. Par ailleurs en 2014 le foyer n'a été prospecté pour des raisons financières ce qui justifie le chiffre 0, par contre en 2015 l'équipe mobile a dépisté 8 cas dans les aires à risque de Ngabé.

Foyer de MOSSAKA.

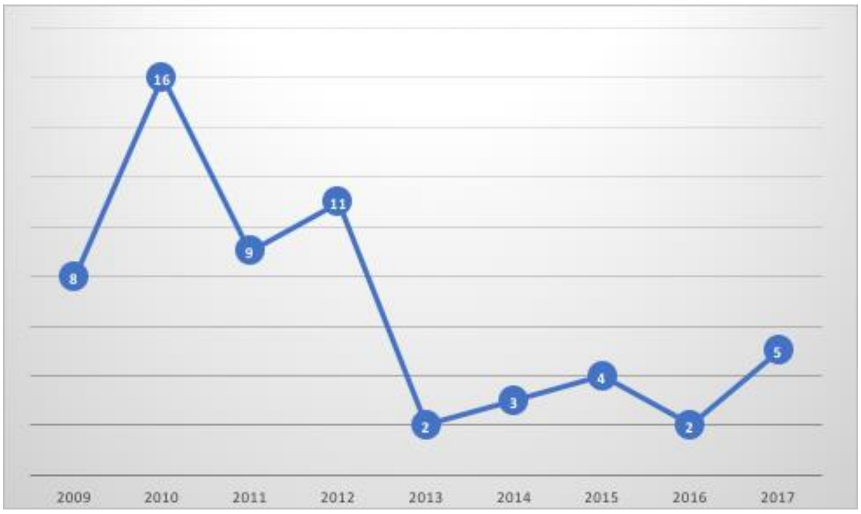

Graphique 3 : Situation épidémiologique du foyer de Mossaka

Le foyer de Mossaka est l'un des foyers actifs de THA au Congo où, chaque année des cas sont recensés. L'éloignement des villages et campements, les difficultés d'accès aux villages lointains et la faible participation des populations constituent les faiblesses dans cette localité, en plus des difficultés en moyens fluviaux..

Foyer de GAMBOMA.

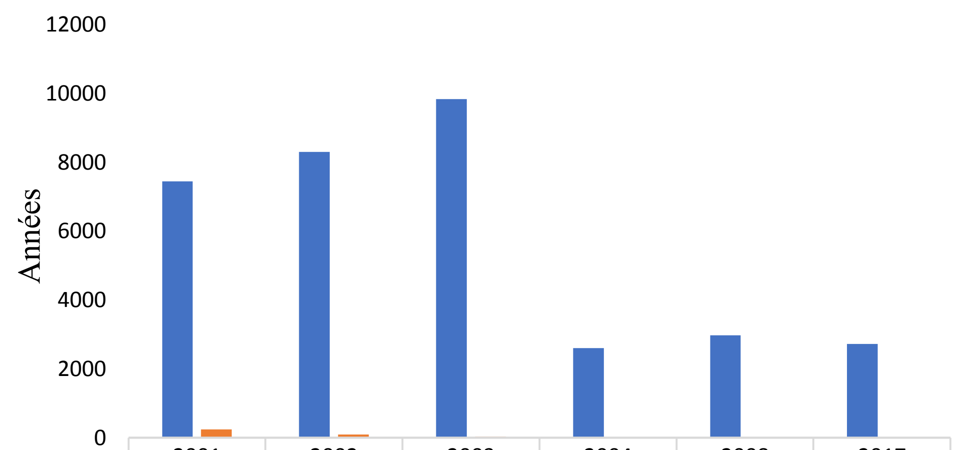

	2001	2002	2003	2004	2008	2017
Nr de personne examinées	7453	8310	9835	2603	2981	2725
Nr de Cas	242	95	29	10	3	4

Nr de personne examinées   Nr de Cas

Graphique 4 : Situation épidémiologique du foyer de Gamboma

Il y a une baisse significative des cas de THA dans le foyer de Gamboma où on note aussi des faibles taux de participation des populations aux activités de dépistage.

Foyer de LOUKOLELA

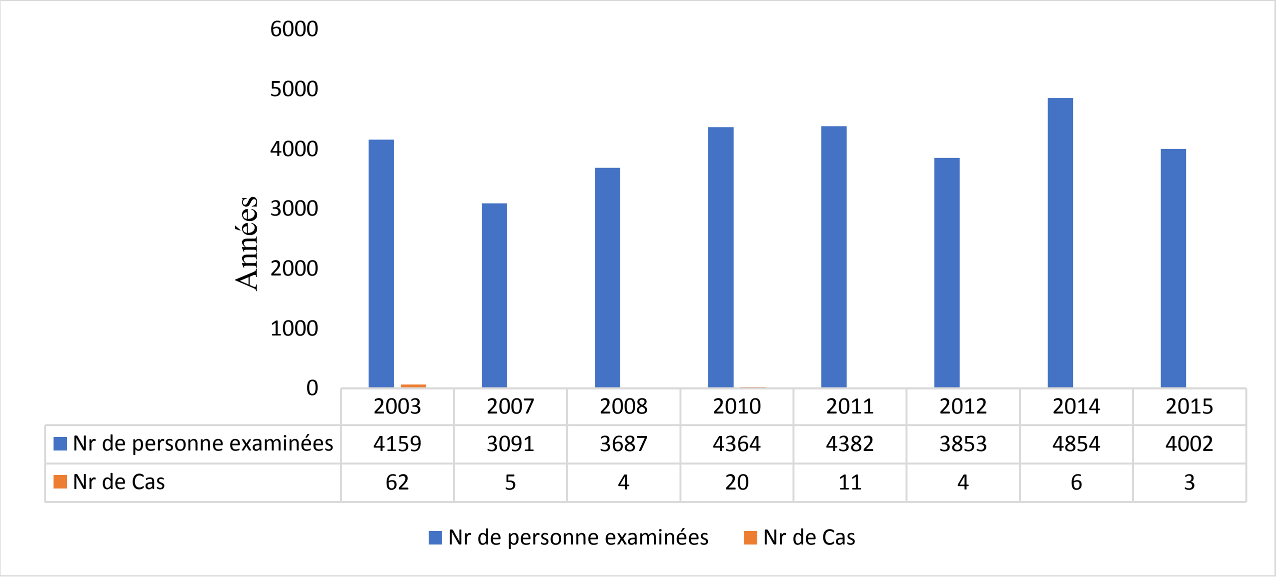

Graphique 5 : Situation épidémiologique du foyer de LOUKOLELA.

La situation actuelle de la THA dans le foyer de Loukolela paraît maitrisée. Le renforcement de la surveillance passive dans cette localité s'avère nécessaire.

Par ailleurs en 2017 un foyer sur trois a été prospecté pour des raisons financières, la faible participation des populations aux dépistages, les difficultés d'accès ce qui justifie le chiffre 0.

Tableau VIII : Dépistage et traitement de la THA durant cinq dernières années.

Année	Nbre de cas	Cas traités	Cas non traités	Taux de PEC(%)
2013	20	20	0	100
2014	21	21	0	100
2015	35	**35**	0	100
2016	18	18	0	100
2017	15	**15**	0	100
Total	109	109	0	100

Situation de la lèpre, l'ulcère de Buruli et le pian

Le Congo est placé parmi les pays Co-endémiques de ces trois affections dans la Région Africaine de l'OMS à l'instar de ses pays limitrophes que sont le Cameroun, l'Angola, la RDC, la RCA et le Gabon.

Suite à l'élaboration par l'OMS, d'un plan mondial d'élimination du pian et d'autres tréponématoses endémiques (Bejel en Afrique saharo sahélienne) et Pinta (en Amérique latine), en janvier 2007, le

Congo a pris l'engagement de mettre en place un programme combiné pour l'élimination durable de la lèpre, du pian et l'ulcère de Buruli.

La Lèpre

Après d'intenses activités de sensibilisation, de dépistage et de traitement qui ont eu lieu après l'introduction de la poly chimiothérapie (PCT) contre la lèpre en 1982, ont permis l'atteinte du seuil national d'élimination fixé par l'OMS de moins d'un cas pour 10.000 habitants en 2003. Cependant les poches d'endémicité demeurent dans les zones forestières et à climat tropical humide.

Cependant, depuis 2011, le programme a connu un relâchement progressif des activités par absence d'un soutien financier, ce qui a valu une réapparition significative des cas de lèpre, en particulier les cas multi bacillaires (64 cas) dans plusieurs départements du pays illustrant le dépistage tardif.

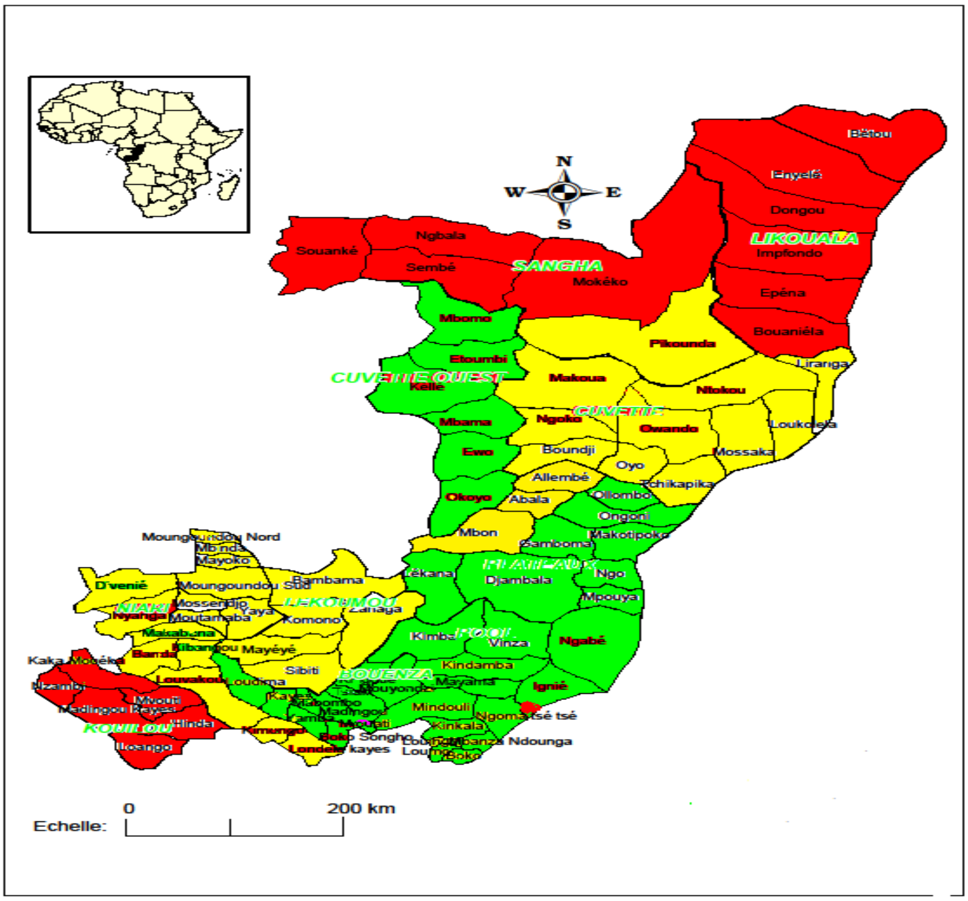

Figure 10 : Carte de l'endémicité de la Lèpre au Congo 2016

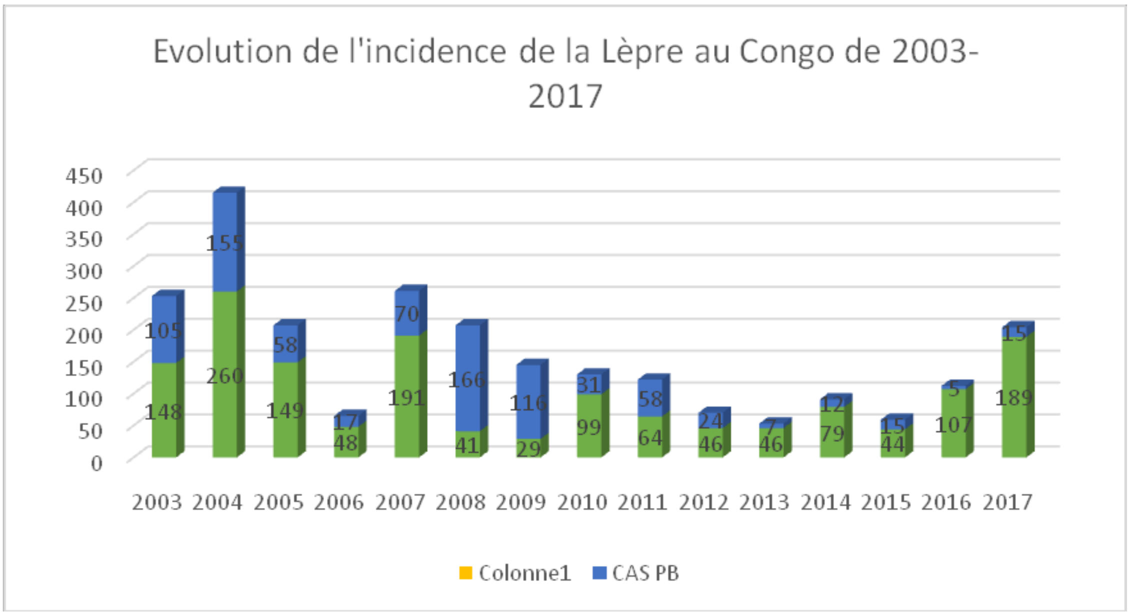

Graphique 6 : Evolution de l'incidence de la Lèpre au Congo de 2003-2017

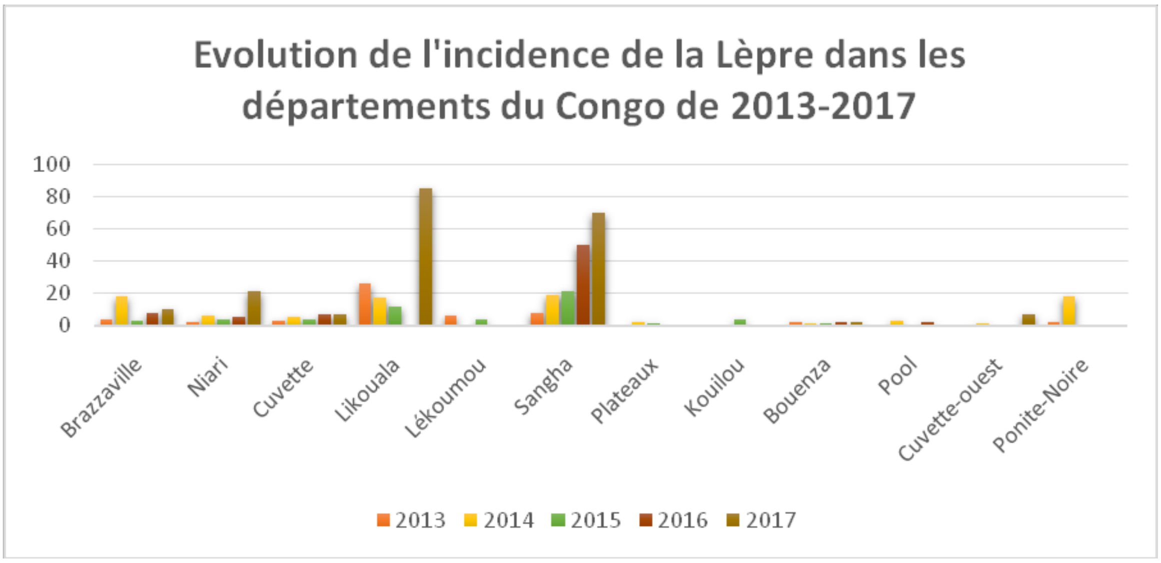

Graphique 7 : Evolution de l'incidence de la Lèpre dans les départements du Congo de 2013-2017

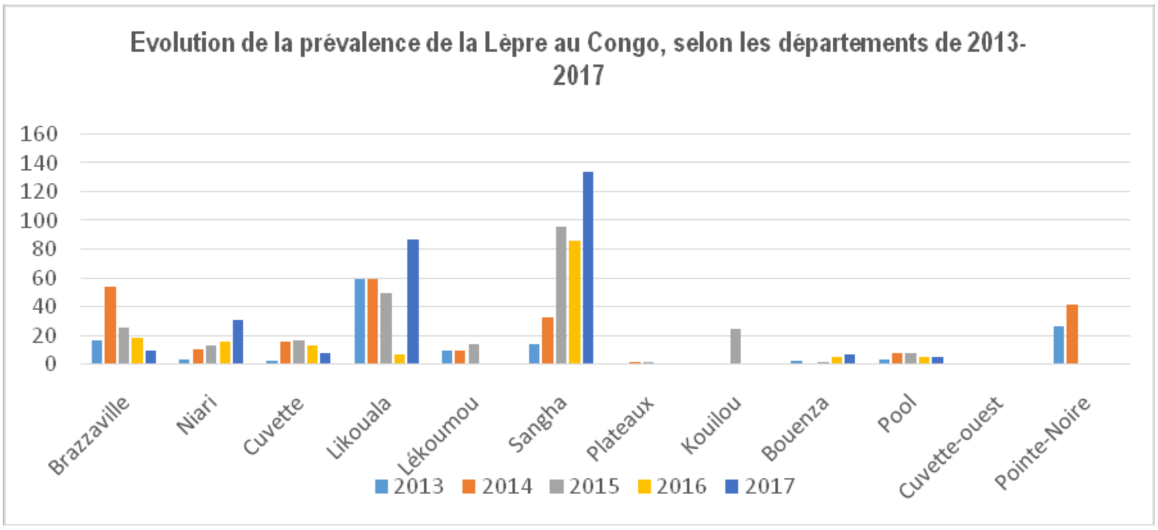

Graphique 8 : Evolution de la prévalence de la Lèpre au Congo, selon les départements de 2013-2017

L'Ulcère de Buruli

Les premiers cas d'Ulcère de Buruli sont dépistés en 2001, avec une moyenne de cent (100) cas par an. Le pic est atteint en 2006 avec un peu moins de 400 cas. C'est alors que débute la lutte contre l'ulcère de Buruli au Congo, intégrée à celle de la Lèpre. Il s'en est suivi une réduction progressive de l'incidence jusqu'à 100 cas an en 2011, puis un arrêt des activités de terrain (dépistage actif, supervision, recyclage du personnel soignant, etc…). De cet arrêt des activités, il en résulte une limitation au dénombrement passif, une sous notification des cas et une perte de la maîtrise de l'épidémiologie réelle de cette endémie, bien que les poches d'endémicité soient bien connues. Le dépistage tardif qui en découle, se traduit par une forte proportion des formes ulcérées, notamment dans la catégorie II (57,1 %) et dans la catégorie III (28,6%).

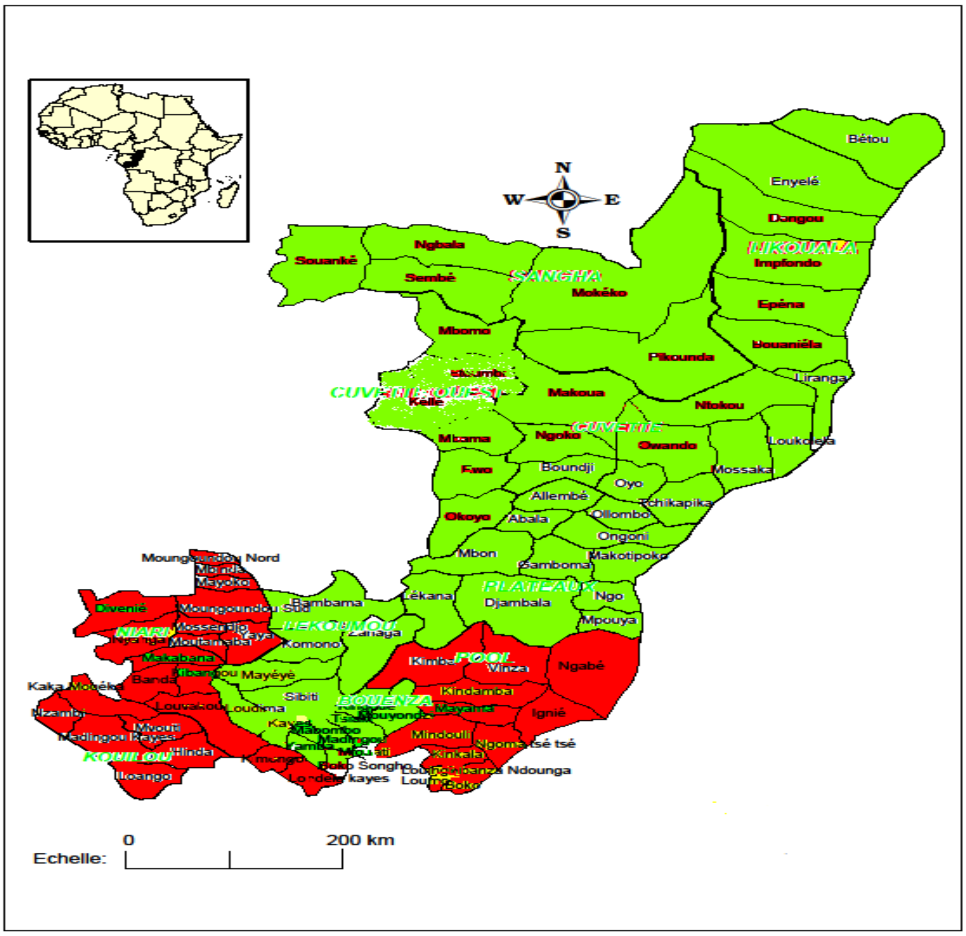

Figure 11 : Carte de l'endémicité de l'ulcère de Buruli au Congo en 2016

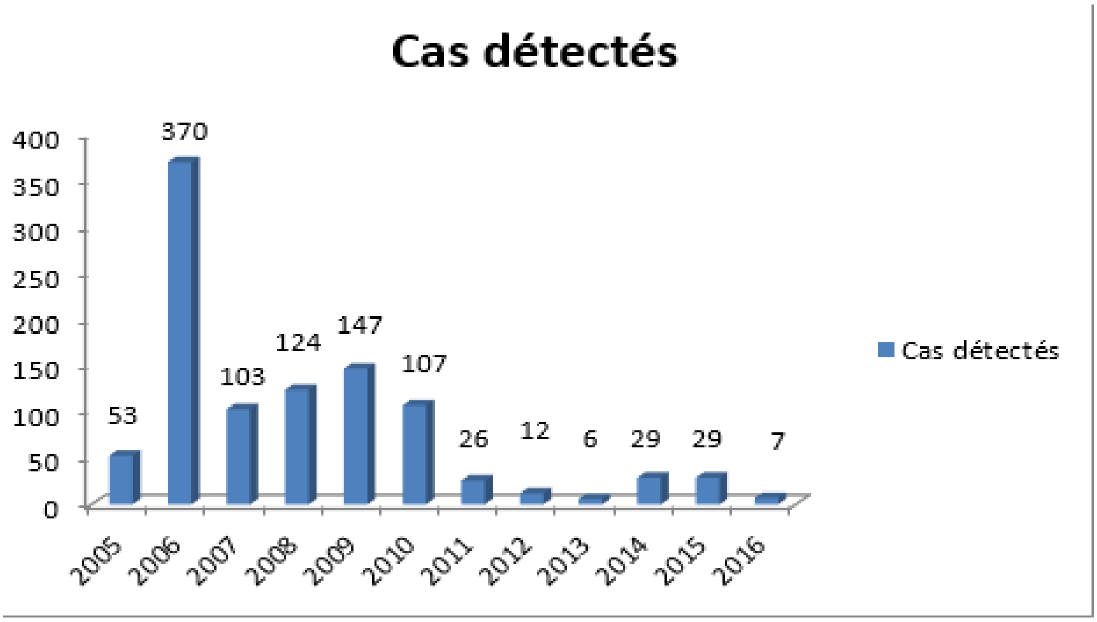

Graphique 9 : Incidence des cas d'ulcère de Buruli au Congo de 2006 à 2016

Tableau IX : Evolution des cas d'ulcère de Buruli au Congo par départements de 2013 à 2017

Départements	Années				
	2013	2014	2015	2016	2017
Kouilou	-	12	-	-	-
Pointe-Noire	-	06	-	-	-
Niari	06	06	12	07	13
Pool	-	-	06	-	-
Brazzaville	-	5	11	-	-
**Total**	**06**	**29**	**29**	**07**	**13**

Le Pian

Le pian est endémique au Congo dans les départements forestiers à climat chaud et humide de la Likouala, la Lékoumou et la Sangha ; les populations les plus touchées sont les minorités ethniques (notamment les peuples autochtones) qui constituent près du 1/3 de la population de ces départements où près de 65% des habitants sont atteints.

Tableau X : Evolution des cas de Pian au Congo par départements de 2013 à 2017

Départements	Années				
	2013	2014	2015	2016	2017
Likouala	-	40	06	-	-
Sangha	-	-	04	08	-
Lékoumou	-	-	-	-	-
**Total**	**-**	**40**	**10**	**08**	**-**

LES AUTRES MALADIES TROPICALES NEGLIGEES A POTENTIEL EPIDEMIQUE

LE CHIKUNGUNYA

Après la première épidémie de Chikungunya en 2010-2011 qui a touché les départements de Brazzaville et du Pool, avec environ 10.000 cas enregistrés par les services de santé, quelques cas sont régulièrement notifiés dans le pays.

LA RAGE

Les morsures de chiens sont régulièrement enregistrées au cours de ces 5 dernières années, particulièrement dans les départements de Pointe-Noire, Kouilou, Niari, Lékoumou et Bouenza.

En 2018 de la semaine 1 à la semaine 41,122 cas de morsures de chiens ont été enregistrés dont : 5 cas dans la Bouenza, 1 dans la Lékoumou, 11 dans le Niari, 1 dans les Plateaux, 101 à Pointe-Noire, 1 dans le Pool et 2 dans la Sangha. ;

LA DENGUE ET LA FIEVRE JAUNE

Les données sur la dengue sont peu documentées

En 2018 de la semaine 1 à la semaine 41, 292 cas ont été enregistrées dans les douze départements du pays allant de 7cas (Cuvette) à 110 cas à Pointe-Noire

Co endémicité des MTN

Tableau XI : La Co endémicité des MTN et la situation de la cartographie se résument comme suit

Département	District sanitaire	LF	Oncho	Schisto	GH	Loase	THA	Lèpre	UB	Pian
**Niari**	Dolisie	1	2	0	1	1	0	1	1	0
	Kibangou	1	2	0	1	1	0	1	0	0
	Mossendjo	0	2	0	1	1	0	1	0	0
**Bouenza**	Loudima	0	0	1	1	1	1	1	2	0
	Madingou	1	1	0	1	1	1	1	2	0
	Mouyondzi	1	1	1	1	1	0	1	2	0
	Loutété	1	1	1	1	1	1	1	2	0
**Pool**	Kinkala-Boko	0	1	0	1	1	0	1	0	0
	Mindouli	0	1	0	1	1	1	1	0	0
	Kindamba	0	1	0	1	1	0	1	1	0
	Goma tsétsé	0	1	0	1	1	0	1	0	0
	Ignié	1	0	0	1	1	1	1	0	0
**Cuvette Ouest**	Ewo	1	0	0	1	1	0	0	0	0
	Etoumbi	1	0	0	1	1	2	2	0	0
**Sangha**	Sembé-Souanké	1	0	0	1	1	0	1	0	1
	Ouesso	1	0	0	1	1	0	1	0	1
**Likouala**	Impfondo	1	0	0	1	1	0	1	2	1
	Bétou	0	0	0	1	1	0	1	2	1
**Kouilou**	Madingo Kayes	0	2	1	1	1	0	1	1	0
	Hinda-Mvouti	0	2	1	1	1	0	1	1	0
**Lékoumou**	Sibiti	0	0	1	1	1	0	1	0	2
	Zanaga	0	0	0	1	1	0	1	0	2
**Plateaux**	Djambala-Lékana	0	0	0	1	1	0	0	0	0
	Gamboma	0	0	0	1	1	1	0	0	0
	Abala	0	0	0	1	1	0	0	0	0
**Cuvette**	Mossaka-Loukoléla	0	0	0	1	1	1	1	1	0
	Owando	1	0	0	1	1	2	1	0	0
	Oyo	0	0	0	1	1	2	1	0	0
**Pointe-Noire**	Lumbuma	0	0	0	1	0	0	1	0	0
	Mvoumvou	0	0	0	1	0	0	1	0	0
	TiéTié	0	0	0	1	0	0	1	0	0
	Loandjili	0	0	0	1	0	0	1	0	0
	Ngoyo	0	0	0	1	0	0	1	0	0
	Mongo Poukou	0	0	0	1	0	0	1	0	0
**Brazzaville**	Makélékélé	0	1	0	1	0	0	1	0	0
	Bacongo	0	1	0	1	0	0	1	0	0
	Poto-Poto	0	0	0	1	0	0	1	0	0
	Moungali	0	0	0	1	0	0	1	0	0
	Ouenzé	0	0	0	1	0	0	1	0	0
	Talangai	0	0	0	1	0	0	1	0	0
	Mfilou-Ngamaba	0	1	0	1	0	0	1	0	0
	Madibou	0	1	0	1	0	0	1	0	0
	Djiri	0	0	0	1	0	0	1	0	0

1 : endémique (pour la LF, l'onchocercose, la schistosomiase et les GH, il s'agit des districts éligibles à la DMM) ;0 : non endémique ; 2 : cartographie à raffiner ou compléter *Nb : La situation ne prend pas en compte la nouvelle division sanitaire de 2018*

Mise en œuvre du Plan Directeur de lutte contre les MTN

Le Ministère de la Santé et de la population a créé quatre programmes de lutte contre les MTN que sont

- Le Programme National de Lutte contre l'Onchocercose et les autres Filarioses (PNLO)
- Le Programme National de Lutte contre la Schistosomiase et les Géohelminthiases (PNLSCH)
- Le Programme National de lutte contre la Trypanosomiase Humaine Africaine (PNLTHA)
- Le Programme National de lutte contre la Lèpre, l'Ulcère de Buruli et Pian (PNLL) ;

Tableau XII : Les interventions des Programmes de Lutte contre les MTN-CP au Congo de 2013-2017

**Maladies Tropicales Négligées**	**Date de Démarrage des activités (TDM TIDC)**	**Total des districts ciblés en 2013**	**Total des districts ciblés en 2017**	**Nbre de districts couverts en 2017**	**Population cible des districts sanitaires ciblés 2017**	**Nbre de personnes traitées en 2017**	**CT**	**CG**	**Principales stratégies utilisées**	**Principaux partenaires**
ONCHOCERCOSE	1984	16	17	17	781762	630019	80,6%	100%	TIDC+DMM, évaluation épidémiologique et entomologique	OMS/ESPEN, OPC, MDP SIGHTSAVERS Communautés
FILARIOSE LYMPHATIQUE	1984	11	12	6	615651	232725	37,80%	50%	DMM+ évaluation épidémiologique et entomologique	OMS/ESPEN, OPC, MDP. GSK, SIGHTSAVERS Communautés
SCHISTOSOMIASE	1986	6	6	6	110794 EAS	71390	64,4%	100%	DMM/IDC	OMS, PAM, UNICEF, IPHD, OPCEnd – Fund, PDM
GEO-HELMINTHIASES	1986	43	43	43	1097942	824428	75,1%	100%	DMM/IDC	OMS, PAM, UNICEF, IPHD, OPCEnd - Fund, GSK,
TRACHOME	-	-	-	-	-	-	-	-	-	-

NB dans les districts couverts par la DMM contre la FL, la couverture thérapeutique est de 80,0%

Tableau XIII : Les interventions des Programmes de Lutte contre les MTN-PCC au Congo de 2013 à 2017.

Maladies Tropicales Négligées	Date de Démarrage des activités	Population totale en 2017	Nbre cas dépistes en 2013	Nbre cas dépistes en 2017	Nombre de cas pris en charge en 2017	Taux de détection (pour 10000) en 2017	CT	Stratégie clé utilisée	Partenaires
Lèpre	1980	4584561 projection de 2013	53	204	204	0,44	100%	Dépistage des cas<br>Prise en charge des cas<br>Prévention des invalidités<br>Réhabilitation	FRF, OMS, ORDRE DE MALTE, ALM
Pian	2006	473162	0	0	0	0	0%	Dépistage des cas<br>Prise en charge des cas<br>IEC/CCC	OMS, ORDRE DE MALTE, ALM
Ulcère de Buruli	2005	787774	06	13	13	0,16	100%	Dépistage des cas<br>Prise en charge des cas<br>Prévention des invalidités<br>Réhabilitation<br>IEC/CCC	OMS, ORDRE DE MALTE, ALM, FRF
THA	1980	9321 (Population examinée)	20	15	15	16,09	100%	Dépistage actif Et Passif<br>Prise en charge thérapeutique<br>Lutte contre le vecteur de la maladie<br>La recherche	OMS, OCEAC, MSF-H, PF-DNDI, FIND

NB une campagne de dépistage et traitement du pian dans la Likouala par MSF a ***notifié 17440 cas*** *de Pian en 2012*

ANALYSE DE FFOM

Matrice des forces, faiblesses, opportunités et menaces du contexte de la lutte contre les MTN

**Forces**	**Faiblesses**
- Existence d'une politique nationale de santé incluant la lutte contre les MTN<br>- Plan national de développent sanitaire 2018-2022 incluant la lutte contre les MTN<br>- Existence d'un Coordonnateur des MTN<br>- Existence de 4 programmes qui compose les MTN<br>- Gratuité de soins pour les MTN<br>- Existence des cartographies pour la plupart des MTN<br>- Existence de distributeurs communautaires de médicaments	- Insuffisance des ressources financières allouées par le gouvernement<br>- Absence de mécanisme de coordination<br>- Insuffisance de motivation des distributeurs communautaires<br>- Insuffisance de capacité technique pour le diagnostic et la PEC de certaines MTN et leurs complications<br>- Faible collaboration multisectorielle<br>- Absence de lutte anti vectorielle intégrée<br>- Faible couverture thérapeutique pour certaines interventions<br>- Insuffisance de recherche opérationnelle<br>- Absence des indicateurs MTN dans le SNIS
**Opportunités**<br>- Existence du PND<br>- Donation des médicaments<br>- Engagement des partenaires<br>- Existence d'initiatives de lutte intégré sous régionale<br>- Disponibilité des Organisations à Base Communautaire<br>- Existence d'un service de lutte intégrée contre les vecteurs<br>- Création d'un Projet MTN pour la Région l'OCEAC	**Menaces**<br>- Endémicité transfrontalière des MTN<br>- Forte dépendance au financement extérieur<br>- Instabilité sociopolitique nationale et régionale<br>- Mouvement permanent du personnel de santé<br>- Faible renouvellement des RH admises à la retraite

I.3.2 Lacunes et priorités

Sur la base de l'analyse situationnelle du pays, du système de santé et des MTN, les forces, faiblesses, opportunités et menaces sont listées dans le tableau ci-dessus :

Sur la base de l'analyse de forces, faiblesses, opportunités et menaces, les priorités du programme sont :

- Renforcement de l'appropriation du programme par le gouvernement ;
- Mettre en place les mécanismes de coordination MTN
- Renforcement de la collaboration multisectorielle et du partenariat ;
- Renforcement des capacités des ressources humaines ;
- mise à l'échelle des interventions de traitement de MTN.
