# Supplementary material for: Historical overview and geographical distribution of neglected tropical diseases amenable to preventive chemotherapy in the Republic of the Congo: A systematic review
Source: PLoS Negl Trop Dis. 2022 Jul 11;16(7):e0010560. doi: 10.1371/journal.pntd.0010560 (PMC9302787; doi:10.1371/journal.pntd.0010560)
Supplement: S8 Appendix — (DOCX) [file pntd.0010560.s008.docx]

**S2. Results of the joint mapping of LF and Loaisis at the national level of the RoC, 2015.** Data come from the National Programs for onchocerciasis control.

| **Department** | **Health District** | **Administrative District** | **Village** | **Examined people, n** | **Positive LF cases (ICT+), n** | **Confirmed cases by night, n*** | **Loaisis positive cases, n** | **LF prevalence rate (%)** | **Loaisis prevalence rate (%)** | **Loaisis maximum load** |
| --- | --- | --- | --- | --- | --- | --- | --- | --- | --- | --- |
| **Cuvette Ouest** | Mbomo - Kelle - Etoumbi | Mbomo | Bossouaka | 32 | 1 | 0 | 5 | 3.1 | 15.6 | 12 200 |
|  |  | Kelle | Akombo | 34 | 0 | - | 20 | 0 | 58.8 | 61 200 |
|  |  |  | Ndouba | 56 | 0 | - | 19 | 0 | 33.9 | 68 000 |
|  | Ewo-Okoyo | Ewo | Vaga | 93 | 0 | - | 2 | 0 | 2.1 | 4 940 |
|  |  |  | Opigui | 113 | 0 | - | 1 | 0 | 0.8 | 8 260 |
|  |  |  | Alleme | 46 | 0 | - | 6 | 0 | 13 | 420 |
| **Sangha** | Ouesso | Mokeko | Attention | 53 | 0 | - | 22 | 0 | 41.5 | 19 800 |
|  |  |  | Liouesso | 46 | 0 | - | 9 | 0 | 17.3 | 14 500 |
|  |  |  | Nouveau Village | 58 | 0 | - | 26 | 0 | 44.8 | 33 400 |
|  | Sembe-Souanke | Sembe | Menguelakoum | 43 | 0 | - | 16 | 0 | 37,2 | 31 700 |
|  |  |  | Goa | 50 | 0 | - | 9 | 0 | 18.3 | 4 500 |
|  |  | Souanke | Mediao | 50 | 0 | - | 20 | 0 | 40 | 29 300 |
| **Pool** | Goma Tsé-Tsé | Goma Tsé-Tsé | Linzolo | 105 | 0 | - | 8 | 0 | 7.6 | 10 860 |
|  |  |  | Loumou | 69 | 0 | - | 0 | 0 | 0 | - |
|  | Mindouli | Mindouli | Kindamba-Ngouéri | 94 | 0 | - | 0 | 0 | 0 | - |
|  |  |  | Kinkoumba | 86 | 0 | - | 0 | 0 | 0 | - |
|  |  |  | Kindzoundou | 61 | 0 | - | 1 | 0 | 1.6 | 1 580 |
|  | Kindamba | Vinza | Mbakou Maboundou | 65 | 0 | - | 1 | 0 | 1.5 | 1 600 |
|  |  | Kindamba | Loukouo | 66 | 0 | - | 3 | 0 | 4.5 | 2 400 |
|  |  |  | Kilebe Moussia | 52 | 0 | - | 1 | 0 | 1.9 | 5 000 |
|  | Kinkala-Boko | Loumo | Badimossi | 53 | 0 | - | 0 | 0 | 0 | - |
|  |  | Boko | Nziéto | 58 | 0 | - | 1 | 0 | 1.7 | 1 680 |
|  |  | Louingui | Mangala | 58 | 0 | - | 0 | 0 | 0 | - |
|  |  | Kinkala | Matoumbou | 57 | 0 | - | 1 | 0 | 1.7 | 500 |
| **Plateaux** | Gamboma | Gamboma | Angouere | 63 | 0 | 0 | 0 | 0 | 0 | 0 |
|  |  |  | Ntsou | 70 | 0 | 0 | 5 | 0 | 7.1 | 3960 |
|  |  |  | Akana | 61 | 0 | 0 | 0 | 0 | 0 | 0 |
|  | Abala | Abala | Ekouassende | 52 | 0 | 0 | 5 | 0 | 9.6 | 5880 |
|  |  |  | Nkoulou | 56 | 0 | 0 | 6 | 0 | 10.7 | 4620 |
|  |  |  | Ngouene | 61 | 0 | 0 | 14 | 0 | 23 | 9640 |
|  | Djambala-Lekana | Djambala | Etsouali | 101 | 0 | 0 | 1 | 0 | 1 | 440 |
|  |  |  | Kiale | 55 | 0 | 0 | 0 | 0 | 0 | 0 |
|  |  | Lekana | Kebara | 57 | 0 | 0 | 1 | 0 | 1.8 | 140 |
| **Cuvette** | Owando | Owando | Moundzeli | 60 | 0 | 0 | 10 | 0 | 16.7 | 7120 |
|  |  |  | Kouyou-Ngandza | 73 | 0 | 0 | 7 | 0 | 9.6 | 6520 |
|  |  | Makoua | Tsiako | 42 | 0 | 0 | 0 | 0 | 0 | 0 |
|  | Oyo | Ngoko | Ngoko | 55 | 0 | 0 | 0 | 0 | 0 | 0 |
|  |  |  | Okia | 32 | 1 | 1 | 0 | 3.1 | 0 | 0 |
|  |  | Tchikapika | Illanga | 51 | 0 | 0 | 0 | 0 | 0 | 0 |
|  | Mossaka-Loukolela | Loukolela | Matoko | 60 | 0 | 0 | 0 | 0 | 0 | 0 |
|  |  | Mossaka | Konda | 26 | 0 | 0 | 0 | 0 | 0 | 0 |
|  |  |  | Mossaka | 79 | 0 | 0 | 2 | 0 | 2.5 | 5000 |
| **Likouala** | Impfondo | Impfondo | Bokata | 76 | 0 | 0 | 1 | 0 | 1.3 | 540 |
|  |  | Epena | Botala | 51 | 4 | 0 | 0 | 7.8 | 0 | 0 |
|  |  | Dongou | Motaba | 53 | 0 | 0 | 1 | 0 | 1.9 | 960 |
|  | Betou | Enyelle | Mimbelly | 50 | 0 | 0 | 1 | 0 | 2 | 760 |
|  |  | Betou | Bissambi | 70 | 0 | 0 | 3 | 0 | 4.3 | 1100 |
|  |  |  | Boyele port | 49 | 0 | 0 | 1 | 0 | 2 | 520 |
| **Bouenza** | Loutete | Mfouati | Mfouati | 75 | 0 | 0 | 0 | 0 | 0 | 0 |
|  |  | Yamba | Mfila | 80 | 1 | 0 | 1 | 1.25 | 1.25 | 1 360 |
|  | Nkayi-Loudima | Loudima | Mouindi | 89 | 0 | 0 | 0 | 0 | 0 | 0 |
|  |  | Kayes | Kimpalanga | 73 | 0 | 0 | 0 | 0 | 0 | 0 |
| **Lékoumou** | Sibiti | KOMONO | MBAYA | 36 | 0 | 0 | 9 | 0 | 25 | 14 100 |
|  |  | SIBITI | OUANDZI | 36 | 0 | 0 | 5 | 0 | 13.89 | 12 246 |
|  | Zanaga | ZANAGA | TONGO | 48 | 0 | 0 | 7 | 0 | 14.29 | 24 000 |
|  |  | ZANAGA | OGOUE | 42 | 0 | 0 | 11 | 0 | 26.19 | 11 600 |
| **Niari** | Mossendjo | Mayoko | Mbaka | 70 | 0 | 0 | 12 | 0 | 17.14 |  |
|  |  | Moutamba | Itsotso | 70 | 0 | 0 | 15 | 0 | 21.43 |  |
| **Kouilou** | Madingou-Kayes | Madingo-Kayes | Goudou-makanda | 50 | 0 | 0 | 13 | 0 | 26 |  |
|  |  | Kakamoeka | Manzi | 66 | 0 | 0 | 11 | 0 | 16.67 |  |
|  | Hinda-Mvouti | Mvouti | Tchivala | 51 | 0 | 0 | 51 | 0 | 100 |  |
|  |  | Hinda | Ndembouanou | 79 | 0 | 0 | 79 | 0 | 100 |  |
| **S/Total rural** | **23** | **43** | **63** | **3766** | **7** | **1** | **443** | **0.2** | **11.76** | **68000** |
| **Pointe Noire** | E.P.Lumumba | 101 | Q.101 | 42 | 0 | 0 | NA | 0 | NA | NA |
|  |  | 106 | Q.106 | 57 | 0 | 0 | NA | 0 | NA | NA |
|  | Mvoumvou | 202 | Q.202 | 105 | 0 | 0 | NA | 0 | NA | NA |
|  |  | 203 | Q.203 | 33 | 0 | 0 | NA | 0 | NA | NA |
|  | Ntie-tie | 305 | Q.305 | 100 | 0 | 0 | NA | 0 | NA | NA |
|  |  | 312 | Q.312 | 78 | 0 | 0 | NA | 0 | NA | NA |
|  | Louandjili | 401 | Q.401 | 107 | 0 | 0 | NA | 0 | NA | NA |
|  |  | 407 | Q.407 | 59 | 0 | 0 | NA | 0 | NA | NA |
|  | Mongo-Poukou | 501 | Q.501 | 55 | 0 | 0 | NA | 0 | NA | NA |
|  |  | 505 | Q.505 | 17 | 0 | 0 | NA | 0 | NA | NA |
|  | Ngoyo | 601 | Q.601 | 82 | 0 | 0 | NA | 0 | NA | NA |
|  |  | 604 | Q.604 | 102 | 0 | 0 | NA | 0 | NA | NA |
| **Brazzaville** | Makélékélé | Makélékélé | Ngassa Mayoma | 76 | 0 | 0 | NA | 0 | NA | NA |
|  | Makélékélé | Makélékélé | Kinsoundi | 58 | 0 | 0 | NA | 0 | NA | NA |
|  | Bacongo | Bacongo | Mpissa (CQ29) | 94 | 0 | 0 | NA | 0 | NA | NA |
|  | Bacongo | Bacongo | Mbama (CQ23) | 111 | 0 | 0 | NA | 0 | NA | NA |
|  | Poto-Poto | Poto-Poto | CQ33 | 55 | 0 | 0 | NA | 0 | NA | NA |
|  |  | Poto-Poto | CQ31 | 51 | 0 | 0 | NA | 0 | NA | NA |
|  | Moungali | Moungali | CQ46 | 67 | 0 | 0 | NA | 0 | NA | NA |
|  |  | Moungali | CQ49 | 68 | 0 | 0 | NA | 0 | NA | NA |
|  | Ouenzé | Ouenzé | CQ52 | 95 | 0 | 0 | NA | 0 | NA | NA |
|  |  | Ouenzé | CQ58 | 27 | 0 | 0 | NA | 0 | NA | NA |
|  | Talangaï | Talangaï | CQ63 | 31 | 0 | 0 | NA | 0 | NA | NA |
|  |  | Talangaï | CQ65 | 92 | 0 | 0 | NA | 0 | NA | NA |
|  | Mfilou-Ngamaba | Mfilou Ngamaba | Indzouli | 74 | 0 | 0 | NA | 0 | NA | NA |
|  |  | Mfilou Ngamaba | Moutabala | 59 | 0 | 0 | NA | 0 | NA | NA |
|  | Madibou | Madibou | Poto-Poto Djoué | 112 | 0 | 0 | NA | 0 | NA | NA |
|  | Madibou | Madibou | Kombé | 96 | 0 | 0 | NA | 0 | NA | NA |
|  | Djiri | Djiri | Manyanga (CQ906) | 34 | 0 | 0 | NA | 0 | NA | NA |
|  |  | Djiri | CQ902 | 46 | 0 | 0 | NA | 0 | NA | NA |
| **S/Total urbain** | **16** | **16** | **32** | **2083** | **0** | **0** | **NA** | **0** | **NA** | **NA** |
| **Total pays** | **39** |  | **95** | **5849** | **7** | **1** | **443** | **0.19** | **7.57** | **68000** |

*by thick calibrated drop
