# Supplementary material for: Historical overview and geographical distribution of neglected tropical diseases amenable to preventive chemotherapy in the Republic of the Congo: A systematic review
Source: PLoS Negl Trop Dis. 2022 Jul 11;16(7):e0010560. doi: 10.1371/journal.pntd.0010560 (PMC9302787; doi:10.1371/journal.pntd.0010560)
Supplement: S9 Appendix — (DOCX) [file pntd.0010560.s009.docx]

**Table.** Number of people (N) tested for LF in each HD selected during the 2015 nationwide survey conducted by the PNLO.

| **Department** | **HD** | **N** | **Department** | **HD** | **N** |
| --- | --- | --- | --- | --- | --- |
| Cuvette-Ouest | Mbomo-Kelle-Etoumbi | 122 | Lekoumou | Sibiti | 72 |
|  | Ewo-Okoyo | 252 |  | Zanaga | 90 |
|  |  |  |  |  |  |
| Sangha | Ouesso | 157 | Niari | Mossendjo | 140 |
|  | Sembé-Souanke | 143 | Kouilou | Madingou-Kayes | 116 |
|  |  |  |  | Hinda-Mvouti | 130 |
|  |  |  |  |  |  |
| Pool | Goma Tse-Tse | 174 |  |  |  |
|  | Mindouli | 241 | Pointe-Noire | E.P. Lumumba | 99 |
|  | Kindamba | 183 |  | Mvoumvou | 138 |
|  | Kinkala - Boko | 226 |  | Ntie-Tie | 178 |
|  |  |  |  | Louandjili | 166 |
|  |  |  |  |  |  |
| Plateaux | Gamboma | 194 |  | Mongo-Poukou | 72 |
|  | Abala | 169 |  | Ngoyo | 184 |
|  | Djambala | 213 |  |  |  |
|  | Owando | 175 | Likouala | Impfondo | 180 |
|  |  |  |  | Betou | 169 |
|  |  |  |  |  |  |
| Cuvette | Oyo | 138 |  |  |  |
|  | Mossaka-Loukolela | 165 | Brazzaville | Makelekele | 134 |
|  |  |  |  | Nfilou-Ngamaba | 133 |
| Bouenza | Loutete | 155 |  |  |  |
|  | Nkayi-Loudima | 162 |  |  |  |
