# Supplementary material for: Historical overview and geographical distribution of neglected tropical diseases amenable to preventive chemotherapy in the Republic of the Congo: A systematic review
Source: PLoS Negl Trop Dis. 2022 Jul 11;16(7):e0010560. doi: 10.1371/journal.pntd.0010560 (PMC9302787; doi:10.1371/journal.pntd.0010560)
Supplement: S10 Appendix — (DOCX) [file pntd.0010560.s010.docx]

**S3. Mass drug administration of albendazole for lymphatic filariasis, in the Republic of Congo. Source: National Program for Onchocerciasis Control**

|  | **Communities** | | | **Population** | | | **Health District** | | |
| --- | --- | --- | --- | --- | --- | --- | --- | --- | --- |
| **Years** | **Total number** | **Number of treated communities** | **Geographic coverage (%)** | **Total** | **Number of treated people** | **Therapeutic coverage (%)** | **Total** | **Number of treated HD** | **Geographic coverage (%)** |
| 2016 | 998 | 998 | 100,0 | 176 036 | 142 207 | 80,8 | 6 | 6 | 100 |
| 2017 | 998 | 980 | 98,2 | 265 070 | 206 999 | 78,1 | 6 | 6 | 100 |
| 2018 | 1 085 | 1 050 | 96,8 | 358 108 | 279 129 | 77,9 | 6 | 6 | 100 |
| 2019 | 2 069 | 2 069 | 100,0 | 653 254 | 530 232 | 81,2 | 13 | 13 | 100 |
