# Supplementary material for: Historical overview and geographical distribution of neglected tropical diseases amenable to preventive chemotherapy in the Republic of the Congo: A systematic review
Source: PLoS Negl Trop Dis. 2022 Jul 11;16(7):e0010560. doi: 10.1371/journal.pntd.0010560 (PMC9302787; doi:10.1371/journal.pntd.0010560)
Supplement: S11 Appendix — (DOCX) [file pntd.0010560.s011.docx]

**S4.** Results of the mapping of schistosomiasis and soil-transmitted helminths in the RoC in 2011

| **Department** | **Health District** | **Administrative District** | **Pop administrative district** | **Prevalence of schistosomiasis** | **Prevalence of Soil-transmitted helminths** |
| --- | --- | --- | --- | --- | --- |
| **Kouilou** | **Hinda-Mvouti** | Hinda | 37 734 | 23,3 | 67,6 |
|  |  | Mvouti | 20 685 | 30,0 | 50,6 |
|  |  | Loango | 18 995 | 3,9 | 39,1 |
|  | **Madingo Kayes** | Madingo Kayes | 15 186 | 58,8 | 61,4 |
|  |  | Kakamoeka | 8 931 | 24,1 | 58,2 |
|  |  | Nzambi | 3 585 | 0 | 68,9 |
| **Niari** | **Dolisie** | Louvakou | 15 320 | 7,3 | 55,3 |
|  |  | Kimongo | 22 369 | 4,8 | 79,0 |
|  |  | Londélakayes | 9 312 | 4,6 | 92,5 |
|  |  | Makabana | 16 182 | 0 | 37,8 |
|  |  | Dolisie | 95 743 | 0,6 | 15,1 |
|  | **Kibangou** | Kibangou | 20 493 | 0 | 66,5 |
|  |  | Divenié | 15 483 | 0 | 79,8 |
|  |  | Banda | 7 896 | 0 | 84,7 |
|  |  | Nyanga | 10 672 | 0 | 78,4 |
|  | **Mossendjo** | Mayoko | 5 880 | 0 | 48,7 |
|  |  | Moutamba | 11 866 | 0 | 73,3 |
|  |  | Mbinda | 5 466 | 0 | 45,6 |
|  |  | Moungoundou-sud | 6 517 | 0 | 53,6 |
|  |  | Moungoundou-Nord | 1 582 | 0 | 53,3 |
|  |  | Yaya | 4 348 | 0 | 53,3 |
|  |  | Mossendjo | 15 125 | 0 | 33,1 |
| **Lékoumou** | **Sibiti** | Sibiti | 53 277 | 3,0 | 67,8 |
|  |  | Komono | 16 662 | 17,8 | 75,0 |
|  |  | Mayéyé | 15 596 | 0 | 64,2 |
|  | **Zanaga** | Zanaga | 19 022 | 0 | 46,1 |
|  |  | Bambama | 5 606 | 0 | 84,7 |
| **Bouenza** | **Madingou** | Madingou | 71 775 | 0 | 29,9 |
|  |  | Boko-Songho | 14 367 | 0 | 53,3 |
|  |  | Mabombo | 13 527 | 0 | 64,2 |
|  | **Mouyondzi** | Mouyondzi | 42 066 | 23,3 | 51,1 |
|  |  | Ntsiaki | 12 927 | 0,6 | 51,4 |
|  |  | Kingoué | 13 607 | 0,6 | 64,4 |
|  | **Loutété** | Mfouati | 34 204 | 9,5 | 29,6 |
|  |  | Yamba | 16 419 | 7,8 | 52,8 |
|  | **Nkayi-Loudima** | Loudima | 37 451 | 5,0 | DND |
|  |  | Kayes | 14 994 | 9,6 | 30,9 |
|  |  | Nkayi | 81 829 | 41,4 | 03,6 |
| **Pool** | **Kinkala-Boko** | Kinkala | 39 543 | 0 | 37,2 |
|  |  | Boko | 15 588 | 0 | 21,7 |
|  |  | Louingui | 12 057 | 0 | 52,2 |
|  |  | Loumo | 5 387 | 0 | 18,3 |
|  | **Goma Tsétsé** | Goma-tsetse | 17 841 | 0 | 36,3 |
|  |  | Mbanza-Ndounga | 11 126 | 0 | 42,9 |
|  | **Mindouli** | Mindouli | 61 223 | 0,3 | 33,5 |
|  | **Kindamba** | Kindamba | 19 762 | 0 | 38,9 |
|  |  | Vindza | 6 754 | 0 | 34,1 |
|  |  | Kimba | 5 159 | 0 | 40,2 |
|  | **Ignié** | Mayama | 8 039 | 0 | 36,1 |
|  |  | Ignié | 33 466 | 0 | 62,8 |
|  |  | Ngabe | 34 388 | 0,6 | 43,3 |
| **Plateaux** | **Djambala-Lékana** | Djambala | 19 732 | 0 | 72,2 |
|  |  | Lekana | 19 150 | 0 | 78,0 |
|  |  | Mbon | 3 614 | 0 | 21,1 |
|  |  | Mpouya | 10 607 | 0 | 71,8 |
|  |  | Ngo | 19 138 | 0 | 66,9 |
|  | **Gamboma** | Gamboma | 49 391 | 0 | 43,6 |
|  |  | Makotimpoko | 21 431 | 0 | DND |
|  |  | Ongogni | 13 920 | 0 | 69,9 |
|  | **Abala** | Abala | 12 906 | 0 | 09,4 |
|  |  | Allembe | 5 301 | 0 | DND |
|  |  | Ollombo | 24 309 | 0 | 15,0 |
| **Cuvette** | **Mossaka-Loukoléla** | Mossaka | 29 298 | 0 | 20,0 |
|  |  | Loukolela | 24 500 | 0,6 | 37,7 |
|  | **Owando** | Owando | 45 936 | 0,6 | 05,0 |
|  |  | Makoua | 25 055 | 0 | DND |
|  |  | Ntokou | 4 656 | 0 | 11,2 |
|  |  | Ngoko | 3 276 | 0 | 17,3 |
|  | **Oyo** | Boundji | 18 318 | 0 | 08,9 |
|  |  | Oyo | 20 517 | 0 | 01,6 |
|  |  | Tchikapika | 6 830 | 0 | 16,1 |
| **Cuvette-Ouest** | **Ewo** | Ewo | 23 076 | 0 | 56,1 |
|  |  | Okoyo | 11 436 | 0,6 | 44,7 |
|  |  | Mbama | 9 186 | 0 | 26,1 |

| **Département** | **District sanitaire** | **District administratif** | **Pop District administratif** | **Prévalence de la Schisto** | **Prévalence des Géo Helminthiases** |
| --- | --- | --- | --- | --- | --- |
| **Cuvette-Ouest (suite)** | **Etoumbi** | Kelle | 17 377 | 0 | 37,2 |
|  |  | Mbomo | 8 185 | 0 | 57,5 |
|  |  | Etoumbi | 14 154 | 0 | 40,0 |
| **Sangha** | **Sembé-Souanké** | Sembé | 10 913 | 0 | 81,8 |
|  |  | Souanké | 11 096 | 0 | 84,5 |
|  |  | Ngbala | 5 271 | 0 | 63,5 |
|  | **Ouesso** | Pikounda | 4 014 | 0 | 68,9 |
|  |  | Mokeko | 34 550 | 0 | 45,0 |
|  |  | Ouesso | 32 220 | 0,6 | 36,9 |
| **Likouala** | **Impfondo** | Impfondo | 54 809 | 0 | 24,4 |
|  |  | Epena | 19 992 | 0 | 45,8 |
|  |  | Dongou | 22 127 | 0 | 32,2 |
|  |  | Liranga | 12 895 | 0 | 29,4 |
|  |  | Bouaniela | 8 093 | 0 | 36,1 |
|  | **Betou** | Betou | 33 989 | 0 | 34,4 |
|  |  | Enyelle | 24 214 | 0 | 31,7 |
| **Pointe-Noire** | **Lumumba** | Lumumba | 124 499 | 1,7 | 11,7 |
|  | **Mvoumvou** | Mvoumvou | 99 745 | 1,2 | 12,0 |
|  | **Tie-Tie** | Tie-Tie | 292 047 | 0 | 21,1 |
|  | **Loandjili** | Loandjili | 296 165 | 3,4 | 12,8 |
| **Brazzaville** | **Makélékélé** | Makélékélé | 341 247 | 0 | 06,8 |
|  | **Bacongo** | Bacongo | 112 990 | 0,7 | 02,7 |
|  | **Poto-Poto** | Poto-Poto | 106 403 | 0 | 01,7 |
|  | **Moungali** | Moungali | 190 813 | 1,0 | 07,0 |
|  | **Ouenzé** | Ouenze | 208 133 | 0,3 | 10,6 |
|  | **Talangaî** | Talangaî | 386 699 | 0 | 06,4 |
|  | **Mfilou** | Mfilou | 224 909 | 0 | 11,0 |
| **Congo** | **39** | **101** | **4 222 164** |  |  |
