# Supplementary material for: Historical overview and geographical distribution of neglected tropical diseases amenable to preventive chemotherapy in the Republic of the Congo: A systematic review
Source: PLoS Negl Trop Dis. 2022 Jul 11;16(7):e0010560. doi: 10.1371/journal.pntd.0010560 (PMC9302787; doi:10.1371/journal.pntd.0010560)
Supplement: S12 Appendix — (DOCX) [file pntd.0010560.s012.docx]

**S5. Mass drug administration of albendazole for soil-transmitted helminths, in the Republic of Congo. Source: National Program for Onchocerciasis Control**

|  | **Population** | | |
| --- | --- | --- | --- |
| **Years** | **Total** | **Number of treated people** | **Therapeutic coverage (%)** |
| 2014 | 5 133 | 4 373 | 85,2 |
| 2015 | 343 462 | 248 323 | 72,3 |
| 2016 | 26 846 | 22 900 | 85,3 |
| 2017 | 962 242 | 740 926 | 77,0 |
| 2018 | 1 071 167 | 896 567 | 83,7 |
