# Supplementary material for: Historical overview and geographical distribution of neglected tropical diseases amenable to preventive chemotherapy in the Republic of the Congo: A systematic review
Source: PLoS Negl Trop Dis. 2022 Jul 11;16(7):e0010560. doi: 10.1371/journal.pntd.0010560 (PMC9302787; doi:10.1371/journal.pntd.0010560)
Supplement: S13 Appendix — (DOCX) [file pntd.0010560.s013.docx]

**S6. Mass drug administration of praziquantel for schistosomiasis, in the Republic of Congo. Source: National Program for Onchocerciasis Control**

|  | **Population** | | |
| --- | --- | --- | --- |
| **Years** | **Total** | **Number of treated people** | **Therapeutic coverage (%)** |
| 2014 | 5 133 | 4 373 | 85,2 |
| 2015 | 56 634 | 30 922 | 54,6 |
| 2016 | 26 846 | 22 900 | 85,3 |
| 2017 | 110 854 | 71 390 | 64,4 |
| 2018 | 19 739 | 15 219 | 77,1 |
| 2019 | NO MDA | | |
| 2020 | 90 232 | 62 350 | 69,1 |
