# Supplementary material for: Historical overview and geographical distribution of neglected tropical diseases amenable to preventive chemotherapy in the Republic of the Congo: A systematic review
Source: PLoS Negl Trop Dis. 2022 Jul 11;16(7):e0010560. doi: 10.1371/journal.pntd.0010560 (PMC9302787; doi:10.1371/journal.pntd.0010560)
Supplement: Attachment — Submitted filename: Cover_Letter_Review_NTD_CP_Plos_NTD.docx [file pntd.0010560.s014.docx]

Editor-in-chief

PLoS Neglected Tropical Diseases

January the 10, 2022

Dear Editor-in-chief,

We hereby submit the revised version of our manuscript entitled “Historical overview and geographical distribution of Neglected Tropical Diseases amenable to preventive chemotherapy in the Republic of the Congo: a systematic review” (PNTD-D-21-01433). We thank the two reviewers for their positive comments and appreciation of the paper.

We addressed each of the reviewers’ comments with detailed responses. We hope our manuscript will now meet the standards for publication.

Best regards,

Cédric Chesnais

I. **Reviewer 1:**

All line numbers for the changes correspond to the line numbers of the new clean version, without the apparent changes.

The paper is a systematic review that has the appropriate methodology and design. The exclusion criteria are laid out clearly. However, the inclusion criteria needed to be more detailed. The identification of records, screening were well described and illustrated in figure 1. The final number of records reviews is less than 100 but has yielded substantial power and data that has been properly processed and analyzed. There were no ethical issues associated with the systematic review. The structure of the paper should be revised to insert the disease descriptions together at the beginning -mainly as a sub-section of the introduction.

Response. We thank the reviewer for this positive comment.

Regarding the inclusion criteria, we decided to include all the community-based epidemiological studies conducted in the RoC with available data; as well as unpublished reports of the national control programs or doctoral theses. We did not apply other inclusion criteria, but rather a number of exclusion criteria, as presented in the document.

Regarding a new sub-section of the introduction, we think that the introduction is already relatively long (75 lines); we prefer add “In this context, we conducted a monograph-type historical review on the five PC-NTDs in the RoC, for which a brief disease description is presented in the results section,”

Editorial and Data Presentation Modifications

There are some editorial modification that need consideration:

1. Line 29: The qualification “surprising” might not be accurate because NTDs are not well known and are widely documented. This mention of “surprise” is unnecessary and should be removed. We removed “surprising”
2. Line 33 -34: Like “LF” the acronym “STH” should be included because they are used later in the text. We deleted the “LF” acronym as well as “STH” to decrease the number of acronyms in the abstract. We started when necessary the acronyms in the main text.
3. Line 42: Please clarify what “LF cases” means.  It is referring the LF antigenemia or LF morbidity cases/ Please be more specific. In the abstract, “LF cases” refers to both *W.* *bancrofti* antigenaemia and microfilaraemia presence. We added “lymphatic cases (based on *Wuchereria bancrofti* antigenaemia and/or microfilaemia)”. Later in the text, one of the two terms was used.
4. Line 45: Please specify if the prevalence of trachoma in the Likouala department. Was the prevalence below 5% TF? Yes, it was. In the text, we added “at the national level (< 5%), but still present (>5%)”.
5. Line: 46-47 – The sentence - However, the overall number of publications was low for the period under study and remained so in recent years – it does not fit well in the conclusion section. It should be better moved up in the finding section. The sentence was moved to the findings section. We modified the sentence “Despite a low number over the studied period, since 2005 there has been a steady increase in the number of publications”
6. Line 80: - Neglected Tropical Diseases (NTDs) are a group of primarily communicable and parasitic tropical diseases - Please note here that Trachoma is not a parasitic disease. It is bacterial. The sentence should be amended. We removed “parasitic”.
7. Line: 88: “symptoms” and “clinical signs” look redundant; please stick with one of them. We removed “symptoms and”.
8. Line 107: The figures here do not add up. The total of the numbers shows less than 100,300 deaths. We replaced by “approximately 100,000” individuals.
9. Line 140: Please change “on” by “about”. We replaced “on” by “about”
10. Line 145: Method: It is clear if the non-PC NTDs were excluded from the method. Please clarify. On line 163, we added “We excluded”. We deleted “were excluded from the study”. On line 164, we added “non PC-NTDs-related studies”
11. Line 147: Please specify the starting date and time. We added “The exhaustive literature search was conducted from July 4, 2019, to July 4, 2019 and identified eligible references published from 1914 to 2019.”
12. Line: 175: The sentence is in the past. Please correct. We removed “will” and added “present”. Indeed, the statement is about what is being done in the manuscript, not what the manuscript did.
13. Line 189: The method section does not mention inclusion criteria. In line 158, we added “chose as inclusion criteria” and removed “included”
14. Line 202: “and then” please remove the “and”. We removed “and”
15. Line 207 and 210: Please spell out the acronyms ‘PNLO” and “PNLSCH” as they appear for the first time in the manuscript. PNLSCH was spelt out in line 207. PNLO was spelt out in line 179.
16. Line 210-211: The sentence is confusing; please rephrase. We rephrased as follows : “From 1984 to 1991, the PNLO conducted epidemiological and entomological surveys to identify onchocerciasis foci”
17. Line 236: Please remove “as well as” and place by ‘and”. We replaced “as well as” by “and”
18. Line 264: Please insert a reference. The references begin on line 274. This was an introductory sentence to several studies. The beginning was just an introductory sentence of a series of articles.
19. Line 267: Please provide specific data instead of “very low prevalence”. We added “at very low prevalence as presented in the Table 1”
20. Line 276: “PNod” please spell out. PNod is an unusual acronym for nodule prevalence; please change it throughout the manuscript. PNod was already spelt out in line 172. We added a list of abbreviations in the S1 Supplementary Materials. In addition, we intentionally chose “prevalence of nodules” (PNod) and “prevalence of skin microfilariae” (PMF) for onchocerciasis, instead of “cystic index” and “microfilariae index”, respectively. The reason was to clarify that these were onchocerciasis nodules (and not simple cysts), for PNod – (we added “PNod (also wrongly called “cystic index”)). But we let the final decision to the editor for modifying these acronyms.
21. Line 276: “PMF” – What does this stand for? Please spell out. PMF was already spelt out in line 172: prevalence of skin microfilariae.
22. Line 301: Please change “of” by “with”. We replaced “of” by “with”.
23. Line 329: Please replace “serious’ by “severe” for adverse events. We replaced “serious” by “severe”.
24. Line 333: Please provide a reference – Please note that high level loiasis microfilaria might still be a significant risk of severe adverse events for onchocerciasis MDA. Reference provided in line 338.
25. Line 365 and 368: Please insert references. Figure 5 serves as a reference as stated with the sentence “Results are presented in Figure 5”.
26. Line 396: Please clarify if this is “therapeutic coverage”. Yes, it is. We replaced “treatment” by “therapeutic”.
27. Line 423: Please remove “possible” before ‘presence’. We removed “possible”.
28. Line 456, 470, and 473: Please include references. Reference for lines 456 to 468 was added in line 449 ([74]: Missamou F, Pion SD, Louya F, Badila C, Fischer PU, Weil GJ, Boussinesq M: First parasitologic evidence of a focus of lymphatic filariasis in the Republic of Congo, 60th annual meeting of the American Society of Tropical Medicine and Hygiene, Late-breaker abstract LB-2343; 2011). For line 470, we do not understand which reference we can add. For line 473, we have now written “PNLO reports”.
29. Line 499: HD and IU are used interchangeably; please stick with one. We removed HD.
30. Line 504: Table 4 - This table is not indispensable. The treatment schedule is already described in the text. We moved this table as Supplementary Material.
31. Line 524: Strongyloidiasis is not commonly cited as part of the main STH. The STH here should stick to hookworm, Ascaris and Trichiuris. We thank the reviewer for this comment. Strongyloidiasis is included in the group of STH in the WHO roadmap 2021-2030 (see our ref 1).
32. Line 631-632: Please clarify that this guidance applies only to the inception of a control program from baseline mapping. In moderate and high-risk areas, praziquantel is applied once and twice a year, respectively. Hence, this is the recommendation in a treated population; and not just after a baseline mapping.
33. Line 660 – 663: This sentence looks like an expert opinion made at in 1964. It might be more relevant and useful to provide data and evidence. We corrected by “The author could not confirm the endemicity of intestinal schistosomiasis (due to *S. mansoni*) in the RoC, despite a few reported cases from the Prefecture of Djoué (i.e., in the Brazzaville area)”
34. Line 637: When was the schistosomiasis program created? Please clarify. The date first appeared in line 135. We added it in line 678.
35. Line 731: Please add that blinding trachoma is targeted for elimination by WHO. We added : “and the WHO now targets its elimination as a public health problem by 2030”
36. Line 749: Please remove “included” in the title of table 8. This mention is not necessary. We removed “included”
37. Line 782-783: Please clarify if “low number of cases” refers to LF antigenemia or morbidity cases. We modified the sentence with “very low numbers of antigenemia-positive individuals at national level”.
38. Line 793:  The authors should note that there has been no nationwide mapping of trachoma in RoC. No desk review and no trachoma rapid assessment were done in suspected areas. We added “Additional trachoma rapid assessment surveys should also be conducted in other departments where the disease could be present”.
39. Lines 799 to 811: The paragraph should be moved up next to the section of the disease description. We believe this short paragraph better fits the discussion section, since we wanted to report on similar work.
40. Lines 812 -826: This section should be called “limitation of the review”. We added “As a limitation of this review, we have encountered…” at the beginning of this section.
41. Line 823: It should be noted that now serological tests (OV16) are more sensitive for the mapping and might provide different endemicity prospects than the nodules and microfilaremia used in the past. We added “Current mapping of onchocerciasis and LF in central Africa should be interpreted with caution in *L. loa* co-endemic areas. Furthermore, mapping should jointly assess *L. loa* microfilarial density during these surveys. Alternatively, Ov16 RDT could be key to addressing false positive skin snips related to loiasis for mapping of onchocerciasis”.
42. Figure 1 to 9 are titles missing. Titles of each figures are present as a “caption” in the main text, as recommended by Plos NTD.

In the results part:

The results need to be restructured to strictly emphasize the endemicity of the PC-NTDs and provide a good understanding of each disease's extent. The results section should focus more on actual disease prevalence data and trends rather than the availability of publications and the review processes. There are significant data on treatments that are unnecessary. The paper should be focused more on the geographical distribution and historical data - as stated in the title.

Response: As indicated by the reviewer, the objective of this review was to present all existing epidemiological data in RoC; this implies a geographical and historical approach. For this reason, we aimed to follow a precise plan: 1- The flow chart, to show the process of selecting the papers chosen for the review; 2- The distribution of the selected papers over the years of the study; 3-History of national PC-NTD programs; and 4-Description of each PC-NTD (with subheadings: clinical presentation, etc.). We believe that this review presents a historical overview while taking into account the significantly relevant periods according to the diseases, with illustrations and maps accordingly. Concerning tables 1 and 3 (on treatment data), we put them as supplementary material to lighten the reading of the main document and in the text.

On line 347 we added “and the population size” and on line 473, we added “In S7 Appendix we reported results of the survey and in S8 Appendix the number of tested individuals”.

Some tables are poorly presented, many are of poor quality. Most tables should be reformatted because the characters are small and difficult to read. They are long tables and are spread over multiple pages example, tables 2,5, 6, and 7.

Response: We put a police size of 11, where it was 10. We reformatted all the tables.

The onchocerciasis section would have been more helpful to present the data using nodules prevalence, CMFL, and skin snip microfilaremia.

Response: The prevalence of nodules was mapped (Fig. 4 and S3 Appendix) for all the villages studied at the national level in the REMO survey, which only used the PNod (or cystic index) indicator into account for mapping onchocerciasis. Between 2011 and 2015, this prevalence was again assessed in the formerly meso- and hyper-endemic areas (Fig. 5).

Data on CMFL were presented when available.

In the conclusions section:

This systematic review is of interest to the general public because it provides records and endemicity data to understand the extend of NTDs in RoC. For example, the information concerning the presence of loiasis in areas endemic for onchocerciasis or Lymphatic Filariasis, and the potential for severe adverse events is critical. The geographical distribution of the diseases provides valuable information that the national NTD programs could use. Research can use the data for modeling.

Response: we thank the reviewer for this comment.

Some statements (line 638) are more speculative since no study was done to ascertain that the disease was imported from other countries. In addition, it is unlikely that observations in 1953 (line 638) would validate a statement from 1920 (line 635). The first sentence of the conclusion is not in line with the finding, which notes that Trachoma was not considered endemic at the national level.  Although the last section of the paper eludes to some significant limitations that the authors should bring up, there is no limitation section.

Response:

We modified “This argument is based on “ with “This hypothesis is based on”.

The limitation section begins at line 803: “As a limitation of this review,…”.

Indeed, we had written in the abstract that trachoma is non-endemic at a national level but still present in the Likouala department and we stated in the first sentence of the conclusion that PC-NTDs are endemic in the RoC. We modified the first sentence of the conclusion with “Except for trachoma, whose epidemiological situation should be better evaluated, PC-NTDs are endemic in the RoC”.

In the summary and general comments:

The paper should provide a summary of NTD prevalence and epidemiological situation. But, Instead, it focuses on the availability of publications and literature. It does not focus enough on the NTD situation.

Response: We have developed maps showing, to the extent that data are available, the earliest and most recent status of each NTD. Furthermore, in the summary section, summarizing the prevalence of NTDs and the epidemiological situation in a few words seems very difficult.

II. **Reviewer 2:**

This is a well researched review article with clear objectives in the introduction which have been achieved by the authors

Editorial and Data Presentation Modifications

This is a long article and for those unfamiliar with some of the subjects in might be good to have a list of abbreviations to avoid searching back through the document to understand it.
Response: we added the list of abbreviations as Appendix 1. In line 182, we added the introductory sentence for it: “To facilitate the reading of the abbreviations, a list was provided (S1 Appendix)”

With regard to Onchocerciasis different authors use different descriptive terms. I think PNod, presumably Nodule Prevalence and PMF, microfilaria prevalence, not density or CMFL could be more clearly defined.
*Loa* has been mentioned, it might also have been good to add an old reference to spontaneous *Loa* encephalopathy in one of the places you have discussed this.

Response: CMFL was only defined in the Table 1, and PNod and PMF were defined at line 172.

Regarding *Loa* encephalopathy, we added “Even if loiasis is not currently included in the WHO’s list of NTDs, reports suggest that it can induce spontaneous encephalopathy [Lukiana et al. Filaria Journal 2006] and a study in Cameroon suggested that it can induce excess mortality [Chesnais et al., LID]. As loiasis is highly endemic in the RoC, morbidity and mortality related to this disease might be worth investigating in this country”.

In line 613 I would add the risk of HIV infection in female urogenital schistosomiasis.

Response: we added it, in line 603.

In the results part:

There is a wealth of material that has been researched from the literature. This has been clearly presented in detail both in descriptive sections and particularly the tables. Figures 2 and 8 could be presented with a little more contrast to make it easier to read but they can be read when downloaded.

Response: we thank the reviewer for its positive comment. For Figures 2 and 8, as noted by the reviewer, once downloaded correspond to the quality recommended by Plos NTD. We leave to the Editor to decide whether modifications of these figures are needed.

In the conclusions section:

The authors propose that this information would be useful for all those planning to work on NTDs in Congo and it is an excellent summary. There are suggestions for filling some of the gaps, particularly where better diagnostics could help. This would be a very useful public health resource.

Response: we thank the reviewer for this comment.

In the summary and general comments:

This is a well-researched article and contains much information and very useful for anyone working in the Republic of Congo. I think it will prove to be a useful resource.

I think this would be of interest to those working in Congo but not of a general interest to warrant special media coverage.

Response: we thank the reviewer for this comment.
