# Supplementary material for: Historical overview and geographical distribution of neglected tropical diseases amenable to preventive chemotherapy in the Republic of the Congo: A systematic review
Source: PLoS Negl Trop Dis. 2022 Jul 11;16(7):e0010560. doi: 10.1371/journal.pntd.0010560 (PMC9302787; doi:10.1371/journal.pntd.0010560)
Supplement: Attachment — Submitted filename: Cover_Letter_Review_NTD_CP_Plos_NTD_NGATSE.docx [file pntd.0010560.s015.docx]

*Unité Mixte Internationale TransVIHMI*

*UMI233 IRD – U1175 INSERM – Université de Montpellier*

Institut de recherche pour le développement

911 avenue Agropolis – BP 64501 – 34394 Montpellier Cedex 5 - France

Tél. + 33 (4) 67 41 61 62 ; Mail. cedric.chesnais@ird.fr

To Editor-in-chief

PLoS Neglected Tropical Diseases

May 2^nd^, 2022

Dear Editor-in-chief,

We hereby submit the second revised version of our manuscript entitled “Historical overview and geographical distribution of Neglected Tropical Diseases amenable to preventive chemotherapy in the Republic of the Congo: a systematic review” (PNTD-D-21-01433). We thank the two reviewers for their positive comments and appreciation of the paper.

We addressed each of the reviewers’ comments with detailed responses and hope that our manuscript will now meet the standards for publication.

Best regards,

Cédric Chesnais

I. **Reviewer 1:**

All line numbers for changes correspond to the line numbers of the new clean version, without the apparent changes.

Summary and general comments

Here below are my comments for the second review and for the responses to my first observations:

1) Line 41-42 Abstract - I am still struggling with the statement of the authors noting that "trachoma is not endemic at the national level". Trachoma is a focal disease and therefore if one or several districts are endemic (TF> 5%) then epidemiologically the country is endemic for Trachoma.  I’d suggest rephrasing to read as follows: “Trachoma is only endemic in the department of Likouala (prevalence>5%), further mapping is essential to properly assess the burden of the disease in the country”

Response: we replaced (lines 41-43) “Trachoma is now considered non-endemic at the national level (trachomatous inflammation-follicular [TF] < 5%), but still present (TF > 5%) in the Likouala department, and”

**by**

“Trachoma is endemic in the Sangha and Likouala department (prevalence of trachomatous inflammation-follicular > 5% in some villages), and further mapping is essential to properly assess the burden of this disease in the country”. We also added sentences in the text (lines 749-753) to clarify this.

In lines 43-45, we added “While the prevalence of soil-transmitted helminths is still high (over 20%) in a large part of Congo, cases of lymphatic filariasis”.

In lines 45-46, we added “and onchocerciasis are becoming rare and very focused”.

In line 46, we replaced “However, t” by “T”. Also, we deleted “other”

2) Response Line 631-632: For Schistosomiasis, the WHO 2011 Guidelines recommend that AFTER 5-6 years of treatment, in high and moderate setting, preventive chemotherapy be conducted once every two years (for areas with prev 10-50%) or two times a year (for areas with prevalence still high Prev >50%)

Response: We used the recommended WHO 2011 progress report, published in 2013 (p.5)^[1]^ and the last available data from the WHO 2022 guideline (p.23)^[2]^. Therefore, we wrote (in lines 630-633): “a 2013 WHO progress report recommended praziquantel MDA once every two years or once a year, respectively. In a more recent guideline, issued in 2022, WHO recommends praziquantel MDA once a year or twice a year for moderate and high risk districts, respectively“.

II. **Reviewer 2:**

All line numbers for the changes correspond to the line numbers of the new clean version, without the apparent changes.

Methods

The objectives are stated as a review article and in this is what has been done. More actual data in this revision has improved the paper.

Response: we thank the reviewer for this comment

Editorial and Data Presentation Modifications

Line 383: The definition of Geographical Coverage is incorrect although correctly used further in the document. For APOC the Geographical Coverage is related to Meso and Hyper endemic foci. I would suggest changing the section in brackets to (During APOC GC was the proportion…..

Response: We added “During APOC,” and replaced “is” by “was”, in line 392.

Line 435, 440 etc, it is a bit difficult to understand the administrative system and how the public health framework fits into all this.  Are health districts based on ADs.  It would help to understand if this could be explained.

Response: In lines 185-191 (methods part), we added: “In the results, we define "administrative district" (AD) as the territorial administrative subdivision immediately following the department. "Health district" (HD) corresponds to the geographic and administrative subdivision of the health system consisting of a referral hospital (or district hospital) surrounded by a network of public (health posts and centers) and private (medical and social centers, medical offices, clinics, etc.) health facilities. In the RoC, the HD corresponds to either an AD, an arrondissement or a grouping of ADs or arrondissements. ”

For this change, we added two new references in the text, in line 189^[3],^^[4]^.

In line 356, we deleted “administrative districts [” and also “]”.

In lines 357, we replaced “health district” by “HD”

Line 607.  I think this is usually described as a “point of care” test.

Response: we replaced “s” par “r” in the group of words “point-of-care”, line 617

Line 714.  Trachoma is worldwide and still present in many Asian countries. The “first” infection may be with the under 5s but as stated further it is the repeated infection that creates the problem.

Response: We added “and is always endemic in remote areas and poorest populations of Africa, Asia, Australia and the Middle East resulting in blindness” (lines 726-727). We deleted “primarily in Africa and causes blindness” (line 726).

In line 728, we added “active” and replaced “primarily” by “mainly”. In lines 728-729, we added “and its prevalence decreases with age”.

Line 719: Initially the objective was to eliminate “blinding trachoma” but now is stated as eliminating trachoma as a “public health problem”

Response: we added “blinding” (before trachoma), in line 733

Line 724: Treatment for very small children is based on tetracycline eye ointment.

Response: in lines 738-741, we added “Treatment of infants under 6 months of age is classically based on topical application of tetracycline ointment daily for 6 weeks.”

For this change, we added two new references to the text.^[5],^^[6]^

Line 817:  Could be the key “to fix” not “for fix”

Response: we replaced “for” by “to”, in line 834

Summary and general comments

A very thorough document that would be of use to someone new to NTDs in the Republic of Congo

Response: we thank the reviewer for this comment

1. World Health Organization. Schistosomiasis: progress report 2001 - 2011, strategic plan 2012 - 2020. World Health Organization; 2013. Available from: <https://apps.who.int/iris/handle/10665/78074>
2. World Health Organization. WHO guideline on control and elimination of human schistosomiasis [Internet]. World Health Organization; 2022 [cited 2022 Apr 18]. 142 p. Available from: <https://apps.who.int/iris/handle/10665/351856>
3. Tarimo E, Organization WH. Towards a healthy district : organizing and managing district health systems based on primary health care [Internet]. World Health Organization; 1991 [cited 2022 Apr 18]. Available from: <https://apps.who.int/iris/handle/10665/40785>
4. Chatora R, Tumusiime P. Health sector reform and district health systems. Brazzaville WHO. 2004. Available from: <https://staging.afro.who.int/sites/default/files/2017-06/dsdAFR-DHS-0301_0.pdf>
5. Initiative IT. Zithromax management guide 2019. Atlanta: International Trachoma Initiative n.d. Available from: <https://www.trachoma.org/sites/default/files/2020-12/ITI_ZMG_ENG_121120_Online.pdf>
6. Control IC for T. Trachoma Action Planning. International Coalition for Trachoma Control London; 2015. Available from: <https://www.trachomacoalition.org/sites/default/files/content/resources/files/ICTC%20TAP%20planning%20guide%20eng.pdf>
